# Improvement of intestinal barrier function, gut microbiota, and metabolic endotoxemia in type 2 diabetes rats by curcumin

**DOI:** 10.1080/21655979.2021.2009322

**Published:** 2021-12-19

**Authors:** Jingze Huang, Binbin Guan, Lijing Lin, Yanping Wang

**Affiliations:** Department of Endocrinology and Metabolism, Fujian Medical University Union Hospital, Fuzhou, Fujian, China

**Keywords:** T2DM, LPS, TLR4, curcumin

## Abstract

Type 2 diabetes mellitus (T2DM) is known as a complex genetic disease characterized by genetic and environmental factors. The imbalanced intestinal flora and intestinal mucosal barrier are considered to be related to T2DM. Curcumin has been proved to affect the progression of T2DM. T2DM animal was established by low-dose streptozotocin intraperitoneal injection combined with high-fat diet (HFD) feeding. Hematoxylin and eosin (HE) staining and transfer electron microscopy (TEM) were used to observe morphological changes of intestinal tissues of T2DM rats. Insulin and glucose tolerance tests were performed to investigate the influence of curcumin on blood glucose. Curcumin significantly improved the intestinal integrity, hyperglycemia and insulin resistance in diabetic rats. The metabolic endotoxemia induced by HFD in diabetic rats was inhibited remarkably. Curcumin reversed gut microbiota dysbiosis in diabetic rats caused by HFD. We demonstrated that curcumin could protect intestinal mucosal barrier, improve insulin resistance and reduce blood glucose in diabetic rats. This study might provide experimental evidence for the prevention and treatment in T2DM.

## 1. Introduction

Type 2 diabetes mellitus (T2DM) is a chronic and persistent inflammatory disease [1], and it is characterized by persistent elevated blood glucose. Currently, T2DM has become a heavy burden in the world, and the prevalence is predicted to be 13.5% in the world in 2040 [2]. Lipopolysaccharide (LPS) in the blood circulation forms a complex with CD14 of monocyte macrophages, which is recognized by toll-like receptor 4 (TLR4) on the surface of immune cells. They could activate nuclear transcription factors through myeloid differentiation molecule 88 (MyD88) [3] and promote the synthesis and secretion of many inflammatory factors including tumor necrosis factor α, interferon γ, and interleukin-6, which may further affect the insulin resistance and T2DM [4].

The pathological phenomenon that the level of LPS in blood circulation is obviously higher than that in normal is known as metabolic endotoxemia. The metabolic endotoxemia has been believed to be closely related with the development of diabetes [5]. It was reported that metabolic endotoxemia initiated obesity and insulin resistance [6].

Unhealthy lifestyle, such as high-fat and high sugar diet, can cause changes in the intestinal microenvironment (intestinal flora), trigger intestinal inflammation, destroy the tight junction structure, increase intestinal permeability and damage the intestinal mucosal barrier function [7,8]. The main mechanism of metabolic endotoxemia is the change of intestinal flora and the damage of intestinal mucosal barrier function. Regulating intestinal flora and maintaining the integrity of intestinal mucosal barrier are of great significance for T2DM patients [4].

Curcumin is a natural polyphenol compound extracted from Curcuma tuber and Curcuma rhizome. It is believed to possess the ability of anti-tumor, anti-inflammatory, antioxidant and anti-fibrosis effects [9]. Studies have shown that curcumin could inhibit nuclear factor-κB (NF-κB) mediated interleukin-1β (IL-1β) and tumor necrosis factor α (TNF-α) release in db/db diabetic mice, and further reduce the inflammatory reaction of mouse liver [10]. In obese rats fed with high-fat diet and obese mice with leptin gene deficiency, 3% curcumin can inhibit the secretion of TNF and monocyte chemoattractant protein-1 (MCP-1), improve inflammatory response, and reduce the levels of blood glucose and glycosylated hemoglobin [11,12]. Clinical trials indicated that oral curcumin can improve the function of islet B cells in prediabetic patients, reduce insulin resistance, prevent progression to type 2 diabetes [13]. Therefore, curcumin has good clinical application prospects. However, the specific mechanism of these protective effects is not fully clear. Moreover, the effect of curcumin on intestinal health of T2DM has not been discussed in detail from the perspective of intestinal mucosal barrier integrity and intestinal flora.

In this study, streptozotocin (STZ) injection combined with high-fat diet (HFD) was used to induce type 2 diabetic rats. We hypothesized that curcumin might improve intestinal barrier function, gut microbiota, and metabolic endotoxemia in type 2 diabetes rats. We aimed to investigate the morphology of intestinal mucosa, expression of TLR4/NF-κB in intestinal mucosa, intestinal mucosal barrier and intestinal flora changes after curcumin treatment. This study explored the possible mechanism of curcumin protecting intestinal mucosal barrier, improving insulin resistance and reducing blood glucose, so as to provide experimental evidence for its prevention and treatment in T2DM.

## 2. Materials and Methods

### 2.1 T2DM animal experiment

Low-dose STZ (Sigma, US) intraperitoneal injection combined with HFD feeding were used to establish type 2 diabetes rat model by destroying part of the islets β cells and insulin resistance. Thirty specificpathogen-free (SPF) organism grade male rats (Charles River Laboratories, China) were adaptively fed with basic diet for 1 week, and 20 rats were randomly selected and fed with HFD for 4 weeks. STZ (prepared with 0.01 mol/L pH 4.5 sodium citrate buffer) was injected intraperitoneally (25 mg/kg) once a week for two times. The blood glucose level was measured through tail vein blood every week. After 2 weeks, the fasting plasma glucose reached more than 11.1 mmol/L for stable 7 days, the type 2 diabetic rats model was established successfully. Ten normal rats fed with basic diet were set as normal control group. Twenty diabetic rats were randomly divided into diabetic model group and curcumin intervention group. The animals in the curcumin intervention group was given 200 mg/kg curcumin intragastric administration (prepared with 0.5% carboxymethyl cellulose sodium) at a fixed time of day. The animals in the control group and diabetic model group were given the corresponding volume of 0.5% carboxymethyl cellulose sodium for 10 weeks. During the experiment, the appetite, hair, feces and urine, body weight, behavior and overall state of rats in each group were recorded. After 10 weeks of gavage, fresh formed stool was taken by sterile forceps and placed in sterile cryopreservation tube, and stored at –80°C. After the last administration, all rats were fasting overnight, and the animals were anesthetized with ketamine (0.2 mL/100 g body weight, intraperitoneal injection). Prolonged exposure to isofluorane (Sigma, US) inhalation was performed to euthanize mice. Then, the blood samples and proximal colon of the ileocecal regions (4 cm) were collected, and stored at −80°C. All experimental protocols were approved by the Institutional Animal Care and Use Committee of Fujian Medical University Union Hospital, and all methods were carried out in accordance with relevant guidelines and regulations in manuscript. The study was carried out in compliance with the ARRIVE guidelines [14].

### 2.2 Fecal microbiota identification

The fecal DNA was amplified with different primers and diluted relatively. The standard curve between copies and cycle threshold (CT) value was made by RT-PCR. RT-PCR was carried out with the extracted DNA sample as the template. The copies of each bacterium in the sample were calculated through the standard curve, and the relative content was calculated by the ratio of gene copies encoding 16S rRNA of different bacteria to total DNA copies. 2-△△ct method was used to analyze gene relative expression. Fecal DNA was extracted by fecal DNA extraction kit (QIAamp PowerFecal DNA kit, Qiagen, US) and stored at -20℃ for analysis. The concentration of fecal DNA was detected with a NanoDrop Spectrophotometer ND-1000 (Thermo Fisher, USA). The PCR conditions were set as follows: 30 cycles at 95°C for 30 s; 56°C for 30 s; and 72°C for 45 s. The primers were listed as follows: Bacteroidetes F: GTTTAATTCGATGATACGCGAG, R: TTAASCCGACACCTCACGG; Entrobacterizies F: TAGGCTTGACATTGATAGAATC, R: CTTACGAAGGCAGTCTCCTTA; Firmioutes F: GGAGYATGTGGTTTAATTCGAAGCA, R: AGCTGACGACAACCATGCAC; Bifidobacterium F: GTCAGCTCGTGTCGTGAG, R: GTCGCATCCCGTTGTACC.

### 2.3 Morphological examination

The tissues were fixed with 4% formaldehyde solution (Beyotime, China). The tissues were dehydrated, embedded, made into wax blocks and cut into paraffin sections. After hematoxylin and eosin (HE) staining, the morphological changes were observed under a light microscope. Intestinal mucosal thickness and villus height were measured randomly. For the transfer electron microscopy (TEM) measurement, the tissues were quickly fixed in 3% glutaraldehyde solution for 2 h, then fixed in 1% osmic acid for 1 h, and dehydrated by acetone gradient. After epoxy propane replacement, epon-812 resin embedding, ultra-thin slicing, uranium acetate lead citrate double electron staining, the tight junction of intestinal epithelial cells was observed using TEM.

### 2.4 Insulin and glucose tolerance tests

At week 10, the rats were fasting overnight. Then, 0.5 g/kg glucose was administered intraperitoneally to conduct glucose tolerance test. Then, blood glucose was detected at different time points (0, 15, 30, 60, 90, and 120 min) with an UltraTouch glucometer using the whole blood taken from cut tail tips immediately. After fasting for 6 h, insulin tolerance test was performed. After intraperitoneal injection with insulin (2 IU/kg), the blood glucose was detected at different time points (0, 15, 30, 60, 90, and 120 min) with an UltraTouch glucometer using the whole blood taken from cut tail tips immediately.

### 2.5 Homeostasis model assessment of insulin resistance (HOMA-IR) detection

HOMA-IR is commonly used to measure insulin resistance. The fasting values of glucose and insulin were used to calculate HOMA-IR index. HOMA-IR index = insulin (μU/mL) × glucose (mM)/22.5.

### 2.7 2.6 Biochemical analyses

The blood was collected as described in Section 2.1. The levels of LPS (#CSB-E13066, Huamei Bio, Wuhan, China), diamine oxidase (DAO, Shanghai Yueyan Biological Technology, China), a TNF-α (#PT516, Beyotime, Shanghai, China) were measured according to the instruction of kits.

### 2.8 2.7 Bininformatic analysis

DESeq2 was used to analyze the differential gene expression analysis between groups. The raw counts of sequencing were set as the starting point for differential expression analysis, and ‘DESeq Data Set From Matrix’ was ran. The produced R object was used for differential gene expression analysis. The ‘contrast’ function was used for differential gene expression analysis, and fold changes of each gene and related *P* values were obtained. GAGE analysis was used to obtain standard deviation and mean. Then, *t* test statistic and *P* value between the background and gene set were obtained. Meanwhile the downregulated or upregulated pathway identified by GAGE was also obtained. Mestrenova 6.1 (Mestrelab Research S.L, Spain) software was used to perform partial least squares discriminant analysis (PLS-DA) and orthogonal partial least squares discriminant analysis (OPLS-DA).

### 2.9 2.8 Immunofluorescence staining

The tissues were fixed with 4% paraformaldehyde (Beyotime, China) and embedded in paraffin. The tissues were cut into 6 mm sections. After antigen repair, dewaxing, hydrogen peroxide treatment, the tissues were treated with bovine serum albumin for 30 min. After incubation with primary antibody (Abcam, UK) overnight, the sections were washed 3 times with PBS. Then, the sections were incubated with a secondary antibody for 2 h. After incubation with DAB (3,3ʹg-diaminobenzidine) chromogenic solution (Abcam, UK), the slides were analyzed using a fluorescence microscope.

### 2.10 2.9 Statistical analysis

Data are presented as mean ± standard error. SPSS software (SPSS Co., Ltd., USA) was used to analyze data. Student’s *t*-test was used for statistical analysis between two groups, and one-way ANOVA was used to analyze data among three groups. *P* values less than 0.05 were believed to be statistically different.

## 3. Results

### 3.1 Curcumin remarkably improved intestinal integrity through increasing the tight junction proteins expression

**Figure 1.**
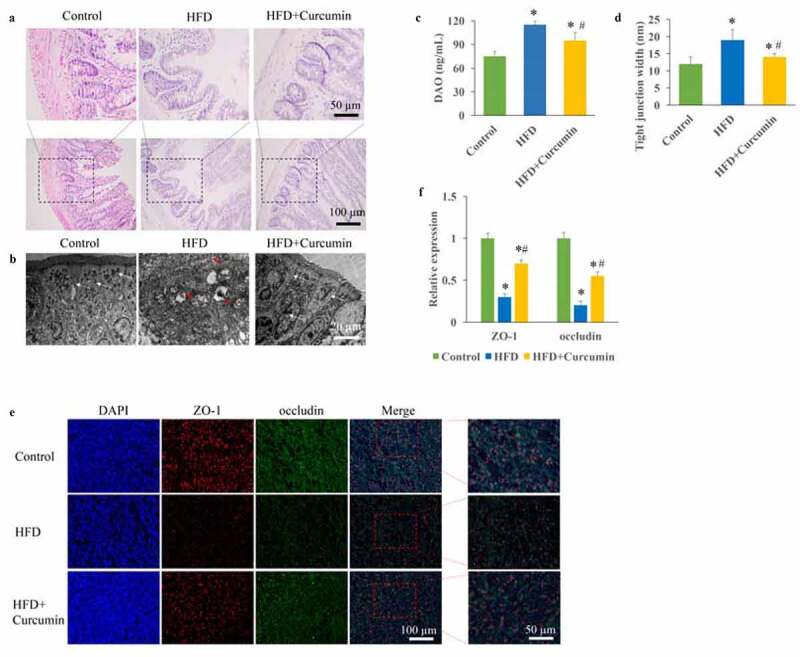
Improvement of intestinal integrity by curcumin. (a) Intact tight junctions in the ileal tissues were investigated through HE staining; (b) Intact tight junctions in the ileal tissues were investigated through TEM; (c) The contents of DAO in the serum were measured; (d) Tight junction width in the ileal tissue were investigated; (e) The protein expression of occludin and ZO-1 were measured through immunofluorescence staining; (f) The protein expression of occludin and ZO-1 were analyzed. *P< 0.05 compared to control group. #*P* < 0.05 compared to the HFD group. White arrows indicate tight junctions and red arrows indicate tight junctions’ disruption. Ten rats were used in each group. Three independent experiments were performed to detect DAO, tight junction width, and expression of tight junction proteins

The T2DM animal was established through STZ injection and HFD diet. Intact tight junctions in the ileal tissues of control group animals were remarkably widened after HFD treatment. However, curcumin administration markedly reversed the adverse influence of HFD on tissue damage (Figure 1(a-b)). The contents of DAO in the serum were remarkably evaluated in the HFD group compared with the control group (Figure 1(c)), but curcumin treatment significantly suppressed it. Meanwhile, remarkable wider intact tight junctions in the ileal tissue were observed in the HFD group, and reversed by curcumin (Figure 1(d)). The levels of tight junction molecules including occludin and ZO-1 are closely linked with intestinal permeability. We found that the protein expression of occludin and ZO-1 were remarkably inhibited in the HFD group (Figure 1(e-f)), but treatment with curcumin significantly restored their expression.

### 3.2 Curcumin improved hyperglycemia and insulin resistance in diabetic mice

**Figure 2.**
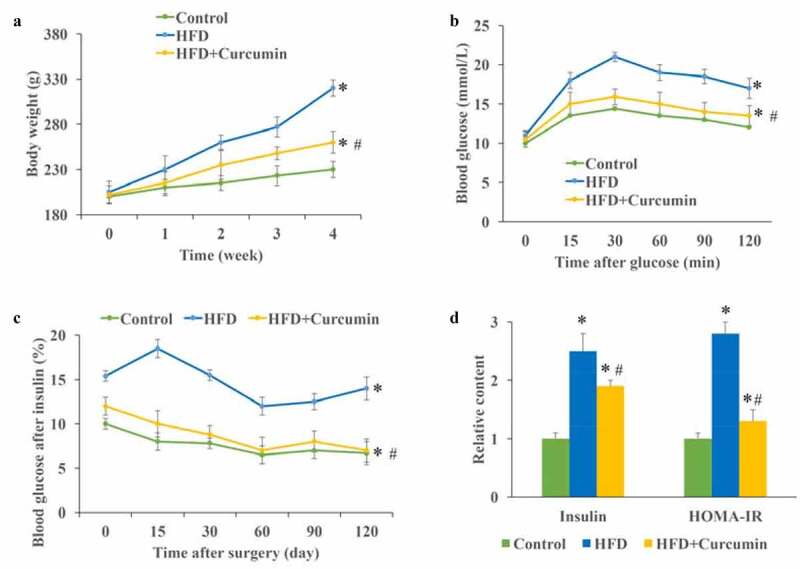
Curcumin improved hyperglycemia and insulin resistance in diabetic mice. (a) Curcumin significantly slowed down the increase of body weight caused by HFD; (b) The glucose tolerance test was measured to investigate the influence of curcumin on hyperglycemia-lowering effect; (c) The insulin tolerance test was measured to investigate the influence of curcumin on hyperglycemia-lowering effect; (d) The levels of fasting insulin and HOMA-IR were measured. *P < 0.05 compared to control group. #*P* < 0.05 compared to the HFD group. Ten rats were used in each group. Three independent experiments were performed in these experiments

The influence of curcumin on body weight, blood glucose, insulin, and HOMA-IR were investigated. The body weight of animals in the HFD group increased greatly compared with that of the control and HFD+curcumin groups, and curcumin significantly slowed down the increase in body weight (Figure 2(a)). The insulin tolerance test and glucose tolerance test were performed to investigate the influence of curcumin on hyperglycemia-lowering effect in vivo. We found that the glucose and insulin tolerance of HFD animals were remarkably improved by curcumin treatment (Figure 2(b-c)). The levels of fasting insulin and homeostatic model assessment for insulin resistance (HOMA-IR) were significantly promoted in the HFD group, and inhibited by curcumin treatment (Figure 2(d)). These data indicated that curcumin could suppressed the insulin resistance caused by HFD.

### 3.3 *Curcumin remarkably suppressed metabolic endotoxemia by decreasing LPS, TNF-*α, *and TLR4/NF-κB signaling pathway*

**Figure 3.**
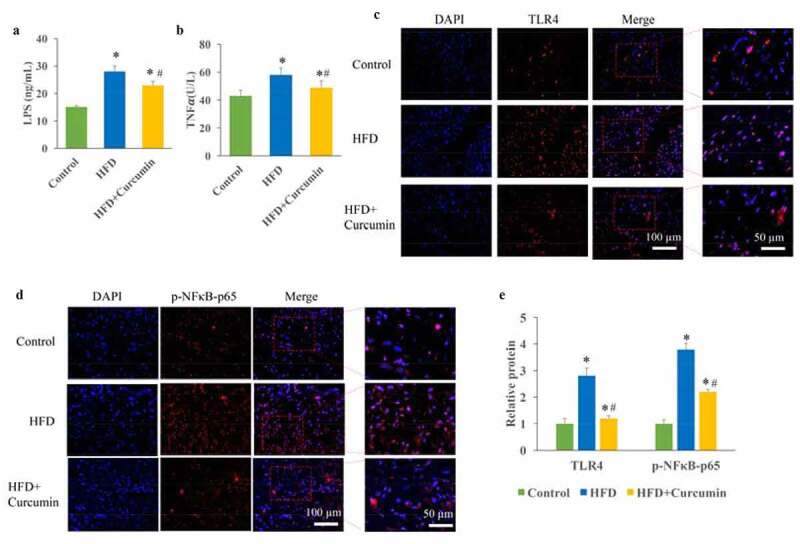
Inhibition of metabolic endotoxemia by curcumin. (a) The increased LPS caused by HFD was decreased by curcumin; (b) The increased TNF-***α*** caused by HFD was decreased by curcumin; (c) The TLR4 expression and NF-κB activation were measured through Western blotting; (d) The increase of TLR4 and NF-κB activation caused by HFD were reduced by curcumin administration. *P < 0.05 compared to control group. #*P* < 0.05 compared to the HFD group. Ten rats were used in each group. Three independent experiments were performed in these experiments

The influence of curcumin on TLR4/NF-κB was investigated. The increase of LPS in the serum could result in the metabolic endotoxemia, and further lead to insulin resistance and pro-inflammatory cytokines increase. In this study, we found that the levels of LPS and TNF-***α*** were markedly promoted in the HFD group. However, the increased LPS and TNF-***α*** were decreased after curcumin treatment (Figure 3(a-b)). In addition, remarkable increase of TLR4 up-regulation and NF-κB activation was found in the HFD group, but reduced by curcumin administration (Figure 3(c-e)).

### 3.4 Curcumin reversed gut microbiota dysbiosis in diabetic mice by increasing Bacteroidetes and *Bifidobacterium* spp., but supressing Enterobacterales and Firmicutes

**Figure 4.**
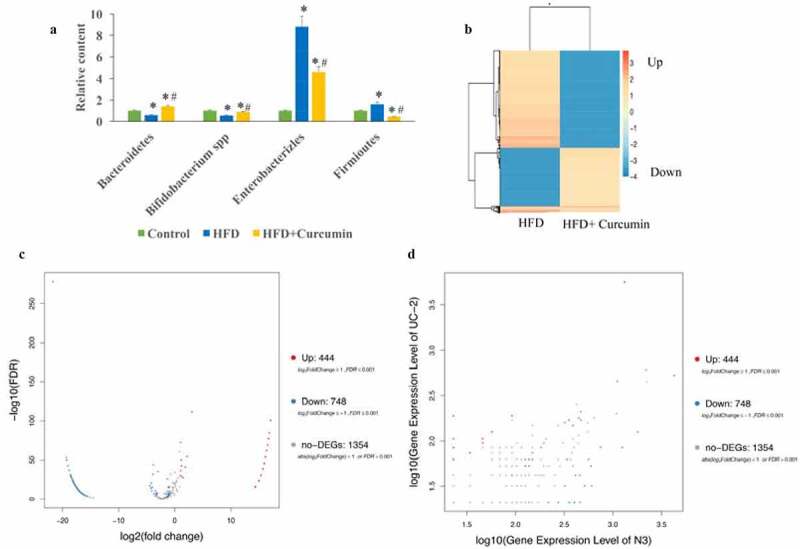
Curcumin reversed gut microbiota dysbiosis in diabetic mice. (a) The gut microbiota dysbiosis change caused by HFD was reversed by curcumin; (b) The differently expressed genes in the tissues between the HFD group and HFD+curcumin group were analyzed through Pheatmap method; (c) The differently expressed genes in the tissues between HFD group and HFD+curcumin group were analyzed through volcano plot method; (d) The differently expressed genes in the tissues between the HFD group and the HFD+curcumin group were analyzed through scatter plot method. *P < 0.05 compared to group control. #*P* < 0.05 compared to the HFD group. Ten rats were used in each group. Three independent experiments were performed in these experiments

In this study, the levels of four common bacteria were investigated. In the HFD group, the levels of Bacteroidetes and *Bifidobacterium* spp. were suppressed, the levels of Enterobacterales and Firmicutes were increased in the HFD group. However, the effects of HFD were reversed by curcumin administration (Figure 4(a)). The differently expressed genes in the tissues between HFD group and HFD+curcumin group were analyzed through Pheatmap (Figure 4(b)), volcano plot and scatter plot methods (Figure 4(c-d)). These data suggested that 444 upregulated genes and 748 downregulated genes between normal and UC groups were found.

### 3.5 Metabonomics, gene ontology and enriched KEGG biological pathway analysis

**Figure 5.**
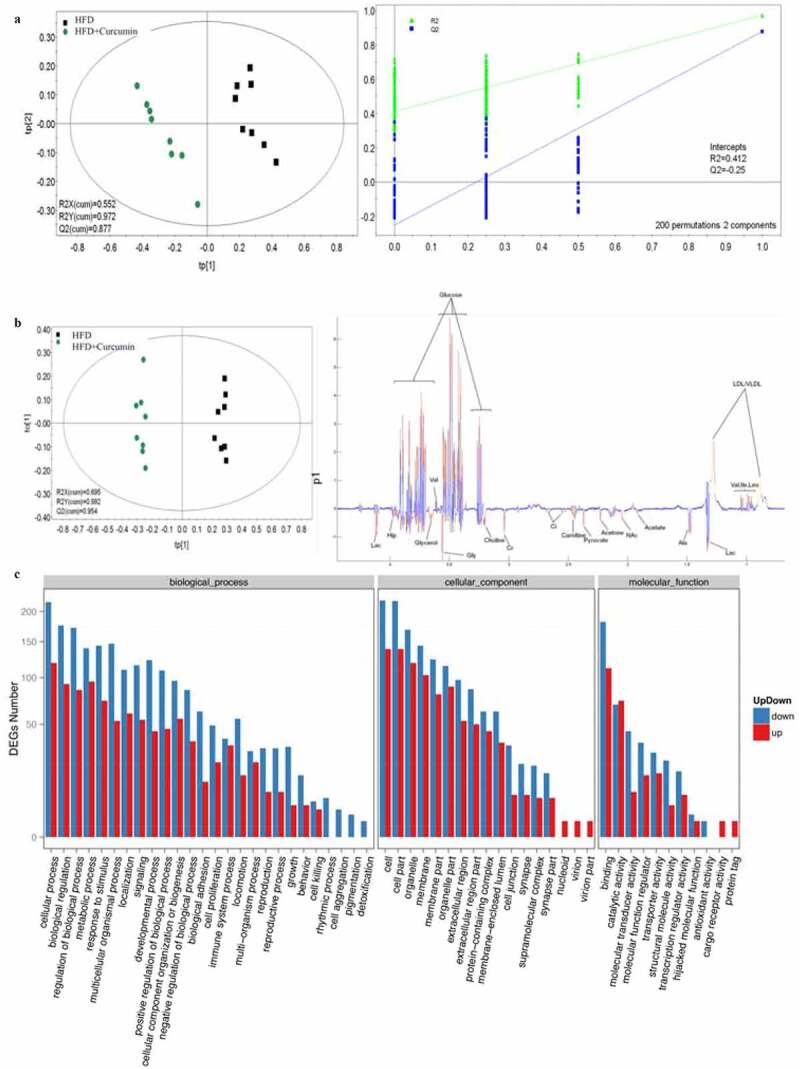
Influence of curcumin on the metabonomics of HFD animals. (a) PLS-DA was used to investigate the influence of curcumin on the metabonomics of HFD animals; (b) OPLS-DA was used to investigate the influence of curcumin on the metabonomics of HFD animals; (c) The DEGs number of the most enriched pathway was analyzed through gene ontology. Ten rats were used in each group. Three independent experiments were performed in these experiments

**Figure 6.**
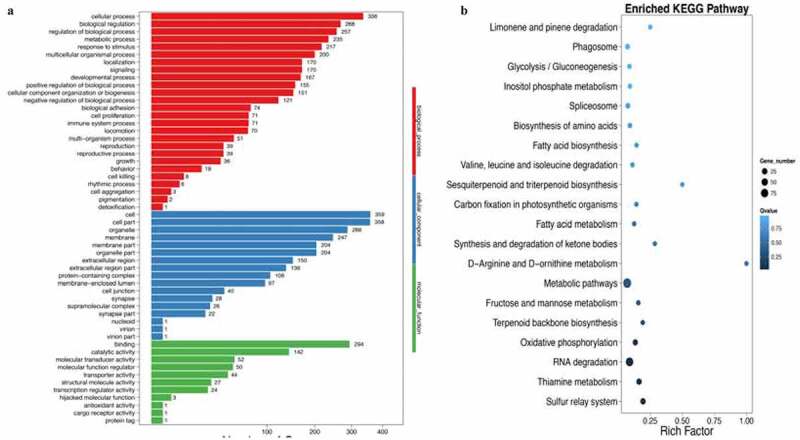
Gene ontology and enriched KEGG biological pathway analysis. (a) Gene ontology analysis was used to investigate biological, cellular component, and molecular function processes; (b) Enriched KEGG method was used to analyze differentially expressed genes. Ten rats were used in each group. Three independent experiments were performed in these experiments

Partial least squares discriminant analysis (PLS-DA) and orthogonal partial least squares discriminant analysis (OPLS-DA) methods were used to investigate the influence of curcumin on the metabonomics of HFD animals. The metabolic patterns in the serum of HFD group and HFD+curcumin group were distinguished effectively (Figure 5(a-b)). Meanwhile, the DEGs number of the most enriched pathway was analyzed through gene ontology (Figure 5(c)). Gene ontology analysis was used to investigate biological, cellular component, and molecular function processes (Figure 6(a)), and enriched KEGG method was used to analyze differentially expressed genes (Figure 6(b)). The number of differentially expressed genes were presented based on the size of dots.

## 4. Discussion

In this study, we demonstrated that curcumin could improve the intestinal integrity in the T2DM animal model and promoted the expression of ZO-1 and occludin (Figure 1). In addition, the hyperglycemia and insulin resistance of T2DM rats were relieved by curcumin (Figure 2). The suppressive effect of curcumin on TLR4/NF-κB was also observed (Figure 3). Curcumin also improved the gut microbiota dysbiosis in T2DM (Figures 4–6). However, some studies have indicated the protective role of curcumin in the intestinal barrier of diabetes. Their conclusions [9,15] that curcumin could improve the intestinal barrier function are in line with our study. However, these studies did not investigate the role of curcumin in gut microbiota and TLR4/NF-κB signaling pathway.

T2DM is a complex genetic disease characterized by genetic and environmental factors. In recent years, the incidence rate has increased year by year, which has seriously endangered human health [16]. Systematic chronic inflammation is now considered to play an important role in the pathogenesis of T2DM [17].

Systemic inflammation induced by LPS is an important factor in the pathogenesis of metabolic diseases including T2DM [18]. This pathological phenomenon of circulating lipopolysaccharide level 2.3 times higher than normal is defined as metabolic endotoxemia [19]. The increase of LPS in the internal circulation can not only cause liver inflammation and promote liver fibrosis but also lead to inflammation of muscle, fat and other tissues, and insulin resistance [20,21]. In this study, we found that the significant increase of LPS caused by HFD could be remarkably reduced by curcumin (Figure 3(a)). This might be a potential mechanism how curcumin improves the intestinal injury of type 2 diabetic rats. It was reported that LPS could further cause the activation of TLR4/NF-κB [22]. Our findings that curcumin suppressed the expression of TLR4/NF-κB could further confirm the inhibition role against metabolic endotoxemia.

Tight junction plays an important role in maintaining the normal function of intestinal mucosal barrier. It is not only the most important structural basis of intestinal epithelial mechanical barrier but also the rate limiting step in regulating the transport of paracellular substances [23]. Tight junctions are mainly composed of transmembrane proteins occludin and ZO-1. Their main functions are to maintain the polarity of intestinal epithelial cells, regulate the permeability of intestinal barrier, and reduce the entry of intestinal macromolecules and microorganisms into the internal environment through the intestinal wall. We found that the levels of occludin and ZO-1 could be markedly increased by curcumin in the type 2 diabetic rats (Figure 1(e-f)), which is in line with previous research [24]. Occludin and ZO-1 might be the functioning targets of curcumin in improving intestinal barrier injury caused by HFD.

It was reported that the intestinal imbalance could be observed in the T2DM patients. Opportunistic pathogens such as Bacteroides, *Escherichia coli*, and *Thiobacillus* spp. producing bacteria increased significantly in T2DM patients. In patients with T2DM, the cell membrane of intestinal flora is more active in the transportation of sugar and branched chain amino acids, but the synthesis of butyric acid is reduced [25]. We found that the imbalanced intestinal caused by HFD could be significantly reversed by curcumin (Figure 4(a)). To the best of our knowledge, we first reported the regulation function of curcumin in intestinal flora disorder in T2DM rats.

Adjusting intestinal flora or improving intestinal inflammation can alleviate intestinal inflammation and reduce the entry of intestinal LPS into the body by reducing the injury of intestinal tight junction protein in T2DM [26,27]. Improving intestinal mucosal barrier function, reducing intestinal permeability and reducing intestinal bacterial metabolites such as LPS into the blood circulation are currently considered as new therapeutic strategies for improving metabolic diseases such as T2DM [28].

There are several limitations in this research. Only four kinds of intestinal bacteria were measured, and more types of intestinal bacteria need to be screened. In addition, the specific functioning signaling pathway and target, through which curcumin improves intestinal barrier function, gut microbiota, and metabolic endotoxemia, remain unclear. In our further research, we will focus on the function signaling pathway and specific target of curcumin in protecting barrier function, and improving gut microbiota and metabolic endotoxemia.

## 5. Conclusion

In this study, we demonstrated that curcumin could improve the intestinal barrier function, gut microbiota, and metabolic endotoxemia in T2DM rats. Therefore, curcumin might be a potential therapeutic agent for the treatment of T2DM.

## Supplementary Material

Supplemental Material

## Data Availability

The data and material used to support the findings of this study are included within the manuscript and supplementary files.

## Abbreviations

Type 2 diabetes mellitus (T2DM); Lipopolysaccharide (LPS); Toll-like receptor 4 (TLR4); Myeloid differentiation molecule 88 (MyD88); Streptozotocin (STZ); High-fat diet (HFD); Transfer electron microscopy (TEM); Homeostasis model assessment of insulin resistance (HOMA-IR); Diamine oxidase (DAO); Partial least squares discriminant analysis (PLS-DA); Orthogonal partial least squares discriminant analysis (OPLS-DA); Nuclear factor-κB (NF-κB); Interleukin-1β (IL-1β); Tumor necrosis factor α (TNF-α); Monocyte chemoattractant protein-1 (MCP-1); Hematoxylin and Eosin (HE); Specificpathogen-free organism (SPF); 3,3ʹ-diaminobenzidine (DAB); Cycle threshold (CT).
