# Supplementary material for: Improvement of intestinal barrier function, gut microbiota, and metabolic endotoxemia in type 2 diabetes rats by curcumin
Source: Bioengineered. 2021 Dec 19;12(2):11947–58. doi: 10.1080/21655979.2021.2009322 (PMC8810160; doi:10.1080/21655979.2021.2009322)
Supplement: Supplemental Material [file KBIE_A_2009322_SM6765.zip › supplementary details/GOView.html]

GOView
 
