# Supplementary material for: Improvement of intestinal barrier function, gut microbiota, and metabolic endotoxemia in type 2 diabetes rats by curcumin
Source: Bioengineered. 2021 Dec 19;12(2):11947–58. doi: 10.1080/21655979.2021.2009322 (PMC8810160; doi:10.1080/21655979.2021.2009322)
Supplement: Supplemental Material [file KBIE_A_2009322_SM6765.zip › supplementary details/N-VS-UC.DEGseq_Method.htm]

N-VS-UC.DEGseq\_Method

1. N-VS-UC.DEGseq\_Method

| # | Pathway | N-VS-UC.DEGseq\_Method (3259) | All-gene (17954) | Pvalue | Qvalue | Pathway ID | Level 1 | Level 2 |
| 1 | Endocytosis | 131 | 407 | 3.812293e-12 | 1.284743e-09 | ko04144 | Cellular Processes | Transport and catabolism |
| 2 | Ubiquitin mediated proteolysis | 69 | 190 | 1.963323e-09 | 3.308199e-07 | ko04120 | Genetic Information Processing | Folding, sorting and degradation |
| 3 | AMPK signaling pathway | 53 | 152 | 6.254972e-07 | 7.026419e-05 | ko04152 | Environmental Information Processing | Signal transduction |
| 4 | Phosphatidylinositol signaling system | 48 | 140 | 3.561622e-06 | 2.985819e-04 | ko04070 | Environmental Information Processing | Signal transduction |
| 5 | Inositol phosphate metabolism | 36 | 95 | 4.429998e-06 | 2.985819e-04 | ko00562 | Metabolism | Carbohydrate metabolism |
| 6 | Insulin resistance | 42 | 123 | 1.57926e-05 | 8.870177e-04 | ko04931 | Human Diseases | Endocrine and metabolic diseases |
| 7 | Protein processing in endoplasmic reticulum | 65 | 219 | 1.983344e-05 | 9.548385e-04 | ko04141 | Genetic Information Processing | Folding, sorting and degradation |
| 8 | HIF-1 signaling pathway | 38 | 114 | 7.116422e-05 | 2.997793e-03 | ko04066 | Environmental Information Processing | Signal transduction |
| 9 | Neurotrophin signaling pathway | 51 | 171 | 0.0001291421 | 4.835654e-03 | ko04722 | Organismal Systems | Nervous system |
| 10 | Regulation of actin cytoskeleton | 85 | 323 | 0.0001557444 | 5.056866e-03 | ko04810 | Cellular Processes | Cell motility |
| 11 | Thyroid hormone signaling pathway | 49 | 164 | 0.0001650609 | 5.056866e-03 | ko04919 | Organismal Systems | Endocrine system |
| 12 | Hepatitis B | 55 | 191 | 0.0001989391 | 5.586873e-03 | ko05161 | Human Diseases | Infectious diseases: Viral |
| 13 | Fc gamma R-mediated phagocytosis | 42 | 137 | 0.0002545523 | 6.598779e-03 | ko04666 | Organismal Systems | Immune system |
| 14 | B cell receptor signaling pathway | 28 | 82 | 0.000388927 | 8.963452e-03 | ko04662 | Organismal Systems | Immune system |
| 15 | Osteoclast differentiation | 46 | 157 | 0.0004172943 | 8.963452e-03 | ko04380 | Organismal Systems | Development |
| 16 | Prostate cancer | 37 | 119 | 0.0004255645 | 8.963452e-03 | ko05215 | Human Diseases | Cancers: Specific types |
| 17 | EGFR tyrosine kinase inhibitor resistance | 33 | 103 | 0.0004686328 | 9.289956e-03 | ko01521 | Human Diseases | Drug resistance: Antineoplastic |
| 18 | Rap1 signaling pathway | 75 | 288 | 0.0005125772 | 9.596584e-03 | ko04015 | Environmental Information Processing | Signal transduction |
| 19 | Central carbon metabolism in cancer | 24 | 68 | 0.0005734734 | 1.017161e-02 | ko05230 | Human Diseases | Cancers: Overview |
| 20 | Cellular senescence | 57 | 208 | 0.0006144633 | 1.035371e-02 | ko04218 | Cellular Processes | Cell growth and death |
| 21 | Pancreatic cancer | 28 | 85 | 0.0007482641 | 1.100515e-02 | ko05212 | Human Diseases | Cancers: Specific types |
| 22 | Th17 cell differentiation | 36 | 118 | 0.0007485764 | 1.100515e-02 | ko04659 | Organismal Systems | Immune system |
| 23 | Non-small cell lung cancer | 27 | 81 | 0.0007510932 | 1.100515e-02 | ko05223 | Human Diseases | Cancers: Specific types |
| 24 | Insulin signaling pathway | 53 | 194 | 0.001008183 | 1.415657e-02 | ko04910 | Organismal Systems | Endocrine system |
| 25 | Leukocyte transendothelial migration | 45 | 160 | 0.001231125 | 1.659556e-02 | ko04670 | Organismal Systems | Immune system |
| 26 | Longevity regulating pathway | 35 | 117 | 0.001293758 | 1.676909e-02 | ko04211 | Organismal Systems | Aging |
| 27 | ErbB signaling pathway | 33 | 109 | 0.001403226 | 1.751434e-02 | ko04012 | Environmental Information Processing | Signal transduction |
| 28 | Adherens junction | 38 | 132 | 0.001797472 | 2.163386e-02 | ko04520 | Cellular Processes | Cellular community - eukaryotes |
| 29 | Jak-STAT signaling pathway | 49 | 182 | 0.002126057 | 2.443536e-02 | ko04630 | Environmental Information Processing | Signal transduction |
| 30 | Apoptosis | 56 | 214 | 0.002175255 | 2.443536e-02 | ko04210 | Cellular Processes | Cell growth and death |
| 31 | MAPK signaling pathway - fly | 36 | 125 | 0.002315963 | 2.463283e-02 | ko04013 | Environmental Information Processing | Signal transduction |
| 32 | Sphingolipid signaling pathway | 41 | 147 | 0.002339022 | 2.463283e-02 | ko04071 | Environmental Information Processing | Signal transduction |
| 33 | Pathways in cancer | 204 | 940 | 0.002528827 | 2.582469e-02 | ko05200 | Human Diseases | Cancers: Overview |
| 34 | C-type lectin receptor signaling pathway | 37 | 131 | 0.002938063 | 2.868724e-02 | ko04625 | Organismal Systems | Immune system |
| 35 | Th1 and Th2 cell differentiation | 34 | 118 | 0.002986889 | 2.868724e-02 | ko04658 | Organismal Systems | Immune system |
| 36 | Renal cell carcinoma | 26 | 84 | 0.003064512 | 2.868724e-02 | ko05211 | Human Diseases | Cancers: Specific types |
| 37 | Endometrial cancer | 24 | 76 | 0.00325168 | 2.961665e-02 | ko05213 | Human Diseases | Cancers: Specific types |
| 38 | Human T-cell leukemia virus 1 infection | 64 | 256 | 0.003623879 | 3.154914e-02 | ko05166 | Human Diseases | Infectious diseases: Viral |
| 39 | Colorectal cancer | 30 | 102 | 0.003651087 | 3.154914e-02 | ko05210 | Human Diseases | Cancers: Specific types |
| 40 | Fc epsilon RI signaling pathway | 25 | 82 | 0.004541876 | 3.733201e-02 | ko04664 | Organismal Systems | Immune system |
| 41 | Platinum drug resistance | 25 | 82 | 0.004541876 | 3.733201e-02 | ko01524 | Human Diseases | Drug resistance: Antineoplastic |
| 42 | MicroRNAs in cancer | 51 | 198 | 0.004683889 | 3.758263e-02 | ko05206 | Human Diseases | Cancers: Overview |
| 43 | TNF signaling pathway | 35 | 126 | 0.005009634 | 3.926155e-02 | ko04668 | Environmental Information Processing | Signal transduction |
| 44 | Cholinergic synapse | 37 | 136 | 0.005777571 | 4.425094e-02 | ko04725 | Organismal Systems | Nervous system |
| 45 | Progesterone-mediated oocyte maturation | 34 | 123 | 0.006092179 | 4.562365e-02 | ko04914 | Organismal Systems | Endocrine system |
| 46 | Phospholipase D signaling pathway | 48 | 187 | 0.006365287 | 4.663265e-02 | ko04072 | Environmental Information Processing | Signal transduction |
| 47 | Autophagy - animal | 89 | 383 | 0.006613784 | 4.742224e-02 | ko04140 | Cellular Processes | Transport and catabolism |
| 48 | Endocrine resistance | 39 | 147 | 0.007369028 | 5.173672e-02 | ko01522 | Human Diseases | Drug resistance: Antineoplastic |
| 49 | FoxO signaling pathway | 40 | 152 | 0.007763428 | 5.339337e-02 | ko04068 | Environmental Information Processing | Signal transduction |
| 50 | Acute myeloid leukemia | 23 | 78 | 0.009766328 | 6.582505e-02 | ko05221 | Human Diseases | Cancers: Specific types |
| 51 | DNA replication | 16 | 49 | 0.01049398 | 6.832465e-02 | ko03030 | Genetic Information Processing | Replication and repair |
| 52 | Bacterial invasion of epithelial cells | 37 | 141 | 0.01063907 | 6.832465e-02 | ko05100 | Human Diseases | Infectious diseases: Bacterial |
| 53 | Tight junction | 71 | 302 | 0.01074542 | 6.832465e-02 | ko04530 | Cellular Processes | Cellular community - eukaryotes |
| 54 | Proteoglycans in cancer | 101 | 450 | 0.01127206 | 7.025494e-02 | ko05205 | Human Diseases | Cancers: Overview |
| 55 | Adipocytokine signaling pathway | 23 | 79 | 0.01146594 | 7.025494e-02 | ko04920 | Organismal Systems | Endocrine system |
| 56 | Lysosome | 48 | 193 | 0.01170392 | 7.043252e-02 | ko04142 | Cellular Processes | Transport and catabolism |
| 57 | Hedgehog signaling pathway | 17 | 54 | 0.0125371 | 7.378005e-02 | ko04340 | Environmental Information Processing | Signal transduction |
| 58 | Apoptosis - fly | 21 | 71 | 0.01269805 | 7.378005e-02 | ko04214 | Cellular Processes | Cell growth and death |
| 59 | Lysine degradation | 34 | 129 | 0.0130008 | 7.425881e-02 | ko00310 | Metabolism | Amino acid metabolism |
| 60 | Choline metabolism in cancer | 38 | 148 | 0.01392101 | 7.818967e-02 | ko05231 | Human Diseases | Cancers: Overview |
| 61 | N-Glycan biosynthesis | 19 | 64 | 0.01654894 | 9.142611e-02 | ko00510 | Metabolism | Glycan biosynthesis and metabolism |
| 62 | Chronic myeloid leukemia | 27 | 100 | 0.0183178 | 9.823320e-02 | ko05220 | Human Diseases | Cancers: Specific types |
| 63 | cGMP-PKG signaling pathway | 52 | 217 | 0.01836407 | 9.823320e-02 | ko04022 | Environmental Information Processing | Signal transduction |
| 64 | Homologous recombination | 16 | 52 | 0.01894544 | 9.975958e-02 | ko03440 | Genetic Information Processing | Replication and repair |
| 65 | Human immunodeficiency virus 1 infection | 65 | 281 | 0.01986864 | 1.027387e-01 | ko05170 | Human Diseases | Infectious diseases: Viral |
| 66 | T cell receptor signaling pathway | 31 | 119 | 0.02012094 | 1.027387e-01 | ko04660 | Organismal Systems | Immune system |
| 67 | Ras signaling pathway | 71 | 311 | 0.02069082 | 1.040717e-01 | ko04014 | Environmental Information Processing | Signal transduction |
| 68 | Dorso-ventral axis formation | 24 | 88 | 0.02231245 | 1.105779e-01 | ko04320 | Organismal Systems | Development |
| 69 | Glioma | 29 | 112 | 0.02591502 | 1.265705e-01 | ko05214 | Human Diseases | Cancers: Specific types |
| 70 | Circadian rhythm | 11 | 33 | 0.02676086 | 1.279733e-01 | ko04710 | Organismal Systems | Environmental adaptation |
| 71 | Type II diabetes mellitus | 16 | 54 | 0.02696173 | 1.279733e-01 | ko04930 | Human Diseases | Endocrine and metabolic diseases |
| 72 | Kaposi sarcoma-associated herpesvirus infection | 53 | 227 | 0.02786759 | 1.304358e-01 | ko05167 | Human Diseases | Infectious diseases: Viral |
| 73 | Chemokine signaling pathway | 58 | 253 | 0.03126588 | 1.443370e-01 | ko04062 | Organismal Systems | Immune system |
| 74 | Epstein-Barr virus infection | 54 | 234 | 0.03268364 | 1.462200e-01 | ko05169 | Human Diseases | Infectious diseases: Viral |
| 75 | Valine, leucine and isoleucine degradation | 18 | 64 | 0.03289021 | 1.462200e-01 | ko00280 | Metabolism | Amino acid metabolism |
| 76 | Hedgehog signaling pathway - fly | 11 | 34 | 0.03325521 | 1.462200e-01 | ko04341 | Environmental Information Processing | Signal transduction |
| 77 | Peroxisome | 27 | 105 | 0.03340931 | 1.462200e-01 | ko04146 | Cellular Processes | Transport and catabolism |
| 78 | Viral carcinogenesis | 56 | 246 | 0.03817969 | 1.649558e-01 | ko05203 | Human Diseases | Cancers: Overview |
| 79 | Hippo signaling pathway - fly | 29 | 116 | 0.03992159 | 1.702984e-01 | ko04391 | Environmental Information Processing | Signal transduction |
| 80 | Notch signaling pathway | 20 | 75 | 0.04334651 | 1.818013e-01 | ko04330 | Environmental Information Processing | Signal transduction |
| 81 | Nucleotide excision repair | 18 | 66 | 0.04369706 | 1.818013e-01 | ko03420 | Genetic Information Processing | Replication and repair |
| 82 | VEGF signaling pathway | 21 | 80 | 0.04571006 | 1.862668e-01 | ko04370 | Environmental Information Processing | Signal transduction |
| 83 | Prolactin signaling pathway | 24 | 94 | 0.04637159 | 1.862668e-01 | ko04917 | Organismal Systems | Endocrine system |
| 84 | Shigellosis | 31 | 127 | 0.04642853 | 1.862668e-01 | ko05131 | Human Diseases | Infectious diseases: Bacterial |
| 85 | Longevity regulating pathway - worm | 25 | 99 | 0.04807451 | 1.906013e-01 | ko04212 | Organismal Systems | Aging |
| 86 | RNA degradation | 70 | 320 | 0.04985842 | 1.953754e-01 | ko03018 | Genetic Information Processing | Folding, sorting and degradation |
| 87 | Apelin signaling pathway | 41 | 177 | 0.05363149 | 2.077450e-01 | ko04371 | Environmental Information Processing | Signal transduction |
| 88 | Glycosaminoglycan biosynthesis - keratan sulfate | 6 | 16 | 0.05457717 | 2.090058e-01 | ko00533 | Metabolism | Glycan biosynthesis and metabolism |
| 89 | Toll and Imd signaling pathway | 16 | 59 | 0.0576322 | 2.166867e-01 | ko04624 | Organismal Systems | Immune system |
| 90 | Toxoplasmosis | 41 | 178 | 0.05786884 | 2.166867e-01 | ko05145 | Human Diseases | Infectious diseases: Parasitic |
| 91 | Transcriptional misregulation in cancer | 75 | 350 | 0.06444178 | 2.386470e-01 | ko05202 | Human Diseases | Cancers: Overview |
| 92 | ABC transporters | 14 | 51 | 0.06650721 | 2.436188e-01 | ko02010 | Environmental Information Processing | Membrane transport |
| 93 | Long-term potentiation | 23 | 93 | 0.06874533 | 2.491094e-01 | ko04720 | Organismal Systems | Nervous system |
| 94 | SNARE interactions in vesicular transport | 11 | 38 | 0.07019217 | 2.498035e-01 | ko04130 | Genetic Information Processing | Folding, sorting and degradation |
| 95 | Focal adhesion | 100 | 480 | 0.07057073 | 2.498035e-01 | ko04510 | Cellular Processes | Cellular community - eukaryotes |
| 96 | Fatty acid biosynthesis | 6 | 17 | 0.0718188 | 2.498035e-01 | ko00061 | Metabolism | Lipid metabolism |
| 97 | Melanoma | 21 | 84 | 0.07207118 | 2.498035e-01 | ko05218 | Human Diseases | Cancers: Specific types |
| 98 | Axon guidance | 55 | 251 | 0.07264316 | 2.498035e-01 | ko04360 | Organismal Systems | Development |
| 99 | Caffeine metabolism | 3 | 6 | 0.07746798 | 2.621423e-01 | ko00232 | Metabolism | Biosynthesis of other secondary metabolites |
| 100 | Longevity regulating pathway - multiple species | 20 | 80 | 0.07778703 | 2.621423e-01 | ko04213 | Organismal Systems | Aging |
| 101 | Glucagon signaling pathway | 30 | 129 | 0.0844754 | 2.808500e-01 | ko04922 | Organismal Systems | Endocrine system |
| 102 | GnRH signaling pathway | 31 | 134 | 0.08520254 | 2.808500e-01 | ko04912 | Organismal Systems | Endocrine system |
| 103 | Natural killer cell mediated cytotoxicity | 32 | 139 | 0.08583842 | 2.808500e-01 | ko04650 | Organismal Systems | Immune system |
| 104 | Cutin, suberine and wax biosynthesis | 2 | 3 | 0.08687023 | 2.814756e-01 | ko00073 | Metabolism | Lipid metabolism |
| 105 | Chagas disease (American trypanosomiasis) | 26 | 110 | 0.08791727 | 2.814756e-01 | ko05142 | Human Diseases | Infectious diseases: Parasitic |
| 106 | Small cell lung cancer | 42 | 189 | 0.08853534 | 2.814756e-01 | ko05222 | Human Diseases | Cancers: Specific types |
| 107 | Circadian entrainment | 30 | 130 | 0.09151079 | 2.863306e-01 | ko04713 | Organismal Systems | Environmental adaptation |
| 108 | Regulation of lipolysis in adipocytes | 19 | 77 | 0.09337237 | 2.863306e-01 | ko04923 | Organismal Systems | Endocrine system |
| 109 | MAPK signaling pathway | 78 | 373 | 0.09344059 | 2.863306e-01 | ko04010 | Environmental Information Processing | Signal transduction |
| 110 | Human cytomegalovirus infection | 61 | 286 | 0.09393284 | 2.863306e-01 | ko05163 | Human Diseases | Infectious diseases: Viral |
| 111 | Bladder cancer | 13 | 49 | 0.09431068 | 2.863306e-01 | ko05219 | Human Diseases | Cancers: Specific types |
| 112 | Fatty acid degradation | 14 | 54 | 0.09898468 | 2.978378e-01 | ko00071 | Metabolism | Lipid metabolism |
| 113 | Calcium signaling pathway | 53 | 248 | 0.1087264 | 3.242548e-01 | ko04020 | Environmental Information Processing | Signal transduction |
| 114 | Hepatitis C | 39 | 178 | 0.1147442 | 3.392000e-01 | ko05160 | Human Diseases | Infectious diseases: Viral |
| 115 | Ubiquinone and other terpenoid-quinone biosynthesis | 5 | 15 | 0.1199214 | 3.479113e-01 | ko00130 | Metabolism | Metabolism of cofactors and vitamins |
| 116 | p53 signaling pathway | 24 | 104 | 0.1207882 | 3.479113e-01 | ko04115 | Cellular Processes | Cell growth and death |
| 117 | TGF-beta signaling pathway | 24 | 104 | 0.1207882 | 3.479113e-01 | ko04350 | Environmental Information Processing | Signal transduction |
| 118 | mTOR signaling pathway | 80 | 390 | 0.1245492 | 3.557041e-01 | ko04150 | Environmental Information Processing | Signal transduction |
| 119 | Propanoate metabolism | 11 | 42 | 0.1263718 | 3.578764e-01 | ko00640 | Metabolism | Carbohydrate metabolism |
| 120 | Fatty acid metabolism | 16 | 66 | 0.1313709 | 3.668952e-01 | ko01212 | Metabolism | Global and overview maps |
| 121 | Aldosterone-regulated sodium reabsorption | 12 | 47 | 0.1317339 | 3.668952e-01 | ko04960 | Organismal Systems | Excretory system |
| 122 | Influenza A | 52 | 247 | 0.1346522 | 3.719491e-01 | ko05164 | Human Diseases | Infectious diseases: Viral |
| 123 | Fluid shear stress and atherosclerosis | 39 | 181 | 0.1376197 | 3.770556e-01 | ko05418 | Human Diseases | Cardiovascular diseases |
| 124 | Mismatch repair | 8 | 29 | 0.1411834 | 3.830552e-01 | ko03430 | Genetic Information Processing | Replication and repair |
| 125 | Morphine addiction | 27 | 121 | 0.1420828 | 3.830552e-01 | ko05032 | Human Diseases | Substance dependence |
| 126 | Oocyte meiosis | 36 | 167 | 0.1480306 | 3.959231e-01 | ko04114 | Cellular Processes | Cell growth and death |
| 127 | Cell cycle | 32 | 147 | 0.1505166 | 3.994023e-01 | ko04110 | Cellular Processes | Cell growth and death |
| 128 | NF-kappa B signaling pathway | 27 | 122 | 0.1524338 | 4.013296e-01 | ko04064 | Environmental Information Processing | Signal transduction |
| 129 | NOD-like receptor signaling pathway | 46 | 220 | 0.1633641 | 4.247665e-01 | ko04621 | Organismal Systems | Immune system |
| 130 | Phosphonate and phosphinate metabolism | 3 | 8 | 0.1638565 | 4.247665e-01 | ko00440 | Metabolism | Metabolism of other amino acids |
| 131 | Retrograde endocannabinoid signaling | 37 | 174 | 0.1652214 | 4.250352e-01 | ko04723 | Organismal Systems | Nervous system |
| 132 | Dopaminergic synapse | 36 | 170 | 0.175842 | 4.476923e-01 | ko04728 | Organismal Systems | Nervous system |
| 133 | Fructose and mannose metabolism | 10 | 40 | 0.1766857 | 4.476923e-01 | ko00051 | Metabolism | Carbohydrate metabolism |
| 134 | cAMP signaling pathway | 56 | 274 | 0.1805318 | 4.540240e-01 | ko04024 | Environmental Information Processing | Signal transduction |
| 135 | Pyruvate metabolism | 13 | 55 | 0.1864882 | 4.619316e-01 | ko00620 | Metabolism | Carbohydrate metabolism |
| 136 | Ferroptosis | 13 | 55 | 0.1864882 | 4.619316e-01 | ko04216 | Cellular Processes | Cell growth and death |
| 137 | Gap junction | 25 | 115 | 0.1877882 | 4.619316e-01 | ko04540 | Cellular Processes | Cellular community - eukaryotes |
| 138 | Vasopressin-regulated water reabsorption | 12 | 51 | 0.2034636 | 4.968640e-01 | ko04962 | Organismal Systems | Excretory system |
| 139 | Fanconi anemia pathway | 15 | 66 | 0.2065705 | 5.008220e-01 | ko03460 | Genetic Information Processing | Replication and repair |
| 140 | Glycerophospholipid metabolism | 26 | 122 | 0.2118382 | 5.099248e-01 | ko00564 | Metabolism | Lipid metabolism |
| 141 | Vitamin B6 metabolism | 3 | 9 | 0.2142072 | 5.119704e-01 | ko00750 | Metabolism | Metabolism of cofactors and vitamins |
| 142 | Apoptosis - multiple species | 9 | 37 | 0.2173856 | 5.159081e-01 | ko04215 | Cellular Processes | Cell growth and death |
| 143 | Thermogenesis | 57 | 284 | 0.219322 | 5.168637e-01 | ko04714 | Organismal Systems | Environmental adaptation |
| 144 | Parathyroid hormone synthesis, secretion and action | 33 | 159 | 0.2232252 | 5.224090e-01 | ko04928 | Organismal Systems | Endocrine system |
| 145 | Adrenergic signaling in cardiomyocytes | 39 | 191 | 0.2320372 | 5.377357e-01 | ko04261 | Organismal Systems | Circulatory system |
| 146 | Necroptosis | 43 | 212 | 0.2329656 | 5.377357e-01 | ko04217 | Cellular Processes | Cell growth and death |
| 147 | Measles | 31 | 150 | 0.2396429 | 5.493854e-01 | ko05162 | Human Diseases | Infectious diseases: Viral |
| 148 | Amino sugar and nucleotide sugar metabolism | 14 | 63 | 0.2436503 | 5.513392e-01 | ko00520 | Metabolism | Carbohydrate metabolism |
| 149 | Hippo signaling pathway - multiple species | 10 | 43 | 0.2437672 | 5.513392e-01 | ko04392 | Environmental Information Processing | Signal transduction |
| 150 | Pancreatic secretion | 25 | 120 | 0.2545645 | 5.719216e-01 | ko04972 | Organismal Systems | Digestive system |
| 151 | Tuberculosis | 50 | 252 | 0.264746 | 5.881095e-01 | ko05152 | Human Diseases | Infectious diseases: Bacterial |
| 152 | Mitophagy - animal | 56 | 284 | 0.2667711 | 5.881095e-01 | ko04137 | Cellular Processes | Transport and catabolism |
| 153 | Mannose type O-glycan biosynthesis | 8 | 34 | 0.2670052 | 5.881095e-01 | ko00515 | Metabolism | Glycan biosynthesis and metabolism |
| 154 | Inflammatory mediator regulation of TRP channels | 27 | 132 | 0.2771821 | 6.015599e-01 | ko04750 | Organismal Systems | Sensory system |
| 155 | Epithelial cell signaling in Helicobacter pylori infection | 16 | 75 | 0.2784669 | 6.015599e-01 | ko05120 | Human Diseases | Infectious diseases: Bacterial |
| 156 | Glycerolipid metabolism | 16 | 75 | 0.2784669 | 6.015599e-01 | ko00561 | Metabolism | Lipid metabolism |
| 157 | Vibrio cholerae infection | 15 | 70 | 0.2811079 | 6.033972e-01 | ko05110 | Human Diseases | Infectious diseases: Bacterial |
| 158 | Hepatocellular carcinoma | 80 | 414 | 0.284285 | 6.063547e-01 | ko05225 | Human Diseases | Cancers: Specific types |
| 159 | Oxytocin signaling pathway | 41 | 207 | 0.2932322 | 6.183780e-01 | ko04921 | Organismal Systems | Endocrine system |
| 160 | Signaling pathways regulating pluripotency of stem cells | 71 | 367 | 0.2942052 | 6.183780e-01 | ko04550 | Cellular Processes | Cellular community - eukaryotes |
| 161 | beta-Alanine metabolism | 8 | 35 | 0.2954269 | 6.183780e-01 | ko00410 | Metabolism | Metabolism of other amino acids |
| 162 | Insulin secretion | 24 | 118 | 0.3025041 | 6.292832e-01 | ko04911 | Organismal Systems | Endocrine system |
| 163 | Phototransduction - fly | 13 | 61 | 0.3079282 | 6.331866e-01 | ko04745 | Organismal Systems | Sensory system |
| 164 | Gastric cancer | 96 | 503 | 0.3081383 | 6.331866e-01 | ko05226 | Human Diseases | Cancers: Specific types |
| 165 | Salmonella infection | 29 | 145 | 0.3124596 | 6.381751e-01 | ko05132 | Human Diseases | Infectious diseases: Bacterial |
| 166 | Base excision repair | 11 | 51 | 0.3148402 | 6.391635e-01 | ko03410 | Genetic Information Processing | Replication and repair |
| 167 | GABAergic synapse | 24 | 119 | 0.3181759 | 6.396631e-01 | ko04727 | Organismal Systems | Nervous system |
| 168 | PI3K-Akt signaling pathway | 115 | 607 | 0.3188825 | 6.396631e-01 | ko04151 | Environmental Information Processing | Signal transduction |
| 169 | Protein export | 6 | 26 | 0.3291269 | 6.543703e-01 | ko03060 | Genetic Information Processing | Folding, sorting and degradation |
| 170 | Monobactam biosynthesis | 1 | 2 | 0.3300978 | 6.543703e-01 | ko00261 | Metabolism | Biosynthesis of other secondary metabolites |
| 171 | Gastric acid secretion | 22 | 110 | 0.34371 | 6.742110e-01 | ko04971 | Organismal Systems | Digestive system |
| 172 | Carbohydrate digestion and absorption | 10 | 47 | 0.3441077 | 6.742110e-01 | ko04973 | Organismal Systems | Digestive system |
| 173 | Vitamin digestion and absorption | 7 | 32 | 0.3591466 | 6.996093e-01 | ko04977 | Organismal Systems | Digestive system |
| 174 | Pyrimidine metabolism | 14 | 69 | 0.3688867 | 7.144530e-01 | ko00240 | Metabolism | Nucleotide metabolism |
| 175 | mRNA surveillance pathway | 36 | 187 | 0.3765041 | 7.175966e-01 | ko03015 | Genetic Information Processing | Translation |
| 176 | Galactose metabolism | 9 | 43 | 0.3768979 | 7.175966e-01 | ko00052 | Metabolism | Carbohydrate metabolism |
| 177 | Nicotinate and nicotinamide metabolism | 9 | 43 | 0.3768979 | 7.175966e-01 | ko00760 | Metabolism | Metabolism of cofactors and vitamins |
| 178 | Alzheimer disease | 40 | 209 | 0.3824129 | 7.235938e-01 | ko05010 | Human Diseases | Neurodegenerative diseases |
| 179 | Amphetamine addiction | 18 | 91 | 0.3843421 | 7.235938e-01 | ko05031 | Human Diseases | Substance dependence |
| 180 | Dilated cardiomyopathy (DCM) | 26 | 134 | 0.3872943 | 7.251010e-01 | ko05414 | Human Diseases | Cardiovascular diseases |
| 181 | Aldosterone synthesis and secretion | 25 | 129 | 0.3932435 | 7.321716e-01 | ko04925 | Organismal Systems | Endocrine system |
| 182 | RNA transport | 58 | 309 | 0.4111778 | 7.613567e-01 | ko03013 | Genetic Information Processing | Translation |
| 183 | Proteasome | 11 | 55 | 0.4142266 | 7.628107e-01 | ko03050 | Genetic Information Processing | Folding, sorting and degradation |
| 184 | Non-homologous end-joining | 4 | 18 | 0.4179982 | 7.640743e-01 | ko03450 | Genetic Information Processing | Replication and repair |
| 185 | Arrhythmogenic right ventricular cardiomyopathy (ARVC) | 21 | 109 | 0.4194473 | 7.640743e-01 | ko05412 | Human Diseases | Cardiovascular diseases |
| 186 | Glutamatergic synapse | 28 | 147 | 0.4218559 | 7.643303e-01 | ko04724 | Organismal Systems | Nervous system |
| 187 | Terpenoid backbone biosynthesis | 6 | 29 | 0.4345719 | 7.831590e-01 | ko00900 | Metabolism | Metabolism of terpenoids and polyketides |
| 188 | RIG-I-like receptor signaling pathway | 16 | 83 | 0.4390242 | 7.869742e-01 | ko04622 | Organismal Systems | Immune system |
| 189 | Antifolate resistance | 8 | 40 | 0.4436884 | 7.876900e-01 | ko01523 | Human Diseases | Drug resistance: Antineoplastic |
| 190 | Inflammatory bowel disease (IBD) | 13 | 67 | 0.4440982 | 7.876900e-01 | ko05321 | Human Diseases | Immune diseases |
| 191 | Hippo signaling pathway | 74 | 400 | 0.4482217 | 7.908414e-01 | ko04390 | Environmental Information Processing | Signal transduction |
| 192 | Aminoacyl-tRNA biosynthesis | 12 | 62 | 0.4538639 | 7.966257e-01 | ko00970 | Genetic Information Processing | Translation |
| 193 | Non-alcoholic fatty liver disease (NAFLD) | 30 | 161 | 0.4689342 | 8.175448e-01 | ko04932 | Human Diseases | Endocrine and metabolic diseases |
| 194 | Thyroid cancer | 17 | 90 | 0.4706341 | 8.175448e-01 | ko05216 | Human Diseases | Cancers: Specific types |
| 195 | Butanoate metabolism | 7 | 36 | 0.4873303 | 8.382948e-01 | ko00650 | Metabolism | Carbohydrate metabolism |
| 196 | Pathogenic Escherichia coli infection | 19 | 102 | 0.4906706 | 8.382948e-01 | ko05130 | Human Diseases | Infectious diseases: Bacterial |
| 197 | Hypertrophic cardiomyopathy (HCM) | 23 | 124 | 0.4909588 | 8.382948e-01 | ko05410 | Human Diseases | Cardiovascular diseases |
| 198 | Human papillomavirus infection | 146 | 801 | 0.4925293 | 8.382948e-01 | ko05165 | Human Diseases | Infectious diseases: Viral |
| 199 | Long-term depression | 14 | 75 | 0.5008958 | 8.389564e-01 | ko04730 | Organismal Systems | Nervous system |
| 200 | Glyoxylate and dicarboxylate metabolism | 8 | 42 | 0.5025584 | 8.389564e-01 | ko00630 | Metabolism | Carbohydrate metabolism |
| 201 | Citrate cycle (TCA cycle) | 8 | 42 | 0.5025584 | 8.389564e-01 | ko00020 | Metabolism | Carbohydrate metabolism |
| 202 | Glycosphingolipid biosynthesis - ganglio series | 4 | 20 | 0.5046235 | 8.389564e-01 | ko00604 | Metabolism | Glycan biosynthesis and metabolism |
| 203 | Sulfur metabolism | 2 | 9 | 0.5061679 | 8.389564e-01 | ko00920 | Metabolism | Energy metabolism |
| 204 | Toll-like receptor signaling pathway | 23 | 125 | 0.5078549 | 8.389564e-01 | ko04620 | Organismal Systems | Immune system |
| 205 | Histidine metabolism | 5 | 26 | 0.5224163 | 8.588014e-01 | ko00340 | Metabolism | Amino acid metabolism |
| 206 | Cholesterol metabolism | 13 | 71 | 0.5346674 | 8.737858e-01 | ko04979 | Organismal Systems | Digestive system |
| 207 | Glycosylphosphatidylinositol (GPI)-anchor biosynthesis | 6 | 32 | 0.5367171 | 8.737858e-01 | ko00563 | Metabolism | Glycan biosynthesis and metabolism |
| 208 | Drug metabolism - other enzymes | 16 | 88 | 0.5406989 | 8.760362e-01 | ko00983 | Metabolism | Xenobiotics biodegradation and metabolism |
| 209 | Cortisol synthesis and secretion | 14 | 78 | 0.5648773 | 9.081515e-01 | ko04927 | Organismal Systems | Endocrine system |
| 210 | Riboflavin metabolism | 2 | 10 | 0.5659104 | 9.081515e-01 | ko00740 | Metabolism | Metabolism of cofactors and vitamins |
| 211 | Biosynthesis of unsaturated fatty acids | 6 | 33 | 0.5690149 | 9.088058e-01 | ko01040 | Metabolism | Lipid metabolism |
| 212 | Estrogen signaling pathway | 35 | 197 | 0.5853393 | 9.304686e-01 | ko04915 | Organismal Systems | Endocrine system |
| 213 | Bile secretion | 15 | 85 | 0.592402 | 9.320622e-01 | ko04976 | Organismal Systems | Digestive system |
| 214 | Glycolysis / Gluconeogenesis | 15 | 85 | 0.592402 | 9.320622e-01 | ko00010 | Metabolism | Carbohydrate metabolism |
| 215 | alpha-Linolenic acid metabolism | 6 | 34 | 0.6001706 | 9.320622e-01 | ko00592 | Metabolism | Lipid metabolism |
| 216 | Pentose phosphate pathway | 6 | 34 | 0.6001706 | 9.320622e-01 | ko00030 | Metabolism | Carbohydrate metabolism |
| 217 | Fatty acid elongation | 6 | 34 | 0.6001706 | 9.320622e-01 | ko00062 | Metabolism | Lipid metabolism |
| 218 | Vascular smooth muscle contraction | 32 | 182 | 0.6096298 | 9.424094e-01 | ko04270 | Organismal Systems | Circulatory system |
| 219 | Endocrine and other factor-regulated calcium reabsorption | 11 | 64 | 0.630072 | 9.648382e-01 | ko04961 | Organismal Systems | Excretory system |
| 220 | PPAR signaling pathway | 15 | 87 | 0.6307982 | 9.648382e-01 | ko03320 | Organismal Systems | Endocrine system |
| 221 | Insect hormone biosynthesis | 1 | 5 | 0.6327277 | 9.648382e-01 | ko00981 | Metabolism | Metabolism of terpenoids and polyketides |
| 222 | Parkinson disease | 25 | 145 | 0.6458861 | 9.804667e-01 | ko05012 | Human Diseases | Neurodegenerative diseases |
| 223 | Synthesis and degradation of ketone bodies | 2 | 12 | 0.6691589 | 1.000000e+00 | ko00072 | Metabolism | Lipid metabolism |
| 224 | Purine metabolism | 30 | 176 | 0.6789944 | 1.000000e+00 | ko00230 | Metabolism | Nucleotide metabolism |
| 225 | Other types of O-glycan biosynthesis | 7 | 43 | 0.685938 | 1.000000e+00 | ko00514 | Metabolism | Glycan biosynthesis and metabolism |
| 226 | Glycosaminoglycan biosynthesis - heparan sulfate / heparin | 5 | 31 | 0.6868083 | 1.000000e+00 | ko00534 | Metabolism | Glycan biosynthesis and metabolism |
| 227 | Malaria | 9 | 55 | 0.6884038 | 1.000000e+00 | ko05144 | Human Diseases | Infectious diseases: Parasitic |
| 228 | 2-Oxocarboxylic acid metabolism | 4 | 25 | 0.6899887 | 1.000000e+00 | ko01210 | Metabolism | Global and overview maps |
| 229 | One carbon pool by folate | 4 | 25 | 0.6899887 | 1.000000e+00 | ko00670 | Metabolism | Metabolism of cofactors and vitamins |
| 230 | Glutathione metabolism | 11 | 67 | 0.6923116 | 1.000000e+00 | ko00480 | Metabolism | Metabolism of other amino acids |
| 231 | Neomycin, kanamycin and gentamicin biosynthesis | 1 | 6 | 0.6994133 | 1.000000e+00 | ko00524 | Metabolism | Biosynthesis of other secondary metabolites |
| 232 | Melanogenesis | 56 | 327 | 0.7081856 | 1.000000e+00 | ko04916 | Organismal Systems | Endocrine system |
| 233 | D-Glutamine and D-glutamate metabolism | 2 | 13 | 0.7128353 | 1.000000e+00 | ko00471 | Metabolism | Metabolism of other amino acids |
| 234 | Wnt signaling pathway | 69 | 402 | 0.7177736 | 1.000000e+00 | ko04310 | Environmental Information Processing | Signal transduction |
| 235 | Serotonergic synapse | 22 | 133 | 0.719355 | 1.000000e+00 | ko04726 | Organismal Systems | Nervous system |
| 236 | Pertussis | 17 | 104 | 0.7221448 | 1.000000e+00 | ko05133 | Human Diseases | Infectious diseases: Bacterial |
| 237 | Platelet activation | 55 | 323 | 0.7230992 | 1.000000e+00 | ko04611 | Organismal Systems | Immune system |
| 238 | Breast cancer | 67 | 392 | 0.7285303 | 1.000000e+00 | ko05224 | Human Diseases | Cancers: Specific types |
| 239 | Leishmaniasis | 14 | 87 | 0.7327341 | 1.000000e+00 | ko05140 | Human Diseases | Infectious diseases: Parasitic |
| 240 | Thiamine metabolism | 9 | 58 | 0.7493081 | 1.000000e+00 | ko00730 | Metabolism | Metabolism of cofactors and vitamins |
| 241 | Circadian rhythm - fly | 1 | 7 | 0.7539939 | 1.000000e+00 | ko04711 | Organismal Systems | Environmental adaptation |
| 242 | Selenocompound metabolism | 3 | 21 | 0.7619756 | 1.000000e+00 | ko00450 | Metabolism | Metabolism of other amino acids |
| 243 | Prion diseases | 9 | 59 | 0.7676604 | 1.000000e+00 | ko05020 | Human Diseases | Neurodegenerative diseases |
| 244 | IL-17 signaling pathway | 29 | 178 | 0.7689769 | 1.000000e+00 | ko04657 | Organismal Systems | Immune system |
| 245 | AGE-RAGE signaling pathway in diabetic complications | 50 | 302 | 0.7867734 | 1.000000e+00 | ko04933 | Human Diseases | Endocrine and metabolic diseases |
| 246 | Tryptophan metabolism | 8 | 54 | 0.7884609 | 1.000000e+00 | ko00380 | Metabolism | Amino acid metabolism |
| 247 | Renin secretion | 15 | 97 | 0.7915163 | 1.000000e+00 | ko04924 | Organismal Systems | Endocrine system |
| 248 | Phenylalanine, tyrosine and tryptophan biosynthesis | 1 | 8 | 0.7986662 | 1.000000e+00 | ko00400 | Metabolism | Amino acid metabolism |
| 249 | Starch and sucrose metabolism | 6 | 42 | 0.7993921 | 1.000000e+00 | ko00500 | Metabolism | Carbohydrate metabolism |
| 250 | Synaptic vesicle cycle | 15 | 98 | 0.804416 | 1.000000e+00 | ko04721 | Organismal Systems | Nervous system |
| 251 | Salivary secretion | 25 | 158 | 0.805477 | 1.000000e+00 | ko04970 | Organismal Systems | Digestive system |
| 252 | Alcoholism | 36 | 223 | 0.8067955 | 1.000000e+00 | ko05034 | Human Diseases | Substance dependence |
| 253 | Metabolic pathways | 283 | 1629 | 0.8128069 | 1.000000e+00 | ko01100 | Metabolism | Global and overview maps |
| 254 | Phagosome | 36 | 224 | 0.8151191 | 1.000000e+00 | ko04145 | Cellular Processes | Transport and catabolism |
| 255 | Cushing syndrome | 64 | 387 | 0.8151966 | 1.000000e+00 | ko04934 | Human Diseases | Endocrine and metabolic diseases |
| 256 | Autophagy - other | 6 | 43 | 0.81779 | 1.000000e+00 | ko04136 | Cellular Processes | Transport and catabolism |
| 257 | Herpes simplex infection | 57 | 347 | 0.818616 | 1.000000e+00 | ko05168 | Human Diseases | Infectious diseases: Viral |
| 258 | Cysteine and methionine metabolism | 8 | 56 | 0.8214622 | 1.000000e+00 | ko00270 | Metabolism | Amino acid metabolism |
| 259 | Glycosaminoglycan biosynthesis - chondroitin sulfate / dermatan sulfate | 3 | 24 | 0.8376253 | 1.000000e+00 | ko00532 | Metabolism | Glycan biosynthesis and metabolism |
| 260 | Ribosome biogenesis in eukaryotes | 20 | 132 | 0.8443159 | 1.000000e+00 | ko03008 | Genetic Information Processing | Translation |
| 261 | Drug metabolism - cytochrome P450 | 11 | 77 | 0.8493791 | 1.000000e+00 | ko00982 | Metabolism | Xenobiotics biodegradation and metabolism |
| 262 | Mineral absorption | 8 | 58 | 0.8503331 | 1.000000e+00 | ko04978 | Organismal Systems | Digestive system |
| 263 | Carbon metabolism | 25 | 163 | 0.8510812 | 1.000000e+00 | ko01200 | Metabolism | Global and overview maps |
| 264 | Viral myocarditis | 13 | 90 | 0.8546474 | 1.000000e+00 | ko05416 | Human Diseases | Cardiovascular diseases |
| 265 | Glycosaminoglycan degradation | 3 | 25 | 0.8577575 | 1.000000e+00 | ko00531 | Metabolism | Glycan biosynthesis and metabolism |
| 266 | Primary bile acid biosynthesis | 2 | 18 | 0.86448 | 1.000000e+00 | ko00120 | Metabolism | Lipid metabolism |
| 267 | Arginine biosynthesis | 4 | 33 | 0.8739051 | 1.000000e+00 | ko00220 | Metabolism | Amino acid metabolism |
| 268 | Maturity onset diabetes of the young | 4 | 33 | 0.8739051 | 1.000000e+00 | ko04950 | Human Diseases | Endocrine and metabolic diseases |
| 269 | Other glycan degradation | 3 | 26 | 0.8756664 | 1.000000e+00 | ko00511 | Metabolism | Glycan biosynthesis and metabolism |
| 270 | Relaxin signaling pathway | 54 | 340 | 0.8796349 | 1.000000e+00 | ko04926 | Organismal Systems | Endocrine system |
| 271 | Antigen processing and presentation | 18 | 124 | 0.8814237 | 1.000000e+00 | ko04612 | Organismal Systems | Immune system |
| 272 | Thyroid hormone synthesis | 13 | 93 | 0.8841506 | 1.000000e+00 | ko04918 | Organismal Systems | Endocrine system |
| 273 | Spliceosome | 32 | 210 | 0.8851622 | 1.000000e+00 | ko03040 | Genetic Information Processing | Transcription |
| 274 | Ovarian steroidogenesis | 8 | 61 | 0.886497 | 1.000000e+00 | ko04913 | Organismal Systems | Endocrine system |
| 275 | Sulfur relay system | 5 | 41 | 0.8886093 | 1.000000e+00 | ko04122 | Genetic Information Processing | Folding, sorting and degradation |
| 276 | Ether lipid metabolism | 9 | 68 | 0.8909754 | 1.000000e+00 | ko00565 | Metabolism | Lipid metabolism |
| 277 | Tyrosine metabolism | 7 | 55 | 0.8933921 | 1.000000e+00 | ko00350 | Metabolism | Amino acid metabolism |
| 278 | Folate biosynthesis | 5 | 42 | 0.9006656 | 1.000000e+00 | ko00790 | Metabolism | Metabolism of cofactors and vitamins |
| 279 | Alanine, aspartate and glutamate metabolism | 6 | 49 | 0.9016383 | 1.000000e+00 | ko00250 | Metabolism | Amino acid metabolism |
| 280 | Amyotrophic lateral sclerosis (ALS) | 10 | 76 | 0.9043349 | 1.000000e+00 | ko05014 | Human Diseases | Neurodegenerative diseases |
| 281 | Arginine and proline metabolism | 8 | 63 | 0.9063197 | 1.000000e+00 | ko00330 | Metabolism | Amino acid metabolism |
| 282 | Biosynthesis of amino acids | 12 | 90 | 0.9120123 | 1.000000e+00 | ko01230 | Metabolism | Global and overview maps |
| 283 | Proximal tubule bicarbonate reclamation | 4 | 36 | 0.9131918 | 1.000000e+00 | ko04964 | Organismal Systems | Excretory system |
| 284 | Pantothenate and CoA biosynthesis | 2 | 21 | 0.9158325 | 1.000000e+00 | ko00770 | Metabolism | Metabolism of cofactors and vitamins |
| 285 | Nitrogen metabolism | 3 | 29 | 0.9179453 | 1.000000e+00 | ko00910 | Metabolism | Energy metabolism |
| 286 | Steroid biosynthesis | 3 | 29 | 0.9179453 | 1.000000e+00 | ko00100 | Metabolism | Lipid metabolism |
| 287 | Amoebiasis | 71 | 450 | 0.918922 | 1.000000e+00 | ko05146 | Human Diseases | Infectious diseases: Parasitic |
| 288 | Huntington disease | 48 | 314 | 0.9220789 | 1.000000e+00 | ko05016 | Human Diseases | Neurodegenerative diseases |
| 289 | Legionellosis | 9 | 72 | 0.9249999 | 1.000000e+00 | ko05134 | Human Diseases | Infectious diseases: Bacterial |
| 290 | Intestinal immune network for IgA production | 6 | 52 | 0.9293538 | 1.000000e+00 | ko04672 | Organismal Systems | Immune system |
| 291 | Cardiac muscle contraction | 11 | 86 | 0.9295081 | 1.000000e+00 | ko04260 | Organismal Systems | Circulatory system |
| 292 | Mucin type O-glycan biosynthesis | 4 | 38 | 0.9329141 | 1.000000e+00 | ko00512 | Metabolism | Glycan biosynthesis and metabolism |
| 293 | ECM-receptor interaction | 46 | 305 | 0.9329711 | 1.000000e+00 | ko04512 | Environmental Information Processing | Signaling molecules and interaction |
| 294 | Basal cell carcinoma | 40 | 269 | 0.9343912 | 1.000000e+00 | ko05217 | Human Diseases | Cancers: Specific types |
| 295 | Basal transcription factors | 11 | 88 | 0.9412339 | 1.000000e+00 | ko03022 | Genetic Information Processing | Transcription |
| 296 | Porphyrin and chlorophyll metabolism | 6 | 54 | 0.9437641 | 1.000000e+00 | ko00860 | Metabolism | Metabolism of cofactors and vitamins |
| 297 | Retinol metabolism | 10 | 82 | 0.9451525 | 1.000000e+00 | ko00830 | Metabolism | Metabolism of cofactors and vitamins |
| 298 | Glycosphingolipid biosynthesis - lacto and neolacto series | 3 | 32 | 0.946686 | 1.000000e+00 | ko00601 | Metabolism | Glycan biosynthesis and metabolism |
| 299 | RNA polymerase | 12 | 96 | 0.9478779 | 1.000000e+00 | ko03020 | Genetic Information Processing | Transcription |
| 300 | African trypanosomiasis | 4 | 40 | 0.9484852 | 1.000000e+00 | ko05143 | Human Diseases | Infectious diseases: Parasitic |
| 301 | Fat digestion and absorption | 5 | 48 | 0.9517245 | 1.000000e+00 | ko04975 | Organismal Systems | Digestive system |
| 302 | Glycine, serine and threonine metabolism | 4 | 41 | 0.9549582 | 1.000000e+00 | ko00260 | Metabolism | Amino acid metabolism |
| 303 | Type I diabetes mellitus | 5 | 50 | 0.9624873 | 1.000000e+00 | ko04940 | Human Diseases | Endocrine and metabolic diseases |
| 304 | Metabolism of xenobiotics by cytochrome P450 | 10 | 86 | 0.9630168 | 1.000000e+00 | ko00980 | Metabolism | Xenobiotics biodegradation and metabolism |
| 305 | Cocaine addiction | 6 | 60 | 0.9725329 | 1.000000e+00 | ko05030 | Human Diseases | Substance dependence |
| 306 | Taurine and hypotaurine metabolism | 1 | 18 | 0.9728775 | 1.000000e+00 | ko00430 | Metabolism | Metabolism of other amino acids |
| 307 | Oxidative phosphorylation | 18 | 144 | 0.9741034 | 1.000000e+00 | ko00190 | Metabolism | Energy metabolism |
| 308 | Glycosphingolipid biosynthesis - globo and isoglobo series | 1 | 19 | 0.9778057 | 1.000000e+00 | ko00603 | Metabolism | Glycan biosynthesis and metabolism |
| 309 | Nicotine addiction | 6 | 63 | 0.9811139 | 1.000000e+00 | ko05033 | Human Diseases | Substance dependence |
| 310 | Primary immunodeficiency | 3 | 39 | 0.9814279 | 1.000000e+00 | ko05340 | Human Diseases | Immune diseases |
| 311 | Linoleic acid metabolism | 3 | 39 | 0.9814279 | 1.000000e+00 | ko00591 | Metabolism | Lipid metabolism |
| 312 | Pentose and glucuronate interconversions | 3 | 39 | 0.9814279 | 1.000000e+00 | ko00040 | Metabolism | Carbohydrate metabolism |
| 313 | Cell adhesion molecules (CAMs) | 23 | 182 | 0.9826949 | 1.000000e+00 | ko04514 | Environmental Information Processing | Signaling molecules and interaction |
| 314 | Collecting duct acid secretion | 2 | 32 | 0.9867345 | 1.000000e+00 | ko04966 | Organismal Systems | Excretory system |
| 315 | Phototransduction | 5 | 58 | 0.9869773 | 1.000000e+00 | ko04744 | Organismal Systems | Sensory system |
| 316 | Phenylalanine metabolism | 1 | 22 | 0.9878397 | 1.000000e+00 | ko00360 | Metabolism | Amino acid metabolism |
| 317 | Chemical carcinogenesis | 9 | 93 | 0.9922144 | 1.000000e+00 | ko05204 | Human Diseases | Cancers: Overview |
| 318 | Sphingolipid metabolism | 5 | 63 | 0.9935028 | 1.000000e+00 | ko00600 | Metabolism | Lipid metabolism |
| 319 | Cytosolic DNA-sensing pathway | 6 | 72 | 0.9941908 | 1.000000e+00 | ko04623 | Organismal Systems | Immune system |
| 320 | Graft-versus-host disease | 2 | 37 | 0.9944698 | 1.000000e+00 | ko05332 | Human Diseases | Immune diseases |
| 321 | Renin-angiotensin system | 1 | 26 | 0.994549 | 1.000000e+00 | ko04614 | Organismal Systems | Endocrine system |
| 322 | Allograft rejection | 2 | 41 | 0.9972835 | 1.000000e+00 | ko05330 | Human Diseases | Immune diseases |
| 323 | Ascorbate and aldarate metabolism | 1 | 31 | 0.9980012 | 1.000000e+00 | ko00053 | Metabolism | Carbohydrate metabolism |
| 324 | Taste transduction | 8 | 98 | 0.9984319 | 1.000000e+00 | ko04742 | Organismal Systems | Sensory system |
| 325 | Arachidonic acid metabolism | 5 | 87 | 0.9998225 | 1.000000e+00 | ko00590 | Metabolism | Lipid metabolism |
| 326 | Rheumatoid arthritis | 6 | 102 | 0.9999261 | 1.000000e+00 | ko05323 | Human Diseases | Immune diseases |
| 327 | Steroid hormone biosynthesis | 3 | 74 | 0.9999464 | 1.000000e+00 | ko00140 | Metabolism | Lipid metabolism |
| 328 | Autoimmune thyroid disease | 2 | 63 | 0.9999515 | 1.000000e+00 | ko05320 | Human Diseases | Immune diseases |
| 329 | Cytokine-cytokine receptor interaction | 33 | 318 | 0.9999605 | 1.000000e+00 | ko04060 | Environmental Information Processing | Signaling molecules and interaction |
| 330 | Staphylococcus aureus infection | 3 | 83 | 0.9999891 | 1.000000e+00 | ko05150 | Human Diseases | Infectious diseases: Bacterial |
| 331 | Ribosome | 16 | 202 | 0.9999901 | 1.000000e+00 | ko03010 | Genetic Information Processing | Translation |
| 332 | Hematopoietic cell lineage | 6 | 118 | 0.999994 | 1.000000e+00 | ko04640 | Organismal Systems | Immune system |
| 333 | Systemic lupus erythematosus | 9 | 149 | 0.9999961 | 1.000000e+00 | ko05322 | Human Diseases | Immune diseases |
| 334 | Complement and coagulation cascades | 6 | 127 | 0.9999986 | 1.000000e+00 | ko04610 | Organismal Systems | Immune system |
| 335 | Protein digestion and absorption | 35 | 480 | 1 | 1.000000e+00 | ko04974 | Organismal Systems | Digestive system |
| 336 | Olfactory transduction | 16 | 457 | 1 | 1.000000e+00 | ko04740 | Organismal Systems | Sensory system |
| 337 | Neuroactive ligand-receptor interaction | 27 | 404 | 1 | 1.000000e+00 | ko04080 | Environmental Information Processing | Signaling molecules and interaction |

| # | Pathway | Differentially expressed genes |
| --- | --- | --- |
| 1 | Endocytosis | 3949, 5590, 128866, 117583, 867, 80223, 9921, 23362, 1785, 51430, 2869, 10095, 3480, 122773, 377, 118813, 51100, 51324, 8411, 147179, 6645, 27131, 9922, 8603, 4087, 84629, 116985, 196441, 57154, 23111, 868, 10890, 8853, 57403, 9980, 58513, 408, 6455, 23396, 79729, 1212, 50807, 83737, 55680, 6642, 11033, 58533, 23327, 9146, 9135, 64788, 89853, 157, 57149, 2060, 9101, 8394, 129531, 51123, 23624, 829, 55616, 9685, 6744, 161, 80230, 9267, 55737, 84954, 60685, 10193, 51028, 5156, 10109, 116984, 30846, 156, 9744, 7879, 4193, 9265, 158219, 116987, 9727, 60682, 23041, 23033, 7037, 22905, 152002, 23527, 23550, 146691, 57609, 84364, 10564, 23201, 9815, 57590, 54540, 5868, 93343, 1956, 23325, 116983, 11059, 26286, 2217, 22996, 8976, 22902, 5337, 10617, 51534, 832, 23360, 144100, 25978, 151556, 1213, 125950, 3799, 151195, 64750, 257160, 375, 219771, 26040, 4088, 116986, 26207 |
| 2 | Ubiquitin mediated proteolysis | 8452, 867, 7326, 7337, 10054, 55255, 23295, 118424, 25998, 51366, 64326, 8065, 57154, 868, 7328, 26259, 11060, 26272, 51588, 1642, 83737, 7322, 23327, 4214, 57448, 55294, 10075, 23624, 8925, 89910, 54926, 91133, 10277, 7323, 3093, 94097, 4193, 29882, 158219, 55120, 9817, 8924, 119559, 996, 92912, 9354, 55622, 80820, 9039, 4281, 84456, 11059, 22996, 7428, 4591, 8453, 22888, 144100, 84447, 9978, 8881, 23291, 54799, 23335, 26091, 25898, 64750, 8451, 339487 |
| 3 | AMPK signaling pathway | 1374, 23216, 3480, 55844, 5468, 10890, 2194, 6720, 2308, 29904, 51719, 7249, 32, 57149, 5291, 5527, 91050, 23417, 51422, 5214, 57186, 5521, 23411, 6198, 64764, 90993, 5290, 208, 1080, 284001, 31, 5211, 55012, 5208, 2309, 1994, 3643, 2997, 207, 79602, 57521, 10645, 5525, 1938, 5296, 200186, 5210, 3156, 3172, 9586, 6009, 5295, 5106 |
| 4 | Phosphatidylinositol signaling system | 200576, 8527, 3632, 51196, 1606, 8897, 8821, 113026, 8760, 23396, 5287, 80271, 5578, 5291, 5297, 8394, 3707, 5286, 8526, 5579, 57732, 5336, 5290, 9807, 5298, 3633, 3631, 10423, 3636, 55361, 84288, 8871, 3709, 51447, 64419, 64800, 5332, 5296, 5289, 5305, 805, 56623, 64747, 5728, 5295, 4534, 22908, 3705 |
| 5 | Inositol phosphate metabolism | 200576, 3632, 51196, 8897, 8821, 113026, 23396, 5287, 80271, 5291, 5297, 8394, 3707, 5286, 57732, 5336, 5290, 5298, 3633, 3631, 10423, 3636, 55361, 8871, 64419, 5332, 5289, 9562, 5305, 80127, 56623, 64747, 5728, 4534, 22908, 3705 |
| 6 | Insulin resistance | 5590, 79573, 1374, 2673, 133522, 5599, 3551, 10724, 6720, 2308, 5581, 32, 5291, 6197, 5500, 5465, 5781, 51422, 5579, 27330, 55147, 6198, 5602, 5792, 64764, 90993, 5290, 208, 5524, 6774, 3643, 11000, 2997, 207, 5296, 200186, 6196, 9586, 5770, 5728, 5295, 5106 |
| 7 | Protein processing in endoplasmic reticulum | 201595, 64224, 29979, 23231, 7326, 23193, 22872, 596, 118424, 55757, 5599, 11231, 4780, 56886, 7186, 165324, 7322, 10427, 54788, 4217, 10484, 2923, 22926, 6185, 10483, 267, 5589, 10277, 440275, 55666, 7323, 5602, 5034, 821, 64374, 9352, 23645, 824, 30001, 823, 10130, 4287, 6745, 27248, 8720, 10905, 5886, 80331, 653583, 9871, 9679, 80318, 3326, 81567, 10802, 9709, 56681, 84447, 9978, 5887, 51360, 55768, 6400, 7494, 339487 |
| 8 | HIF-1 signaling pathway | 3460, 5163, 3480, 596, 817, 388403, 5578, 5291, 3091, 5594, 2033, 5604, 1387, 2064, 5579, 4843, 6198, 285, 5336, 3099, 5290, 208, 6774, 818, 7037, 5162, 3643, 207, 1956, 5296, 7428, 2872, 8453, 9978, 317649, 5295, 4055, 1536 |
| 9 | Neurotrophin signaling pathway | 8615, 11213, 5663, 6655, 596, 5599, 3551, 817, 2889, 4217, 388403, 4214, 7531, 6654, 5291, 5594, 6197, 114815, 5781, 10019, 5604, 27330, 5602, 5336, 5290, 208, 818, 9252, 2309, 57498, 6272, 84288, 4915, 25970, 4794, 207, 79706, 64800, 4804, 25, 5296, 5603, 4793, 805, 6196, 5906, 5894, 9261, 814, 5295, 11108 |
| 10 | Regulation of actin cytoskeleton | 3683, 3679, 200576, 10095, 221178, 85464, 6655, 57121, 5747, 23365, 54961, 81, 3687, 4638, 7410, 6548, 10458, 23396, 2909, 10985, 388403, 54847, 79703, 6654, 1729, 23191, 5291, 6093, 8516, 1793, 5594, 10788, 5500, 8394, 3675, 1730, 55740, 2260, 5604, 57459, 8826, 60685, 2934, 5156, 10109, 54776, 5290, 3985, 3694, 145282, 57407, 5058, 7074, 5159, 54434, 57609, 624, 26999, 55691, 7414, 1902, 130872, 3688, 79140, 1956, 87, 10451, 752, 5296, 8976, 85477, 9564, 60, 5305, 7430, 64423, 5894, 80206, 55845, 3693, 23150, 5295, 71, 3672, 3071 |
| 11 | Thyroid hormone signaling pathway | 9969, 23389, 51196, 22889, 25992, 113026, 6548, 2308, 8648, 7068, 388403, 5578, 25942, 7249, 5291, 3091, 5594, 10499, 6256, 2033, 5604, 1387, 57186, 5579, 7067, 5336, 5290, 208, 4193, 5208, 5469, 55689, 9862, 207, 114786, 205428, 842, 5332, 5296, 10025, 60, 54980, 6009, 5894, 286046, 6772, 5295, 71, 8202 |
| 12 | Hepatitis B | 6655, 6776, 596, 5599, 3551, 4087, 1642, 7040, 6416, 3339, 388403, 4214, 5578, 4773, 6654, 5291, 5594, 4775, 2033, 55225, 5604, 1387, 5579, 5602, 64764, 90993, 5290, 208, 6774, 114609, 7419, 10906, 207, 4615, 3454, 6775, 6777, 842, 5296, 843, 5603, 317, 64135, 9586, 2185, 5894, 57506, 51015, 125950, 6772, 5295, 3717, 4772, 84514, 4088 |
| 13 | Fc gamma R-mediated phagocytosis | 10095, 122773, 7408, 1794, 8853, 7410, 23396, 50807, 388403, 5581, 5578, 5291, 5594, 8394, 5604, 55616, 5579, 6198, 60685, 2934, 10109, 5336, 5290, 208, 3985, 5058, 4651, 57609, 3636, 207, 10451, 5296, 8976, 9644, 5337, 85477, 6850, 4067, 5894, 51466, 5295, 9846 |
| 14 | B cell receptor signaling pathway | 6655, 3551, 10892, 7410, 388403, 4773, 6654, 5291, 5594, 4775, 5604, 5579, 5336, 5290, 208, 5530, 3636, 695, 4794, 207, 10451, 5296, 4793, 6850, 4067, 5894, 5295, 4772 |
| 15 | Osteoclast differentiation | 8792, 3460, 8878, 10990, 10288, 11026, 353514, 3726, 3932, 5599, 3551, 5468, 9020, 2355, 7186, 7040, 5971, 388403, 4773, 5291, 5594, 11024, 10859, 55225, 5604, 5602, 5336, 3455, 5290, 208, 5530, 695, 207, 3454, 5296, 9644, 23547, 5603, 6850, 2354, 125950, 6772, 814, 5295, 4772, 9846 |
| 16 | Prostate cancer | 6655, 3480, 596, 3551, 2308, 388403, 6654, 5291, 5594, 3429, 2033, 2260, 5604, 1387, 2064, 5156, 64764, 90993, 5290, 208, 4193, 6692, 5159, 10653, 207, 6934, 1956, 842, 5296, 3326, 84918, 9586, 5894, 5728, 7113, 5295, 2537 |
| 17 | EGFR tyrosine kinase inhibitor resistance | 6655, 3480, 596, 4763, 558, 388403, 5578, 6654, 5291, 5594, 5604, 2064, 5579, 6198, 5156, 5336, 5290, 208, 6774, 5159, 2309, 207, 2621, 598, 2065, 1956, 5296, 4233, 5894, 317649, 5728, 5295, 3717 |
| 18 | Rap1 signaling pathway | 3683, 260425, 5590, 117583, 9771, 57568, 51196, 3480, 26037, 57121, 7057, 115, 7408, 2771, 51195, 10235, 83660, 2889, 9223, 388403, 5578, 113, 5291, 111, 5594, 23094, 999, 2260, 5604, 5900, 5579, 5156, 285, 1500, 5290, 208, 9855, 8935, 6494, 79613, 7074, 5159, 266747, 65059, 3643, 84288, 207, 5899, 1902, 83786, 83593, 3688, 25865, 135, 1956, 64800, 5332, 4804, 5296, 4233, 9564, 60, 5603, 889, 805, 8502, 5906, 5894, 51466, 51015, 5295, 109, 71, 7423, 54518 |
| 19 | Central carbon metabolism in cancer | 5163, 2744, 388403, 5291, 3091, 5594, 5214, 2260, 5604, 2064, 5156, 3099, 5290, 208, 5162, 5211, 5159, 207, 1956, 5296, 4233, 5894, 5728, 5295 |
| 20 | Cellular senescence | 472, 677, 8615, 90550, 8878, 7417, 4087, 3487, 54822, 132660, 2308, 7040, 388403, 4773, 7249, 5291, 891, 5594, 5500, 4775, 5604, 4361, 57186, 23411, 5290, 208, 4193, 5530, 824, 823, 28996, 2309, 7419, 11200, 896, 84288, 3709, 2113, 207, 83593, 64800, 2217, 5296, 286826, 5603, 805, 291, 6009, 5894, 51015, 5728, 9261, 5295, 23291, 23335, 4772, 4088 |
| 21 | Pancreatic cancer | 10443, 5599, 3551, 4087, 7040, 388403, 5291, 5594, 5604, 2064, 5900, 6198, 5602, 83941, 5290, 208, 6774, 266747, 207, 5899, 598, 1956, 842, 5296, 5894, 6772, 5295, 4088 |
| 22 | Th17 cell differentiation | 3460, 6776, 3932, 5599, 3551, 4087, 3566, 7040, 861, 388403, 4773, 3091, 5594, 6256, 4775, 55225, 5602, 149233, 6774, 5530, 919, 3572, 3594, 4794, 50615, 6777, 9466, 5603, 3326, 4793, 125950, 6772, 3717, 4772, 84514, 4088 |
| 23 | Non-small cell lung cancer | 6655, 6776, 388403, 5578, 6654, 5291, 5594, 6256, 5604, 2272, 2064, 5579, 83941, 5336, 5290, 208, 6774, 2309, 207, 83593, 6777, 1956, 842, 5296, 5894, 5295, 84514 |
| 24 | Insulin signaling pathway | 5590, 867, 5257, 6655, 5599, 3551, 868, 2194, 6720, 2308, 23265, 2889, 388403, 6654, 7249, 32, 5260, 57149, 5291, 5594, 5500, 5573, 5140, 51422, 5604, 57186, 6198, 5602, 5792, 3099, 5290, 208, 284001, 5576, 31, 3636, 3643, 84288, 2997, 207, 57521, 79706, 64800, 5296, 2872, 805, 6009, 5894, 317649, 5770, 5295, 5106, 5575 |
| 25 | Leukocyte transendothelial migration | 3683, 1364, 5747, 7408, 2771, 51195, 81, 1495, 7410, 2909, 10985, 5578, 5291, 6093, 5781, 5579, 5336, 1500, 5290, 145282, 57407, 6494, 1366, 55691, 7414, 100506658, 83593, 3688, 87, 10451, 5296, 9644, 9564, 60, 5603, 7430, 8502, 5906, 2185, 51466, 23150, 5295, 71, 83692, 1536 |
| 26 | Longevity regulating pathway | 26060, 3480, 115, 5468, 79813, 2308, 7249, 113, 57149, 5291, 111, 51422, 57186, 23411, 6198, 64764, 90993, 5290, 208, 2309, 3643, 207, 9474, 79602, 57521, 10645, 5296, 199870, 9586, 6009, 317649, 814, 5295, 109, 9821 |
| 27 | ErbB signaling pathway | 867, 6655, 6776, 5747, 5599, 817, 868, 6416, 388403, 5578, 6654, 27, 5291, 5594, 5604, 2064, 5579, 6198, 5602, 5336, 5290, 208, 818, 5058, 207, 2065, 6777, 1956, 25, 5296, 5894, 5295, 84514 |
| 28 | Adherens junction | 5819, 117583, 3480, 4008, 4087, 81, 51701, 1495, 10458, 388403, 5795, 5797, 5594, 999, 2033, 2260, 1387, 2064, 8826, 60685, 5792, 1500, 9855, 79613, 57609, 3643, 6934, 7414, 1956, 87, 8976, 4233, 60, 8502, 9231, 5770, 71, 4088 |
| 29 | Jak-STAT signaling pathway | 3460, 54497, 3977, 6655, 6776, 596, 1439, 51588, 25938, 3566, 163702, 6654, 5291, 5781, 2033, 55225, 1387, 149233, 5156, 3455, 5290, 208, 3595, 6774, 22874, 5159, 146691, 3572, 896, 3594, 5008, 207, 50615, 598, 5771, 3454, 58985, 6775, 6777, 1956, 5296, 9466, 5894, 3597, 125950, 6772, 5295, 3717, 84514 |
| 30 | Apoptosis | 472, 8837, 596, 6645, 5599, 3551, 1520, 1439, 84823, 9020, 153090, 7186, 8717, 143, 4217, 388403, 203197, 835, 5291, 5594, 1522, 5604, 50809, 5602, 23143, 27297, 5290, 208, 146712, 824, 4034, 8743, 823, 1509, 5783, 1508, 1676, 3709, 207, 57470, 64081, 23184, 598, 4000, 842, 5296, 60, 843, 2021, 317, 4013, 5894, 5295, 71, 91663, 1075 |
| 31 | MAPK signaling pathway - fly | 7514, 5495, 22821, 6239, 6655, 79370, 5599, 2120, 6416, 7322, 4217, 388403, 6654, 26010, 5594, 5781, 5604, 10527, 7323, 5602, 4293, 22874, 116966, 2113, 83786, 65244, 1956, 79718, 51513, 4216, 5603, 29888, 51015, 23335, 154043, 339487 |
| 32 | Sphingolipid signaling pathway | 5590, 596, 5599, 2771, 55844, 7186, 8717, 4217, 388403, 5581, 5578, 5291, 6093, 5527, 4363, 5594, 91050, 253782, 5604, 5521, 5579, 5602, 5290, 208, 340485, 145282, 55012, 1509, 624, 207, 5332, 5525, 5296, 5337, 9517, 5603, 5894, 5728, 5295, 427, 9846 |
| 33 | Pathways in cancer | 4040, 125488, 3460, 867, 26060, 54462, 8312, 10443, 376940, 6655, 1488, 3480, 3728, 2948, 2946, 2944, 2782, 3714, 2788, 4041, 6776, 596, 57121, 22889, 57488, 5747, 115, 5599, 2771, 23365, 3551, 817, 1439, 4087, 10681, 7257, 5468, 4780, 25992, 1855, 22982, 1495, 10235, 25938, 3566, 2308, 7704, 7186, 7040, 57587, 8648, 861, 1487, 388403, 5578, 150864, 6654, 7188, 91355, 129293, 113, 5291, 182, 6093, 111, 3091, 9842, 100505549, 5594, 6256, 999, 4292, 3675, 2033, 79591, 199990, 23635, 23051, 2260, 5604, 1387, 2064, 5900, 51684, 50809, 5579, 4843, 51379, 6198, 54879, 5602, 83941, 149233, 5156, 5336, 113452, 3455, 11333, 1287, 5290, 208, 3595, 4193, 119504, 1613, 6774, 818, 84928, 145282, 79613, 6608, 22874, 9817, 5159, 80856, 8076, 9252, 7690, 266747, 55200, 59345, 148362, 57609, 3572, 5939, 113201, 285237, 896, 3594, 84288, 10906, 624, 162427, 91748, 2113, 207, 5899, 7170, 6934, 59339, 162073, 51316, 79647, 780776, 54477, 1902, 83593, 598, 26033, 3688, 2034, 65244, 1039, 79932, 3454, 3915, 64399, 100134938, 1630, 6775, 205428, 54331, 6777, 1956, 64800, 842, 5332, 25, 5296, 120400, 1612, 4233, 7428, 199870, 8453, 100129792, 6414, 55654, 3326, 159195, 1728, 54980, 4258, 805, 317, 146177, 5894, 9978, 3597, 196074, 140775, 5728, 6772, 5295, 109, 8202, 80008, 3717, 84514, 23181, 9618, 4088, 339487, 7423 |
| 34 | C-type lectin receptor signaling pathway | 8615, 5599, 23365, 3551, 10892, 3659, 868, 9020, 602, 5971, 388403, 4773, 5291, 79830, 5594, 4775, 5781, 5602, 5336, 5290, 208, 4193, 5530, 5058, 84288, 3709, 207, 64800, 5296, 5603, 805, 6850, 5894, 9261, 6772, 5295, 4772 |
| 35 | Th1 and Th2 cell differentiation | 3460, 3714, 6776, 22889, 3932, 5599, 3551, 25992, 3566, 388403, 4773, 182, 84441, 5594, 4775, 55225, 55534, 5602, 3595, 5530, 919, 3594, 4794, 6775, 205428, 6777, 5603, 4793, 54980, 125950, 6772, 3717, 4772, 84514 |
| 36 | Renal cell carcinoma | 6655, 7040, 2889, 388403, 6654, 5291, 3091, 5594, 5781, 2033, 5604, 1387, 5290, 208, 5058, 2113, 207, 2034, 5296, 4233, 7428, 8453, 5906, 5894, 9978, 5295 |
| 37 | Endometrial cancer | 8312, 6655, 1495, 388403, 6654, 5291, 5594, 999, 4292, 5604, 2064, 83941, 5290, 208, 79613, 2309, 207, 6934, 1956, 842, 5296, 5894, 5728, 5295 |
| 38 | Human T-cell leukemia virus 1 infection | 3683, 1739, 7514, 472, 2005, 6776, 7417, 3932, 115, 5599, 3551, 4087, 9020, 8379, 83660, 7040, 6929, 5971, 6416, 388403, 4214, 4773, 113, 5291, 5423, 111, 5594, 4775, 2033, 5604, 1387, 5602, 7538, 94097, 64764, 90993, 821, 5290, 208, 29882, 5530, 8295, 119559, 996, 7419, 11200, 896, 2113, 207, 598, 6777, 2217, 5296, 200186, 9586, 291, 8881, 5728, 5295, 109, 4055, 4772, 84514, 4088 |
| 39 | Colorectal cancer | 26060, 8312, 6655, 596, 5599, 4087, 7040, 388403, 6654, 5291, 5594, 4292, 5604, 5900, 6198, 5602, 83941, 5290, 208, 266747, 207, 5899, 6934, 1630, 1956, 842, 5296, 5894, 5295, 4088 |
| 40 | Fc epsilon RI signaling pathway | 6655, 5599, 7410, 6416, 388403, 5578, 6654, 5291, 5594, 5604, 5602, 5336, 5290, 208, 695, 207, 10451, 5296, 5603, 6850, 4067, 5894, 51015, 5295, 9846 |
| 41 | Platinum drug resistance | 57507, 472, 2948, 2946, 2944, 596, 4217, 388403, 5980, 5291, 23060, 5594, 4292, 2064, 5290, 208, 4193, 207, 598, 842, 5296, 4258, 317, 1317, 5295 |
| 42 | MicroRNAs in cancer | 472, 1788, 6655, 596, 22889, 7057, 3551, 25992, 2744, 599, 1789, 6768, 23405, 5581, 5578, 659, 6654, 6093, 23414, 4363, 2033, 5604, 1387, 2064, 5579, 23411, 5156, 5336, 4194, 4193, 27250, 6774, 7168, 5159, 9252, 55691, 960, 57521, 2065, 205428, 1956, 25, 4233, 55714, 54980, 7430, 5894, 6541, 5728, 23150, 8434 |
| 43 | TNF signaling pathway | 8837, 3726, 5599, 3551, 3659, 9020, 2919, 602, 153090, 7186, 8717, 83737, 6416, 4217, 388403, 7188, 5291, 182, 5594, 5604, 5602, 64764, 90993, 5290, 208, 1326, 9252, 10906, 207, 5296, 843, 5603, 9586, 51015, 5295 |
| 44 | Cholinergic synapse | 9592, 2782, 2788, 596, 115, 2771, 817, 10681, 388403, 5578, 113, 5291, 111, 5594, 5604, 5579, 64764, 90993, 5290, 208, 3784, 775, 818, 59345, 3709, 207, 56479, 130872, 79140, 54331, 5332, 5296, 9586, 814, 5295, 109, 3717 |
| 45 | Progesterone-mediated oocyte maturation | 3480, 115, 5599, 2771, 8379, 388403, 113, 5291, 111, 891, 5594, 6197, 5140, 5604, 27330, 5602, 94097, 5290, 208, 29882, 6793, 119559, 996, 207, 5296, 5603, 3326, 6196, 5894, 8881, 5295, 109, 6790, 22849 |
| 46 | Phospholipase D signaling pathway | 8527, 1785, 51430, 6655, 1606, 57121, 115, 51195, 23396, 388403, 5578, 6654, 7249, 113, 5291, 111, 5594, 5781, 8394, 5604, 8526, 57186, 5900, 9267, 5156, 5336, 2917, 5290, 208, 9265, 5159, 266747, 3643, 207, 5899, 1902, 1956, 5332, 5296, 5337, 6850, 2185, 6009, 5894, 5295, 109, 375, 9846 |
| 47 | Autophagy - animal | 81671, 25963, 29063, 116064, 10533, 55626, 148534, 8837, 3480, 84901, 596, 51100, 8897, 4289, 5599, 125228, 79041, 9205, 64121, 23589, 54826, 55684, 388403, 55014, 7249, 129293, 7247, 440026, 57149, 5291, 3091, 9842, 5594, 55062, 55824, 23051, 5604, 57186, 221960, 57732, 6198, 440275, 55751, 23731, 5602, 149466, 5290, 208, 7879, 1613, 84928, 3916, 348110, 8076, 55007, 9711, 1509, 114826, 148362, 1508, 55206, 207, 9474, 64081, 598, 64419, 57798, 57521, 10645, 150372, 5296, 5289, 7405, 1612, 89849, 100129792, 6009, 5894, 140775, 5728, 5295, 23080, 26043, 54849, 219771, 23164, 80167, 9821, 221477 |
| 48 | Endocrine resistance | 6655, 3480, 3714, 596, 22889, 5747, 115, 5599, 25992, 388403, 6654, 113, 5291, 182, 111, 5594, 10498, 5604, 2064, 1565, 6198, 5602, 5290, 208, 4193, 5469, 55689, 207, 114786, 205428, 1956, 5296, 5603, 54980, 5894, 286046, 5295, 109, 8202 |
| 49 | FoxO signaling pathway | 472, 6655, 3480, 5599, 3551, 4087, 51701, 2308, 7040, 388403, 6654, 57149, 5291, 891, 5594, 51422, 2033, 5604, 1387, 23411, 5602, 5290, 208, 4193, 6774, 8743, 2309, 3643, 207, 1956, 5296, 5603, 5894, 5728, 5295, 5106, 100533105, 23678, 4088, 116986 |
| 50 | Acute myeloid leukemia | 6655, 3728, 6776, 3551, 7704, 861, 388403, 8864, 6654, 5291, 5594, 5604, 6198, 5290, 208, 6774, 207, 6934, 6777, 5296, 5894, 5295, 84514 |
| 51 | DNA replication | 5424, 91833, 4175, 3978, 54827, 55680, 79621, 6117, 5558, 10535, 79706, 22902, 120400, 4172, 5984, 1762 |
| 52 | Bacterial invasion of epithelial cells | 867, 1785, 51430, 10095, 23607, 2017, 5747, 9844, 1495, 10801, 3059, 79729, 1212, 79658, 10985, 23157, 5291, 1793, 999, 60685, 10109, 5290, 79613, 55752, 57609, 7414, 3688, 5296, 8976, 4233, 9564, 60, 1213, 5295, 71, 4735, 79641 |
| 53 | Tight junction | 1739, 5590, 117583, 8189, 4627, 79784, 91833, 1364, 4253, 2017, 55114, 5599, 7408, 55844, 81, 813, 2802, 3059, 4771, 27134, 9223, 861, 4217, 23327, 4214, 5581, 6093, 64091, 440193, 10096, 51422, 2064, 5521, 57530, 64097, 60685, 5602, 8495, 55227, 1080, 1741, 5937, 57669, 146712, 145282, 57407, 23288, 1740, 7074, 57609, 1366, 55691, 100506658, 3688, 3993, 87, 8976, 10207, 60, 57644, 7430, 9368, 5906, 123355, 9231, 51466, 23150, 71, 1762, 84952, 374618 |
| 54 | Proteoglycans in cancer | 125488, 867, 54462, 376940, 6655, 51196, 3480, 754, 2017, 57488, 7057, 5747, 23365, 817, 7257, 3549, 6548, 3059, 25938, 7040, 10985, 3339, 57587, 388403, 5578, 54847, 150864, 6654, 5291, 6093, 3091, 9842, 5594, 5500, 1634, 5781, 79591, 199990, 23051, 2260, 5604, 288, 2064, 50809, 5579, 8826, 6198, 5336, 54776, 5290, 208, 4193, 2318, 119504, 27250, 7023, 6774, 818, 145282, 6382, 6608, 5058, 7074, 80856, 8076, 5939, 162427, 91748, 3709, 207, 59339, 162073, 55691, 51316, 79647, 780776, 54477, 960, 3688, 65244, 2065, 79932, 1956, 5296, 4233, 199870, 60, 100129792, 5603, 55654, 2317, 7430, 6385, 5894, 3693, 196074, 23150, 5295, 71, 80008, 339487 |
| 55 | Adipocytokine signaling pathway | 1374, 5599, 3551, 7186, 8717, 32, 57149, 6256, 5465, 5781, 51422, 5602, 208, 6774, 51703, 4794, 207, 79602, 10645, 23305, 4793, 5106, 3717 |
| 56 | Lysosome | 2720, 4126, 6645, 535, 10239, 100526783, 8546, 1520, 1201, 1212, 4891, 285362, 203197, 79158, 8943, 114815, 1522, 256471, 50809, 9853, 23659, 10053, 164, 27297, 51172, 22937, 9179, 3916, 130340, 1509, 285237, 6272, 1508, 7805, 1497, 64081, 23184, 23431, 3423, 162, 138050, 26088, 23163, 1213, 91663, 427, 1075, 9516 |
| 57 | Hedgehog signaling pathway | 8452, 596, 3549, 57154, 408, 157, 51684, 156, 6608, 1453, 53944, 132884, 64399, 23291, 23335, 64750, 339745 |
| 58 | Apoptosis - fly | 6788, 472, 1998, 5599, 55288, 84823, 143, 4217, 388403, 254251, 57448, 5594, 9927, 5602, 9113, 55622, 1956, 2000, 317, 4013, 9618 |
| 59 | Lysine degradation | 84193, 6310, 4967, 58508, 8085, 9592, 55870, 9757, 27324, 79813, 5352, 29072, 79823, 84444, 55904, 84954, 83852, 107303344, 23731, 79723, 64324, 54904, 79180, 90007, 7468, 7799, 85007, 100506127, 5351, 84787, 2145, 79746, 26040, 217 |
| 60 | Choline metabolism in cancer | 8527, 6655, 1606, 5599, 23396, 388403, 5578, 6654, 7249, 5291, 3091, 5594, 56994, 8394, 5604, 8526, 57186, 5900, 5579, 6198, 60685, 5602, 5156, 5290, 208, 56261, 5159, 266747, 57609, 207, 1956, 5296, 8976, 5337, 6009, 5894, 5295, 5130 |
| 61 | N-Glycan biosynthesis | 4124, 4249, 201595, 2530, 23193, 11282, 199857, 6185, 79053, 29880, 8703, 10905, 57688, 4122, 4245, 57171, 84620, 11320, 79796 |
| 62 | Chronic myeloid leukemia | 867, 6655, 1488, 6776, 3551, 7040, 861, 1487, 388403, 6654, 5291, 5594, 5781, 5604, 83941, 5290, 208, 4193, 207, 598, 6777, 25, 5296, 5894, 5295, 84514, 9846 |
| 63 | cGMP-PKG signaling pathway | 5592, 7417, 115, 7408, 2771, 493, 4638, 488, 388403, 5581, 4773, 113, 4033, 57149, 6093, 111, 5594, 5500, 4775, 5140, 6546, 5604, 3778, 64764, 90993, 208, 775, 5530, 145282, 10242, 7419, 3643, 84288, 624, 3709, 207, 489, 84173, 64800, 5332, 9569, 805, 9586, 4209, 291, 5894, 100271849, 4207, 51466, 2977, 109, 4772 |
| 64 | Homologous recombination | 29086, 472, 5424, 51720, 10443, 84250, 79728, 11073, 4361, 6117, 580, 79706, 9577, 7517, 7156, 8940 |
| 65 | Human immunodeficiency virus 1 infection | 472, 9921, 2782, 2788, 596, 5747, 5599, 2771, 3551, 25939, 8065, 10681, 1642, 7186, 8717, 10985, 388403, 5578, 2923, 4773, 7188, 5291, 891, 5594, 4775, 5604, 5579, 6198, 5602, 5336, 115004, 10053, 164, 5290, 208, 3985, 5530, 60489, 919, 5058, 130340, 59345, 84288, 10513, 3709, 207, 4615, 598, 54331, 64800, 842, 2217, 5296, 162, 5603, 805, 2185, 5894, 9978, 51015, 5295, 23291, 23335, 4772, 8451 |
| 66 | T cell receptor signaling pathway | 1739, 6655, 3932, 3551, 10892, 868, 9020, 7410, 388403, 4773, 6654, 5291, 5594, 4775, 5604, 5290, 208, 1326, 5530, 919, 5058, 9402, 4794, 207, 10451, 5296, 5603, 4793, 5894, 5295, 4772 |
| 67 | Ras signaling pathway | 22821, 9771, 6655, 51196, 3480, 2782, 54819, 2788, 4763, 54453, 5599, 8315, 3551, 8437, 10681, 10235, 388403, 5578, 6654, 79890, 27, 5291, 5594, 5781, 2260, 5604, 5924, 5900, 5579, 5602, 5156, 285, 5336, 5290, 208, 84859, 55103, 5058, 7074, 5159, 266747, 59345, 147011, 3643, 84288, 4915, 2113, 8036, 207, 5899, 83593, 598, 5868, 100134938, 54331, 1956, 64800, 4804, 55770, 25, 5296, 4233, 5337, 805, 5906, 9462, 5894, 5295, 9846, 7423, 5920 |
| 68 | Dorso-ventral axis formation | 64319, 6655, 22889, 79370, 25992, 2120, 388403, 6654, 26010, 5594, 55084, 5604, 149473, 84310, 2113, 65244, 205428, 23518, 1956, 51513, 54980, 56776, 84501, 22849 |
| 69 | Glioma | 6655, 3480, 817, 388403, 5578, 6654, 5291, 5594, 5604, 5579, 83941, 5156, 5336, 5290, 208, 4193, 818, 5159, 84288, 207, 57118, 1956, 64800, 5296, 805, 5894, 5728, 814, 5295 |
| 70 | Circadian rhythm | 8864, 51422, 9572, 1407, 1453, 5187, 8863, 4862, 9978, 23291, 23335 |
| 71 | Type II diabetes mellitus | 5590, 9592, 5599, 3551, 388403, 5581, 57149, 5291, 5594, 5602, 3099, 5290, 775, 3643, 5296, 5295 |
| 72 | Kaposi sarcoma-associated herpesvirus infection | 8615, 2782, 2788, 5599, 3551, 10681, 2919, 7186, 8717, 6416, 388403, 4773, 5291, 3091, 5594, 4775, 2033, 55225, 5604, 1387, 5602, 7538, 285, 5336, 3455, 5290, 208, 6774, 5530, 59345, 3572, 84288, 10906, 3709, 207, 3454, 54331, 64800, 842, 2217, 5296, 5289, 5603, 805, 6850, 4067, 5894, 125950, 9261, 6772, 5295, 3717, 4772 |
| 73 | Chemokine signaling pathway | 5590, 117583, 2869, 6655, 2782, 2788, 5747, 115, 2771, 3551, 10681, 9844, 1794, 2919, 10235, 408, 7410, 10985, 388403, 6654, 113, 157, 5291, 6093, 111, 5594, 5604, 5579, 60685, 156, 5290, 208, 6774, 145282, 5058, 7074, 2309, 59345, 57609, 207, 54331, 6777, 5332, 10451, 5296, 8976, 9644, 9564, 4793, 4067, 5906, 2185, 5894, 6772, 5295, 109, 3717, 84514 |
| 74 | Epstein-Barr virus infection | 3683, 4940, 596, 5599, 3551, 9020, 54826, 7186, 8717, 5971, 6416, 25942, 2923, 7188, 5291, 55225, 5602, 83941, 5336, 3455, 5290, 208, 4193, 6774, 919, 896, 695, 10906, 4794, 207, 23184, 9612, 4615, 960, 3454, 842, 2217, 5296, 22938, 9636, 5603, 4793, 317, 6850, 4067, 953, 57506, 51015, 125950, 6772, 5295, 9778, 5702, 5710 |
| 75 | Valine, leucine and isoleucine degradation | 54497, 34, 18, 197322, 25938, 3032, 11112, 56922, 5095, 84747, 3157, 84656, 3712, 80127, 3155, 79746, 217, 30 |
| 76 | Hedgehog signaling pathway - fly | 8452, 2869, 57154, 51684, 6608, 53944, 9978, 23291, 23335, 64750, 339745 |
| 77 | Peroxisome | 55297, 5190, 84188, 60526, 79830, 10005, 23417, 3418, 9409, 4843, 55670, 51, 6342, 23214, 4358, 51703, 373156, 54677, 5189, 254295, 7498, 23305, 225, 3155, 5825, 5195, 30 |
| 78 | Viral carcinogenesis | 1739, 7337, 79885, 8615, 6776, 1108, 81, 8379, 51564, 1642, 7186, 8717, 10985, 388403, 7531, 7188, 9734, 3431, 5291, 5423, 5594, 2033, 1387, 2934, 64764, 90993, 5290, 55227, 4193, 6774, 10014, 7419, 3572, 896, 10906, 27044, 9093, 6777, 8365, 87, 2217, 5296, 22938, 85477, 6850, 9586, 23352, 4067, 123355, 93349, 9261, 5295, 4055, 84514, 3020, 55869 |
| 79 | Hippo signaling pathway - fly | 6788, 1739, 117583, 55233, 5599, 55844, 4026, 126308, 473, 51114, 4771, 7531, 440193, 5521, 167410, 5602, 9113, 128077, 55227, 146691, 83937, 55691, 79683, 55841, 3993, 7003, 60, 123355, 71 |
| 80 | Notch signaling pathway | 5663, 1488, 3714, 22889, 25992, 1855, 54826, 1487, 182, 84441, 2033, 1387, 151636, 55534, 9612, 205428, 22938, 54980, 8650, 9778 |
| 81 | Nucleotide excision repair | 57507, 5424, 3978, 2074, 1642, 54827, 23060, 6117, 2068, 54841, 100533467, 5886, 79706, 120400, 9978, 5984, 5887, 8451 |
| 82 | VEGF signaling pathway | 8615, 5747, 10985, 388403, 5578, 4773, 5291, 5594, 5604, 5579, 5336, 5290, 208, 5530, 207, 842, 5296, 5603, 5894, 9261, 5295 |
| 83 | Prolactin signaling pathway | 8792, 6655, 6776, 5599, 3659, 388403, 6654, 5291, 5594, 5604, 5602, 5290, 208, 6774, 2309, 207, 6777, 5296, 5603, 5894, 6772, 5295, 3717, 84514 |
| 84 | Shigellosis | 10095, 2017, 5599, 3551, 9844, 3059, 388403, 1729, 6093, 1793, 5594, 57459, 60685, 5602, 10109, 145282, 57609, 9474, 7414, 960, 3688, 25, 8976, 60, 5603, 8233, 4793, 80206, 23291, 71, 23335 |
| 85 | Longevity regulating pathway - worm | 55297, 84188, 23314, 5599, 4780, 143, 5291, 3091, 256536, 221960, 23411, 6198, 5602, 5290, 208, 2309, 3643, 207, 83786, 3054, 7428, 5603, 889, 4013, 5728 |
| 86 | RNA degradation | 23019, 51013, 54462, 57583, 148534, 4848, 84901, 22803, 10766, 10950, 57488, 9528, 246175, 100534012, 79005, 51768, 7257, 9205, 5073, 64282, 4850, 9980, 57587, 5394, 203197, 255967, 26010, 79830, 100505549, 55824, 10200, 5214, 55802, 199990, 8496, 167227, 25904, 374354, 139322, 55751, 83941, 54952, 57472, 5211, 55007, 85379, 29883, 26036, 113201, 26502, 87178, 151525, 91748, 54834, 254295, 23184, 79647, 153443, 79932, 124491, 100134938, 694, 201931, 1656, 91663, 80153, 219771, 79664, 339487, 51691 |
| 87 | Apelin signaling pathway | 2782, 2788, 115, 2771, 4087, 10681, 4638, 6548, 388403, 5581, 113, 182, 111, 5594, 999, 5140, 51422, 6546, 5604, 4843, 6198, 208, 6263, 79613, 10014, 59345, 84288, 3709, 207, 54331, 64800, 5332, 5289, 805, 4209, 5894, 100271849, 4207, 814, 109, 4088 |
| 88 | Glycosaminoglycan biosynthesis - keratan sulfate | 2530, 4166, 23563, 93010, 8703, 8702 |
| 89 | Toll and Imd signaling pathway | 5599, 3551, 7322, 288, 11124, 7323, 5602, 152007, 4615, 4216, 5603, 51015, 7336, 23291, 23335, 339487 |
| 90 | Toxoplasmosis | 3949, 3460, 596, 5599, 2771, 3551, 22982, 25938, 7040, 388403, 129293, 100505549, 5594, 55225, 4843, 51379, 5602, 11333, 208, 6774, 84928, 148362, 57609, 113201, 207, 4615, 598, 26033, 3688, 3915, 100134938, 842, 5603, 159195, 4793, 51015, 140775, 125950, 6772, 3717, 23181 |
| 91 | Transcriptional misregulation in cancer | 153768, 472, 2005, 2530, 64919, 57583, 5316, 3480, 3728, 5079, 57488, 9528, 79370, 5747, 81669, 5468, 3487, 2120, 9980, 64332, 2308, 7704, 6929, 8079, 861, 25942, 8864, 150864, 26010, 286205, 6256, 3429, 84444, 221037, 3248, 6760, 374354, 84954, 83941, 4193, 23241, 5218, 6692, 8842, 80856, 7690, 10653, 85379, 26036, 9204, 2130, 147011, 7468, 55589, 91748, 4299, 79647, 598, 65244, 92140, 79932, 4804, 51513, 4233, 55654, 8351, 8148, 84918, 2115, 151556, 7113, 2537, 26043, 3020, 5087 |
| 92 | ABC transporters | 23460, 10257, 368, 23461, 4363, 10057, 1080, 225, 21, 10349, 22, 10351, 8714, 5825 |
| 93 | Long-term potentiation | 817, 388403, 5578, 5594, 6197, 5500, 2033, 5604, 1387, 5579, 27330, 775, 818, 5530, 84288, 3709, 64800, 5332, 805, 6196, 5906, 5894, 814 |
| 94 | SNARE interactions in vesicular transport | 9341, 55014, 9527, 10228, 6804, 143187, 6811, 6810, 6844, 6809, 55850 |
| 95 | Focal adhesion | 3679, 6655, 3480, 170394, 754, 596, 7057, 5747, 5599, 7408, 81, 22982, 4638, 148223, 51608, 83660, 7410, 25938, 7791, 2909, 2889, 10985, 388403, 5578, 54847, 6654, 129293, 1729, 5291, 6093, 8516, 1793, 100505549, 5594, 5500, 3675, 23635, 5604, 2064, 57459, 221749, 5579, 51379, 5602, 5156, 54776, 11333, 1287, 5290, 208, 2318, 55103, 3694, 84928, 824, 145282, 57407, 5058, 5159, 283310, 55742, 148362, 57609, 113201, 896, 207, 162073, 54751, 7414, 9473, 26033, 3688, 54991, 3915, 100134938, 1956, 87, 10451, 5296, 4233, 147650, 9564, 60, 1278, 159195, 2317, 55714, 5906, 5894, 80206, 3693, 51466, 140775, 5728, 5295, 71, 3672, 56654, 23181, 7423 |
| 96 | Fatty acid biosynthesis | 2194, 32, 284001, 51703, 31, 23305 |
| 97 | Melanoma | 3480, 388403, 5291, 5594, 999, 2260, 5604, 83941, 5156, 5290, 208, 4193, 79613, 5159, 207, 1956, 5296, 4233, 5894, 5728, 5295 |
| 98 | Axon guidance | 2050, 5590, 117583, 5163, 5362, 85464, 2048, 5747, 2771, 23365, 817, 54961, 23654, 55684, 10509, 388403, 5578, 659, 4773, 5291, 6093, 5594, 4775, 5781, 23074, 55740, 5336, 10512, 5290, 3985, 6259, 818, 5530, 145282, 57407, 6608, 5058, 10154, 54434, 9204, 655, 2043, 57715, 3688, 1630, 25, 5296, 4233, 1948, 1808, 57522, 5894, 5998, 22885, 5295 |
| 99 | Caffeine metabolism | 60526, 79830, 7498 |
| 100 | Longevity regulating pathway - multiple species | 3480, 115, 2308, 113, 57149, 5291, 111, 51422, 23411, 6198, 5290, 208, 2309, 3643, 207, 9474, 57521, 5296, 5295, 109 |
| 101 | Glucagon signaling pathway | 1374, 5257, 817, 2308, 32, 5260, 5465, 5140, 51422, 2033, 1387, 23411, 64764, 90993, 208, 55293, 818, 5530, 5162, 31, 84288, 2997, 3709, 207, 64800, 5332, 200186, 805, 9586, 5106 |
| 102 | GnRH signaling pathway | 6655, 115, 5599, 817, 6416, 388403, 4214, 5578, 6654, 113, 111, 10746, 5594, 5604, 5579, 5602, 775, 818, 84288, 3709, 1956, 64800, 5332, 5337, 4216, 5603, 805, 2185, 5894, 51015, 109 |
| 103 | Natural killer cell mediated cytotoxicity | 3683, 3460, 6655, 3932, 7410, 388403, 5578, 4773, 6654, 5291, 5594, 5781, 5604, 5579, 5336, 3455, 5290, 5530, 6452, 919, 5058, 8743, 3454, 10451, 22914, 100528032, 5296, 6850, 2185, 5894, 5295, 4772 |
| 104 | Cutin, suberine and wax biosynthesis | 55297, 84188 |
| 105 | Chagas disease (American trypanosomiasis) | 3460, 8837, 5599, 2771, 3551, 4087, 55844, 7040, 6416, 388403, 5291, 5594, 5521, 4843, 5602, 5290, 208, 919, 624, 207, 4615, 5332, 5296, 5603, 5295, 4088 |
| 106 | Small cell lung cancer | 596, 5747, 3551, 22982, 25938, 7186, 7188, 129293, 5291, 100505549, 6256, 3675, 23635, 2272, 4843, 51379, 83941, 11333, 1287, 5290, 208, 84928, 7690, 148362, 57609, 113201, 10906, 207, 598, 26033, 3688, 3915, 100134938, 842, 5296, 159195, 317, 140775, 5728, 5295, 23181, 9618 |
| 107 | Circadian entrainment | 5592, 2782, 2788, 9722, 115, 2771, 817, 10681, 3765, 388403, 5578, 8864, 113, 111, 5594, 5579, 6263, 775, 818, 9252, 59345, 84288, 5187, 8863, 54331, 64800, 5332, 805, 2977, 109 |
| 108 | Regulation of lipolysis in adipocytes | 5592, 115, 2771, 11343, 113, 57149, 5291, 111, 5140, 133308, 5290, 208, 3643, 207, 5296, 57104, 5295, 109, 5920 |
| 109 | MAPK signaling pathway | 6788, 2005, 5495, 8491, 8615, 6655, 3480, 9592, 754, 4763, 5599, 3551, 51701, 9020, 10235, 408, 7186, 8717, 7040, 5971, 9175, 6416, 4217, 388403, 4214, 5578, 6654, 10746, 5594, 6197, 4775, 51347, 2260, 5604, 2064, 5924, 8550, 5579, 27330, 5602, 5156, 285, 208, 2318, 55103, 775, 1326, 5530, 5058, 5159, 9252, 3643, 4915, 51295, 207, 4615, 83786, 2065, 57551, 1956, 4804, 4233, 1850, 5536, 2872, 4216, 5603, 2317, 6196, 5906, 5894, 64746, 51015, 9261, 23335, 3164, 4772, 7423 |
| 110 | Human cytomegalovirus infection | 6655, 2782, 2788, 5747, 115, 2771, 23365, 3551, 10681, 7186, 8717, 10985, 388403, 5578, 2923, 4773, 6654, 7249, 7188, 113, 5291, 6093, 111, 5594, 4775, 5604, 57186, 5579, 6198, 5156, 115004, 64764, 90993, 5290, 208, 4193, 6774, 5530, 145282, 11214, 59345, 84288, 3709, 207, 54331, 1956, 64800, 842, 5332, 2217, 5296, 9564, 5603, 805, 9586, 2185, 6009, 5894, 5295, 109, 4772 |
| 111 | Bladder cancer | 7057, 388403, 5594, 999, 5604, 2064, 4193, 1613, 79613, 9252, 1956, 1612, 5894 |
| 112 | Fatty acid degradation | 124, 125, 126, 1374, 34, 3032, 51, 1632, 84747, 51703, 23305, 79746, 217, 30 |
| 113 | Calcium signaling pathway | 5257, 3269, 90550, 51196, 9592, 3360, 7417, 115, 817, 493, 113026, 4638, 488, 80271, 5578, 54847, 113, 5260, 79830, 5025, 6546, 3707, 2064, 5579, 4843, 5156, 5336, 6263, 775, 818, 5530, 5159, 7419, 57620, 84288, 624, 3709, 489, 130872, 79140, 2065, 57118, 135, 1956, 64800, 5332, 805, 2185, 291, 814, 109, 6786, 6870 |
| 114 | Hepatitis C | 3949, 8837, 6655, 1364, 4940, 3551, 55844, 7186, 8717, 388403, 7531, 6654, 5291, 5594, 6256, 5465, 55225, 129531, 5604, 5521, 440275, 3455, 5290, 208, 6774, 10906, 1366, 207, 100506658, 3454, 1956, 842, 5296, 317, 5894, 57506, 125950, 6772, 5295 |
| 115 | Ubiquinone and other terpenoid-quinone biosynthesis | 79001, 84680, 1728, 10229, 2677 |
| 116 | p53 signaling pathway | 472, 8493, 596, 7057, 64326, 3487, 7249, 891, 57186, 4194, 23143, 4193, 50484, 4034, 11200, 896, 57470, 598, 842, 375190, 199870, 317, 5728, 25898 |
| 117 | TGF-beta signaling pathway | 92, 7057, 51232, 4087, 57154, 4052, 7040, 388403, 659, 6093, 5594, 100527943, 1634, 2033, 1387, 91, 6198, 655, 7027, 4090, 657, 9978, 64750, 4088 |
| 118 | mTOR signaling pathway | 4040, 125488, 54462, 376940, 6655, 3480, 4041, 57488, 253260, 3551, 6520, 7257, 1855, 64121, 6249, 25938, 9681, 57587, 51719, 388403, 5578, 150864, 6654, 7249, 91355, 5291, 9842, 5594, 6197, 79591, 199990, 23051, 79899, 5604, 57186, 221960, 50809, 5579, 27330, 6198, 153129, 5290, 208, 119504, 529, 80856, 8076, 5939, 3643, 162427, 91748, 55615, 207, 59339, 162073, 51316, 79647, 780776, 54477, 65244, 57521, 79932, 5296, 57600, 8131, 199870, 80318, 100129792, 55654, 79726, 6196, 6009, 5894, 196074, 317649, 5728, 5295, 80008, 2887, 339487 |
| 119 | Propanoate metabolism | 34, 18, 8801, 32, 23417, 5095, 55293, 31, 23255, 80127, 79746 |
| 120 | Fatty acid metabolism | 1374, 34, 2194, 7871, 3032, 9200, 51, 6342, 84747, 284001, 51703, 31, 23305, 79746, 1528, 30 |
| 121 | Aldosterone-regulated sodium reabsorption | 23327, 388403, 5578, 5291, 5594, 5579, 5290, 3643, 6340, 5296, 5295, 4306 |
| 122 | Influenza A | 7514, 3460, 3841, 4940, 5599, 3551, 150709, 5045, 100529063, 4928, 6416, 388403, 5578, 7706, 5291, 79830, 5594, 2033, 55225, 5604, 3839, 1387, 440275, 5602, 3455, 5290, 208, 10482, 8743, 3836, 23633, 207, 4615, 3454, 842, 5296, 10625, 60, 5603, 4793, 64135, 5894, 57506, 51015, 125950, 6772, 56649, 7113, 5295, 71, 3717, 3268 |
| 123 | Fluid shear stress and atherosclerosis | 5590, 92, 8878, 2948, 2946, 2944, 754, 596, 5747, 5599, 3551, 4780, 445, 51588, 6416, 4217, 659, 5291, 5602, 5290, 208, 9352, 6382, 9817, 84288, 207, 64800, 5296, 9644, 60, 657, 5603, 3326, 1728, 4258, 805, 6385, 5295, 71 |
| 124 | Mismatch repair | 5424, 3978, 113146, 79026, 4292, 6117, 79706, 5984 |
| 125 | Morphine addiction | 5151, 112476, 2869, 9592, 2782, 2788, 115, 2771, 10681, 3765, 408, 5578, 113, 157, 111, 5140, 5579, 156, 4302, 59345, 54331, 155435, 5143, 8028, 5144, 109, 2570 |
| 126 | Oocyte meiosis | 3480, 8243, 115, 817, 8379, 388403, 7531, 113, 111, 5527, 891, 5594, 6197, 5500, 91050, 5604, 27330, 94097, 29882, 818, 5530, 119559, 996, 84288, 3709, 64800, 5525, 805, 6196, 9978, 8881, 109, 23291, 6790, 23335, 22849 |
| 127 | Cell cycle | 472, 5000, 10274, 8243, 4175, 4087, 8379, 7040, 7531, 891, 8556, 2033, 5001, 1387, 94097, 4193, 29882, 5591, 4999, 119559, 7690, 996, 7029, 11200, 896, 7027, 11188, 25, 4172, 9978, 8881, 4088 |
| 128 | NF-kappa B signaling pathway | 8792, 472, 8837, 596, 3932, 3551, 10892, 9020, 51588, 7186, 8717, 5971, 7188, 7706, 5579, 5336, 23143, 114609, 4034, 695, 10906, 57470, 4615, 598, 6850, 4067, 4055 |
| 129 | NOD-like receptor signaling pathway | 90550, 4940, 596, 7417, 55914, 5599, 3551, 813, 54822, 2919, 7186, 388403, 140609, 7188, 79830, 7158, 5594, 10010, 55225, 22900, 3428, 9927, 5602, 3455, 55669, 9352, 7419, 10628, 1508, 10906, 3709, 9474, 4615, 2635, 598, 3454, 5332, 5603, 3326, 4793, 55072, 92714, 57506, 125950, 6772, 1536 |
| 130 | Phosphonate and phosphinate metabolism | 56994, 85465, 5130 |
| 131 | Retrograde endocannabinoid signaling | 112476, 9592, 2782, 2788, 4719, 115, 5599, 2771, 10681, 3765, 22982, 11343, 388403, 5578, 4708, 113, 111, 5594, 5579, 4724, 5602, 775, 4302, 59345, 221955, 3709, 54331, 5332, 51079, 5603, 155435, 2166, 8028, 109, 2570, 4705, 4726 |
| 132 | Dopaminergic synapse | 9592, 2782, 2788, 5599, 2771, 817, 10681, 55844, 3765, 5578, 111, 5527, 5500, 91050, 5521, 5579, 5602, 64764, 90993, 208, 775, 818, 5530, 55012, 59345, 84288, 3709, 207, 54331, 64800, 5332, 5525, 5603, 805, 9586, 3799 |
| 133 | Fructose and mannose metabolism | 2762, 5373, 5372, 197258, 5214, 3099, 55556, 5211, 5208, 5210 |
| 134 | cAMP signaling pathway | 10257, 22990, 51196, 3360, 115, 5599, 2771, 817, 51195, 84067, 493, 7410, 6548, 388403, 113, 5291, 6093, 111, 5594, 5500, 5465, 5140, 2033, 5604, 1387, 51, 5602, 64764, 90993, 5290, 208, 1080, 775, 818, 145282, 5058, 7074, 84288, 207, 79140, 64399, 135, 64800, 10451, 5296, 5337, 5143, 805, 9586, 5906, 5144, 5894, 814, 5295, 109, 4772 |
| 135 | Pyruvate metabolism | 9380, 98, 3029, 32, 1737, 10873, 55293, 5162, 31, 23255, 4199, 5106, 217 |
| 136 | Ferroptosis | 64116, 10533, 7417, 6520, 4891, 2730, 139322, 7037, 51703, 7419, 9474, 23305, 1536 |
| 137 | Gap junction | 6655, 5592, 115, 2771, 388403, 5578, 6654, 113, 111, 10746, 5594, 5604, 5579, 5156, 146712, 1453, 5159, 3709, 1902, 1956, 5332, 5894, 9231, 2977, 109 |
| 138 | Vasopressin-regulated water reabsorption | 115, 1783, 51626, 64764, 90993, 6810, 6844, 1778, 5868, 9586, 51143, 109 |
| 139 | Fanconi anemia pathway | 11201, 10443, 5980, 4292, 2175, 79728, 6117, 83941, 51455, 55120, 2177, 22909, 201254, 7156, 8940 |
| 140 | Glycerophospholipid metabolism | 8527, 23646, 23175, 1606, 6901, 253558, 9489, 8760, 129642, 56994, 8526, 85465, 23659, 56261, 23171, 10423, 375775, 147011, 55500, 9663, 23761, 5337, 151056, 5130, 56654, 5920 |
| 141 | Vitamin B6 metabolism | 54497, 8566, 25938 |
| 142 | Apoptosis - multiple species | 596, 5599, 57448, 5602, 55622, 598, 842, 4804, 317 |
| 143 | Thermogenesis | 1374, 8578, 6655, 5592, 57492, 4719, 115, 5468, 7385, 9377, 11343, 4217, 4708, 6654, 7249, 539, 113, 23028, 111, 6197, 6602, 6599, 51422, 2260, 57186, 133308, 4724, 27330, 6198, 6598, 64764, 90993, 51780, 51703, 10476, 1345, 11180, 137682, 1953, 10975, 57688, 57521, 51079, 57104, 23305, 60, 5603, 6196, 9586, 6009, 9658, 51015, 513, 109, 71, 4705, 4726 |
| 144 | Parathyroid hormone synthesis, secretion and action | 4040, 4041, 596, 115, 2771, 408, 388403, 5578, 7421, 91355, 113, 111, 5594, 6256, 2260, 5604, 5579, 64764, 90993, 11214, 3709, 10893, 1956, 5332, 5337, 5143, 9368, 9586, 4209, 5144, 5894, 109, 85456 |
| 145 | Adrenergic signaling in cardiomyocytes | 596, 115, 2771, 817, 51195, 55844, 493, 6548, 388403, 5578, 113, 111, 5527, 5594, 5500, 91050, 5521, 132320, 64764, 90993, 208, 3784, 775, 818, 7168, 55012, 7171, 9252, 84288, 207, 7170, 64800, 5332, 5525, 5603, 805, 9586, 109, 23335 |
| 146 | Necroptosis | 3460, 128866, 8837, 8878, 6776, 596, 124044, 7417, 6645, 5599, 817, 54822, 7186, 8717, 143, 7188, 79830, 55225, 129531, 11124, 5602, 3455, 6774, 818, 824, 8743, 823, 7419, 3454, 6775, 6777, 100996485, 3326, 55072, 9555, 4013, 25978, 291, 125950, 6772, 3717, 84514, 1536 |
| 147 | Measles | 3460, 4940, 6776, 868, 5291, 55225, 440275, 3455, 5290, 208, 8667, 6774, 8743, 896, 207, 55691, 4615, 3454, 6777, 5296, 10399, 4793, 64135, 57506, 125950, 6772, 23150, 5295, 25898, 3717, 84514 |
| 148 | Amino sugar and nucleotide sugar metabolism | 7360, 2673, 2762, 5373, 606495, 5372, 197258, 80146, 10020, 3099, 54187, 55577, 30816, 51005 |
| 149 | Hippo signaling pathway - multiple species | 6788, 55233, 126308, 4771, 9113, 5058, 83937, 55691, 55841, 7003 |
| 150 | Pancreatic secretion | 115, 22802, 6558, 493, 6548, 488, 5578, 113, 111, 9545, 8671, 1811, 5579, 3778, 3784, 1080, 147011, 3475, 3709, 489, 130872, 30816, 5332, 5906, 109 |
| 151 | Tuberculosis | 3460, 596, 8411, 6645, 535, 5599, 23365, 817, 1520, 10892, 3687, 8717, 7040, 388403, 7421, 203197, 7247, 5594, 2033, 1387, 50809, 4843, 5602, 27297, 208, 7879, 818, 5530, 3916, 114609, 1509, 84288, 207, 64081, 23184, 4615, 5868, 64800, 842, 5289, 843, 5603, 805, 317, 6850, 5894, 972, 6772, 91663, 3717 |
| 152 | Mitophagy - animal | 25963, 29063, 116064, 55626, 148534, 8878, 84901, 4289, 5599, 125228, 79041, 55288, 9205, 23589, 54826, 129293, 7247, 440026, 3091, 9101, 55824, 84749, 23051, 4077, 55751, 9927, 23731, 5602, 8887, 55669, 149466, 7879, 84928, 348110, 8076, 55007, 2309, 114826, 148362, 55206, 9474, 64081, 139341, 598, 57798, 150372, 100129792, 140775, 23786, 23080, 26043, 219771, 23164, 80167, 221477, 665 |
| 153 | Mannose type O-glycan biosynthesis | 729920, 10585, 9215, 1038, 131177, 152002, 8703, 148789 |
| 154 | Inflammatory mediator regulation of TRP channels | 3269, 115, 5599, 817, 5581, 5578, 113, 5291, 111, 5500, 5579, 5602, 5336, 5290, 818, 5029, 84288, 624, 3709, 64800, 5332, 5296, 5603, 805, 51015, 5295, 109 |
| 155 | Epithelial cell signaling in Helicobacter pylori infection | 535, 5599, 3551, 9020, 6416, 5781, 5602, 5336, 529, 5058, 1956, 4233, 5603, 102, 4067, 9231 |
| 156 | Glycerolipid metabolism | 8527, 346606, 23175, 1606, 253558, 129642, 10327, 11343, 8526, 133308, 158219, 2710, 9663, 22996, 57104, 217 |
| 157 | Vibrio cholerae infection | 535, 115, 6558, 11014, 5578, 5336, 3784, 1080, 529, 30001, 60, 9231, 109, 71, 375 |
| 158 | Hepatocellular carcinoma | 4040, 125488, 54462, 8312, 376940, 6655, 3480, 2948, 2946, 2944, 4041, 57488, 57492, 4087, 7257, 4780, 1855, 25938, 55193, 7040, 57587, 388403, 5578, 150864, 6654, 91355, 5291, 9842, 5594, 6602, 6599, 79591, 199990, 23051, 5604, 50809, 5579, 6198, 6598, 83941, 5336, 5290, 208, 119504, 9817, 80856, 8076, 5939, 162427, 91748, 207, 6934, 59339, 162073, 51316, 79647, 780776, 54477, 57688, 598, 65244, 79932, 1956, 5296, 4233, 199870, 60, 100129792, 55654, 1728, 4258, 5894, 196074, 5728, 5295, 71, 196528, 80008, 4088, 339487 |
| 159 | Oxytocin signaling pathway | 115, 2771, 817, 3765, 4638, 29904, 388403, 5578, 4773, 113, 6093, 111, 5594, 5500, 4775, 51422, 5604, 5579, 54776, 6263, 775, 818, 5530, 145282, 84288, 3709, 57118, 10645, 1956, 64800, 5332, 1938, 60, 805, 5894, 2977, 814, 109, 71, 23335, 4772 |
| 160 | Signaling pathways regulating pluripotency of stem cells | 125488, 54462, 92, 3977, 9314, 8312, 5316, 376940, 3480, 57488, 4087, 7257, 1855, 25938, 6497, 6929, 57587, 388403, 659, 150864, 5291, 9842, 5594, 463, 79591, 199990, 23051, 2260, 5604, 50809, 91, 27291, 284106, 7703, 5290, 208, 119504, 6774, 80856, 8076, 3572, 5939, 55571, 162427, 91748, 207, 55183, 59339, 162073, 51316, 79647, 780776, 54477, 65244, 79932, 7994, 5296, 4090, 199870, 100129792, 657, 5603, 55654, 5894, 196074, 5295, 80008, 3717, 3720, 4088, 339487 |
| 161 | beta-Alanine metabolism | 34, 1806, 18, 55748, 23417, 80127, 79746, 217 |
| 162 | Insulin secretion | 54795, 170394, 22889, 115, 817, 51195, 5578, 113, 111, 3781, 5579, 3778, 64764, 6804, 90993, 775, 818, 10242, 6844, 130872, 5332, 9586, 109, 9847 |
| 163 | Phototransduction - fly | 817, 55684, 5578, 157, 156, 818, 221955, 84288, 64800, 5332, 60, 805, 71 |
| 164 | Gastric cancer | 4040, 125488, 54462, 100288142, 400818, 8312, 376940, 6655, 3728, 57224, 4041, 4583, 596, 57488, 4087, 7257, 1855, 100132406, 199953, 1495, 25938, 7040, 29998, 57587, 388403, 150864, 6654, 91355, 5291, 9842, 5594, 6256, 999, 4292, 79591, 23013, 199990, 51599, 23051, 5604, 2064, 50809, 79098, 6311, 6198, 58986, 83941, 79567, 169611, 1015, 5290, 208, 119504, 92736, 55672, 23241, 79613, 54897, 80856, 8076, 4302, 5939, 23145, 162427, 91748, 207, 6934, 59339, 162073, 25849, 26053, 51316, 79647, 780776, 54477, 9672, 65244, 79932, 100131755, 1956, 57605, 5296, 4233, 114823, 199870, 100129792, 55654, 5894, 140688, 196074, 5295, 23199, 80008, 25829, 4088, 339487 |
| 165 | Salmonella infection | 3460, 10095, 754, 5599, 1783, 2919, 388403, 5585, 6093, 5594, 4843, 60685, 5602, 10109, 3831, 7879, 2318, 145282, 23207, 57609, 4615, 1778, 8976, 60, 5603, 2317, 9231, 51143, 71 |
| 166 | Base excision repair | 5424, 91833, 3978, 54827, 143, 5423, 79706, 120400, 8930, 4013, 1762 |
| 167 | GABAergic synapse | 112476, 9592, 66008, 81539, 2782, 2788, 18, 115, 2771, 10681, 2744, 5578, 113, 111, 5579, 84816, 775, 4302, 59345, 54331, 155435, 8028, 109, 2570 |
| 168 | PI3K-Akt signaling pathway | 3679, 6655, 3480, 170394, 2782, 2788, 596, 57121, 7057, 5747, 3551, 10681, 55844, 22982, 148223, 51608, 25938, 3566, 9223, 388403, 7531, 5578, 5585, 54847, 6654, 7249, 129293, 5291, 8516, 5527, 100505549, 5594, 6256, 91050, 3675, 23635, 2260, 5604, 57186, 2064, 5521, 221749, 51379, 6198, 5156, 285, 3455, 64764, 90993, 11333, 1287, 5290, 208, 4193, 3694, 84928, 5159, 283310, 55012, 2309, 59345, 148362, 57609, 113201, 896, 5008, 3643, 4915, 2997, 207, 162073, 9473, 1902, 598, 26033, 3688, 79140, 57521, 54991, 2065, 3454, 3915, 100134938, 54331, 1956, 842, 4804, 5525, 5296, 4233, 147650, 200186, 1278, 117145, 3326, 159195, 55714, 6850, 9586, 6009, 5894, 3693, 317649, 140775, 5728, 5295, 5106, 3672, 3717, 3164, 100533105, 23678, 56654, 23181, 7423 |
| 169 | Protein export | 11231, 23478, 6729, 55830, 5018, 6730 |
| 170 | Monobactam biosynthesis | 9061 |
| 171 | Gastric acid secretion | 115, 2771, 817, 4638, 6548, 5578, 113, 111, 5579, 3784, 1080, 818, 84288, 3475, 3709, 115111, 130872, 64800, 5332, 7430, 805, 109 |
| 172 | Carbohydrate digestion and absorption | 6476, 5291, 5579, 3099, 5290, 208, 207, 93432, 5296, 5295 |
| 173 | Vitamin digestion and absorption | 4363, 55788, 10560, 8884, 51374, 151056, 25974 |
| 174 | Pyrimidine metabolism | 22978, 8615, 1806, 1635, 51727, 957, 318, 84450, 50484, 1503, 955, 7371, 7298, 953 |
| 175 | mRNA surveillance pathway | 23008, 8189, 81608, 91833, 285527, 79811, 10914, 55844, 196441, 100529063, 26019, 55339, 54815, 79830, 5527, 5500, 91050, 64895, 5521, 221749, 8732, 54952, 54676, 54823, 10482, 23293, 55012, 23381, 5976, 2935, 114786, 5411, 5525, 22794, 22985, 1762 |
| 176 | Galactose metabolism | 7360, 2720, 2595, 6476, 5214, 3099, 5211, 130589, 93432 |
| 177 | Nicotinate and nicotinamide metabolism | 22978, 54497, 64802, 23530, 25938, 64788, 133686, 93100, 65220 |
| 178 | Alzheimer disease | 8883, 57142, 5663, 4719, 25825, 7385, 488, 6653, 9377, 388403, 4708, 22926, 539, 5594, 4724, 6263, 775, 131177, 5530, 824, 823, 10476, 1345, 84288, 3709, 489, 10975, 351, 64800, 842, 5332, 51079, 102, 805, 317, 322, 10313, 513, 4705, 4726 |
| 179 | Amphetamine addiction | 817, 5578, 111, 5500, 5579, 23411, 64764, 6804, 90993, 775, 818, 5530, 84288, 64800, 805, 9586, 2354, 814 |
| 180 | Dilated cardiomyopathy (DCM) | 3679, 64778, 115, 7040, 7402, 54847, 113, 111, 8516, 3675, 1756, 775, 3694, 7168, 7171, 1838, 7170, 3688, 4000, 60, 3693, 22862, 109, 71, 23335, 3672 |
| 181 | Aldosterone synthesis and secretion | 3949, 115, 817, 493, 5581, 5578, 113, 111, 5579, 64764, 90993, 775, 818, 221955, 84288, 3709, 57118, 25865, 64800, 5332, 805, 9586, 814, 109, 3164 |
| 182 | RNA transport | 7514, 1207, 79811, 754, 9972, 8661, 23279, 196441, 23165, 26019, 4928, 10419, 54815, 8662, 222229, 23191, 8668, 79830, 2631, 9631, 53371, 221749, 54879, 55746, 10940, 113452, 8087, 54952, 8667, 57187, 146712, 10482, 8672, 54558, 5976, 51095, 285237, 129401, 57510, 10460, 26999, 114786, 8669, 79140, 1039, 50628, 79902, 5411, 22794, 120400, 6414, 9818, 11097, 146177, 22985, 317649, 51808, 57122 |
| 183 | Proteasome | 5688, 5693, 23198, 129531, 5685, 23184, 51016, 22996, 5682, 5702, 5710 |
| 184 | Non-homologous end-joining | 91833, 4361, 5591, 1762 |
| 185 | Arrhythmogenic right ventricular cardiomyopathy (ARVC) | 3679, 3728, 1495, 7402, 54847, 8516, 3675, 1756, 55286, 775, 3694, 9204, 1838, 6934, 3688, 4000, 60, 3693, 71, 23335, 3672 |
| 186 | Glutamatergic synapse | 9592, 81539, 2782, 2788, 115, 2771, 22941, 10681, 2744, 388403, 5578, 113, 157, 111, 5594, 5579, 2917, 156, 775, 5530, 59345, 3709, 54331, 5332, 5337, 2898, 109, 22839 |
| 187 | Terpenoid backbone biosynthesis | 79703, 2339, 10269, 79947, 3157, 3156 |
| 188 | RIG-I-like receptor signaling pathway | 5599, 3551, 7186, 8717, 4214, 7706, 10010, 5602, 10906, 9474, 9636, 843, 5603, 4793, 64135, 57506 |
| 189 | Antifolate resistance | 10257, 3551, 4363, 10057, 6472, 64645, 7298, 8714 |
| 190 | Inflammatory bowel disease (IBD) | 3460, 4087, 3566, 7040, 149233, 3595, 6774, 3594, 50615, 6775, 6772, 4772, 4088 |
| 191 | Hippo signaling pathway | 6788, 1739, 5590, 125488, 117583, 54462, 8312, 55233, 376940, 57488, 4087, 55844, 7257, 1855, 126308, 1495, 25938, 4771, 7040, 57587, 7531, 659, 150864, 9842, 5500, 999, 440193, 7159, 79591, 199990, 23051, 5521, 50809, 9113, 55227, 119504, 1741, 1740, 79613, 1453, 80856, 8076, 655, 5939, 896, 162427, 91748, 6934, 59339, 162073, 55691, 51316, 79647, 780776, 54477, 55841, 65244, 3993, 79932, 7003, 10207, 199870, 60, 100129792, 657, 55654, 123355, 196074, 23291, 71, 23335, 80008, 4088, 339487 |
| 192 | Aminoacyl-tRNA biosynthesis | 10667, 2193, 57038, 2617, 79731, 23731, 83941, 5917, 5859, 3376, 23548, 8565 |
| 193 | Non-alcoholic fatty liver disease (NAFLD) | 4719, 5599, 3551, 7385, 6720, 7186, 7040, 83737, 9377, 4217, 4708, 5291, 6256, 5465, 51422, 4724, 5602, 5290, 208, 1345, 3643, 207, 10975, 79602, 51079, 5296, 5295, 4705, 7494, 4726 |
| 194 | Thyroid cancer | 5468, 388403, 5594, 6256, 999, 5604, 54879, 83941, 113452, 79613, 285237, 7170, 6934, 1039, 120400, 6414, 146177 |
| 195 | Butanoate metabolism | 18, 79944, 84747, 3157, 84656, 3155, 79746 |
| 196 | Pathogenic Escherichia coli infection | 10095, 2017, 3059, 5578, 6093, 999, 60685, 10109, 146712, 145282, 79613, 57609, 100506658, 3688, 25, 8976, 60, 7430, 71 |
| 197 | Hypertrophic cardiomyopathy (HCM) | 3679, 64778, 7040, 7402, 54847, 8516, 3675, 51422, 1756, 775, 3694, 7168, 7171, 1838, 7170, 3688, 4000, 60, 3693, 22862, 71, 23335, 3672 |
| 198 | Human papillomavirus infection | 3679, 1739, 5590, 125488, 472, 117583, 54462, 7337, 4799, 8312, 5663, 376940, 6655, 170394, 1108, 22889, 57488, 7057, 5747, 535, 3551, 55844, 7257, 3659, 25992, 1855, 22982, 148223, 51608, 25938, 2308, 8717, 10985, 9223, 57587, 388403, 54847, 150864, 6654, 7249, 129293, 5291, 182, 8516, 5527, 9842, 84441, 100505549, 5594, 91050, 440193, 3675, 2033, 79591, 55225, 199990, 23635, 23051, 5604, 1387, 57186, 5521, 221749, 50809, 51379, 55534, 6198, 3455, 64764, 90993, 11333, 1287, 5290, 208, 55227, 4193, 119504, 1741, 3694, 84928, 529, 1740, 5159, 283310, 55012, 80856, 8076, 148362, 57609, 5939, 113201, 896, 10906, 162427, 91748, 207, 6934, 59339, 162073, 51316, 79647, 780776, 54477, 9473, 26033, 3688, 65244, 54991, 3993, 79932, 3454, 3915, 100134938, 205428, 1956, 2217, 5525, 5296, 9636, 147650, 10207, 199870, 100129792, 55654, 1278, 159195, 55714, 54980, 9368, 9586, 23352, 123355, 6009, 5894, 3693, 196074, 140775, 125950, 5728, 6772, 5295, 3672, 80008, 56654, 23181, 339487 |
| 199 | Long-term depression | 3480, 5592, 9592, 2771, 388403, 5578, 5594, 5604, 5579, 3709, 5332, 4067, 5894, 2977 |
| 200 | Glyoxylate and dicarboxylate metabolism | 9380, 1431, 125061, 5095, 6472, 23255, 84656, 23334 |
| 201 | Citrate cycle (TCA cycle) | 4967, 1431, 8801, 3418, 1737, 5162, 3421, 5106 |
| 202 | Glycosphingolipid biosynthesis - ganglio series | 9197, 2720, 30815, 8869 |
| 203 | Sulfur metabolism | 23474, 9061 |
| 204 | Toll-like receptor signaling pathway | 5599, 3551, 6416, 388403, 5291, 5594, 941, 5604, 5602, 3455, 5290, 208, 1326, 114609, 10906, 207, 4615, 3454, 5296, 5603, 51015, 6772, 5295 |
| 205 | Histidine metabolism | 26, 138199, 55748, 3176, 217 |
| 206 | Cholesterol metabolism | 3949, 6646, 7417, 80765, 6653, 9217, 114815, 22937, 27329, 7419, 6272, 4043, 134429 |
| 207 | Glycosylphosphatidylinositol (GPI)-anchor biosynthesis | 23556, 2822, 9487, 84747, 80055, 51227 |
| 208 | Drug metabolism - other enzymes | 8615, 1806, 2948, 2946, 2944, 60526, 51727, 8824, 8833, 79830, 84450, 50484, 7498, 283848, 4258, 7371 |
| 209 | Cortisol synthesis and secretion | 5151, 3949, 115, 113, 111, 64764, 90993, 775, 3709, 5332, 9586, 109, 3164, 5087 |
| 210 | Riboflavin metabolism | 645, 80308 |
| 211 | Biosynthesis of unsaturated fatty acids | 7871, 9200, 51, 6342, 1528, 30 |
| 212 | Estrogen signaling pathway | 6655, 596, 115, 2771, 3765, 8648, 388403, 6654, 113, 5291, 111, 5594, 10499, 114825, 5604, 64764, 90993, 5290, 208, 1509, 84288, 3709, 207, 1956, 64800, 3880, 5332, 5296, 3326, 805, 9586, 5894, 5295, 109, 8202 |
| 213 | Bile secretion | 3949, 10257, 115, 343, 6548, 113, 111, 3781, 6256, 8671, 1080, 3475, 3156, 8714, 109 |
| 214 | Glycolysis / Gluconeogenesis | 124, 125, 126, 10327, 5214, 3099, 1737, 55293, 5162, 5211, 130589, 23255, 9562, 5106, 217 |
| 215 | alpha-Linolenic acid metabolism | 51, 147011, 151056, 1528, 5920, 30 |
| 216 | Pentose phosphate pathway | 7086, 5214, 5636, 5211, 5226, 25796 |
| 217 | Fatty acid elongation | 7871, 3032, 9200, 117145, 58489, 79746 |
| 218 | Vascular smooth muscle contraction | 5592, 115, 23365, 4638, 388403, 5581, 5578, 113, 4033, 6093, 111, 5594, 5500, 5604, 5579, 23190, 3778, 54776, 775, 145282, 10242, 147011, 84288, 3709, 135, 64800, 5332, 805, 5894, 2977, 109, 800 |
| 219 | Endocrine and other factor-regulated calcium reabsorption | 1785, 51430, 115, 1212, 5578, 7421, 5579, 161, 624, 5332, 1213 |
| 220 | PPAR signaling pathway | 1374, 34, 5468, 6256, 5465, 51, 6342, 2168, 51703, 2710, 23305, 7316, 5106, 1528, 30 |
| 221 | Insect hormone biosynthesis | 217 |
| 222 | Parkinson disease | 7326, 118424, 7417, 4719, 2771, 7385, 9377, 4708, 539, 111, 4724, 120892, 79887, 10476, 1345, 7419, 10975, 135, 842, 51079, 317, 291, 513, 4705, 4726 |
| 223 | Synthesis and degradation of ketone bodies | 3157, 3155 |
| 224 | Purine metabolism | 5151, 22978, 115, 60526, 9061, 113, 8833, 79830, 111, 957, 5140, 79591, 2272, 5636, 318, 50484, 58497, 55007, 955, 122622, 7498, 84284, 2984, 5143, 5144, 953, 2977, 5152, 109, 56654 |
| 225 | Other types of O-glycan biosynthesis | 79573, 10585, 55969, 8703, 9326, 23275, 84620 |
| 226 | Glycosaminoglycan biosynthesis - heparan sulfate / heparin | 29063, 2137, 57037, 79746, 1831 |
| 227 | Malaria | 3683, 7057, 7040, 6653, 6382, 4615, 22914, 100528032, 4233 |
| 228 | 2-Oxocarboxylic acid metabolism | 1431, 3418, 84680, 3421 |
| 229 | One carbon pool by folate | 121053, 4548, 6472, 7298 |
| 230 | Glutathione metabolism | 2948, 2946, 2944, 2730, 3418, 50484, 373156, 5226, 2936, 4258, 124975 |
| 231 | Neomycin, kanamycin and gentamicin biosynthesis | 3099 |
| 232 | Melanogenesis | 125488, 54462, 376940, 57488, 115, 2771, 817, 7257, 1855, 25938, 57587, 388403, 5578, 150864, 113, 111, 9842, 5594, 2033, 79591, 199990, 23051, 5604, 1387, 50809, 5579, 64764, 90993, 119504, 818, 80856, 8076, 5939, 84288, 162427, 91748, 6934, 59339, 162073, 51316, 79647, 780776, 54477, 65244, 79932, 64800, 5332, 199870, 100129792, 55654, 805, 5894, 196074, 109, 80008, 339487 |
| 233 | D-Glutamine and D-glutamate metabolism | 2744, 80017 |
| 234 | Wnt signaling pathway | 4040, 125488, 54462, 8312, 5663, 376940, 1488, 4041, 57488, 5599, 817, 7257, 1855, 51701, 25938, 57587, 1487, 5578, 4773, 150864, 91355, 9842, 4775, 2033, 79591, 199990, 23051, 1387, 50809, 56998, 5579, 5602, 6259, 119504, 5937, 818, 5530, 145282, 80856, 8076, 116966, 57680, 5939, 896, 162427, 91748, 6934, 59339, 162073, 51316, 79647, 780776, 54477, 65244, 79932, 79718, 5332, 752, 199870, 100129792, 55654, 27122, 9978, 196074, 23291, 23335, 80008, 4772, 339487 |
| 235 | Serotonergic synapse | 9592, 3360, 2782, 2788, 2771, 10681, 3765, 388403, 5578, 111, 3781, 5594, 5604, 5579, 1565, 775, 59345, 3709, 351, 54331, 5332, 5894 |
| 236 | Pertussis | 5599, 2771, 3659, 51279, 388403, 79830, 5594, 4843, 5602, 114609, 84288, 4615, 3688, 64800, 3394, 5603, 805 |
| 237 | Platelet activation | 5590, 5592, 170394, 115, 7408, 2771, 23365, 4638, 148223, 10235, 51608, 83660, 2909, 388403, 54847, 113, 5291, 6093, 111, 5594, 5500, 221749, 5336, 23214, 5290, 208, 145282, 57407, 283310, 695, 65059, 3709, 207, 162073, 9473, 3688, 54991, 5332, 5296, 147650, 60, 5603, 1278, 6850, 4067, 5906, 51466, 2977, 5295, 109, 71, 6786, 23648, 56654, 54518 |
| 238 | Breast cancer | 4040, 125488, 54462, 8312, 10443, 376940, 6655, 3480, 3714, 4041, 22889, 57488, 7257, 25992, 1855, 25938, 57587, 8648, 388403, 150864, 6654, 91355, 5291, 182, 9842, 5594, 79591, 199990, 23051, 2260, 5604, 2064, 50809, 6198, 83941, 5290, 208, 119504, 80856, 8076, 5939, 162427, 91748, 207, 6934, 59339, 162073, 51316, 79647, 780776, 54477, 65244, 79932, 205428, 1956, 5296, 199870, 100129792, 55654, 54980, 5894, 196074, 5728, 5295, 8202, 80008, 339487 |
| 239 | Leishmaniasis | 3460, 7040, 388403, 5594, 5579, 4843, 4615, 3688, 9644, 5603, 4793, 6772, 3717, 1536 |
| 240 | Thiamine metabolism | 27010, 100534012, 79005, 26010, 79591, 79178, 55007, 23184, 84284 |
| 241 | Circadian rhythm - fly | 8864 |
| 242 | Selenocompound metabolism | 9061, 4548, 84680 |
| 243 | Prion diseases | 22889, 25992, 388403, 5594, 5604, 3915, 205428, 388364, 54980 |
| 244 | IL-17 signaling pathway | 55833, 6310, 8848, 5599, 3551, 79041, 2919, 7186, 8717, 388403, 64788, 7188, 5594, 23264, 5597, 79832, 5602, 23241, 727897, 283310, 1994, 10906, 29761, 5603, 283987, 3326, 57655, 2354, 9618 |
| 245 | AGE-RAGE signaling pathway in diabetic complications | 5590, 51196, 170394, 6776, 596, 5599, 4087, 113026, 148223, 51608, 2308, 7040, 388403, 5581, 5578, 1729, 5291, 5594, 23635, 57459, 221749, 5579, 5602, 5336, 23214, 1287, 5290, 208, 6774, 207, 162073, 9473, 54991, 6777, 5332, 5296, 147650, 5603, 1278, 80206, 6772, 5295, 23648, 3717, 4772, 84514, 56654, 1536, 4088, 7423 |
| 246 | Tryptophan metabolism | 54497, 4967, 26, 25938, 125061, 84680, 79746, 217 |
| 247 | Renin secretion | 2771, 22802, 111, 5140, 3778, 775, 5530, 1508, 84288, 3709, 30816, 64800, 5332, 805, 2977 |
| 248 | Phenylalanine, tyrosine and tryptophan biosynthesis | 84680 |
| 249 | Starch and sucrose metabolism | 7360, 2595, 6476, 3099, 2997, 93432 |
| 250 | Synaptic vesicle cycle | 1785, 51430, 9592, 535, 1212, 6536, 161, 6804, 149473, 529, 6844, 10497, 6809, 1213, 9847 |
| 251 | Salivary secretion | 1755, 5592, 115, 6558, 493, 6548, 5578, 113, 111, 5579, 3778, 6263, 727897, 6844, 84288, 3475, 3709, 130872, 136853, 64800, 5332, 805, 125950, 2977, 109 |
| 252 | Alcoholism | 79885, 6655, 2782, 2788, 2771, 10681, 51564, 388403, 6654, 8520, 9734, 111, 5594, 5500, 5604, 64764, 90993, 10014, 59345, 84288, 4915, 10645, 135, 54331, 8365, 64800, 100996485, 8351, 9555, 805, 9586, 2354, 5894, 814, 3020, 55869 |
| 253 | Metabolic pathways (no map in kegg database) | 4124, 4249, 262, 200576, 729920, 9380, 124, 125, 126, 22978, 201595, 79719, 9197, 54497, 7360, 2530, 23193, 8527, 8578, 2673, 34, 256987, 4967, 8615, 27010, 64802, 346606, 8566, 3632, 29063, 23556, 23646, 1788, 2720, 23530, 23175, 1806, 51196, 79811, 11282, 2595, 66008, 1606, 2762, 6476, 283358, 30815, 18, 5373, 1431, 10585, 9215, 4719, 79370, 10955, 535, 8897, 22856, 1038, 60526, 100534012, 79005, 8869, 26, 1635, 7385, 8821, 23382, 2744, 253558, 80025, 2194, 23589, 1789, 2643, 113026, 7086, 645, 51727, 2590, 445, 9489, 8760, 25938, 23396, 199857, 84105, 5372, 55748, 9061, 5287, 4047, 8801, 80271, 129642, 9377, 10327, 11343, 9334, 64788, 7871, 3032, 4708, 5860, 2730, 197258, 6185, 539, 4548, 32, 26010, 79053, 11112, 8833, 2524, 9249, 5291, 79830, 125061, 79001, 2822, 56994, 10005, 23417, 80146, 5297, 253782, 8394, 5214, 79591, 10020, 3707, 5286, 8526, 85465, 133308, 3418, 4843, 4724, 57732, 9200, 56922, 139322, 5636, 51, 84680, 29880, 5336, 2137, 9487, 3099, 29785, 1737, 6342, 5290, 133686, 10873, 5095, 84747, 158219, 84816, 284001, 5298, 54187, 55293, 131177, 340485, 84450, 529, 2222, 50484, 3633, 3631, 5162, 79178, 4048, 51703, 31, 5211, 152002, 10170, 8703, 1503, 55007, 10476, 1345, 130589, 80055, 10423, 3636, 5226, 57037, 147011, 55361, 55577, 122622, 7390, 6472, 2997, 80142, 2710, 8871, 23255, 1953, 84795, 23184, 85007, 7498, 10975, 55500, 10905, 25796, 81932, 3157, 57688, 26033, 64419, 84284, 93432, 84656, 3423, 9663, 23761, 5048, 93100, 5332, 51079, 5289, 22996, 57104, 4122, 3294, 283848, 4245, 5337, 23305, 51809, 9517, 9562, 5305, 138050, 3156, 65220, 1152, 3712, 23334, 5859, 151056, 80127, 56623, 3155, 7371, 84620, 64747, 23548, 7298, 3421, 4199, 8702, 513, 5728, 79746, 5106, 91663, 4534, 22908, 11320, 5130, 79796, 10229, 4705, 427, 148789, 51227, 4726, 2677, 124975, 80308, 217, 5920, 30, 3163, 3705 |
| 254 | Phagosome | 200576, 7057, 8411, 6645, 535, 1520, 1783, 51279, 9341, 9146, 203197, 54847, 50809, 27297, 821, 7879, 146712, 529, 7037, 3916, 64081, 23184, 23673, 1778, 3688, 57590, 5868, 2217, 5289, 9644, 60, 3693, 51143, 71, 91663, 1536 |
| 255 | Cushing syndrome | 5151, 3949, 125488, 54462, 8312, 376940, 8085, 57488, 115, 2771, 817, 7257, 1855, 27324, 25938, 57587, 388403, 150864, 113, 111, 9842, 9101, 5594, 79591, 199990, 23051, 5604, 50809, 84954, 64764, 90993, 119504, 9070, 775, 818, 80856, 8076, 5939, 162427, 91748, 3709, 11091, 6934, 59339, 162073, 51316, 79647, 780776, 54477, 65244, 79932, 1956, 5332, 199870, 100129792, 55654, 9586, 5906, 196074, 109, 80008, 3164, 5087, 339487 |
| 256 | Autophagy - other | 10533, 55062, 221960, 9474, 57521, 5289 |
| 257 | Herpes simplex infection | 5819, 3460, 11030, 4940, 27097, 6874, 1038, 5599, 3551, 80139, 7186, 8864, 7188, 6429, 3431, 5500, 5781, 2033, 55225, 1387, 8764, 54926, 440275, 5602, 3455, 79157, 10629, 6875, 1936, 10482, 84310, 11180, 51237, 10906, 5187, 4615, 3454, 8863, 54758, 83860, 3054, 2217, 6732, 6427, 9569, 4793, 5430, 64135, 93349, 57506, 972, 125950, 6772, 85456, 3717, 84514, 79641 |
| 258 | Cysteine and methionine metabolism | 262, 1788, 23382, 1789, 2730, 4548, 84680, 55293 |
| 259 | Glycosaminoglycan biosynthesis - chondroitin sulfate / dermatan sulfate | 22856, 51363, 50515 |
| 260 | Ribosome biogenesis in eukaryotes | 144404, 7514, 27341, 51096, 57455, 54552, 22803, 23078, 6645, 51602, 10171, 10940, 23195, 79613, 10482, 285237, 55341, 4931, 55813, 55226 |
| 261 | Drug metabolism - cytochrome P450 | 124, 125, 126, 54497, 2948, 2946, 2944, 25938, 1565, 373156, 4258 |
| 262 | Mineral absorption | 54822, 4891, 7421, 1811, 140803, 65010, 1317, 3163 |
| 263 | Carbon metabolism | 34, 4967, 1431, 7086, 8801, 5214, 3418, 5636, 84680, 3099, 1737, 10873, 5095, 84747, 5162, 5211, 5226, 6472, 23255, 25796, 84656, 80127, 3421, 4199, 79746 |
| 264 | Viral myocarditis | 3683, 7402, 27, 941, 1756, 132320, 8672, 1838, 842, 2217, 25, 60, 71 |
| 265 | Glycosaminoglycan degradation | 2720, 3423, 138050 |
| 266 | Primary bile acid biosynthesis | 10005, 6342 |
| 267 | Arginine biosynthesis | 2744, 445, 4843, 84680 |
| 268 | Maturity onset diabetes of the young | 79618, 55571, 2494, 3172 |
| 269 | Other glycan degradation | 2720, 4126, 23324 |
| 270 | Relaxin signaling pathway | 5590, 6655, 170394, 2782, 2788, 115, 5599, 2771, 4087, 10681, 148223, 51608, 408, 7040, 6416, 388403, 79819, 5578, 6654, 113, 5291, 111, 5594, 23635, 5604, 221749, 4843, 5602, 64764, 90993, 23214, 1287, 5290, 208, 59345, 207, 162073, 9473, 54991, 54331, 1956, 5332, 5296, 147650, 5603, 1278, 9586, 5894, 5295, 109, 23648, 56654, 4088, 7423 |
| 271 | Antigen processing and presentation | 6645, 1520, 203197, 2923, 7247, 10437, 129531, 50809, 27297, 821, 1508, 64081, 23184, 51016, 2217, 3326, 972, 91663 |
| 272 | Thyroid hormone synthesis | 115, 5578, 113, 111, 5579, 64764, 90993, 821, 3709, 5332, 2936, 9586, 109 |
| 273 | Spliceosome | 79811, 9716, 196441, 1659, 6429, 51362, 23451, 3183, 55969, 256536, 6434, 91133, 55660, 79157, 57187, 9410, 4670, 10992, 10594, 162427, 11325, 11188, 6625, 84456, 22938, 6427, 144983, 8233, 8175, 22985, 54799, 51691 |
| 274 | Ovarian steroidogenesis | 3949, 3480, 115, 113, 111, 3643, 3294, 109 |
| 275 | Sulfur relay system | 6856, 100534012, 79005, 26010, 23184 |
| 276 | Ether lipid metabolism | 23646, 56994, 85465, 147011, 5048, 5337, 151056, 284161, 5920 |
| 277 | Tyrosine metabolism | 124, 125, 126, 54497, 23589, 25938, 84680 |
| 278 | Folate biosynthesis | 2643, 84105, 5860, 84816, 55034 |
| 279 | Alanine, aspartate and glutamate metabolism | 2673, 18, 2744, 445, 84680, 122622 |
| 280 | Amyotrophic lateral sclerosis (ALS) | 596, 259173, 4217, 5530, 598, 5868, 842, 5603, 317, 51015 |
| 281 | Arginine and proline metabolism | 262, 26, 55748, 4843, 139322, 1152, 91663, 217 |
| 282 | Biosynthesis of amino acids | 1431, 7086, 445, 4548, 5214, 3418, 5636, 84680, 5211, 6472, 3421, 91663 |
| 283 | Proximal tubule bicarbonate reclamation | 2744, 8671, 762, 5106 |
| 284 | Pantothenate and CoA biosynthesis | 1806, 80025 |
| 285 | Nitrogen metabolism | 377677, 771, 762 |
| 286 | Steroid biosynthesis | 6646, 4047, 2222 |
| 287 | Amoebiasis | 170394, 57224, 4583, 5747, 81, 199953, 22982, 148223, 51608, 25938, 7040, 1992, 29998, 5578, 129293, 5291, 100505549, 23013, 51599, 23635, 221749, 79098, 5579, 4843, 51379, 6311, 58986, 79567, 169611, 11333, 23214, 1287, 5290, 7879, 92736, 84928, 23241, 54897, 4302, 148362, 57609, 113201, 23145, 162073, 25849, 26053, 7414, 9473, 9672, 26033, 54991, 5868, 3915, 100131755, 100134938, 87, 5332, 57605, 5296, 114823, 147650, 1278, 159195, 140688, 140775, 5295, 23199, 23648, 25829, 56654, 23181 |
| 288 | Huntington disease | 3064, 11030, 6874, 7417, 4719, 1038, 5468, 7385, 80139, 1212, 9377, 79819, 25942, 4708, 539, 2033, 1387, 4724, 161, 64764, 90993, 6875, 10476, 84310, 1345, 7419, 23186, 10975, 54758, 246721, 842, 5332, 51079, 3092, 22893, 5430, 317, 9586, 291, 1213, 513, 85456, 84514, 4705, 1767, 5431, 4726, 79641 |
| 289 | Legionellosis | 5861, 2919, 54676, 4615, 842, 56681, 317, 23786, 375 |
| 290 | Intestinal immune network for IgA production | 5284, 9020, 7040, 941, 8174, 4055 |
| 291 | Cardiac muscle contraction | 7385, 6548, 9377, 132320, 775, 7168, 7171, 1345, 7170, 10975, 23335 |
| 292 | Mucin type O-glycan biosynthesis | 79719, 2590, 9334, 51809 |
| 293 | ECM-receptor interaction | 3679, 170394, 7057, 22982, 148223, 51608, 25938, 3339, 54847, 129293, 8516, 100505549, 3675, 23635, 221749, 51379, 11333, 1287, 8495, 3694, 84928, 6382, 283310, 148362, 57609, 113201, 162073, 9473, 960, 26033, 3688, 54991, 3915, 100134938, 147650, 1278, 159195, 55714, 100526694, 8577, 6385, 3693, 140775, 3672, 56654, 23181 |
| 294 | Basal cell carcinoma | 125488, 54462, 8312, 376940, 57488, 7257, 1855, 25938, 57587, 150864, 9842, 79591, 199990, 23051, 51684, 50809, 83941, 119504, 6608, 80856, 8076, 5939, 162427, 91748, 6934, 59339, 162073, 51316, 79647, 780776, 54477, 65244, 79932, 64399, 199870, 100129792, 55654, 196074, 80008, 339487 |
| 295 | Basal transcription factors | 27097, 6874, 6883, 2068, 10629, 6875, 84310, 11180, 83860, 9569, 8148 |
| 296 | Porphyrin and chlorophyll metabolism | 148534, 100527978, 645, 7390, 81932, 3163 |
| 297 | Retinol metabolism | 124, 125, 126, 54497, 25938, 9249, 29785, 158219, 10170, 22996 |
| 298 | Glycosphingolipid biosynthesis - lacto and neolacto series | 2524, 8703, 8702 |
| 299 | RNA polymerase | 11030, 55703, 1038, 10623, 80139, 54758, 246721, 5430, 85456, 84514, 5431, 79641 |
| 300 | African trypanosomiasis | 5578, 5579, 4615, 5332 |
| 301 | Fat digestion and absorption | 346606, 2168, 158219, 147011, 22996 |
| 302 | Glycine, serine and threonine metabolism | 9380, 23464, 107080644, 6472 |
| 303 | Type I diabetes mellitus | 5799, 3382, 941, 1363, 2217 |
| 304 | Metabolism of xenobiotics by cytochrome P450 | 124, 125, 126, 2948, 2946, 2944, 1565, 29785, 373156, 4258 |
| 305 | Cocaine addiction | 2771, 111, 64764, 90993, 9586, 2354 |
| 306 | Taurine and hypotaurine metabolism | 124975 |
| 307 | Oxidative phosphorylation | 27068, 8578, 4719, 535, 7385, 9377, 4708, 539, 4724, 529, 10476, 1345, 1953, 10975, 51079, 513, 4705, 4726 |
| 308 | Glycosphingolipid biosynthesis - globo and isoglobo series | 2524 |
| 309 | Nicotine addiction | 112476, 9592, 4302, 155435, 8028, 2570 |
| 310 | Primary immunodeficiency | 3932, 7247, 695 |
| 311 | Linoleic acid metabolism | 147011, 151056, 5920 |
| 312 | Pentose and glucuronate interconversions | 729920, 7360, 10327 |
| 313 | Cell adhesion molecules (CAMs) | 3683, 5819, 1364, 2734, 9378, 5797, 8516, 999, 941, 5792, 79613, 6382, 1366, 1272, 100506658, 9672, 3688, 2217, 388364, 8174, 6385, 83692, 257194 |
| 314 | Collecting duct acid secretion | 535, 529 |
| 315 | Phototransduction | 2782, 9147, 84288, 64800, 805 |
| 316 | Phenylalanine metabolism | 84680 |
| 317 | Chemical carcinogenesis | 124, 125, 126, 2948, 2946, 2944, 84680, 373156, 4258 |
| 318 | Sphingolipid metabolism | 2720, 253782, 340485, 9517, 427 |
| 319 | Cytosolic DNA-sensing pathway | 55703, 3551, 10623, 115004, 4793, 57506 |
| 320 | Graft-versus-host disease | 941, 2217 |
| 321 | Renin-angiotensin system | 57486 |
| 322 | Allograft rejection | 941, 2217 |
| 323 | Ascorbate and aldarate metabolism | 217 |
| 324 | Taste transduction | 9592, 775, 4302, 130872, 11079, 6340, 8028, 56654 |
| 325 | Arachidonic acid metabolism | 4048, 147011, 80142, 151056, 5920 |
| 326 | Rheumatoid arthritis | 8792, 3683, 535, 7040, 941, 529 |
| 327 | Steroid hormone biosynthesis | 79154, 7871, 3294 |
| 328 | Autoimmune thyroid disease | 941, 2217 |
| 329 | Cytokine-cytokine receptor interaction | 8792, 3460, 92, 3977, 1439, 2919, 3566, 7040, 27242, 659, 163702, 91, 8764, 768211, 149233, 3455, 8718, 3595, 22874, 8743, 3572, 655, 3594, 5008, 50615, 55504, 3454, 58985, 4804, 657, 9466, 3597, 4055 |
| 330 | Staphylococcus aureus infection | 3683, 51279, 79830 |
| 331 | Ribosome | 55831, 25963, 7257, 64968, 51263, 6193, 149466, 84928, 28998, 8076, 83541, 55052, 6141, 26151, 150372, 23080 |
| 332 | Hematopoietic cell lineage | 3566, 3675, 7037, 924, 960, 3672 |
| 333 | Systemic lupus erythematosus | 81, 51279, 941, 8365, 87, 100996485, 8351, 9555, 3020 |
| 334 | Complement and coagulation cascades | 2, 3687, 51279, 5858, 283310, 624 |
| 335 | Protein digestion and absorption | 23196, 170394, 23254, 6520, 283234, 148223, 51608, 113263, 1954, 727957, 22904, 6546, 23635, 221749, 255631, 23214, 1287, 3784, 22980, 162073, 23255, 57597, 9473, 254427, 54991, 205428, 147650, 1278, 57666, 7512, 286046, 85441, 23648, 84514, 56654 |
| 336 | Olfactory transduction | 5592, 2782, 2788, 817, 84067, 408, 157, 6546, 156, 818, 84288, 30816, 64800, 805, 109, 28984 |
| 337 | Neuroactive ligand-receptor interaction | 117583, 3269, 112476, 3360, 7068, 79819, 54847, 79890, 79830, 5025, 7067, 2917, 9340, 7433, 4302, 5029, 624, 84173, 1902, 130872, 79140, 135, 2898, 155435, 8028, 2570, 6870 |
