# Supplementary material for: Improvement of intestinal barrier function, gut microbiota, and metabolic endotoxemia in type 2 diabetes rats by curcumin
Source: Bioengineered. 2021 Dec 19;12(2):11947–58. doi: 10.1080/21655979.2021.2009322 (PMC8810160; doi:10.1080/21655979.2021.2009322)
Supplement: Supplemental Material [file KBIE_A_2009322_SM6765.zip › supplementary details/N-VS-UC.DEGseq_Method_P.html]

Terms for N-VS-UC.DEGseq\_Method\_P


## Terms for N-VS-UC.DEGseq\_Method\_P

---


### Result Table

|  |
| --- |
| **Terms from the Process Ontology with p-value as good or better than 1** |

| Gene Ontology term | Cluster frequency | Genome frequency of use | Corrected P-value |  |
| --- | --- | --- | --- | --- |
| organelle organization | 769 out of 2891 genes, 26.6% | 2842 out of 15382 genes, 18.5% | 8.66e-30 |  |
| cellular component organization | 1136 out of 2891 genes, 39.3% | 4836 out of 15382 genes, 31.4% | 1.03e-19 |  |
| cellular component organization or biogenesis | 1159 out of 2891 genes, 40.1% | 5017 out of 15382 genes, 32.6% | 2.37e-17 |  |
| regulation of cellular component organization | 562 out of 2891 genes, 19.4% | 2165 out of 15382 genes, 14.1% | 2.53e-15 |  |
| vesicle-mediated transport | 409 out of 2891 genes, 14.1% | 1488 out of 15382 genes, 9.7% | 2.37e-14 |  |
| macromolecule localization | 518 out of 2891 genes, 17.9% | 1986 out of 15382 genes, 12.9% | 3.50e-14 |  |
| cellular localization | 502 out of 2891 genes, 17.4% | 1913 out of 15382 genes, 12.4% | 3.73e-14 |  |
| macromolecule modification | 688 out of 2891 genes, 23.8% | 2803 out of 15382 genes, 18.2% | 1.91e-13 |  |
| protein localization | 455 out of 2891 genes, 15.7% | 1740 out of 15382 genes, 11.3% | 3.89e-12 |  |
| intracellular transport | 317 out of 2891 genes, 11.0% | 1125 out of 15382 genes, 7.3% | 6.75e-12 |  |
| covalent chromatin modification | 141 out of 2891 genes, 4.9% | 397 out of 15382 genes, 2.6% | 8.23e-12 |  |
| cellular protein modification process | 623 out of 2891 genes, 21.5% | 2545 out of 15382 genes, 16.5% | 2.10e-11 |  |
| protein modification process | 623 out of 2891 genes, 21.5% | 2545 out of 15382 genes, 16.5% | 2.10e-11 |  |
| localization | 1066 out of 2891 genes, 36.9% | 4744 out of 15382 genes, 30.8% | 5.33e-11 |  |
| establishment of localization in cell | 359 out of 2891 genes, 12.4% | 1329 out of 15382 genes, 8.6% | 6.23e-11 |  |
| membrane organization | 229 out of 2891 genes, 7.9% | 766 out of 15382 genes, 5.0% | 1.15e-10 |  |
| establishment of localization | 872 out of 2891 genes, 30.2% | 3789 out of 15382 genes, 24.6% | 2.01e-10 |  |
| transport | 849 out of 2891 genes, 29.4% | 3678 out of 15382 genes, 23.9% | 2.52e-10 |  |
| regulation of organelle organization | 304 out of 2891 genes, 10.5% | 1105 out of 15382 genes, 7.2% | 8.07e-10 |  |
| cellular protein metabolic process | 716 out of 2891 genes, 24.8% | 3045 out of 15382 genes, 19.8% | 1.25e-09 |  |
| cytoskeleton organization | 246 out of 2891 genes, 8.5% | 858 out of 15382 genes, 5.6% | 1.99e-09 |  |
| establishment of protein localization | 339 out of 2891 genes, 11.7% | 1274 out of 15382 genes, 8.3% | 3.44e-09 |  |
| protein transport | 322 out of 2891 genes, 11.1% | 1204 out of 15382 genes, 7.8% | 7.27e-09 |  |
| regulation of cellular metabolic process | 1183 out of 2891 genes, 40.9% | 5425 out of 15382 genes, 35.3% | 9.72e-09 |  |
| peptide transport | 326 out of 2891 genes, 11.3% | 1228 out of 15382 genes, 8.0% | 1.41e-08 |  |
| actin cytoskeleton organization | 134 out of 2891 genes, 4.6% | 404 out of 15382 genes, 2.6% | 1.49e-08 |  |
| intracellular signal transduction | 373 out of 2891 genes, 12.9% | 1445 out of 15382 genes, 9.4% | 1.89e-08 |  |
| organic substance transport | 438 out of 2891 genes, 15.2% | 1751 out of 15382 genes, 11.4% | 2.88e-08 |  |
| cellular macromolecule metabolic process | 1279 out of 2891 genes, 44.2% | 5942 out of 15382 genes, 38.6% | 3.08e-08 |  |
| amide transport | 328 out of 2891 genes, 11.3% | 1247 out of 15382 genes, 8.1% | 4.16e-08 |  |
| endomembrane system organization | 117 out of 2891 genes, 4.0% | 343 out of 15382 genes, 2.2% | 4.93e-08 |  |
| regulation of metabolic process | 1251 out of 2891 genes, 43.3% | 5819 out of 15382 genes, 37.8% | 1.07e-07 |  |
| cellular protein localization | 315 out of 2891 genes, 10.9% | 1197 out of 15382 genes, 7.8% | 1.08e-07 |  |
| actin filament-based process | 147 out of 2891 genes, 5.1% | 468 out of 15382 genes, 3.0% | 1.35e-07 |  |
| macromolecule metabolic process | 1356 out of 2891 genes, 46.9% | 6371 out of 15382 genes, 41.4% | 1.37e-07 |  |
| nitrogen compound transport | 370 out of 2891 genes, 12.8% | 1453 out of 15382 genes, 9.4% | 1.62e-07 |  |
| chromatin organization | 172 out of 2891 genes, 5.9% | 573 out of 15382 genes, 3.7% | 1.72e-07 |  |
| regulation of catabolic process | 227 out of 2891 genes, 7.9% | 809 out of 15382 genes, 5.3% | 1.78e-07 |  |
| metabolic process | 1725 out of 2891 genes, 59.7% | 8339 out of 15382 genes, 54.2% | 2.12e-07 |  |
| cellular macromolecule localization | 315 out of 2891 genes, 10.9% | 1204 out of 15382 genes, 7.8% | 2.27e-07 |  |
| protein metabolic process | 782 out of 2891 genes, 27.0% | 3446 out of 15382 genes, 22.4% | 2.38e-07 |  |
| histone modification | 99 out of 2891 genes, 3.4% | 281 out of 15382 genes, 1.8% | 2.56e-07 |  |
| regulation of response to stimulus | 787 out of 2891 genes, 27.2% | 3488 out of 15382 genes, 22.7% | 7.02e-07 |  |
| negative regulation of cellular process | 891 out of 2891 genes, 30.8% | 4011 out of 15382 genes, 26.1% | 7.64e-07 |  |
| regulation of macromolecule metabolic process | 1148 out of 2891 genes, 39.7% | 5324 out of 15382 genes, 34.6% | 8.45e-07 |  |
| regulation of primary metabolic process | 1145 out of 2891 genes, 39.6% | 5309 out of 15382 genes, 34.5% | 8.67e-07 |  |
| primary metabolic process | 1598 out of 2891 genes, 55.3% | 7686 out of 15382 genes, 50.0% | 8.75e-07 |  |
| Golgi vesicle transport | 103 out of 2891 genes, 3.6% | 302 out of 15382 genes, 2.0% | 8.96e-07 |  |
| cellular metabolic process | 1622 out of 2891 genes, 56.1% | 7817 out of 15382 genes, 50.8% | 1.00e-06 |  |
| regulation of small GTPase mediated signal transduction | 97 out of 2891 genes, 3.4% | 282 out of 15382 genes, 1.8% | 1.82e-06 |  |
| chromosome organization | 244 out of 2891 genes, 8.4% | 906 out of 15382 genes, 5.9% | 2.55e-06 |  |
| positive regulation of biological process | 1091 out of 2891 genes, 37.7% | 5052 out of 15382 genes, 32.8% | 2.66e-06 |  |
| regulation of nitrogen compound metabolic process | 1109 out of 2891 genes, 38.4% | 5151 out of 15382 genes, 33.5% | 3.72e-06 |  |
| cellular response to stress | 364 out of 2891 genes, 12.6% | 1460 out of 15382 genes, 9.5% | 4.03e-06 |  |
| nitrogen compound metabolic process | 1500 out of 2891 genes, 51.9% | 7196 out of 15382 genes, 46.8% | 4.05e-06 |  |
| organic substance metabolic process | 1660 out of 2891 genes, 57.4% | 8049 out of 15382 genes, 52.3% | 4.11e-06 |  |
| viral life cycle | 53 out of 2891 genes, 1.8% | 126 out of 15382 genes, 0.8% | 8.26e-06 |  |
| regulation of nucleobase-containing compound metabolic process | 821 out of 2891 genes, 28.4% | 3709 out of 15382 genes, 24.1% | 1.41e-05 |  |
| negative regulation of macromolecule metabolic process | 498 out of 2891 genes, 17.2% | 2118 out of 15382 genes, 13.8% | 1.97e-05 |  |
| regulation of gene expression, epigenetic | 69 out of 2891 genes, 2.4% | 186 out of 15382 genes, 1.2% | 2.00e-05 |  |
| positive regulation of cellular process | 979 out of 2891 genes, 33.9% | 4518 out of 15382 genes, 29.4% | 2.14e-05 |  |
| regulation of cellular macromolecule biosynthetic process | 792 out of 2891 genes, 27.4% | 3571 out of 15382 genes, 23.2% | 2.19e-05 |  |
| endocytosis | 130 out of 2891 genes, 4.5% | 429 out of 15382 genes, 2.8% | 2.53e-05 |  |
| regulation of gene expression | 872 out of 2891 genes, 30.2% | 3988 out of 15382 genes, 25.9% | 4.55e-05 |  |
| negative regulation of cellular metabolic process | 495 out of 2891 genes, 17.1% | 2118 out of 15382 genes, 13.8% | 5.43e-05 |  |
| regulation of cellular process | 1914 out of 2891 genes, 66.2% | 9478 out of 15382 genes, 61.6% | 5.50e-05 |  |
| endosomal transport | 79 out of 2891 genes, 2.7% | 228 out of 15382 genes, 1.5% | 5.61e-05 |  |
| regulation of macromolecule biosynthetic process | 805 out of 2891 genes, 27.8% | 3655 out of 15382 genes, 23.8% | 6.13e-05 |  |
| negative regulation of metabolic process | 545 out of 2891 genes, 18.9% | 2367 out of 15382 genes, 15.4% | 7.83e-05 |  |
| regulation of signal transduction | 595 out of 2891 genes, 20.6% | 2615 out of 15382 genes, 17.0% | 9.17e-05 |  |
| phosphorus metabolic process | 419 out of 2891 genes, 14.5% | 1761 out of 15382 genes, 11.4% | 0.00010 |  |
| regulation of cellular catabolic process | 187 out of 2891 genes, 6.5% | 688 out of 15382 genes, 4.5% | 0.00015 |  |
| negative regulation of nitrogen compound metabolic process | 458 out of 2891 genes, 15.8% | 1955 out of 15382 genes, 12.7% | 0.00016 |  |
| positive regulation of metabolic process | 658 out of 2891 genes, 22.8% | 2939 out of 15382 genes, 19.1% | 0.00018 |  |
| positive regulation of cellular metabolic process | 616 out of 2891 genes, 21.3% | 2731 out of 15382 genes, 17.8% | 0.00019 |  |
| cytosolic transport | 47 out of 2891 genes, 1.6% | 115 out of 15382 genes, 0.7% | 0.00020 |  |
| viral process | 142 out of 2891 genes, 4.9% | 494 out of 15382 genes, 3.2% | 0.00021 |  |
| intracellular protein transport | 178 out of 2891 genes, 6.2% | 652 out of 15382 genes, 4.2% | 0.00024 |  |
| cell cycle | 309 out of 2891 genes, 10.7% | 1252 out of 15382 genes, 8.1% | 0.00028 |  |
| regulation of biosynthetic process | 846 out of 2891 genes, 29.3% | 3892 out of 15382 genes, 25.3% | 0.00029 |  |
| regulation of cellular response to stress | 155 out of 2891 genes, 5.4% | 554 out of 15382 genes, 3.6% | 0.00034 |  |
| negative regulation of biological process | 953 out of 2891 genes, 33.0% | 4442 out of 15382 genes, 28.9% | 0.00036 |  |
| positive regulation of cellular component organization | 266 out of 2891 genes, 9.2% | 1056 out of 15382 genes, 6.9% | 0.00040 |  |
| regulation of RNA metabolic process | 741 out of 2891 genes, 25.6% | 3370 out of 15382 genes, 21.9% | 0.00044 |  |
| phosphate-containing compound metabolic process | 396 out of 2891 genes, 13.7% | 1672 out of 15382 genes, 10.9% | 0.00049 |  |
| regulation of cell morphogenesis | 118 out of 2891 genes, 4.1% | 400 out of 15382 genes, 2.6% | 0.00067 |  |
| regulation of plasma membrane bounded cell projection organization | 147 out of 2891 genes, 5.1% | 525 out of 15382 genes, 3.4% | 0.00072 |  |
| regulation of cellular biosynthetic process | 828 out of 2891 genes, 28.6% | 3820 out of 15382 genes, 24.8% | 0.00076 |  |
| regulation of cellular localization | 202 out of 2891 genes, 7.0% | 770 out of 15382 genes, 5.0% | 0.00078 |  |
| positive regulation of macromolecule metabolic process | 610 out of 2891 genes, 21.1% | 2727 out of 15382 genes, 17.7% | 0.00084 |  |
| organonitrogen compound metabolic process | 955 out of 2891 genes, 33.0% | 4470 out of 15382 genes, 29.1% | 0.00084 |  |
| positive regulation of nitrogen compound metabolic process | 589 out of 2891 genes, 20.4% | 2623 out of 15382 genes, 17.1% | 0.00085 |  |
| regulation of programmed cell death | 338 out of 2891 genes, 11.7% | 1404 out of 15382 genes, 9.1% | 0.00094 |  |
| regulation of cell projection organization | 148 out of 2891 genes, 5.1% | 532 out of 15382 genes, 3.5% | 0.00101 |  |
| mitotic cell cycle | 174 out of 2891 genes, 6.0% | 647 out of 15382 genes, 4.2% | 0.00105 |  |
| positive regulation of RNA metabolic process | 311 out of 2891 genes, 10.8% | 1279 out of 15382 genes, 8.3% | 0.00117 |  |
| peptidyl-amino acid modification | 173 out of 2891 genes, 6.0% | 644 out of 15382 genes, 4.2% | 0.00123 |  |
| proteolysis involved in cellular protein catabolic process | 142 out of 2891 genes, 4.9% | 508 out of 15382 genes, 3.3% | 0.00133 |  |
| organelle localization | 146 out of 2891 genes, 5.1% | 526 out of 15382 genes, 3.4% | 0.00143 |  |
| cellular protein catabolic process | 148 out of 2891 genes, 5.1% | 535 out of 15382 genes, 3.5% | 0.00147 |  |
| Golgi organization | 38 out of 2891 genes, 1.3% | 90 out of 15382 genes, 0.6% | 0.00156 |  |
| import into cell | 135 out of 2891 genes, 4.7% | 479 out of 15382 genes, 3.1% | 0.00160 |  |
| regulation of protein metabolic process | 538 out of 2891 genes, 18.6% | 2382 out of 15382 genes, 15.5% | 0.00161 |  |
| regulation of chromatin organization | 52 out of 2891 genes, 1.8% | 141 out of 15382 genes, 0.9% | 0.00211 |  |
| positive regulation of nucleobase-containing compound metabolic process | 355 out of 2891 genes, 12.3% | 1496 out of 15382 genes, 9.7% | 0.00211 |  |
| regulation of Ras protein signal transduction | 65 out of 2891 genes, 2.2% | 191 out of 15382 genes, 1.2% | 0.00241 |  |
| cytoskeleton-dependent intracellular transport | 51 out of 2891 genes, 1.8% | 138 out of 15382 genes, 0.9% | 0.00253 |  |
| histone methylation | 31 out of 2891 genes, 1.1% | 68 out of 15382 genes, 0.4% | 0.00257 |  |
| regulation of apoptotic process | 332 out of 2891 genes, 11.5% | 1390 out of 15382 genes, 9.0% | 0.00279 |  |
| regulation of chromosome organization | 86 out of 2891 genes, 3.0% | 276 out of 15382 genes, 1.8% | 0.00295 |  |
| protein catabolic process | 157 out of 2891 genes, 5.4% | 581 out of 15382 genes, 3.8% | 0.00311 |  |
| myeloid cell differentiation | 60 out of 2891 genes, 2.1% | 173 out of 15382 genes, 1.1% | 0.00323 |  |
| regulation of cell death | 357 out of 2891 genes, 12.3% | 1512 out of 15382 genes, 9.8% | 0.00333 |  |
| proteolysis | 230 out of 2891 genes, 8.0% | 915 out of 15382 genes, 5.9% | 0.00401 |  |
| regulation of cell communication | 641 out of 2891 genes, 22.2% | 2912 out of 15382 genes, 18.9% | 0.00432 |  |
| vesicle organization | 90 out of 2891 genes, 3.1% | 295 out of 15382 genes, 1.9% | 0.00451 |  |
| cellular macromolecule catabolic process | 197 out of 2891 genes, 6.8% | 766 out of 15382 genes, 5.0% | 0.00513 |  |
| transmembrane receptor protein tyrosine kinase signaling pathway | 125 out of 2891 genes, 4.3% | 445 out of 15382 genes, 2.9% | 0.00541 |  |
| regulation of signaling | 649 out of 2891 genes, 22.4% | 2960 out of 15382 genes, 19.2% | 0.00646 |  |
| macromolecule catabolic process | 224 out of 2891 genes, 7.7% | 894 out of 15382 genes, 5.8% | 0.00729 |  |
| enzyme linked receptor protein signaling pathway | 168 out of 2891 genes, 5.8% | 638 out of 15382 genes, 4.1% | 0.00730 |  |
| symbiosis, encompassing mutualism through parasitism | 153 out of 2891 genes, 5.3% | 571 out of 15382 genes, 3.7% | 0.00747 |  |
| cellular response to DNA damage stimulus | 177 out of 2891 genes, 6.1% | 679 out of 15382 genes, 4.4% | 0.00757 |  |
| positive regulation of transcription, DNA-templated | 291 out of 2891 genes, 10.1% | 1210 out of 15382 genes, 7.9% | 0.00842 |  |
| positive regulation of nucleic acid-templated transcription | 291 out of 2891 genes, 10.1% | 1210 out of 15382 genes, 7.9% | 0.00842 |  |
| cellular component assembly | 482 out of 2891 genes, 16.7% | 2135 out of 15382 genes, 13.9% | 0.00856 |  |
| positive regulation of RNA biosynthetic process | 291 out of 2891 genes, 10.1% | 1211 out of 15382 genes, 7.9% | 0.00908 |  |
| regulation of cellular protein metabolic process | 496 out of 2891 genes, 17.2% | 2207 out of 15382 genes, 14.3% | 0.01015 |  |
| nucleic acid metabolic process | 730 out of 2891 genes, 25.3% | 3379 out of 15382 genes, 22.0% | 0.01092 |  |
| regulation of Rho protein signal transduction | 44 out of 2891 genes, 1.5% | 118 out of 15382 genes, 0.8% | 0.01177 |  |
| regulation of localization | 515 out of 2891 genes, 17.8% | 2304 out of 15382 genes, 15.0% | 0.01203 |  |
| negative regulation of cellular component organization | 147 out of 2891 genes, 5.1% | 549 out of 15382 genes, 3.6% | 0.01266 |  |
| regulation of nucleic acid-templated transcription | 688 out of 2891 genes, 23.8% | 3170 out of 15382 genes, 20.6% | 0.01269 |  |
| post-Golgi vesicle-mediated transport | 33 out of 2891 genes, 1.1% | 79 out of 15382 genes, 0.5% | 0.01271 |  |
| regulation of protein localization to cell periphery | 33 out of 2891 genes, 1.1% | 79 out of 15382 genes, 0.5% | 0.01271 |  |
| hematopoietic or lymphoid organ development | 144 out of 2891 genes, 5.0% | 536 out of 15382 genes, 3.5% | 0.01318 |  |
| regulation of transcription, DNA-templated | 687 out of 2891 genes, 23.8% | 3167 out of 15382 genes, 20.6% | 0.01398 |  |
| in utero embryonic development | 88 out of 2891 genes, 3.0% | 294 out of 15382 genes, 1.9% | 0.01444 |  |
| hemopoiesis | 133 out of 2891 genes, 4.6% | 489 out of 15382 genes, 3.2% | 0.01617 |  |
| nucleobase-containing compound metabolic process | 828 out of 2891 genes, 28.6% | 3886 out of 15382 genes, 25.3% | 0.01623 |  |
| phosphorylation | 225 out of 2891 genes, 7.8% | 908 out of 15382 genes, 5.9% | 0.01630 |  |
| modification-dependent macromolecule catabolic process | 126 out of 2891 genes, 4.4% | 459 out of 15382 genes, 3.0% | 0.01789 |  |
| negative regulation of protein metabolic process | 220 out of 2891 genes, 7.6% | 886 out of 15382 genes, 5.8% | 0.01816 |  |
| regulation of RNA biosynthetic process | 688 out of 2891 genes, 23.8% | 3178 out of 15382 genes, 20.7% | 0.01850 |  |
| membrane docking | 53 out of 2891 genes, 1.8% | 154 out of 15382 genes, 1.0% | 0.01912 |  |
| negative regulation of gene expression | 331 out of 2891 genes, 11.4% | 1413 out of 15382 genes, 9.2% | 0.01958 |  |
| regulation of protein localization to membrane | 48 out of 2891 genes, 1.7% | 135 out of 15382 genes, 0.9% | 0.01972 |  |
| positive regulation of catabolic process | 109 out of 2891 genes, 3.8% | 387 out of 15382 genes, 2.5% | 0.02357 |  |
| regulation of cellular protein localization | 139 out of 2891 genes, 4.8% | 519 out of 15382 genes, 3.4% | 0.02370 |  |
| regulation of protein localization to plasma membrane | 30 out of 2891 genes, 1.0% | 71 out of 15382 genes, 0.5% | 0.02778 |  |
| regulation of transcription from RNA polymerase II promoter | 380 out of 2891 genes, 13.1% | 1657 out of 15382 genes, 10.8% | 0.03024 |  |
| protein modification by small protein conjugation or removal | 206 out of 2891 genes, 7.1% | 827 out of 15382 genes, 5.4% | 0.03045 |  |
| regulation of biological process | 1988 out of 2891 genes, 68.8% | 10034 out of 15382 genes, 65.2% | 0.03071 |  |
| positive regulation of neuron projection development | 68 out of 2891 genes, 2.4% | 216 out of 15382 genes, 1.4% | 0.03089 |  |
| endoplasmic reticulum organization | 21 out of 2891 genes, 0.7% | 42 out of 15382 genes, 0.3% | 0.03135 |  |
| membrane disassembly | 21 out of 2891 genes, 0.7% | 42 out of 15382 genes, 0.3% | 0.03135 |  |
| nuclear envelope disassembly | 21 out of 2891 genes, 0.7% | 42 out of 15382 genes, 0.3% | 0.03135 |  |
| mitotic nuclear envelope disassembly | 20 out of 2891 genes, 0.7% | 39 out of 15382 genes, 0.3% | 0.03200 |  |
| modification-dependent protein catabolic process | 123 out of 2891 genes, 4.3% | 451 out of 15382 genes, 2.9% | 0.03320 |  |
| protein methylation | 39 out of 2891 genes, 1.3% | 104 out of 15382 genes, 0.7% | 0.03807 |  |
| protein alkylation | 39 out of 2891 genes, 1.3% | 104 out of 15382 genes, 0.7% | 0.03807 |  |
| gene silencing | 47 out of 2891 genes, 1.6% | 134 out of 15382 genes, 0.9% | 0.03810 |  |
| negative regulation of cellular macromolecule biosynthetic process | 276 out of 2891 genes, 9.5% | 1160 out of 15382 genes, 7.5% | 0.03996 |  |
| positive regulation of macromolecule biosynthetic process | 334 out of 2891 genes, 11.6% | 1439 out of 15382 genes, 9.4% | 0.04167 |  |
| positive regulation of gene expression | 360 out of 2891 genes, 12.5% | 1565 out of 15382 genes, 10.2% | 0.04172 |  |
| establishment or maintenance of cell polarity | 50 out of 2891 genes, 1.7% | 146 out of 15382 genes, 0.9% | 0.04223 |  |
| organelle localization by membrane tethering | 50 out of 2891 genes, 1.7% | 146 out of 15382 genes, 0.9% | 0.04223 |  |
| regulation of cellular component biogenesis | 192 out of 2891 genes, 6.6% | 766 out of 15382 genes, 5.0% | 0.04259 |  |
| negative regulation of cellular protein metabolic process | 206 out of 2891 genes, 7.1% | 831 out of 15382 genes, 5.4% | 0.04275 |  |
| immune system development | 147 out of 2891 genes, 5.1% | 561 out of 15382 genes, 3.6% | 0.04556 |  |
| heterocycle metabolic process | 856 out of 2891 genes, 29.6% | 4054 out of 15382 genes, 26.4% | 0.04608 |  |
| transport along microtubule | 42 out of 2891 genes, 1.5% | 116 out of 15382 genes, 0.8% | 0.04762 |  |
| regulation of histone modification | 42 out of 2891 genes, 1.5% | 116 out of 15382 genes, 0.8% | 0.04762 |  |
| microtubule-based transport | 42 out of 2891 genes, 1.5% | 116 out of 15382 genes, 0.8% | 0.04762 |  |
| positive regulation of dendrite development | 26 out of 2891 genes, 0.9% | 59 out of 15382 genes, 0.4% | 0.04767 |  |
| mitotic cell cycle process | 154 out of 2891 genes, 5.3% | 594 out of 15382 genes, 3.9% | 0.05210 |  |
| negative regulation of organelle organization | 85 out of 2891 genes, 2.9% | 290 out of 15382 genes, 1.9% | 0.05286 |  |
| nucleobase-containing compound biosynthetic process | 541 out of 2891 genes, 18.7% | 2462 out of 15382 genes, 16.0% | 0.05349 |  |
| positive regulation of cell projection organization | 86 out of 2891 genes, 3.0% | 295 out of 15382 genes, 1.9% | 0.05900 |  |
| regulation of cellular component size | 86 out of 2891 genes, 3.0% | 295 out of 15382 genes, 1.9% | 0.05900 |  |
| cell division | 125 out of 2891 genes, 4.3% | 466 out of 15382 genes, 3.0% | 0.06697 |  |
| negative regulation of macromolecule biosynthetic process | 283 out of 2891 genes, 9.8% | 1201 out of 15382 genes, 7.8% | 0.06775 |  |
| DNA metabolic process | 179 out of 2891 genes, 6.2% | 712 out of 15382 genes, 4.6% | 0.07267 |  |
| ubiquitin-dependent protein catabolic process | 120 out of 2891 genes, 4.2% | 445 out of 15382 genes, 2.9% | 0.07682 |  |
| organelle transport along microtubule | 25 out of 2891 genes, 0.9% | 57 out of 15382 genes, 0.4% | 0.07879 |  |
| negative regulation of intracellular signal transduction | 117 out of 2891 genes, 4.0% | 432 out of 15382 genes, 2.8% | 0.07947 |  |
| negative regulation of chromatin organization | 21 out of 2891 genes, 0.7% | 44 out of 15382 genes, 0.3% | 0.07949 |  |
| vesicle docking | 24 out of 2891 genes, 0.8% | 54 out of 15382 genes, 0.4% | 0.08917 |  |
| protein modification by small protein conjugation | 163 out of 2891 genes, 5.6% | 641 out of 15382 genes, 4.2% | 0.09204 |  |
| positive regulation of cellular biosynthetic process | 351 out of 2891 genes, 12.1% | 1535 out of 15382 genes, 10.0% | 0.09604 |  |
| actin filament organization | 60 out of 2891 genes, 2.1% | 190 out of 15382 genes, 1.2% | 0.09841 |  |
| regulation of neuron projection development | 106 out of 2891 genes, 3.7% | 386 out of 15382 genes, 2.5% | 0.10671 |  |
| positive regulation of cellular catabolic process | 95 out of 2891 genes, 3.3% | 338 out of 15382 genes, 2.2% | 0.10687 |  |
| protein phosphorylation | 183 out of 2891 genes, 6.3% | 735 out of 15382 genes, 4.8% | 0.10741 |  |
| peptidyl-lysine modification | 72 out of 2891 genes, 2.5% | 240 out of 15382 genes, 1.6% | 0.10755 |  |
| positive regulation of transcription from RNA polymerase II promoter | 218 out of 2891 genes, 7.5% | 899 out of 15382 genes, 5.8% | 0.11061 |  |
| posttranscriptional gene silencing | 22 out of 2891 genes, 0.8% | 48 out of 15382 genes, 0.3% | 0.11149 |  |
| phosphatidylinositol biosynthetic process | 37 out of 2891 genes, 1.3% | 101 out of 15382 genes, 0.7% | 0.12078 |  |
| regulation of protein localization | 225 out of 2891 genes, 7.8% | 935 out of 15382 genes, 6.1% | 0.13851 |  |
| regulation of intracellular signal transduction | 372 out of 2891 genes, 12.9% | 1644 out of 15382 genes, 10.7% | 0.13947 |  |
| regulation of autophagy | 78 out of 2891 genes, 2.7% | 267 out of 15382 genes, 1.7% | 0.13947 |  |
| cell cycle process | 228 out of 2891 genes, 7.9% | 950 out of 15382 genes, 6.2% | 0.14702 |  |
| heterocycle biosynthetic process | 549 out of 2891 genes, 19.0% | 2524 out of 15382 genes, 16.4% | 0.15313 |  |
| positive regulation of protein localization to membrane | 34 out of 2891 genes, 1.2% | 91 out of 15382 genes, 0.6% | 0.16160 |  |
| histone lysine methylation | 22 out of 2891 genes, 0.8% | 49 out of 15382 genes, 0.3% | 0.16661 |  |
| response to nitrogen compound | 216 out of 2891 genes, 7.5% | 895 out of 15382 genes, 5.8% | 0.16722 |  |
| aromatic compound biosynthetic process | 549 out of 2891 genes, 19.0% | 2527 out of 15382 genes, 16.4% | 0.17588 |  |
| regulation of vesicle-mediated transport | 112 out of 2891 genes, 3.9% | 418 out of 15382 genes, 2.7% | 0.20057 |  |
| chordate embryonic development | 134 out of 2891 genes, 4.6% | 517 out of 15382 genes, 3.4% | 0.20287 |  |
| clathrin-dependent endocytosis | 13 out of 2891 genes, 0.4% | 22 out of 15382 genes, 0.1% | 0.21160 |  |
| regulation of supramolecular fiber organization | 78 out of 2891 genes, 2.7% | 271 out of 15382 genes, 1.8% | 0.24595 |  |
| cell surface receptor signaling pathway | 425 out of 2891 genes, 14.7% | 1916 out of 15382 genes, 12.5% | 0.25064 |  |
| supramolecular fiber organization | 98 out of 2891 genes, 3.4% | 358 out of 15382 genes, 2.3% | 0.25137 |  |
| nuclear envelope organization | 30 out of 2891 genes, 1.0% | 78 out of 15382 genes, 0.5% | 0.25208 |  |
| positive regulation of dendrite morphogenesis | 17 out of 2891 genes, 0.6% | 34 out of 15382 genes, 0.2% | 0.25258 |  |
| posttranscriptional gene silencing by RNA | 21 out of 2891 genes, 0.7% | 47 out of 15382 genes, 0.3% | 0.27715 |  |
| positive regulation of protein localization to cell periphery | 21 out of 2891 genes, 0.7% | 47 out of 15382 genes, 0.3% | 0.27715 |  |
| regulation of actin filament organization | 64 out of 2891 genes, 2.2% | 213 out of 15382 genes, 1.4% | 0.29379 |  |
| cellular nitrogen compound metabolic process | 921 out of 2891 genes, 31.9% | 4437 out of 15382 genes, 28.8% | 0.29559 |  |
| posttranscriptional regulation of gene expression | 123 out of 2891 genes, 4.3% | 471 out of 15382 genes, 3.1% | 0.29633 |  |
| biological regulation | 2075 out of 2891 genes, 71.8% | 10574 out of 15382 genes, 68.7% | 0.30875 |  |
| positive regulation of organelle organization | 140 out of 2891 genes, 4.8% | 549 out of 15382 genes, 3.6% | 0.31793 |  |
| interspecies interaction between organisms | 158 out of 2891 genes, 5.5% | 632 out of 15382 genes, 4.1% | 0.32333 |  |
| positive regulation of biosynthetic process | 353 out of 2891 genes, 12.2% | 1566 out of 15382 genes, 10.2% | 0.32435 |  |
| embryo development ending in birth or egg hatching | 134 out of 2891 genes, 4.6% | 522 out of 15382 genes, 3.4% | 0.33012 |  |
| cellular aromatic compound metabolic process | 853 out of 2891 genes, 29.5% | 4090 out of 15382 genes, 26.6% | 0.33863 |  |
| regulation of establishment or maintenance of cell polarity | 12 out of 2891 genes, 0.4% | 20 out of 15382 genes, 0.1% | 0.34930 |  |
| response to organonitrogen compound | 191 out of 2891 genes, 6.6% | 787 out of 15382 genes, 5.1% | 0.35208 |  |
| apoptotic signaling pathway | 75 out of 2891 genes, 2.6% | 261 out of 15382 genes, 1.7% | 0.36447 |  |
| positive regulation of cell morphogenesis involved in differentiation | 42 out of 2891 genes, 1.5% | 125 out of 15382 genes, 0.8% | 0.37289 |  |
| ER to Golgi vesicle-mediated transport | 51 out of 2891 genes, 1.8% | 161 out of 15382 genes, 1.0% | 0.37996 |  |
| protein localization to nucleus | 50 out of 2891 genes, 1.7% | 157 out of 15382 genes, 1.0% | 0.38323 |  |
| cellular catabolic process | 343 out of 2891 genes, 11.9% | 1521 out of 15382 genes, 9.9% | 0.40497 |  |
| immune response-regulating cell surface receptor signaling pathway | 79 out of 2891 genes, 2.7% | 279 out of 15382 genes, 1.8% | 0.40635 |  |
| cellular component biogenesis | 510 out of 2891 genes, 17.6% | 2350 out of 15382 genes, 15.3% | 0.41121 |  |
| regulation of histone methylation | 23 out of 2891 genes, 0.8% | 55 out of 15382 genes, 0.4% | 0.43645 |  |
| regulation of protein polymerization | 52 out of 2891 genes, 1.8% | 166 out of 15382 genes, 1.1% | 0.44885 |  |
| regulation of molecular function | 484 out of 2891 genes, 16.7% | 2222 out of 15382 genes, 14.4% | 0.45487 |  |
| phosphatidylinositol metabolic process | 46 out of 2891 genes, 1.6% | 142 out of 15382 genes, 0.9% | 0.46989 |  |
| regulation of cell cycle | 241 out of 2891 genes, 8.3% | 1030 out of 15382 genes, 6.7% | 0.51574 |  |
| negative regulation of biosynthetic process | 297 out of 2891 genes, 10.3% | 1301 out of 15382 genes, 8.5% | 0.52720 |  |
| RNA metabolic process | 618 out of 2891 genes, 21.4% | 2902 out of 15382 genes, 18.9% | 0.56146 |  |
| negative regulation of signal transduction | 243 out of 2891 genes, 8.4% | 1041 out of 15382 genes, 6.8% | 0.56512 |  |
| macromolecule methylation | 57 out of 2891 genes, 2.0% | 188 out of 15382 genes, 1.2% | 0.56924 |  |
| regulation of establishment of cell polarity | 11 out of 2891 genes, 0.4% | 18 out of 15382 genes, 0.1% | 0.57445 |  |
| protein K48-linked ubiquitination | 18 out of 2891 genes, 0.6% | 39 out of 15382 genes, 0.3% | 0.57760 |  |
| negative regulation of small GTPase mediated signal transduction | 21 out of 2891 genes, 0.7% | 49 out of 15382 genes, 0.3% | 0.58554 |  |
| autophagy | 66 out of 2891 genes, 2.3% | 226 out of 15382 genes, 1.5% | 0.58674 |  |
| process utilizing autophagic mechanism | 66 out of 2891 genes, 2.3% | 226 out of 15382 genes, 1.5% | 0.58674 |  |
| regulation of dendritic spine development | 23 out of 2891 genes, 0.8% | 56 out of 15382 genes, 0.4% | 0.60996 |  |
| cellular response to nitrogen compound | 129 out of 2891 genes, 4.5% | 506 out of 15382 genes, 3.3% | 0.62972 |  |
| protein dephosphorylation | 37 out of 2891 genes, 1.3% | 108 out of 15382 genes, 0.7% | 0.63670 |  |
| transcription from RNA polymerase II promoter | 116 out of 2891 genes, 4.0% | 447 out of 15382 genes, 2.9% | 0.64044 |  |
| endoplasmic reticulum tubular network organization | 9 out of 2891 genes, 0.3% | 13 out of 15382 genes, 0.1% | 0.65348 |  |
| regulation of gene silencing | 32 out of 2891 genes, 1.1% | 89 out of 15382 genes, 0.6% | 0.65551 |  |
| regulation of cellular component movement | 189 out of 2891 genes, 6.5% | 786 out of 15382 genes, 5.1% | 0.66556 |  |
| entry into host cell | 16 out of 2891 genes, 0.6% | 33 out of 15382 genes, 0.2% | 0.68460 |  |
| entry into host | 16 out of 2891 genes, 0.6% | 33 out of 15382 genes, 0.2% | 0.68460 |  |
| entry into cell of other organism involved in symbiotic interaction | 16 out of 2891 genes, 0.6% | 33 out of 15382 genes, 0.2% | 0.68460 |  |
| entry into other organism involved in symbiotic interaction | 16 out of 2891 genes, 0.6% | 33 out of 15382 genes, 0.2% | 0.68460 |  |
| regulation of anatomical structure morphogenesis | 206 out of 2891 genes, 7.1% | 867 out of 15382 genes, 5.6% | 0.68614 |  |
| regulation of developmental process | 476 out of 2891 genes, 16.5% | 2191 out of 15382 genes, 14.2% | 0.68693 |  |
| apoptotic process | 198 out of 2891 genes, 6.8% | 830 out of 15382 genes, 5.4% | 0.73656 |  |
| organic cyclic compound metabolic process | 891 out of 2891 genes, 30.8% | 4308 out of 15382 genes, 28.0% | 0.74009 |  |
| viral genome replication | 14 out of 2891 genes, 0.5% | 27 out of 15382 genes, 0.2% | 0.74019 |  |
| lysosomal transport | 30 out of 2891 genes, 1.0% | 82 out of 15382 genes, 0.5% | 0.74370 |  |
| regulation of translation | 94 out of 2891 genes, 3.3% | 350 out of 15382 genes, 2.3% | 0.77022 |  |
| regulation of locomotion | 189 out of 2891 genes, 6.5% | 788 out of 15382 genes, 5.1% | 0.77126 |  |
| vacuolar transport | 36 out of 2891 genes, 1.2% | 105 out of 15382 genes, 0.7% | 0.77222 |  |
| regulation of homeostatic process | 110 out of 2891 genes, 3.8% | 422 out of 15382 genes, 2.7% | 0.79404 |  |
| regulation of apoptotic signaling pathway | 93 out of 2891 genes, 3.2% | 346 out of 15382 genes, 2.2% | 0.80951 |  |
| transport of virus | 21 out of 2891 genes, 0.7% | 50 out of 15382 genes, 0.3% | 0.83212 |  |
| organic cyclic compound biosynthetic process | 567 out of 2891 genes, 19.6% | 2654 out of 15382 genes, 17.3% | 0.85327 |  |
| gene silencing by miRNA | 18 out of 2891 genes, 0.6% | 40 out of 15382 genes, 0.3% | 0.86678 |  |
| negative regulation of cellular biosynthetic process | 291 out of 2891 genes, 10.1% | 1281 out of 15382 genes, 8.3% | 0.89817 |  |
| transcription, DNA-templated | 467 out of 2891 genes, 16.2% | 2152 out of 15382 genes, 14.0% | 0.90235 |  |
| DNA damage response, signal transduction by p53 class mediator | 26 out of 2891 genes, 0.9% | 68 out of 15382 genes, 0.4% | 0.90878 |  |
| nucleic acid-templated transcription | 467 out of 2891 genes, 16.2% | 2153 out of 15382 genes, 14.0% | 0.94359 |  |
| peptidyl-lysine methylation | 24 out of 2891 genes, 0.8% | 61 out of 15382 genes, 0.4% | 0.96768 |  |
| regulation of macroautophagy | 46 out of 2891 genes, 1.6% | 146 out of 15382 genes, 0.9% | 0.98597 |  |
| gene expression | 670 out of 2891 genes, 23.2% | 3181 out of 15382 genes, 20.7% | 0.99072 |  |
| protein ubiquitination involved in ubiquitin-dependent protein catabolic process | 41 out of 2891 genes, 1.4% | 126 out of 15382 genes, 0.8% | 1 |  |
| RNA biosynthetic process | 468 out of 2891 genes, 16.2% | 2160 out of 15382 genes, 14.0% | 1 |  |
| protein localization to organelle | 131 out of 2891 genes, 4.5% | 521 out of 15382 genes, 3.4% | 1 |  |
| positive regulation of chromatin organization | 28 out of 2891 genes, 1.0% | 76 out of 15382 genes, 0.5% | 1 |  |
| positive regulation of cell differentiation | 189 out of 2891 genes, 6.5% | 793 out of 15382 genes, 5.2% | 1 |  |
| establishment of mitochondrion localization | 11 out of 2891 genes, 0.4% | 19 out of 15382 genes, 0.1% | 1 |  |
| positive regulation of programmed cell death | 138 out of 2891 genes, 4.8% | 554 out of 15382 genes, 3.6% | 1 |  |
| mitotic G1 DNA damage checkpoint | 23 out of 2891 genes, 0.8% | 58 out of 15382 genes, 0.4% | 1 |  |
| mitotic G1/S transition checkpoint | 23 out of 2891 genes, 0.8% | 58 out of 15382 genes, 0.4% | 1 |  |
| regulation of cellular response to insulin stimulus | 23 out of 2891 genes, 0.8% | 58 out of 15382 genes, 0.4% | 1 |  |
| cortical cytoskeleton organization | 15 out of 2891 genes, 0.5% | 31 out of 15382 genes, 0.2% | 1 |  |
| regulation of actin polymerization or depolymerization | 47 out of 2891 genes, 1.6% | 151 out of 15382 genes, 1.0% | 1 |  |
| phosphatidylinositol phosphorylation | 14 out of 2891 genes, 0.5% | 28 out of 15382 genes, 0.2% | 1 |  |
| negative regulation of RNA metabolic process | 245 out of 2891 genes, 8.5% | 1063 out of 15382 genes, 6.9% | 1 |  |
| negative regulation of nucleobase-containing compound metabolic process | 276 out of 2891 genes, 9.5% | 1214 out of 15382 genes, 7.9% | 1 |  |
| positive regulation of protein localization to plasma membrane | 18 out of 2891 genes, 0.6% | 41 out of 15382 genes, 0.3% | 1 |  |
| regulation of cell motility | 174 out of 2891 genes, 6.0% | 724 out of 15382 genes, 4.7% | 1 |  |
| regulation of cellular response to transforming growth factor beta stimulus | 31 out of 2891 genes, 1.1% | 88 out of 15382 genes, 0.6% | 1 |  |
| regulation of actin filament-based process | 81 out of 2891 genes, 2.8% | 297 out of 15382 genes, 1.9% | 1 |  |
| positive regulation of apoptotic process | 137 out of 2891 genes, 4.7% | 551 out of 15382 genes, 3.6% | 1 |  |
| intracellular transport of virus | 20 out of 2891 genes, 0.7% | 48 out of 15382 genes, 0.3% | 1 |  |
| histone H3-K36 demethylation | 7 out of 2891 genes, 0.2% | 9 out of 15382 genes, 0.1% | 1 |  |
| regulation of hydrolase activity | 189 out of 2891 genes, 6.5% | 796 out of 15382 genes, 5.2% | 1 |  |
| regulation of actin filament length | 47 out of 2891 genes, 1.6% | 152 out of 15382 genes, 1.0% | 1 |  |
| regulation of cytoskeleton organization | 107 out of 2891 genes, 3.7% | 414 out of 15382 genes, 2.7% | 1 |  |
| microtubule-based process | 134 out of 2891 genes, 4.6% | 538 out of 15382 genes, 3.5% | 1 |  |
| positive regulation of molecular function | 301 out of 2891 genes, 10.4% | 1338 out of 15382 genes, 8.7% | 1 |  |
| glycerophospholipid metabolic process | 74 out of 2891 genes, 2.6% | 267 out of 15382 genes, 1.7% | 1 |  |
| regulation of cell migration | 162 out of 2891 genes, 5.6% | 669 out of 15382 genes, 4.3% | 1 |  |
| macroautophagy | 42 out of 2891 genes, 1.5% | 132 out of 15382 genes, 0.9% | 1 |  |
| regulation of dendrite development | 39 out of 2891 genes, 1.3% | 120 out of 15382 genes, 0.8% | 1 |  |
| proteasomal protein catabolic process | 78 out of 2891 genes, 2.7% | 285 out of 15382 genes, 1.9% | 1 |  |
| nuclear pore organization | 9 out of 2891 genes, 0.3% | 14 out of 15382 genes, 0.1% | 1 |  |
| DNA repair | 114 out of 2891 genes, 3.9% | 447 out of 15382 genes, 2.9% | 1 |  |
| G1 DNA damage checkpoint | 23 out of 2891 genes, 0.8% | 59 out of 15382 genes, 0.4% | 1 |  |
| negative regulation of protein modification process | 132 out of 2891 genes, 4.6% | 530 out of 15382 genes, 3.4% | 1 |  |
| gene silencing by RNA | 26 out of 2891 genes, 0.9% | 70 out of 15382 genes, 0.5% | 1 |  |
| positive regulation of histone modification | 26 out of 2891 genes, 0.9% | 70 out of 15382 genes, 0.5% | 1 |  |
| lipid phosphorylation | 16 out of 2891 genes, 0.6% | 35 out of 15382 genes, 0.2% | 1 |  |
| peptidyl-serine phosphorylation | 46 out of 2891 genes, 1.6% | 149 out of 15382 genes, 1.0% | 1 |  |
| ERBB signaling pathway | 24 out of 2891 genes, 0.8% | 63 out of 15382 genes, 0.4% | 1 |  |
| regulation of neurogenesis | 155 out of 2891 genes, 5.4% | 639 out of 15382 genes, 4.2% | 1 |  |
| negative regulation of signaling | 258 out of 2891 genes, 8.9% | 1133 out of 15382 genes, 7.4% | 1 |  |
| cargo loading into vesicle | 10 out of 2891 genes, 0.3% | 17 out of 15382 genes, 0.1% | 1 |  |
| glycerolipid metabolic process | 89 out of 2891 genes, 3.1% | 336 out of 15382 genes, 2.2% | 1 |  |
| immune response-regulating signaling pathway | 95 out of 2891 genes, 3.3% | 363 out of 15382 genes, 2.4% | 1 |  |
| regulation of transforming growth factor beta receptor signaling pathway | 30 out of 2891 genes, 1.0% | 86 out of 15382 genes, 0.6% | 1 |  |
| regulation of cell adhesion | 150 out of 2891 genes, 5.2% | 617 out of 15382 genes, 4.0% | 1 |  |
| regulation of multicellular organismal development | 367 out of 2891 genes, 12.7% | 1671 out of 15382 genes, 10.9% | 1 |  |
| methylation | 59 out of 2891 genes, 2.0% | 205 out of 15382 genes, 1.3% | 1 |  |
| cellular component morphogenesis | 165 out of 2891 genes, 5.7% | 688 out of 15382 genes, 4.5% | 1 |  |
| negative regulation of cell communication | 257 out of 2891 genes, 8.9% | 1130 out of 15382 genes, 7.3% | 1 |  |
| Fc receptor mediated stimulatory signaling pathway | 23 out of 2891 genes, 0.8% | 60 out of 15382 genes, 0.4% | 1 |  |
| cellular response to insulin stimulus | 43 out of 2891 genes, 1.5% | 138 out of 15382 genes, 0.9% | 1 |  |
| catabolic process | 389 out of 2891 genes, 13.5% | 1781 out of 15382 genes, 11.6% | 1 |  |
| vesicle-mediated transport between endosomal compartments | 12 out of 2891 genes, 0.4% | 23 out of 15382 genes, 0.1% | 1 |  |
| retrograde vesicle-mediated transport, Golgi to ER | 27 out of 2891 genes, 0.9% | 75 out of 15382 genes, 0.5% | 1 |  |
| regulation of cell morphogenesis involved in differentiation | 67 out of 2891 genes, 2.3% | 240 out of 15382 genes, 1.6% | 1 |  |
| protein ubiquitination | 138 out of 2891 genes, 4.8% | 562 out of 15382 genes, 3.7% | 1 |  |
| signal transduction in response to DNA damage | 28 out of 2891 genes, 1.0% | 79 out of 15382 genes, 0.5% | 1 |  |
| Fc receptor signaling pathway | 46 out of 2891 genes, 1.6% | 151 out of 15382 genes, 1.0% | 1 |  |
| negative regulation of transcription, DNA-templated | 224 out of 2891 genes, 7.7% | 972 out of 15382 genes, 6.3% | 1 |  |
| regulation of protein complex assembly | 93 out of 2891 genes, 3.2% | 356 out of 15382 genes, 2.3% | 1 |  |
| vesicle docking involved in exocytosis | 16 out of 2891 genes, 0.6% | 36 out of 15382 genes, 0.2% | 1 |  |
| negative regulation of histone modification | 16 out of 2891 genes, 0.6% | 36 out of 15382 genes, 0.2% | 1 |  |
| regulation of organelle assembly | 44 out of 2891 genes, 1.5% | 143 out of 15382 genes, 0.9% | 1 |  |
| regulation of DNA biosynthetic process | 30 out of 2891 genes, 1.0% | 87 out of 15382 genes, 0.6% | 1 |  |
| immune response-regulating cell surface receptor signaling pathway involved in phagocytosis | 22 out of 2891 genes, 0.8% | 57 out of 15382 genes, 0.4% | 1 |  |
| Fc-gamma receptor signaling pathway involved in phagocytosis | 22 out of 2891 genes, 0.8% | 57 out of 15382 genes, 0.4% | 1 |  |
| microtubule cytoskeleton organization | 94 out of 2891 genes, 3.3% | 361 out of 15382 genes, 2.3% | 1 |  |
| nucleus organization | 42 out of 2891 genes, 1.5% | 135 out of 15382 genes, 0.9% | 1 |  |
| regulation of actin cytoskeleton organization | 72 out of 2891 genes, 2.5% | 263 out of 15382 genes, 1.7% | 1 |  |
| cellular response to organonitrogen compound | 108 out of 2891 genes, 3.7% | 425 out of 15382 genes, 2.8% | 1 |  |
| protein autophosphorylation | 53 out of 2891 genes, 1.8% | 181 out of 15382 genes, 1.2% | 1 |  |
| negative regulation of Ras protein signal transduction | 18 out of 2891 genes, 0.6% | 43 out of 15382 genes, 0.3% | 1 |  |
| regulation of epithelial cell migration | 47 out of 2891 genes, 1.6% | 156 out of 15382 genes, 1.0% | 1 |  |
| positive regulation of dendritic spine development | 15 out of 2891 genes, 0.5% | 33 out of 15382 genes, 0.2% | 1 |  |
| regulation of protein catabolic process | 90 out of 2891 genes, 3.1% | 344 out of 15382 genes, 2.2% | 1 |  |
| protein autoubiquitination | 21 out of 2891 genes, 0.7% | 54 out of 15382 genes, 0.4% | 1 |  |
| chromatin remodeling | 41 out of 2891 genes, 1.4% | 132 out of 15382 genes, 0.9% | 1 |  |
| regulation of DNA metabolic process | 90 out of 2891 genes, 3.1% | 345 out of 15382 genes, 2.2% | 1 |  |
| cell morphogenesis | 145 out of 2891 genes, 5.0% | 599 out of 15382 genes, 3.9% | 1 |  |
| dephosphorylation | 46 out of 2891 genes, 1.6% | 153 out of 15382 genes, 1.0% | 1 |  |
| protein import into nucleus | 34 out of 2891 genes, 1.2% | 104 out of 15382 genes, 0.7% | 1 |  |
| positive regulation of cell death | 144 out of 2891 genes, 5.0% | 595 out of 15382 genes, 3.9% | 1 |  |
| regulation of cell development | 176 out of 2891 genes, 6.1% | 747 out of 15382 genes, 4.9% | 1 |  |
| regulation of cellular amide metabolic process | 99 out of 2891 genes, 3.4% | 387 out of 15382 genes, 2.5% | 1 |  |
| organonitrogen compound catabolic process | 209 out of 2891 genes, 7.2% | 906 out of 15382 genes, 5.9% | 1 |  |
| negative regulation of transcription from RNA polymerase II promoter | 155 out of 2891 genes, 5.4% | 648 out of 15382 genes, 4.2% | 1 |  |
| mitochondrion localization | 16 out of 2891 genes, 0.6% | 37 out of 15382 genes, 0.2% | 1 |  |
| platelet-derived growth factor receptor signaling pathway | 12 out of 2891 genes, 0.4% | 24 out of 15382 genes, 0.2% | 1 |  |
| histone lysine demethylation | 12 out of 2891 genes, 0.4% | 24 out of 15382 genes, 0.2% | 1 |  |
| platelet formation | 10 out of 2891 genes, 0.3% | 18 out of 15382 genes, 0.1% | 1 |  |
| negative regulation of histone methylation | 10 out of 2891 genes, 0.3% | 18 out of 15382 genes, 0.1% | 1 |  |
| signal transduction involved in mitotic cell cycle checkpoint | 20 out of 2891 genes, 0.7% | 51 out of 15382 genes, 0.3% | 1 |  |
| signal transduction involved in mitotic G1 DNA damage checkpoint | 20 out of 2891 genes, 0.7% | 51 out of 15382 genes, 0.3% | 1 |  |
| intracellular signal transduction involved in G1 DNA damage checkpoint | 20 out of 2891 genes, 0.7% | 51 out of 15382 genes, 0.3% | 1 |  |
| signal transduction involved in mitotic DNA damage checkpoint | 20 out of 2891 genes, 0.7% | 51 out of 15382 genes, 0.3% | 1 |  |
| signal transduction involved in mitotic DNA integrity checkpoint | 20 out of 2891 genes, 0.7% | 51 out of 15382 genes, 0.3% | 1 |  |
| multi-organism transport | 23 out of 2891 genes, 0.8% | 62 out of 15382 genes, 0.4% | 1 |  |
| multi-organism localization | 23 out of 2891 genes, 0.8% | 62 out of 15382 genes, 0.4% | 1 |  |
| regulation of actin filament polymerization | 41 out of 2891 genes, 1.4% | 133 out of 15382 genes, 0.9% | 1 |  |
| early endosome to late endosome transport | 11 out of 2891 genes, 0.4% | 21 out of 15382 genes, 0.1% | 1 |  |
| microtubule-based movement | 64 out of 2891 genes, 2.2% | 231 out of 15382 genes, 1.5% | 1 |  |
| protein polyubiquitination | 69 out of 2891 genes, 2.4% | 253 out of 15382 genes, 1.6% | 1 |  |
| regulation of cell-matrix adhesion | 29 out of 2891 genes, 1.0% | 85 out of 15382 genes, 0.6% | 1 |  |
| response to organic substance | 537 out of 2891 genes, 18.6% | 2540 out of 15382 genes, 16.5% | 1 |  |
| negative regulation of phosphorylation | 95 out of 2891 genes, 3.3% | 370 out of 15382 genes, 2.4% | 1 |  |
| positive regulation of protein polymerization | 31 out of 2891 genes, 1.1% | 93 out of 15382 genes, 0.6% | 1 |  |
| localization within membrane | 31 out of 2891 genes, 1.1% | 93 out of 15382 genes, 0.6% | 1 |  |
| establishment of organelle localization | 91 out of 2891 genes, 3.1% | 352 out of 15382 genes, 2.3% | 1 |  |
| actin cytoskeleton reorganization | 21 out of 2891 genes, 0.7% | 55 out of 15382 genes, 0.4% | 1 |  |
| nucleocytoplasmic transport | 67 out of 2891 genes, 2.3% | 245 out of 15382 genes, 1.6% | 1 |  |
| Golgi vesicle budding | 25 out of 2891 genes, 0.9% | 70 out of 15382 genes, 0.5% | 1 |  |
| regulation of mitotic cell cycle | 141 out of 2891 genes, 4.9% | 584 out of 15382 genes, 3.8% | 1 |  |
| macromolecule biosynthetic process | 656 out of 2891 genes, 22.7% | 3149 out of 15382 genes, 20.5% | 1 |  |
| negative regulation of nucleic acid-templated transcription | 224 out of 2891 genes, 7.7% | 982 out of 15382 genes, 6.4% | 1 |  |
| insulin receptor signaling pathway | 26 out of 2891 genes, 0.9% | 74 out of 15382 genes, 0.5% | 1 |  |
| negative regulation of phosphate metabolic process | 122 out of 2891 genes, 4.2% | 496 out of 15382 genes, 3.2% | 1 |  |
| vesicle budding from membrane | 28 out of 2891 genes, 1.0% | 82 out of 15382 genes, 0.5% | 1 |  |
| regulation of ERBB signaling pathway | 28 out of 2891 genes, 1.0% | 82 out of 15382 genes, 0.5% | 1 |  |
| negative regulation of G1/S transition of mitotic cell cycle | 28 out of 2891 genes, 1.0% | 82 out of 15382 genes, 0.5% | 1 |  |
| negative regulation of cell cycle G1/S phase transition | 29 out of 2891 genes, 1.0% | 86 out of 15382 genes, 0.6% | 1 |  |
| organic substance catabolic process | 330 out of 2891 genes, 11.4% | 1505 out of 15382 genes, 9.8% | 1 |  |
| negative regulation of RNA biosynthetic process | 224 out of 2891 genes, 7.7% | 984 out of 15382 genes, 6.4% | 1 |  |
| negative regulation of phosphorus metabolic process | 122 out of 2891 genes, 4.2% | 497 out of 15382 genes, 3.2% | 1 |  |
| response to endoplasmic reticulum stress | 63 out of 2891 genes, 2.2% | 229 out of 15382 genes, 1.5% | 1 |  |
| regulation of lamellipodium organization | 16 out of 2891 genes, 0.6% | 38 out of 15382 genes, 0.2% | 1 |  |
| nuclear-transcribed mRNA poly(A) tail shortening | 13 out of 2891 genes, 0.4% | 28 out of 15382 genes, 0.2% | 1 |  |
| protein demethylation | 13 out of 2891 genes, 0.4% | 28 out of 15382 genes, 0.2% | 1 |  |
| protein dealkylation | 13 out of 2891 genes, 0.4% | 28 out of 15382 genes, 0.2% | 1 |  |
| cellular macromolecule biosynthetic process | 643 out of 2891 genes, 22.2% | 3090 out of 15382 genes, 20.1% | 1 |  |
| negative regulation of protein phosphorylation | 87 out of 2891 genes, 3.0% | 337 out of 15382 genes, 2.2% | 1 |  |
| regulation of anatomical structure size | 104 out of 2891 genes, 3.6% | 415 out of 15382 genes, 2.7% | 1 |  |
| Fc-gamma receptor signaling pathway | 22 out of 2891 genes, 0.8% | 60 out of 15382 genes, 0.4% | 1 |  |
| histone demethylation | 12 out of 2891 genes, 0.4% | 25 out of 15382 genes, 0.2% | 1 |  |
| establishment of cell polarity | 30 out of 2891 genes, 1.0% | 91 out of 15382 genes, 0.6% | 1 |  |
| small GTPase mediated signal transduction | 67 out of 2891 genes, 2.3% | 248 out of 15382 genes, 1.6% | 1 |  |
| negative regulation of chromosome organization | 32 out of 2891 genes, 1.1% | 99 out of 15382 genes, 0.6% | 1 |  |
| positive regulation of chromosome organization | 41 out of 2891 genes, 1.4% | 136 out of 15382 genes, 0.9% | 1 |  |
| positive regulation of gene expression, epigenetic | 17 out of 2891 genes, 0.6% | 42 out of 15382 genes, 0.3% | 1 |  |
| establishment of mitochondrion localization, microtubule-mediated | 9 out of 2891 genes, 0.3% | 16 out of 15382 genes, 0.1% | 1 |  |
| mitochondrion transport along microtubule | 9 out of 2891 genes, 0.3% | 16 out of 15382 genes, 0.1% | 1 |  |
| endocytic recycling | 11 out of 2891 genes, 0.4% | 22 out of 15382 genes, 0.1% | 1 |  |
| macromolecular complex assembly | 297 out of 2891 genes, 10.3% | 1347 out of 15382 genes, 8.8% | 1 |  |
| platelet morphogenesis | 10 out of 2891 genes, 0.3% | 19 out of 15382 genes, 0.1% | 1 |  |
| neurotrophin signaling pathway | 10 out of 2891 genes, 0.3% | 19 out of 15382 genes, 0.1% | 1 |  |
| regulation of mitochondrion organization | 55 out of 2891 genes, 1.9% | 196 out of 15382 genes, 1.3% | 1 |  |
| DNA damage checkpoint | 39 out of 2891 genes, 1.3% | 128 out of 15382 genes, 0.8% | 1 |  |
| signal transduction involved in DNA integrity checkpoint | 20 out of 2891 genes, 0.7% | 53 out of 15382 genes, 0.3% | 1 |  |
| signal transduction involved in DNA damage checkpoint | 20 out of 2891 genes, 0.7% | 53 out of 15382 genes, 0.3% | 1 |  |
| exocytic process | 20 out of 2891 genes, 0.7% | 53 out of 15382 genes, 0.3% | 1 |  |
| protein localization to plasma membrane | 44 out of 2891 genes, 1.5% | 149 out of 15382 genes, 1.0% | 1 |  |
| regulation of cell differentiation | 334 out of 2891 genes, 11.6% | 1532 out of 15382 genes, 10.0% | 1 |  |
| regulation of myeloid cell differentiation | 54 out of 2891 genes, 1.9% | 192 out of 15382 genes, 1.2% | 1 |  |
| glycerophospholipid biosynthetic process | 54 out of 2891 genes, 1.9% | 192 out of 15382 genes, 1.2% | 1 |  |
| retrograde transport, endosome to Golgi | 25 out of 2891 genes, 0.9% | 72 out of 15382 genes, 0.5% | 1 |  |
| vacuole organization | 35 out of 2891 genes, 1.2% | 112 out of 15382 genes, 0.7% | 1 |  |
| positive regulation of developmental process | 250 out of 2891 genes, 8.6% | 1118 out of 15382 genes, 7.3% | 1 |  |
| positive regulation of mRNA metabolic process | 22 out of 2891 genes, 0.8% | 61 out of 15382 genes, 0.4% | 1 |  |
| peptidyl-serine modification | 48 out of 2891 genes, 1.7% | 167 out of 15382 genes, 1.1% | 1 |  |
| positive regulation of cell development | 106 out of 2891 genes, 3.7% | 428 out of 15382 genes, 2.8% | 1 |  |
| DNA damage response, signal transduction by p53 class mediator resulting in cell cycle arrest | 19 out of 2891 genes, 0.7% | 50 out of 15382 genes, 0.3% | 1 |  |
| glycerolipid biosynthetic process | 56 out of 2891 genes, 1.9% | 202 out of 15382 genes, 1.3% | 1 |  |
| transcription-dependent tethering of RNA polymerase II gene DNA at nuclear periphery | 5 out of 2891 genes, 0.2% | 6 out of 15382 genes, 0.0% | 1 |  |
| regulation of heart rate by chemical signal | 5 out of 2891 genes, 0.2% | 6 out of 15382 genes, 0.0% | 1 |  |
| establishment or maintenance of cytoskeleton polarity | 5 out of 2891 genes, 0.2% | 6 out of 15382 genes, 0.0% | 1 |  |
| pH elevation | 5 out of 2891 genes, 0.2% | 6 out of 15382 genes, 0.0% | 1 |  |
| intracellular pH elevation | 5 out of 2891 genes, 0.2% | 6 out of 15382 genes, 0.0% | 1 |  |
| regulation of metalloendopeptidase activity | 5 out of 2891 genes, 0.2% | 6 out of 15382 genes, 0.0% | 1 |  |
| regulation of posttranscriptional gene silencing | 24 out of 2891 genes, 0.8% | 69 out of 15382 genes, 0.4% | 1 |  |
| regulation of gene silencing by RNA | 24 out of 2891 genes, 0.8% | 69 out of 15382 genes, 0.4% | 1 |  |
| cellular response to nerve growth factor stimulus | 17 out of 2891 genes, 0.6% | 43 out of 15382 genes, 0.3% | 1 |  |
| NADP biosynthetic process | 4 out of 2891 genes, 0.1% | 4 out of 15382 genes, 0.0% | 1 |  |
| oligopeptide transport | 4 out of 2891 genes, 0.1% | 4 out of 15382 genes, 0.0% | 1 |  |
| endoplasmic reticulum tubular network formation | 4 out of 2891 genes, 0.1% | 4 out of 15382 genes, 0.0% | 1 |  |
| regulation of membrane tubulation | 4 out of 2891 genes, 0.1% | 4 out of 15382 genes, 0.0% | 1 |  |
| signal transduction involved in cell cycle checkpoint | 20 out of 2891 genes, 0.7% | 54 out of 15382 genes, 0.4% | 1 |  |
| regulation of catalytic activity | 360 out of 2891 genes, 12.5% | 1668 out of 15382 genes, 10.8% | 1 |  |
| cellular nitrogen compound biosynthetic process | 600 out of 2891 genes, 20.8% | 2884 out of 15382 genes, 18.7% | 1 |  |
| interaction with host | 29 out of 2891 genes, 1.0% | 89 out of 15382 genes, 0.6% | 1 |  |
| positive regulation of neuron differentiation | 76 out of 2891 genes, 2.6% | 292 out of 15382 genes, 1.9% | 1 |  |
| miRNA mediated inhibition of translation | 7 out of 2891 genes, 0.2% | 11 out of 15382 genes, 0.1% | 1 |  |
| negative regulation of translation, ncRNA-mediated | 7 out of 2891 genes, 0.2% | 11 out of 15382 genes, 0.1% | 1 |  |
| regulation of translation, ncRNA-mediated | 7 out of 2891 genes, 0.2% | 11 out of 15382 genes, 0.1% | 1 |  |
| cargo loading into COPII-coated vesicle | 7 out of 2891 genes, 0.2% | 11 out of 15382 genes, 0.1% | 1 |  |
| cellular response to chemical stimulus | 496 out of 2891 genes, 17.2% | 2356 out of 15382 genes, 15.3% | 1 |  |
| immune system process | 471 out of 2891 genes, 16.3% | 2230 out of 15382 genes, 14.5% | 1 |  |
| positive regulation of neurogenesis | 92 out of 2891 genes, 3.2% | 366 out of 15382 genes, 2.4% | 1 |  |
| cellular response to organic substance | 410 out of 2891 genes, 14.2% | 1922 out of 15382 genes, 12.5% | 1 |  |
| regulation of GTPase activity | 72 out of 2891 genes, 2.5% | 275 out of 15382 genes, 1.8% | 1 |  |
| negative regulation of response to stimulus | 290 out of 2891 genes, 10.0% | 1322 out of 15382 genes, 8.6% | 1 |  |
| peptidyl-threonine phosphorylation | 23 out of 2891 genes, 0.8% | 66 out of 15382 genes, 0.4% | 1 |  |
| regulation of gene silencing by miRNA | 23 out of 2891 genes, 0.8% | 66 out of 15382 genes, 0.4% | 1 |  |
| regulation of autophagosome assembly | 14 out of 2891 genes, 0.5% | 33 out of 15382 genes, 0.2% | 1 |  |
| regulation of epidermal growth factor receptor signaling pathway | 24 out of 2891 genes, 0.8% | 70 out of 15382 genes, 0.5% | 1 |  |
| regulation of type I interferon production | 32 out of 2891 genes, 1.1% | 102 out of 15382 genes, 0.7% | 1 |  |
| regulation of response to stress | 273 out of 2891 genes, 9.4% | 1239 out of 15382 genes, 8.1% | 1 |  |
| nucleotide-sugar biosynthetic process | 10 out of 2891 genes, 0.3% | 20 out of 15382 genes, 0.1% | 1 |  |
| regulation of mRNA 3'-end processing | 10 out of 2891 genes, 0.3% | 20 out of 15382 genes, 0.1% | 1 |  |
| clathrin coat assembly | 9 out of 2891 genes, 0.3% | 17 out of 15382 genes, 0.1% | 1 |  |
| regulation of telomere maintenance via telomerase | 17 out of 2891 genes, 0.6% | 44 out of 15382 genes, 0.3% | 1 |  |
| immune response-activating cell surface receptor signaling pathway | 67 out of 2891 genes, 2.3% | 254 out of 15382 genes, 1.7% | 1 |  |
| nucleotide-sugar metabolic process | 13 out of 2891 genes, 0.4% | 30 out of 15382 genes, 0.2% | 1 |  |
| regulation of cell cycle checkpoint | 13 out of 2891 genes, 0.4% | 30 out of 15382 genes, 0.2% | 1 |  |
| erythrocyte differentiation | 21 out of 2891 genes, 0.7% | 59 out of 15382 genes, 0.4% | 1 |  |
| regulation of vacuole organization | 15 out of 2891 genes, 0.5% | 37 out of 15382 genes, 0.2% | 1 |  |
| regulation of erythrocyte differentiation | 15 out of 2891 genes, 0.5% | 37 out of 15382 genes, 0.2% | 1 |  |
| phospholipid metabolic process | 85 out of 2891 genes, 2.9% | 336 out of 15382 genes, 2.2% | 1 |  |
| vesicle-mediated transport to the plasma membrane | 22 out of 2891 genes, 0.8% | 63 out of 15382 genes, 0.4% | 1 |  |
| regulation of dephosphorylation | 38 out of 2891 genes, 1.3% | 128 out of 15382 genes, 0.8% | 1 |  |
| cellular biosynthetic process | 812 out of 2891 genes, 28.1% | 3988 out of 15382 genes, 25.9% | 1 |  |
| nuclear import | 36 out of 2891 genes, 1.2% | 120 out of 15382 genes, 0.8% | 1 |  |
| cortical actin cytoskeleton organization | 12 out of 2891 genes, 0.4% | 27 out of 15382 genes, 0.2% | 1 |  |
| viral entry into host cell | 12 out of 2891 genes, 0.4% | 27 out of 15382 genes, 0.2% | 1 |  |
| regulation of stress-activated protein kinase signaling cascade | 50 out of 2891 genes, 1.7% | 180 out of 15382 genes, 1.2% | 1 |  |
| protein localization to cell periphery | 47 out of 2891 genes, 1.6% | 167 out of 15382 genes, 1.1% | 1 |  |
| DNA integrity checkpoint | 40 out of 2891 genes, 1.4% | 137 out of 15382 genes, 0.9% | 1 |  |
| organic substance biosynthetic process | 828 out of 2891 genes, 28.6% | 4074 out of 15382 genes, 26.5% | 1 |  |
| positive regulation of cellular protein localization | 84 out of 2891 genes, 2.9% | 333 out of 15382 genes, 2.2% | 1 |  |
| neuromuscular junction development | 14 out of 2891 genes, 0.5% | 34 out of 15382 genes, 0.2% | 1 |  |
| response to epidermal growth factor | 14 out of 2891 genes, 0.5% | 34 out of 15382 genes, 0.2% | 1 |  |
| regulation of protein binding | 49 out of 2891 genes, 1.7% | 176 out of 15382 genes, 1.1% | 1 |  |
| vascular endothelial growth factor receptor signaling pathway | 20 out of 2891 genes, 0.7% | 56 out of 15382 genes, 0.4% | 1 |  |
| positive regulation of transcription from RNA polymerase III promoter | 6 out of 2891 genes, 0.2% | 9 out of 15382 genes, 0.1% | 1 |  |
| regulation of histone H4 acetylation | 6 out of 2891 genes, 0.2% | 9 out of 15382 genes, 0.1% | 1 |  |
| regulation of nervous system development | 166 out of 2891 genes, 5.7% | 722 out of 15382 genes, 4.7% | 1 |  |
| epidermal growth factor receptor signaling pathway | 17 out of 2891 genes, 0.6% | 45 out of 15382 genes, 0.3% | 1 |  |
| regulation of adherens junction organization | 17 out of 2891 genes, 0.6% | 45 out of 15382 genes, 0.3% | 1 |  |
| protein complex biogenesis | 222 out of 2891 genes, 7.7% | 995 out of 15382 genes, 6.5% | 1 |  |
| cell cycle phase transition | 66 out of 2891 genes, 2.3% | 252 out of 15382 genes, 1.6% | 1 |  |
| regulation of axon extension | 22 out of 2891 genes, 0.8% | 64 out of 15382 genes, 0.4% | 1 |  |
| erythrocyte homeostasis | 22 out of 2891 genes, 0.8% | 64 out of 15382 genes, 0.4% | 1 |  |
| positive regulation of epithelial cell migration | 30 out of 2891 genes, 1.0% | 96 out of 15382 genes, 0.6% | 1 |  |
| negative regulation of programmed cell death | 176 out of 2891 genes, 6.1% | 771 out of 15382 genes, 5.0% | 1 |  |
| integrin-mediated signaling pathway | 29 out of 2891 genes, 1.0% | 92 out of 15382 genes, 0.6% | 1 |  |
| stress-activated protein kinase signaling cascade | 36 out of 2891 genes, 1.2% | 121 out of 15382 genes, 0.8% | 1 |  |
| cellular response to peptide hormone stimulus | 59 out of 2891 genes, 2.0% | 221 out of 15382 genes, 1.4% | 1 |  |
| peptidyl-threonine modification | 24 out of 2891 genes, 0.8% | 72 out of 15382 genes, 0.5% | 1 |  |
| positive regulation of actin filament polymerization | 24 out of 2891 genes, 0.8% | 72 out of 15382 genes, 0.5% | 1 |  |
| cellular response to oxygen levels | 49 out of 2891 genes, 1.7% | 177 out of 15382 genes, 1.2% | 1 |  |
| vesicle cytoskeletal trafficking | 15 out of 2891 genes, 0.5% | 38 out of 15382 genes, 0.2% | 1 |  |
| plasma membrane bounded cell projection organization | 218 out of 2891 genes, 7.5% | 977 out of 15382 genes, 6.4% | 1 |  |
| signal transduction by p53 class mediator | 34 out of 2891 genes, 1.2% | 113 out of 15382 genes, 0.7% | 1 |  |
| B cell receptor signaling pathway | 13 out of 2891 genes, 0.4% | 31 out of 15382 genes, 0.2% | 1 |  |
| tRNA transport | 13 out of 2891 genes, 0.4% | 31 out of 15382 genes, 0.2% | 1 |  |
| mitotic cell cycle phase transition | 64 out of 2891 genes, 2.2% | 244 out of 15382 genes, 1.6% | 1 |  |
| regulation of histone H3-K9 methylation | 10 out of 2891 genes, 0.3% | 21 out of 15382 genes, 0.1% | 1 |  |
| regulation of neuron differentiation | 123 out of 2891 genes, 4.3% | 518 out of 15382 genes, 3.4% | 1 |  |
| response to oxygen-containing compound | 289 out of 2891 genes, 10.0% | 1329 out of 15382 genes, 8.6% | 1 |  |
| circulatory system development | 175 out of 2891 genes, 6.1% | 768 out of 15382 genes, 5.0% | 1 |  |
| regulation of protein import into nucleus, translocation | 7 out of 2891 genes, 0.2% | 12 out of 15382 genes, 0.1% | 1 |  |
| negative regulation of ERBB signaling pathway | 16 out of 2891 genes, 0.6% | 42 out of 15382 genes, 0.3% | 1 |  |
| protein complex assembly | 221 out of 2891 genes, 7.6% | 994 out of 15382 genes, 6.5% | 1 |  |
| phospholipid biosynthetic process | 60 out of 2891 genes, 2.1% | 227 out of 15382 genes, 1.5% | 1 |  |
| positive regulation of type I interferon production | 21 out of 2891 genes, 0.7% | 61 out of 15382 genes, 0.4% | 1 |  |
| actomyosin structure organization | 29 out of 2891 genes, 1.0% | 93 out of 15382 genes, 0.6% | 1 |  |
| regulation of cell shape | 39 out of 2891 genes, 1.3% | 135 out of 15382 genes, 0.9% | 1 |  |
| positive regulation of endopeptidase activity | 39 out of 2891 genes, 1.3% | 135 out of 15382 genes, 0.9% | 1 |  |
| Golgi to endosome transport | 8 out of 2891 genes, 0.3% | 15 out of 15382 genes, 0.1% | 1 |  |
| cellular response to endogenous stimulus | 216 out of 2891 genes, 7.5% | 970 out of 15382 genes, 6.3% | 1 |  |
| microtubule cytoskeleton organization involved in mitosis | 27 out of 2891 genes, 0.9% | 85 out of 15382 genes, 0.6% | 1 |  |
| ERAD pathway | 25 out of 2891 genes, 0.9% | 77 out of 15382 genes, 0.5% | 1 |  |
| histone H3-K4 methylation | 12 out of 2891 genes, 0.4% | 28 out of 15382 genes, 0.2% | 1 |  |
| proteasome-mediated ubiquitin-dependent protein catabolic process | 67 out of 2891 genes, 2.3% | 259 out of 15382 genes, 1.7% | 1 |  |
| regulation of mRNA metabolic process | 67 out of 2891 genes, 2.3% | 259 out of 15382 genes, 1.7% | 1 |  |
| regulation of hemopoiesis | 92 out of 2891 genes, 3.2% | 374 out of 15382 genes, 2.4% | 1 |  |
| response to nerve growth factor | 17 out of 2891 genes, 0.6% | 46 out of 15382 genes, 0.3% | 1 |  |
| immune response-activating signal transduction | 84 out of 2891 genes, 2.9% | 337 out of 15382 genes, 2.2% | 1 |  |
| regulation of stress-activated MAPK cascade | 49 out of 2891 genes, 1.7% | 179 out of 15382 genes, 1.2% | 1 |  |
| regulation of axonogenesis | 40 out of 2891 genes, 1.4% | 140 out of 15382 genes, 0.9% | 1 |  |
| positive regulation of catalytic activity | 229 out of 2891 genes, 7.9% | 1036 out of 15382 genes, 6.7% | 1 |  |
| Golgi to plasma membrane transport | 15 out of 2891 genes, 0.5% | 39 out of 15382 genes, 0.3% | 1 |  |
| autophagosome organization | 19 out of 2891 genes, 0.7% | 54 out of 15382 genes, 0.4% | 1 |  |
| biosynthetic process | 837 out of 2891 genes, 29.0% | 4139 out of 15382 genes, 26.9% | 1 |  |
| establishment of epithelial cell polarity | 11 out of 2891 genes, 0.4% | 25 out of 15382 genes, 0.2% | 1 |  |
| programmed cell death | 213 out of 2891 genes, 7.4% | 959 out of 15382 genes, 6.2% | 1 |  |
| cellular response to epidermal growth factor stimulus | 13 out of 2891 genes, 0.4% | 32 out of 15382 genes, 0.2% | 1 |  |
| regulation of mRNA processing | 33 out of 2891 genes, 1.1% | 111 out of 15382 genes, 0.7% | 1 |  |
| positive regulation of protein import into nucleus, translocation | 5 out of 2891 genes, 0.2% | 7 out of 15382 genes, 0.0% | 1 |  |
| regulation of toll-like receptor 3 signaling pathway | 5 out of 2891 genes, 0.2% | 7 out of 15382 genes, 0.0% | 1 |  |
| Golgi to lysosome transport | 5 out of 2891 genes, 0.2% | 7 out of 15382 genes, 0.0% | 1 |  |
| regulation of protein modification process | 339 out of 2891 genes, 11.7% | 1587 out of 15382 genes, 10.3% | 1 |  |
| actin polymerization or depolymerization | 16 out of 2891 genes, 0.6% | 43 out of 15382 genes, 0.3% | 1 |  |
| inositol lipid-mediated signaling | 31 out of 2891 genes, 1.1% | 103 out of 15382 genes, 0.7% | 1 |  |
| positive regulation of protein complex assembly | 52 out of 2891 genes, 1.8% | 194 out of 15382 genes, 1.3% | 1 |  |
| response to insulin | 52 out of 2891 genes, 1.8% | 194 out of 15382 genes, 1.3% | 1 |  |
| double-strand break repair via nonhomologous end joining | 17 out of 2891 genes, 0.6% | 47 out of 15382 genes, 0.3% | 1 |  |
| actin filament bundle assembly | 17 out of 2891 genes, 0.6% | 47 out of 15382 genes, 0.3% | 1 |  |
| Fc-epsilon receptor signaling pathway | 30 out of 2891 genes, 1.0% | 99 out of 15382 genes, 0.6% | 1 |  |
| cell death | 219 out of 2891 genes, 7.6% | 991 out of 15382 genes, 6.4% | 1 |  |
| regulation of cardiac muscle hypertrophy | 14 out of 2891 genes, 0.5% | 36 out of 15382 genes, 0.2% | 1 |  |
| positive regulation of mRNA catabolic process | 14 out of 2891 genes, 0.5% | 36 out of 15382 genes, 0.2% | 1 |  |
| cellular response to stimulus | 1114 out of 2891 genes, 38.5% | 5594 out of 15382 genes, 36.4% | 1 |  |
| organophosphate biosynthetic process | 105 out of 2891 genes, 3.6% | 439 out of 15382 genes, 2.9% | 1 |  |
| autophagosome assembly | 18 out of 2891 genes, 0.6% | 51 out of 15382 genes, 0.3% | 1 |  |
| actin filament bundle organization | 18 out of 2891 genes, 0.6% | 51 out of 15382 genes, 0.3% | 1 |  |
| cell projection organization | 221 out of 2891 genes, 7.6% | 1002 out of 15382 genes, 6.5% | 1 |  |
| tRNA export from nucleus | 12 out of 2891 genes, 0.4% | 29 out of 15382 genes, 0.2% | 1 |  |
| membrane protein proteolysis | 12 out of 2891 genes, 0.4% | 29 out of 15382 genes, 0.2% | 1 |  |
| tRNA-containing ribonucleoprotein complex export from nucleus | 12 out of 2891 genes, 0.4% | 29 out of 15382 genes, 0.2% | 1 |  |
| protein monoubiquitination | 19 out of 2891 genes, 0.7% | 55 out of 15382 genes, 0.4% | 1 |  |
| regulation of antigen receptor-mediated signaling pathway | 19 out of 2891 genes, 0.7% | 55 out of 15382 genes, 0.4% | 1 |  |
| negative regulation of apoptotic process | 171 out of 2891 genes, 5.9% | 757 out of 15382 genes, 4.9% | 1 |  |
| cellular response to decreased oxygen levels | 45 out of 2891 genes, 1.6% | 164 out of 15382 genes, 1.1% | 1 |  |
| organelle fusion | 32 out of 2891 genes, 1.1% | 108 out of 15382 genes, 0.7% | 1 |  |
| negative regulation of MAPK cascade | 36 out of 2891 genes, 1.2% | 125 out of 15382 genes, 0.8% | 1 |  |
| regulation of glucose import | 20 out of 2891 genes, 0.7% | 59 out of 15382 genes, 0.4% | 1 |  |
| positive regulation of protein metabolic process | 313 out of 2891 genes, 10.8% | 1461 out of 15382 genes, 9.5% | 1 |  |
| cellular response to oxygen-containing compound | 183 out of 2891 genes, 6.3% | 816 out of 15382 genes, 5.3% | 1 |  |
| response to endogenous stimulus | 269 out of 2891 genes, 9.3% | 1241 out of 15382 genes, 8.1% | 1 |  |
| regulation of extrinsic apoptotic signaling pathway | 39 out of 2891 genes, 1.3% | 138 out of 15382 genes, 0.9% | 1 |  |
| protein sumoylation | 21 out of 2891 genes, 0.7% | 63 out of 15382 genes, 0.4% | 1 |  |
| lipid translocation | 9 out of 2891 genes, 0.3% | 19 out of 15382 genes, 0.1% | 1 |  |
| negative regulation of Rho protein signal transduction | 9 out of 2891 genes, 0.3% | 19 out of 15382 genes, 0.1% | 1 |  |
| regulation of glucose import in response to insulin stimulus | 9 out of 2891 genes, 0.3% | 19 out of 15382 genes, 0.1% | 1 |  |
| regulation of postsynapse organization | 15 out of 2891 genes, 0.5% | 40 out of 15382 genes, 0.3% | 1 |  |
| organelle assembly | 144 out of 2891 genes, 5.0% | 627 out of 15382 genes, 4.1% | 1 |  |
| negative regulation of cellular catabolic process | 51 out of 2891 genes, 1.8% | 191 out of 15382 genes, 1.2% | 1 |  |
| phosphatidylinositol-mediated signaling | 30 out of 2891 genes, 1.0% | 100 out of 15382 genes, 0.7% | 1 |  |
| regulation of cell-substrate adhesion | 46 out of 2891 genes, 1.6% | 169 out of 15382 genes, 1.1% | 1 |  |
| cellular process | 2585 out of 2891 genes, 89.4% | 13535 out of 15382 genes, 88.0% | 1 |  |
| regulation of multicellular organismal process | 535 out of 2891 genes, 18.5% | 2590 out of 15382 genes, 16.8% | 1 |  |
| membrane protein intracellular domain proteolysis | 6 out of 2891 genes, 0.2% | 10 out of 15382 genes, 0.1% | 1 |  |
| negative regulation of DNA damage response, signal transduction by p53 class mediator | 6 out of 2891 genes, 0.2% | 10 out of 15382 genes, 0.1% | 1 |  |
| nuclear pore complex assembly | 6 out of 2891 genes, 0.2% | 10 out of 15382 genes, 0.1% | 1 |  |
| negative regulation of histone H3-K9 methylation | 6 out of 2891 genes, 0.2% | 10 out of 15382 genes, 0.1% | 1 |  |
| regulation of histone H3-K9 trimethylation | 6 out of 2891 genes, 0.2% | 10 out of 15382 genes, 0.1% | 1 |  |
| regulation of chemokine (C-X-C motif) ligand 2 production | 6 out of 2891 genes, 0.2% | 10 out of 15382 genes, 0.1% | 1 |  |
| regulation of lamellipodium morphogenesis | 6 out of 2891 genes, 0.2% | 10 out of 15382 genes, 0.1% | 1 |  |
| regulation of transport | 340 out of 2891 genes, 11.8% | 1599 out of 15382 genes, 10.4% | 1 |  |
| regulation of binding | 74 out of 2891 genes, 2.6% | 296 out of 15382 genes, 1.9% | 1 |  |
| regulation of gene expression by genetic imprinting | 8 out of 2891 genes, 0.3% | 16 out of 15382 genes, 0.1% | 1 |  |
| N-glycan processing | 8 out of 2891 genes, 0.3% | 16 out of 15382 genes, 0.1% | 1 |  |
| negative regulation of cardiac muscle hypertrophy | 8 out of 2891 genes, 0.3% | 16 out of 15382 genes, 0.1% | 1 |  |
| positive regulation of protein deacetylation | 8 out of 2891 genes, 0.3% | 16 out of 15382 genes, 0.1% | 1 |  |
| positive regulation of axon extension | 13 out of 2891 genes, 0.4% | 33 out of 15382 genes, 0.2% | 1 |  |
| response to stress | 623 out of 2891 genes, 21.5% | 3044 out of 15382 genes, 19.8% | 1 |  |
| histone H3-K9 demethylation | 7 out of 2891 genes, 0.2% | 13 out of 15382 genes, 0.1% | 1 |  |
| cytoplasmic mRNA processing body assembly | 7 out of 2891 genes, 0.2% | 13 out of 15382 genes, 0.1% | 1 |  |
| positive regulation of B cell differentiation | 7 out of 2891 genes, 0.2% | 13 out of 15382 genes, 0.1% | 1 |  |
| neurotrophin TRK receptor signaling pathway | 7 out of 2891 genes, 0.2% | 13 out of 15382 genes, 0.1% | 1 |  |
| regulation of cholesterol homeostasis | 7 out of 2891 genes, 0.2% | 13 out of 15382 genes, 0.1% | 1 |  |
| positive regulation of multicellular organismal process | 298 out of 2891 genes, 10.3% | 1390 out of 15382 genes, 9.0% | 1 |  |
| demethylation | 18 out of 2891 genes, 0.6% | 52 out of 15382 genes, 0.3% | 1 |  |
| neural tube development | 38 out of 2891 genes, 1.3% | 135 out of 15382 genes, 0.9% | 1 |  |
| positive regulation of protein binding | 23 out of 2891 genes, 0.8% | 72 out of 15382 genes, 0.5% | 1 |  |
| ephrin receptor signaling pathway | 23 out of 2891 genes, 0.8% | 72 out of 15382 genes, 0.5% | 1 |  |
| translesion synthesis | 14 out of 2891 genes, 0.5% | 37 out of 15382 genes, 0.2% | 1 |  |
| positive regulation of dephosphorylation | 14 out of 2891 genes, 0.5% | 37 out of 15382 genes, 0.2% | 1 |  |
| regulation of cell junction assembly | 22 out of 2891 genes, 0.8% | 68 out of 15382 genes, 0.4% | 1 |  |
| COPII-coated vesicle budding | 21 out of 2891 genes, 0.7% | 64 out of 15382 genes, 0.4% | 1 |  |
| positive regulation of proteolysis | 81 out of 2891 genes, 2.8% | 330 out of 15382 genes, 2.1% | 1 |  |
| receptor-mediated endocytosis | 40 out of 2891 genes, 1.4% | 144 out of 15382 genes, 0.9% | 1 |  |
| histone H3-K36 methylation | 4 out of 2891 genes, 0.1% | 5 out of 15382 genes, 0.0% | 1 |  |
| inositol trisphosphate metabolic process | 4 out of 2891 genes, 0.1% | 5 out of 15382 genes, 0.0% | 1 |  |
| immunoglobulin V(D)J recombination | 4 out of 2891 genes, 0.1% | 5 out of 15382 genes, 0.0% | 1 |  |
| coenzyme transport | 4 out of 2891 genes, 0.1% | 5 out of 15382 genes, 0.0% | 1 |  |
| regulation of membrane depolarization during action potential | 4 out of 2891 genes, 0.1% | 5 out of 15382 genes, 0.0% | 1 |  |
| regulation of keratinocyte apoptotic process | 4 out of 2891 genes, 0.1% | 5 out of 15382 genes, 0.0% | 1 |  |
| regulation of wound healing, spreading of epidermal cells | 4 out of 2891 genes, 0.1% | 5 out of 15382 genes, 0.0% | 1 |  |
| positive regulation of hydrogen peroxide-induced cell death | 4 out of 2891 genes, 0.1% | 5 out of 15382 genes, 0.0% | 1 |  |
| macromolecular complex subunit organization | 340 out of 2891 genes, 11.8% | 1603 out of 15382 genes, 10.4% | 1 |  |
| negative regulation of apoptotic signaling pathway | 49 out of 2891 genes, 1.7% | 184 out of 15382 genes, 1.2% | 1 |  |
| plasma membrane bounded cell projection assembly | 93 out of 2891 genes, 3.2% | 387 out of 15382 genes, 2.5% | 1 |  |
| antigen processing and presentation of peptide antigen via MHC class II | 28 out of 2891 genes, 1.0% | 93 out of 15382 genes, 0.6% | 1 |  |
| negative regulation of cell death | 188 out of 2891 genes, 6.5% | 846 out of 15382 genes, 5.5% | 1 |  |
| regulation of sodium ion transmembrane transporter activity | 15 out of 2891 genes, 0.5% | 41 out of 15382 genes, 0.3% | 1 |  |
| B cell homeostasis | 10 out of 2891 genes, 0.3% | 23 out of 15382 genes, 0.1% | 1 |  |
| mitochondrial calcium ion transmembrane transport | 10 out of 2891 genes, 0.3% | 23 out of 15382 genes, 0.1% | 1 |  |
| phosphatidylinositol 3-kinase signaling | 12 out of 2891 genes, 0.4% | 30 out of 15382 genes, 0.2% | 1 |  |
| protein localization to microtubule cytoskeleton | 12 out of 2891 genes, 0.4% | 30 out of 15382 genes, 0.2% | 1 |  |
| ncRNA export from nucleus | 12 out of 2891 genes, 0.4% | 30 out of 15382 genes, 0.2% | 1 |  |
| regulation of lymphocyte differentiation | 38 out of 2891 genes, 1.3% | 136 out of 15382 genes, 0.9% | 1 |  |
| intrinsic apoptotic signaling pathway | 38 out of 2891 genes, 1.3% | 136 out of 15382 genes, 0.9% | 1 |  |
| response to starvation | 43 out of 2891 genes, 1.5% | 158 out of 15382 genes, 1.0% | 1 |  |
| regulation of response to DNA damage stimulus | 43 out of 2891 genes, 1.5% | 158 out of 15382 genes, 1.0% | 1 |  |
| cellular response to cAMP | 16 out of 2891 genes, 0.6% | 45 out of 15382 genes, 0.3% | 1 |  |
| striated muscle cell differentiation | 47 out of 2891 genes, 1.6% | 176 out of 15382 genes, 1.1% | 1 |  |
| mRNA transport | 37 out of 2891 genes, 1.3% | 132 out of 15382 genes, 0.9% | 1 |  |
| endosome to lysosome transport | 17 out of 2891 genes, 0.6% | 49 out of 15382 genes, 0.3% | 1 |  |
| vesicle targeting | 24 out of 2891 genes, 0.8% | 77 out of 15382 genes, 0.5% | 1 |  |
| non-recombinational repair | 18 out of 2891 genes, 0.6% | 53 out of 15382 genes, 0.3% | 1 |  |
| positive regulation of peptidase activity | 39 out of 2891 genes, 1.3% | 141 out of 15382 genes, 0.9% | 1 |  |
| positive regulation of protein catabolic process | 58 out of 2891 genes, 2.0% | 226 out of 15382 genes, 1.5% | 1 |  |
| intra-Golgi vesicle-mediated transport | 13 out of 2891 genes, 0.4% | 34 out of 15382 genes, 0.2% | 1 |  |
| protein localization to cytoskeleton | 13 out of 2891 genes, 0.4% | 34 out of 15382 genes, 0.2% | 1 |  |
| positive regulation of alpha-beta T cell differentiation | 13 out of 2891 genes, 0.4% | 34 out of 15382 genes, 0.2% | 1 |  |
| regulation of dendritic spine morphogenesis | 13 out of 2891 genes, 0.4% | 34 out of 15382 genes, 0.2% | 1 |  |
| endosome organization | 22 out of 2891 genes, 0.8% | 69 out of 15382 genes, 0.4% | 1 |  |
| protein import | 41 out of 2891 genes, 1.4% | 150 out of 15382 genes, 1.0% | 1 |  |
| protein localization to centrosome | 9 out of 2891 genes, 0.3% | 20 out of 15382 genes, 0.1% | 1 |  |
| regulation of cardiac muscle cell action potential | 9 out of 2891 genes, 0.3% | 20 out of 15382 genes, 0.1% | 1 |  |
| protein localization to microtubule organizing center | 9 out of 2891 genes, 0.3% | 20 out of 15382 genes, 0.1% | 1 |  |
| antigen processing and presentation of peptide or polysaccharide antigen via MHC class II | 28 out of 2891 genes, 1.0% | 94 out of 15382 genes, 0.6% | 1 |  |
| mitotic DNA integrity checkpoint | 28 out of 2891 genes, 1.0% | 94 out of 15382 genes, 0.6% | 1 |  |
| nuclear export | 38 out of 2891 genes, 1.3% | 137 out of 15382 genes, 0.9% | 1 |  |
| posttranscriptional tethering of RNA polymerase II gene DNA at nuclear periphery | 3 out of 2891 genes, 0.1% | 3 out of 15382 genes, 0.0% | 1 |  |
| response to molecule of fungal origin | 3 out of 2891 genes, 0.1% | 3 out of 15382 genes, 0.0% | 1 |  |
| establishment or maintenance of microtubule cytoskeleton polarity | 3 out of 2891 genes, 0.1% | 3 out of 15382 genes, 0.0% | 1 |  |
| positive regulation of gamma-delta T cell differentiation | 3 out of 2891 genes, 0.1% | 3 out of 15382 genes, 0.0% | 1 |  |
| positive regulation of gamma-delta T cell activation | 3 out of 2891 genes, 0.1% | 3 out of 15382 genes, 0.0% | 1 |  |
| regulation of chromosome condensation | 3 out of 2891 genes, 0.1% | 3 out of 15382 genes, 0.0% | 1 |  |
| epithelial cell differentiation involved in embryonic placenta development | 3 out of 2891 genes, 0.1% | 3 out of 15382 genes, 0.0% | 1 |  |
| epithelial cell morphogenesis involved in placental branching | 3 out of 2891 genes, 0.1% | 3 out of 15382 genes, 0.0% | 1 |  |
| positive regulation of Wnt protein secretion | 3 out of 2891 genes, 0.1% | 3 out of 15382 genes, 0.0% | 1 |  |
| ubiquitin-dependent protein catabolic process via the N-end rule pathway | 3 out of 2891 genes, 0.1% | 3 out of 15382 genes, 0.0% | 1 |  |
| positive regulation of hydrogen peroxide-mediated programmed cell death | 3 out of 2891 genes, 0.1% | 3 out of 15382 genes, 0.0% | 1 |  |
| positive regulation of membrane tubulation | 3 out of 2891 genes, 0.1% | 3 out of 15382 genes, 0.0% | 1 |  |
| regulation of modification of synaptic structure | 3 out of 2891 genes, 0.1% | 3 out of 15382 genes, 0.0% | 1 |  |
| malonyl-CoA metabolic process | 3 out of 2891 genes, 0.1% | 3 out of 15382 genes, 0.0% | 1 |  |
| cellular response to starvation | 35 out of 2891 genes, 1.2% | 124 out of 15382 genes, 0.8% | 1 |  |
| negative regulation of macroautophagy | 11 out of 2891 genes, 0.4% | 27 out of 15382 genes, 0.2% | 1 |  |
| peptidyl-lysine trimethylation | 11 out of 2891 genes, 0.4% | 27 out of 15382 genes, 0.2% | 1 |  |
| dsRNA fragmentation | 11 out of 2891 genes, 0.4% | 27 out of 15382 genes, 0.2% | 1 |  |
| production of small RNA involved in gene silencing by RNA | 11 out of 2891 genes, 0.4% | 27 out of 15382 genes, 0.2% | 1 |  |
| regulation of plasma membrane bounded cell projection assembly | 40 out of 2891 genes, 1.4% | 146 out of 15382 genes, 0.9% | 1 |  |
| cellular response to growth factor stimulus | 100 out of 2891 genes, 3.5% | 423 out of 15382 genes, 2.7% | 1 |  |
| regulation of muscle hypertrophy | 14 out of 2891 genes, 0.5% | 38 out of 15382 genes, 0.2% | 1 |  |
| cytoplasmic microtubule organization | 14 out of 2891 genes, 0.5% | 38 out of 15382 genes, 0.2% | 1 |  |
| lamellipodium organization | 14 out of 2891 genes, 0.5% | 38 out of 15382 genes, 0.2% | 1 |  |
| cell growth | 37 out of 2891 genes, 1.3% | 133 out of 15382 genes, 0.9% | 1 |  |
| cell projection assembly | 93 out of 2891 genes, 3.2% | 390 out of 15382 genes, 2.5% | 1 |  |
| negative regulation of cell adhesion | 56 out of 2891 genes, 1.9% | 218 out of 15382 genes, 1.4% | 1 |  |
| cellular component disassembly | 94 out of 2891 genes, 3.3% | 395 out of 15382 genes, 2.6% | 1 |  |
| protein localization to membrane | 84 out of 2891 genes, 2.9% | 348 out of 15382 genes, 2.3% | 1 |  |
| regulation of megakaryocyte differentiation | 15 out of 2891 genes, 0.5% | 42 out of 15382 genes, 0.3% | 1 |  |
| regulation of focal adhesion assembly | 15 out of 2891 genes, 0.5% | 42 out of 15382 genes, 0.3% | 1 |  |
| regulation of cell-substrate junction assembly | 15 out of 2891 genes, 0.5% | 42 out of 15382 genes, 0.3% | 1 |  |
| regulation of phosphatase activity | 29 out of 2891 genes, 1.0% | 99 out of 15382 genes, 0.6% | 1 |  |
| transcytosis | 8 out of 2891 genes, 0.3% | 17 out of 15382 genes, 0.1% | 1 |  |
| 'de novo' posttranslational protein folding | 8 out of 2891 genes, 0.3% | 17 out of 15382 genes, 0.1% | 1 |  |
| positive regulation of apoptotic signaling pathway | 43 out of 2891 genes, 1.5% | 160 out of 15382 genes, 1.0% | 1 |  |
| muscle cell differentiation | 55 out of 2891 genes, 1.9% | 214 out of 15382 genes, 1.4% | 1 |  |
| vesicle localization | 53 out of 2891 genes, 1.8% | 205 out of 15382 genes, 1.3% | 1 |  |
| generation of neurons | 283 out of 2891 genes, 9.8% | 1325 out of 15382 genes, 8.6% | 1 |  |
| regulation of lymphocyte activation | 90 out of 2891 genes, 3.1% | 377 out of 15382 genes, 2.5% | 1 |  |
| regulation of telomere maintenance | 22 out of 2891 genes, 0.8% | 70 out of 15382 genes, 0.5% | 1 |  |
| regulation of protein complex disassembly | 28 out of 2891 genes, 1.0% | 95 out of 15382 genes, 0.6% | 1 |  |
| DNA biosynthetic process | 28 out of 2891 genes, 1.0% | 95 out of 15382 genes, 0.6% | 1 |  |
| lamellipodium assembly | 12 out of 2891 genes, 0.4% | 31 out of 15382 genes, 0.2% | 1 |  |
| osteoclast differentiation | 12 out of 2891 genes, 0.4% | 31 out of 15382 genes, 0.2% | 1 |  |
| negative regulation of epidermal growth factor receptor signaling pathway | 12 out of 2891 genes, 0.4% | 31 out of 15382 genes, 0.2% | 1 |  |
| vesicle transport along microtubule | 12 out of 2891 genes, 0.4% | 31 out of 15382 genes, 0.2% | 1 |  |
| regulation of cell projection assembly | 40 out of 2891 genes, 1.4% | 147 out of 15382 genes, 1.0% | 1 |  |
| nuclear-transcribed mRNA catabolic process, deadenylation-dependent decay | 20 out of 2891 genes, 0.7% | 62 out of 15382 genes, 0.4% | 1 |  |
| negative regulation of autophagy | 19 out of 2891 genes, 0.7% | 58 out of 15382 genes, 0.4% | 1 |  |
| regulation of muscle adaptation | 19 out of 2891 genes, 0.7% | 58 out of 15382 genes, 0.4% | 1 |  |
| mannose metabolic process | 5 out of 2891 genes, 0.2% | 8 out of 15382 genes, 0.1% | 1 |  |
| actin filament depolymerization | 5 out of 2891 genes, 0.2% | 8 out of 15382 genes, 0.1% | 1 |  |
| negative regulation of chromatin silencing | 5 out of 2891 genes, 0.2% | 8 out of 15382 genes, 0.1% | 1 |  |
| positive regulation of mammary gland epithelial cell proliferation | 5 out of 2891 genes, 0.2% | 8 out of 15382 genes, 0.1% | 1 |  |
| mitochondrial protein processing | 5 out of 2891 genes, 0.2% | 8 out of 15382 genes, 0.1% | 1 |  |
| astrocyte activation | 5 out of 2891 genes, 0.2% | 8 out of 15382 genes, 0.1% | 1 |  |
| hair follicle maturation | 5 out of 2891 genes, 0.2% | 8 out of 15382 genes, 0.1% | 1 |  |
| actin filament severing | 5 out of 2891 genes, 0.2% | 8 out of 15382 genes, 0.1% | 1 |  |
| interleukin-6-mediated signaling pathway | 5 out of 2891 genes, 0.2% | 8 out of 15382 genes, 0.1% | 1 |  |
| antigen processing and presentation of exogenous peptide antigen via MHC class II | 27 out of 2891 genes, 0.9% | 91 out of 15382 genes, 0.6% | 1 |  |
| spindle organization | 34 out of 2891 genes, 1.2% | 121 out of 15382 genes, 0.8% | 1 |  |
| Golgi to plasma membrane protein transport | 10 out of 2891 genes, 0.3% | 24 out of 15382 genes, 0.2% | 1 |  |
| centrosome localization | 10 out of 2891 genes, 0.3% | 24 out of 15382 genes, 0.2% | 1 |  |
| neuron projection organization | 10 out of 2891 genes, 0.3% | 24 out of 15382 genes, 0.2% | 1 |  |
| viral RNA genome replication | 7 out of 2891 genes, 0.2% | 14 out of 15382 genes, 0.1% | 1 |  |
| protein transport within plasma membrane | 7 out of 2891 genes, 0.2% | 14 out of 15382 genes, 0.1% | 1 |  |
| neurotransmitter receptor transport | 7 out of 2891 genes, 0.2% | 14 out of 15382 genes, 0.1% | 1 |  |
| regulation of mitophagy | 7 out of 2891 genes, 0.2% | 14 out of 15382 genes, 0.1% | 1 |  |
| mitotic DNA damage checkpoint | 26 out of 2891 genes, 0.9% | 87 out of 15382 genes, 0.6% | 1 |  |
| regulation of cell size | 36 out of 2891 genes, 1.2% | 130 out of 15382 genes, 0.8% | 1 |  |
| cellular macromolecular complex assembly | 162 out of 2891 genes, 5.6% | 726 out of 15382 genes, 4.7% | 1 |  |
| negative regulation of cellular component movement | 60 out of 2891 genes, 2.1% | 238 out of 15382 genes, 1.5% | 1 |  |
| embryo development | 188 out of 2891 genes, 6.5% | 854 out of 15382 genes, 5.6% | 1 |  |
| regulation of RNA splicing | 33 out of 2891 genes, 1.1% | 117 out of 15382 genes, 0.8% | 1 |  |
| establishment of protein localization to plasma membrane | 13 out of 2891 genes, 0.4% | 35 out of 15382 genes, 0.2% | 1 |  |
| regulation of endocytosis | 49 out of 2891 genes, 1.7% | 188 out of 15382 genes, 1.2% | 1 |  |
| regulation of protein stability | 58 out of 2891 genes, 2.0% | 229 out of 15382 genes, 1.5% | 1 |  |
| regulation of vascular associated smooth muscle cell migration | 6 out of 2891 genes, 0.2% | 11 out of 15382 genes, 0.1% | 1 |  |
| positive regulation of hydrolase activity | 111 out of 2891 genes, 3.8% | 479 out of 15382 genes, 3.1% | 1 |  |
| regulation of JNK cascade | 40 out of 2891 genes, 1.4% | 148 out of 15382 genes, 1.0% | 1 |  |
| regulation of leukocyte activation | 102 out of 2891 genes, 3.5% | 436 out of 15382 genes, 2.8% | 1 |  |
| regulation of extent of cell growth | 24 out of 2891 genes, 0.8% | 79 out of 15382 genes, 0.5% | 1 |  |
| negative regulation of catabolic process | 59 out of 2891 genes, 2.0% | 234 out of 15382 genes, 1.5% | 1 |  |
| cellular response to hypoxia | 42 out of 2891 genes, 1.5% | 157 out of 15382 genes, 1.0% | 1 |  |
| regulation of sodium ion transport | 22 out of 2891 genes, 0.8% | 71 out of 15382 genes, 0.5% | 1 |  |
| negative regulation of locomotion | 63 out of 2891 genes, 2.2% | 253 out of 15382 genes, 1.6% | 1 |  |
| regulation of interferon-beta production | 15 out of 2891 genes, 0.5% | 43 out of 15382 genes, 0.3% | 1 |  |
| polyol metabolic process | 27 out of 2891 genes, 0.9% | 92 out of 15382 genes, 0.6% | 1 |  |
| organelle membrane fusion | 27 out of 2891 genes, 0.9% | 92 out of 15382 genes, 0.6% | 1 |  |
| protein deglycosylation | 11 out of 2891 genes, 0.4% | 28 out of 15382 genes, 0.2% | 1 |  |
| regulation of actin cytoskeleton reorganization | 11 out of 2891 genes, 0.4% | 28 out of 15382 genes, 0.2% | 1 |  |
| heart development | 102 out of 2891 genes, 3.5% | 437 out of 15382 genes, 2.8% | 1 |  |
| protein N-linked glycosylation | 21 out of 2891 genes, 0.7% | 67 out of 15382 genes, 0.4% | 1 |  |
| regulation of B cell activation | 30 out of 2891 genes, 1.0% | 105 out of 15382 genes, 0.7% | 1 |  |
| positive regulation of phosphatase activity | 9 out of 2891 genes, 0.3% | 21 out of 15382 genes, 0.1% | 1 |  |
| regulation of B cell receptor signaling pathway | 9 out of 2891 genes, 0.3% | 21 out of 15382 genes, 0.1% | 1 |  |
| regulation of actin nucleation | 9 out of 2891 genes, 0.3% | 21 out of 15382 genes, 0.1% | 1 |  |
| regulation of histone H3-K4 methylation | 9 out of 2891 genes, 0.3% | 21 out of 15382 genes, 0.1% | 1 |  |
| multivesicular body sorting pathway | 9 out of 2891 genes, 0.3% | 21 out of 15382 genes, 0.1% | 1 |  |
| dendritic spine organization | 9 out of 2891 genes, 0.3% | 21 out of 15382 genes, 0.1% | 1 |  |
| receptor localization to synapse | 9 out of 2891 genes, 0.3% | 21 out of 15382 genes, 0.1% | 1 |  |
| neuronal stem cell population maintenance | 9 out of 2891 genes, 0.3% | 21 out of 15382 genes, 0.1% | 1 |  |
| intrinsic apoptotic signaling pathway in response to DNA damage | 19 out of 2891 genes, 0.7% | 59 out of 15382 genes, 0.4% | 1 |  |
| regulation of telomere maintenance via telomere lengthening | 17 out of 2891 genes, 0.6% | 51 out of 15382 genes, 0.3% | 1 |  |
| response to unfolded protein | 38 out of 2891 genes, 1.3% | 140 out of 15382 genes, 0.9% | 1 |  |
| negative regulation of binding | 38 out of 2891 genes, 1.3% | 140 out of 15382 genes, 0.9% | 1 |  |
| negative regulation of extrinsic apoptotic signaling pathway | 26 out of 2891 genes, 0.9% | 88 out of 15382 genes, 0.6% | 1 |  |
| cellular response to oxidative stress | 50 out of 2891 genes, 1.7% | 194 out of 15382 genes, 1.3% | 1 |  |
| establishment of protein localization to organelle | 74 out of 2891 genes, 2.6% | 305 out of 15382 genes, 2.0% | 1 |  |
| cellular response to peptide | 65 out of 2891 genes, 2.2% | 263 out of 15382 genes, 1.7% | 1 |  |
| regulation of cysteine-type endopeptidase activity involved in apoptotic process | 46 out of 2891 genes, 1.6% | 176 out of 15382 genes, 1.1% | 1 |  |
| regulation of cell cycle process | 139 out of 2891 genes, 4.8% | 617 out of 15382 genes, 4.0% | 1 |  |
| regulation of response to cytokine stimulus | 39 out of 2891 genes, 1.3% | 145 out of 15382 genes, 0.9% | 1 |  |
| cellular response to unfolded protein | 31 out of 2891 genes, 1.1% | 110 out of 15382 genes, 0.7% | 1 |  |
| negative regulation of cell migration | 54 out of 2891 genes, 1.9% | 213 out of 15382 genes, 1.4% | 1 |  |
| neural tube closure | 23 out of 2891 genes, 0.8% | 76 out of 15382 genes, 0.5% | 1 |  |
| regulation of dendrite morphogenesis | 23 out of 2891 genes, 0.8% | 76 out of 15382 genes, 0.5% | 1 |  |
| tube closure | 23 out of 2891 genes, 0.8% | 76 out of 15382 genes, 0.5% | 1 |  |
| negative regulation of cell cycle | 113 out of 2891 genes, 3.9% | 492 out of 15382 genes, 3.2% | 1 |  |
| positive regulation of cysteine-type endopeptidase activity | 33 out of 2891 genes, 1.1% | 119 out of 15382 genes, 0.8% | 1 |  |
| response to growth factor | 104 out of 2891 genes, 3.6% | 449 out of 15382 genes, 2.9% | 1 |  |
| spindle localization | 13 out of 2891 genes, 0.4% | 36 out of 15382 genes, 0.2% | 1 |  |
| regulation of signal transduction by p53 class mediator | 40 out of 2891 genes, 1.4% | 150 out of 15382 genes, 1.0% | 1 |  |
| positive regulation of supramolecular fiber organization | 40 out of 2891 genes, 1.4% | 150 out of 15382 genes, 1.0% | 1 |  |
| regulation of glucose transport | 21 out of 2891 genes, 0.7% | 68 out of 15382 genes, 0.4% | 1 |  |
| cellular response to topologically incorrect protein | 35 out of 2891 genes, 1.2% | 128 out of 15382 genes, 0.8% | 1 |  |
| negative regulation of muscle hypertrophy | 8 out of 2891 genes, 0.3% | 18 out of 15382 genes, 0.1% | 1 |  |
| protein exit from endoplasmic reticulum | 8 out of 2891 genes, 0.3% | 18 out of 15382 genes, 0.1% | 1 |  |
| phospholipid translocation | 8 out of 2891 genes, 0.3% | 18 out of 15382 genes, 0.1% | 1 |  |
| positive regulation of dendritic spine morphogenesis | 8 out of 2891 genes, 0.3% | 18 out of 15382 genes, 0.1% | 1 |  |
| negative regulation of intrinsic apoptotic signaling pathway by p53 class mediator | 8 out of 2891 genes, 0.3% | 18 out of 15382 genes, 0.1% | 1 |  |
| mRNA metabolic process | 132 out of 2891 genes, 4.6% | 585 out of 15382 genes, 3.8% | 1 |  |
| neural tube formation | 26 out of 2891 genes, 0.9% | 89 out of 15382 genes, 0.6% | 1 |  |
| regulation of intracellular transport | 80 out of 2891 genes, 2.8% | 335 out of 15382 genes, 2.2% | 1 |  |
| positive regulation of transforming growth factor beta receptor signaling pathway | 10 out of 2891 genes, 0.3% | 25 out of 15382 genes, 0.2% | 1 |  |
| embryonic placenta morphogenesis | 10 out of 2891 genes, 0.3% | 25 out of 15382 genes, 0.2% | 1 |  |
| microtubule organizing center localization | 10 out of 2891 genes, 0.3% | 25 out of 15382 genes, 0.2% | 1 |  |
| positive regulation of cellular response to transforming growth factor beta stimulus | 10 out of 2891 genes, 0.3% | 25 out of 15382 genes, 0.2% | 1 |  |
| positive regulation of cellular protein metabolic process | 291 out of 2891 genes, 10.1% | 1376 out of 15382 genes, 8.9% | 1 |  |
| regulation of cytokine-mediated signaling pathway | 37 out of 2891 genes, 1.3% | 137 out of 15382 genes, 0.9% | 1 |  |
| growth | 89 out of 2891 genes, 3.1% | 378 out of 15382 genes, 2.5% | 1 |  |
| vesicle targeting, rough ER to cis-Golgi | 19 out of 2891 genes, 0.7% | 60 out of 15382 genes, 0.4% | 1 |  |
| COPII vesicle coating | 19 out of 2891 genes, 0.7% | 60 out of 15382 genes, 0.4% | 1 |  |
| phagocytosis | 43 out of 2891 genes, 1.5% | 164 out of 15382 genes, 1.1% | 1 |  |
| regulation of sodium ion transmembrane transport | 16 out of 2891 genes, 0.6% | 48 out of 15382 genes, 0.3% | 1 |  |
| regulation of autophagy of mitochondrion | 16 out of 2891 genes, 0.6% | 48 out of 15382 genes, 0.3% | 1 |  |
| inositol phosphate metabolic process | 17 out of 2891 genes, 0.6% | 52 out of 15382 genes, 0.3% | 1 |  |
| neurogenesis | 299 out of 2891 genes, 10.3% | 1418 out of 15382 genes, 9.2% | 1 |  |
| immune effector process | 185 out of 2891 genes, 6.4% | 847 out of 15382 genes, 5.5% | 1 |  |
| cytokinesis | 24 out of 2891 genes, 0.8% | 81 out of 15382 genes, 0.5% | 1 |  |
| primary neural tube formation | 24 out of 2891 genes, 0.8% | 81 out of 15382 genes, 0.5% | 1 |  |
| response to oxygen levels | 77 out of 2891 genes, 2.7% | 322 out of 15382 genes, 2.1% | 1 |  |
| response to topologically incorrect protein | 42 out of 2891 genes, 1.5% | 160 out of 15382 genes, 1.0% | 1 |  |
| cell morphogenesis involved in differentiation | 110 out of 2891 genes, 3.8% | 480 out of 15382 genes, 3.1% | 1 |  |
| negative regulation of cell-matrix adhesion | 11 out of 2891 genes, 0.4% | 29 out of 15382 genes, 0.2% | 1 |  |
| ER-nucleus signaling pathway | 11 out of 2891 genes, 0.4% | 29 out of 15382 genes, 0.2% | 1 |  |
| positive regulation of histone methylation | 11 out of 2891 genes, 0.4% | 29 out of 15382 genes, 0.2% | 1 |  |
| positive regulation of protein dephosphorylation | 11 out of 2891 genes, 0.4% | 29 out of 15382 genes, 0.2% | 1 |  |
| centrosome duplication | 11 out of 2891 genes, 0.4% | 29 out of 15382 genes, 0.2% | 1 |  |
| negative regulation of cellular response to insulin stimulus | 11 out of 2891 genes, 0.4% | 29 out of 15382 genes, 0.2% | 1 |  |
| regulation of sprouting angiogenesis | 11 out of 2891 genes, 0.4% | 29 out of 15382 genes, 0.2% | 1 |  |
| activation of cysteine-type endopeptidase activity involved in apoptotic process | 23 out of 2891 genes, 0.8% | 77 out of 15382 genes, 0.5% | 1 |  |
| mitochondrial membrane organization | 30 out of 2891 genes, 1.0% | 107 out of 15382 genes, 0.7% | 1 |  |
| regulation of DNA binding transcription factor activity | 82 out of 2891 genes, 2.8% | 346 out of 15382 genes, 2.2% | 1 |  |
| pore complex assembly | 7 out of 2891 genes, 0.2% | 15 out of 15382 genes, 0.1% | 1 |  |
| histone H3-K4 trimethylation | 7 out of 2891 genes, 0.2% | 15 out of 15382 genes, 0.1% | 1 |  |
| regulation of DNA damage checkpoint | 7 out of 2891 genes, 0.2% | 15 out of 15382 genes, 0.1% | 1 |  |
| positive regulation of glucose import in response to insulin stimulus | 7 out of 2891 genes, 0.2% | 15 out of 15382 genes, 0.1% | 1 |  |
| negative regulation of gene expression, epigenetic | 22 out of 2891 genes, 0.8% | 73 out of 15382 genes, 0.5% | 1 |  |
| positive regulation of exocytosis | 22 out of 2891 genes, 0.8% | 73 out of 15382 genes, 0.5% | 1 |  |
| membrane fusion | 41 out of 2891 genes, 1.4% | 156 out of 15382 genes, 1.0% | 1 |  |
| positive regulation of viral process | 26 out of 2891 genes, 0.9% | 90 out of 15382 genes, 0.6% | 1 |  |
| ciliary basal body-plasma membrane docking | 26 out of 2891 genes, 0.9% | 90 out of 15382 genes, 0.6% | 1 |  |
| regulation of establishment of protein localization | 163 out of 2891 genes, 5.6% | 740 out of 15382 genes, 4.8% | 1 |  |
| negative regulation of supramolecular fiber organization | 29 out of 2891 genes, 1.0% | 103 out of 15382 genes, 0.7% | 1 |  |
| exocytosis | 153 out of 2891 genes, 5.3% | 691 out of 15382 genes, 4.5% | 1 |  |
| regulation of endopeptidase activity | 61 out of 2891 genes, 2.1% | 248 out of 15382 genes, 1.6% | 1 |  |
| NLS-bearing protein import into nucleus | 9 out of 2891 genes, 0.3% | 22 out of 15382 genes, 0.1% | 1 |  |
| positive regulation of erythrocyte differentiation | 9 out of 2891 genes, 0.3% | 22 out of 15382 genes, 0.1% | 1 |  |
| positive regulation of cellular response to insulin stimulus | 9 out of 2891 genes, 0.3% | 22 out of 15382 genes, 0.1% | 1 |  |
| positive regulation of toll-like receptor 3 signaling pathway | 4 out of 2891 genes, 0.1% | 6 out of 15382 genes, 0.0% | 1 |  |
| histone-threonine phosphorylation | 4 out of 2891 genes, 0.1% | 6 out of 15382 genes, 0.0% | 1 |  |
| entry of bacterium into host cell | 4 out of 2891 genes, 0.1% | 6 out of 15382 genes, 0.0% | 1 |  |
| aggrephagy | 4 out of 2891 genes, 0.1% | 6 out of 15382 genes, 0.0% | 1 |  |
| chromatin-mediated maintenance of transcription | 4 out of 2891 genes, 0.1% | 6 out of 15382 genes, 0.0% | 1 |  |
| regulation of metaphase plate congression | 4 out of 2891 genes, 0.1% | 6 out of 15382 genes, 0.0% | 1 |  |
| positive regulation of histone H4 acetylation | 4 out of 2891 genes, 0.1% | 6 out of 15382 genes, 0.0% | 1 |  |
| dendritic spine maintenance | 4 out of 2891 genes, 0.1% | 6 out of 15382 genes, 0.0% | 1 |  |
| negative regulation of histone H3-K9 trimethylation | 4 out of 2891 genes, 0.1% | 6 out of 15382 genes, 0.0% | 1 |  |
| positive regulation of response to reactive oxygen species | 4 out of 2891 genes, 0.1% | 6 out of 15382 genes, 0.0% | 1 |  |
| negative regulation of mitophagy | 4 out of 2891 genes, 0.1% | 6 out of 15382 genes, 0.0% | 1 |  |
| regulation of aspartic-type endopeptidase activity involved in amyloid precursor protein catabolic process | 4 out of 2891 genes, 0.1% | 6 out of 15382 genes, 0.0% | 1 |  |
| regulation of aspartic-type peptidase activity | 4 out of 2891 genes, 0.1% | 6 out of 15382 genes, 0.0% | 1 |  |
| regulation of platelet-derived growth factor receptor-beta signaling pathway | 4 out of 2891 genes, 0.1% | 6 out of 15382 genes, 0.0% | 1 |  |
| positive regulation of cellular response to drug | 4 out of 2891 genes, 0.1% | 6 out of 15382 genes, 0.0% | 1 |  |
| vesicle targeting, to, from or within Golgi | 20 out of 2891 genes, 0.7% | 65 out of 15382 genes, 0.4% | 1 |  |
| regulation of symbiosis, encompassing mutualism through parasitism | 51 out of 2891 genes, 1.8% | 202 out of 15382 genes, 1.3% | 1 |  |
| regulation of cysteine-type endopeptidase activity | 51 out of 2891 genes, 1.8% | 202 out of 15382 genes, 1.3% | 1 |  |
| blood vessel development | 103 out of 2891 genes, 3.6% | 448 out of 15382 genes, 2.9% | 1 |  |
| regulation of cellular response to heat | 19 out of 2891 genes, 0.7% | 61 out of 15382 genes, 0.4% | 1 |  |
| response to decreased oxygen levels | 73 out of 2891 genes, 2.5% | 305 out of 15382 genes, 2.0% | 1 |  |
| axo-dendritic transport | 14 out of 2891 genes, 0.5% | 41 out of 15382 genes, 0.3% | 1 |  |
| exit from mitosis | 6 out of 2891 genes, 0.2% | 12 out of 15382 genes, 0.1% | 1 |  |
| positive regulation of histone H3-K4 methylation | 6 out of 2891 genes, 0.2% | 12 out of 15382 genes, 0.1% | 1 |  |
| positive regulation of sodium ion transmembrane transporter activity | 6 out of 2891 genes, 0.2% | 12 out of 15382 genes, 0.1% | 1 |  |
| positive regulation of autophagy | 27 out of 2891 genes, 0.9% | 95 out of 15382 genes, 0.6% | 1 |  |
| cellular response to alcohol | 17 out of 2891 genes, 0.6% | 53 out of 15382 genes, 0.3% | 1 |  |
| response to peptide hormone | 78 out of 2891 genes, 2.7% | 329 out of 15382 genes, 2.1% | 1 |  |
| postreplication repair | 15 out of 2891 genes, 0.5% | 45 out of 15382 genes, 0.3% | 1 |  |
| labyrinthine layer development | 15 out of 2891 genes, 0.5% | 45 out of 15382 genes, 0.3% | 1 |  |
| negative regulation of molecular function | 192 out of 2891 genes, 6.6% | 886 out of 15382 genes, 5.8% | 1 |  |
| regulation of biological quality | 667 out of 2891 genes, 23.1% | 3314 out of 15382 genes, 21.5% | 1 |  |
| regulation of cellular response to growth factor stimulus | 53 out of 2891 genes, 1.8% | 212 out of 15382 genes, 1.4% | 1 |  |
| cell part morphogenesis | 103 out of 2891 genes, 3.6% | 449 out of 15382 genes, 2.9% | 1 |  |
| positive regulation of nervous system development | 98 out of 2891 genes, 3.4% | 425 out of 15382 genes, 2.8% | 1 |  |
| anatomical structure morphogenesis | 399 out of 2891 genes, 13.8% | 1933 out of 15382 genes, 12.6% | 1 |  |
| regulation of immune system process | 275 out of 2891 genes, 9.5% | 1303 out of 15382 genes, 8.5% | 1 |  |
| negative regulation of translation | 34 out of 2891 genes, 1.2% | 126 out of 15382 genes, 0.8% | 1 |  |
| negative regulation of mRNA 3'-end processing | 5 out of 2891 genes, 0.2% | 9 out of 15382 genes, 0.1% | 1 |  |
| regulation of histone ubiquitination | 5 out of 2891 genes, 0.2% | 9 out of 15382 genes, 0.1% | 1 |  |
| SNARE complex assembly | 5 out of 2891 genes, 0.2% | 9 out of 15382 genes, 0.1% | 1 |  |
| response to leucine | 5 out of 2891 genes, 0.2% | 9 out of 15382 genes, 0.1% | 1 |  |
| regulation of hydrogen peroxide-mediated programmed cell death | 5 out of 2891 genes, 0.2% | 9 out of 15382 genes, 0.1% | 1 |  |
| positive regulation of vascular associated smooth muscle cell migration | 5 out of 2891 genes, 0.2% | 9 out of 15382 genes, 0.1% | 1 |  |
| negative regulation of lymphocyte migration | 5 out of 2891 genes, 0.2% | 9 out of 15382 genes, 0.1% | 1 |  |
| regulation of receptor catabolic process | 5 out of 2891 genes, 0.2% | 9 out of 15382 genes, 0.1% | 1 |  |
| multicellular organism growth | 22 out of 2891 genes, 0.8% | 74 out of 15382 genes, 0.5% | 1 |  |
| positive regulation of lymphocyte differentiation | 22 out of 2891 genes, 0.8% | 74 out of 15382 genes, 0.5% | 1 |  |
| regulation of cellular response to drug | 10 out of 2891 genes, 0.3% | 26 out of 15382 genes, 0.2% | 1 |  |
| regulation of peptidyl-serine phosphorylation | 31 out of 2891 genes, 1.1% | 113 out of 15382 genes, 0.7% | 1 |  |
| positive regulation of cell cycle arrest | 21 out of 2891 genes, 0.7% | 70 out of 15382 genes, 0.5% | 1 |  |
| vesicle fusion | 25 out of 2891 genes, 0.9% | 87 out of 15382 genes, 0.6% | 1 |  |
| negative regulation of protein binding | 25 out of 2891 genes, 0.9% | 87 out of 15382 genes, 0.6% | 1 |  |
| response to cAMP | 25 out of 2891 genes, 0.9% | 87 out of 15382 genes, 0.6% | 1 |  |
| mRNA export from nucleus | 28 out of 2891 genes, 1.0% | 100 out of 15382 genes, 0.7% | 1 |  |
| mRNA-containing ribonucleoprotein complex export from nucleus | 28 out of 2891 genes, 1.0% | 100 out of 15382 genes, 0.7% | 1 |  |
| lipid modification | 41 out of 2891 genes, 1.4% | 158 out of 15382 genes, 1.0% | 1 |  |
| TOR signaling | 8 out of 2891 genes, 0.3% | 19 out of 15382 genes, 0.1% | 1 |  |
| selective autophagy | 8 out of 2891 genes, 0.3% | 19 out of 15382 genes, 0.1% | 1 |  |
| negative regulation of cell motility | 56 out of 2891 genes, 1.9% | 227 out of 15382 genes, 1.5% | 1 |  |
| mitotic spindle organization | 20 out of 2891 genes, 0.7% | 66 out of 15382 genes, 0.4% | 1 |  |
| epithelial cell morphogenesis | 11 out of 2891 genes, 0.4% | 30 out of 15382 genes, 0.2% | 1 |  |
| peroxisome organization | 11 out of 2891 genes, 0.4% | 30 out of 15382 genes, 0.2% | 1 |  |
| RNA polyadenylation | 11 out of 2891 genes, 0.4% | 30 out of 15382 genes, 0.2% | 1 |  |
| vesicle coating | 19 out of 2891 genes, 0.7% | 62 out of 15382 genes, 0.4% | 1 |  |
| negative regulation of dephosphorylation | 23 out of 2891 genes, 0.8% | 79 out of 15382 genes, 0.5% | 1 |  |
| regulation of phosphorylation | 279 out of 2891 genes, 9.7% | 1327 out of 15382 genes, 8.6% | 1 |  |
| endoplasmic reticulum unfolded protein response | 29 out of 2891 genes, 1.0% | 105 out of 15382 genes, 0.7% | 1 |  |
| citrate metabolic process | 12 out of 2891 genes, 0.4% | 34 out of 15382 genes, 0.2% | 1 |  |
| embryonic digit morphogenesis | 17 out of 2891 genes, 0.6% | 54 out of 15382 genes, 0.4% | 1 |  |
| positive regulation of DNA biosynthetic process | 17 out of 2891 genes, 0.6% | 54 out of 15382 genes, 0.4% | 1 |  |
| cardiovascular system development | 109 out of 2891 genes, 3.8% | 481 out of 15382 genes, 3.1% | 1 |  |
| response to glucagon | 13 out of 2891 genes, 0.4% | 38 out of 15382 genes, 0.2% | 1 |  |
| positive regulation of response to stimulus | 383 out of 2891 genes, 13.2% | 1857 out of 15382 genes, 12.1% | 1 |  |
| cell cycle G2/M phase transition | 33 out of 2891 genes, 1.1% | 123 out of 15382 genes, 0.8% | 1 |  |
| regulation of protein transport | 155 out of 2891 genes, 5.4% | 707 out of 15382 genes, 4.6% | 1 |  |
| myeloid cell development | 15 out of 2891 genes, 0.5% | 46 out of 15382 genes, 0.3% | 1 |  |
| positive regulation of hemopoiesis | 39 out of 2891 genes, 1.3% | 150 out of 15382 genes, 1.0% | 1 |  |
| maintenance of location | 35 out of 2891 genes, 1.2% | 132 out of 15382 genes, 0.9% | 1 |  |
| activation of protein kinase activity | 63 out of 2891 genes, 2.2% | 261 out of 15382 genes, 1.7% | 1 |  |
| positive regulation of cell migration | 88 out of 2891 genes, 3.0% | 380 out of 15382 genes, 2.5% | 1 |  |
| positive regulation of cellular component movement | 94 out of 2891 genes, 3.3% | 409 out of 15382 genes, 2.7% | 1 |  |
| cellular response to organic cyclic compound | 94 out of 2891 genes, 3.3% | 409 out of 15382 genes, 2.7% | 1 |  |
| developmental growth | 75 out of 2891 genes, 2.6% | 318 out of 15382 genes, 2.1% | 1 |  |
| regulation of cell cycle phase transition | 89 out of 2891 genes, 3.1% | 385 out of 15382 genes, 2.5% | 1 |  |
| regulation of viral process | 44 out of 2891 genes, 1.5% | 173 out of 15382 genes, 1.1% | 1 |  |
| positive regulation of cysteine-type endopeptidase activity involved in apoptotic process | 30 out of 2891 genes, 1.0% | 110 out of 15382 genes, 0.7% | 1 |  |
| regulation of T cell differentiation | 30 out of 2891 genes, 1.0% | 110 out of 15382 genes, 0.7% | 1 |  |
| protein complex subunit organization | 254 out of 2891 genes, 8.8% | 1203 out of 15382 genes, 7.8% | 1 |  |
| regulation of hydrogen peroxide-induced cell death | 9 out of 2891 genes, 0.3% | 23 out of 15382 genes, 0.1% | 1 |  |
| negative regulation of cellular amide metabolic process | 36 out of 2891 genes, 1.2% | 137 out of 15382 genes, 0.9% | 1 |  |
| DNA synthesis involved in DNA repair | 20 out of 2891 genes, 0.7% | 67 out of 15382 genes, 0.4% | 1 |  |
| positive regulation of axonogenesis | 20 out of 2891 genes, 0.7% | 67 out of 15382 genes, 0.4% | 1 |  |
| regulation of stress fiber assembly | 20 out of 2891 genes, 0.7% | 67 out of 15382 genes, 0.4% | 1 |  |
| smooth muscle tissue development | 7 out of 2891 genes, 0.2% | 16 out of 15382 genes, 0.1% | 1 |  |
| myeloid cell homeostasis | 23 out of 2891 genes, 0.8% | 80 out of 15382 genes, 0.5% | 1 |  |
| movement of cell or subcellular component | 275 out of 2891 genes, 9.5% | 1311 out of 15382 genes, 8.5% | 1 |  |
| positive regulation of cellular component biogenesis | 97 out of 2891 genes, 3.4% | 425 out of 15382 genes, 2.8% | 1 |  |
| regulation of fibroblast migration | 10 out of 2891 genes, 0.3% | 27 out of 15382 genes, 0.2% | 1 |  |
| negative regulation of insulin receptor signaling pathway | 10 out of 2891 genes, 0.3% | 27 out of 15382 genes, 0.2% | 1 |  |
| establishment of protein localization to vacuole | 10 out of 2891 genes, 0.3% | 27 out of 15382 genes, 0.2% | 1 |  |
| blood coagulation | 62 out of 2891 genes, 2.1% | 258 out of 15382 genes, 1.7% | 1 |  |
| mRNA processing | 95 out of 2891 genes, 3.3% | 416 out of 15382 genes, 2.7% | 1 |  |
| regulation of immunoglobulin production | 17 out of 2891 genes, 0.6% | 55 out of 15382 genes, 0.4% | 1 |  |
| regulation of protein dephosphorylation | 17 out of 2891 genes, 0.6% | 55 out of 15382 genes, 0.4% | 1 |  |
| histone deacetylation | 11 out of 2891 genes, 0.4% | 31 out of 15382 genes, 0.2% | 1 |  |
| adherens junction assembly | 11 out of 2891 genes, 0.4% | 31 out of 15382 genes, 0.2% | 1 |  |
| establishment of spindle localization | 11 out of 2891 genes, 0.4% | 31 out of 15382 genes, 0.2% | 1 |  |
| regulation of protein deacetylation | 11 out of 2891 genes, 0.4% | 31 out of 15382 genes, 0.2% | 1 |  |
| regulation of response to reactive oxygen species | 11 out of 2891 genes, 0.4% | 31 out of 15382 genes, 0.2% | 1 |  |
| establishment of vesicle localization | 48 out of 2891 genes, 1.7% | 193 out of 15382 genes, 1.3% | 1 |  |
| regulation of cell activation | 105 out of 2891 genes, 3.6% | 465 out of 15382 genes, 3.0% | 1 |  |
| regulation of actomyosin structure organization | 21 out of 2891 genes, 0.7% | 72 out of 15382 genes, 0.5% | 1 |  |
| cellular response to dsRNA | 15 out of 2891 genes, 0.5% | 47 out of 15382 genes, 0.3% | 1 |  |
| regulation of membrane depolarization | 12 out of 2891 genes, 0.4% | 35 out of 15382 genes, 0.2% | 1 |  |
| negative regulation of protein kinase B signaling | 12 out of 2891 genes, 0.4% | 35 out of 15382 genes, 0.2% | 1 |  |
| protein localization to vacuole | 12 out of 2891 genes, 0.4% | 35 out of 15382 genes, 0.2% | 1 |  |
| regulation of mitotic cell cycle phase transition | 83 out of 2891 genes, 2.9% | 359 out of 15382 genes, 2.3% | 1 |  |
| regulation of histone acetylation | 13 out of 2891 genes, 0.4% | 39 out of 15382 genes, 0.3% | 1 |  |
| regulation of telomerase activity | 13 out of 2891 genes, 0.4% | 39 out of 15382 genes, 0.3% | 1 |  |
| signal transduction | 835 out of 2891 genes, 28.9% | 4208 out of 15382 genes, 27.4% | 1 |  |
| regulation of nucleocytoplasmic transport | 44 out of 2891 genes, 1.5% | 175 out of 15382 genes, 1.1% | 1 |  |
| response to hypoxia | 70 out of 2891 genes, 2.4% | 297 out of 15382 genes, 1.9% | 1 |  |
| Ras protein signal transduction | 37 out of 2891 genes, 1.3% | 143 out of 15382 genes, 0.9% | 1 |  |
| striated muscle cell development | 31 out of 2891 genes, 1.1% | 116 out of 15382 genes, 0.8% | 1 |  |
| N-acetylglucosamine metabolic process | 6 out of 2891 genes, 0.2% | 13 out of 15382 genes, 0.1% | 1 |  |
| Golgi to vacuole transport | 6 out of 2891 genes, 0.2% | 13 out of 15382 genes, 0.1% | 1 |  |
| regulation of cardiac muscle contraction by regulation of the release of sequestered calcium ion | 6 out of 2891 genes, 0.2% | 13 out of 15382 genes, 0.1% | 1 |  |
| positive regulation of histone deacetylation | 6 out of 2891 genes, 0.2% | 13 out of 15382 genes, 0.1% | 1 |  |
| positive regulation of phosphoprotein phosphatase activity | 6 out of 2891 genes, 0.2% | 13 out of 15382 genes, 0.1% | 1 |  |
| cardiac muscle cell proliferation | 6 out of 2891 genes, 0.2% | 13 out of 15382 genes, 0.1% | 1 |  |
| negative regulation of neural precursor cell proliferation | 6 out of 2891 genes, 0.2% | 13 out of 15382 genes, 0.1% | 1 |  |
| regulation of clathrin-dependent endocytosis | 6 out of 2891 genes, 0.2% | 13 out of 15382 genes, 0.1% | 1 |  |
| negative regulation of anoikis | 6 out of 2891 genes, 0.2% | 13 out of 15382 genes, 0.1% | 1 |  |
| activation of immune response | 91 out of 2891 genes, 3.1% | 398 out of 15382 genes, 2.6% | 1 |  |
| regulation of transcription from RNA polymerase III promoter | 8 out of 2891 genes, 0.3% | 20 out of 15382 genes, 0.1% | 1 |  |
| regulation of release of sequestered calcium ion into cytosol by sarcoplasmic reticulum | 8 out of 2891 genes, 0.3% | 20 out of 15382 genes, 0.1% | 1 |  |
| regulation of chromatin silencing | 8 out of 2891 genes, 0.3% | 20 out of 15382 genes, 0.1% | 1 |  |
| phosphatidylinositol dephosphorylation | 8 out of 2891 genes, 0.3% | 20 out of 15382 genes, 0.1% | 1 |  |
| labyrinthine layer morphogenesis | 8 out of 2891 genes, 0.3% | 20 out of 15382 genes, 0.1% | 1 |  |
| mRNA destabilization | 8 out of 2891 genes, 0.3% | 20 out of 15382 genes, 0.1% | 1 |  |
| bone cell development | 8 out of 2891 genes, 0.3% | 20 out of 15382 genes, 0.1% | 1 |  |
| regulation of protein insertion into mitochondrial membrane involved in apoptotic signaling pathway | 8 out of 2891 genes, 0.3% | 20 out of 15382 genes, 0.1% | 1 |  |
| positive regulation of protein insertion into mitochondrial membrane involved in apoptotic signaling pathway | 8 out of 2891 genes, 0.3% | 20 out of 15382 genes, 0.1% | 1 |  |
| positive regulation of transcription from RNA polymerase II promoter involved in cellular response to chemical stimulus | 8 out of 2891 genes, 0.3% | 20 out of 15382 genes, 0.1% | 1 |  |
| positive regulation of locomotion | 98 out of 2891 genes, 3.4% | 432 out of 15382 genes, 2.8% | 1 |  |
| positive regulation of immune response | 126 out of 2891 genes, 4.4% | 569 out of 15382 genes, 3.7% | 1 |  |
| N-terminal protein myristoylation | 3 out of 2891 genes, 0.1% | 4 out of 15382 genes, 0.0% | 1 |  |
| positive regulation of transcription from RNA polymerase II promoter involved in unfolded protein response | 3 out of 2891 genes, 0.1% | 4 out of 15382 genes, 0.0% | 1 |  |
| proteasomal ubiquitin-independent protein catabolic process | 3 out of 2891 genes, 0.1% | 4 out of 15382 genes, 0.0% | 1 |  |
| positive regulation of cytoplasmic mRNA processing body assembly | 3 out of 2891 genes, 0.1% | 4 out of 15382 genes, 0.0% | 1 |  |
| positive regulation of alkaline phosphatase activity | 3 out of 2891 genes, 0.1% | 4 out of 15382 genes, 0.0% | 1 |  |
| regulation of nitric oxide mediated signal transduction | 3 out of 2891 genes, 0.1% | 4 out of 15382 genes, 0.0% | 1 |  |
| nitrobenzene metabolic process | 3 out of 2891 genes, 0.1% | 4 out of 15382 genes, 0.0% | 1 |  |
| protein to membrane docking | 3 out of 2891 genes, 0.1% | 4 out of 15382 genes, 0.0% | 1 |  |
| negative regulation of transforming growth factor beta1 production | 3 out of 2891 genes, 0.1% | 4 out of 15382 genes, 0.0% | 1 |  |
| negative regulation of immature T cell proliferation in thymus | 3 out of 2891 genes, 0.1% | 4 out of 15382 genes, 0.0% | 1 |  |
| negative regulation of myosin-light-chain-phosphatase activity | 3 out of 2891 genes, 0.1% | 4 out of 15382 genes, 0.0% | 1 |  |
| purinergic receptor signaling pathway | 3 out of 2891 genes, 0.1% | 4 out of 15382 genes, 0.0% | 1 |  |
| hematopoietic stem cell migration | 3 out of 2891 genes, 0.1% | 4 out of 15382 genes, 0.0% | 1 |  |
| insulin receptor signaling pathway via phosphatidylinositol 3-kinase | 3 out of 2891 genes, 0.1% | 4 out of 15382 genes, 0.0% | 1 |  |
| oncostatin-M-mediated signaling pathway | 3 out of 2891 genes, 0.1% | 4 out of 15382 genes, 0.0% | 1 |  |
| transcription factor import into nucleus | 3 out of 2891 genes, 0.1% | 4 out of 15382 genes, 0.0% | 1 |  |
| cell-cell adhesion mediated by cadherin | 3 out of 2891 genes, 0.1% | 4 out of 15382 genes, 0.0% | 1 |  |
| regulation of gamma-delta T cell differentiation | 3 out of 2891 genes, 0.1% | 4 out of 15382 genes, 0.0% | 1 |  |
| GDP-L-fucose metabolic process | 3 out of 2891 genes, 0.1% | 4 out of 15382 genes, 0.0% | 1 |  |
| regulation of gamma-delta T cell activation | 3 out of 2891 genes, 0.1% | 4 out of 15382 genes, 0.0% | 1 |  |
| positive regulation of pinocytosis | 3 out of 2891 genes, 0.1% | 4 out of 15382 genes, 0.0% | 1 |  |
| myoblast fate commitment | 3 out of 2891 genes, 0.1% | 4 out of 15382 genes, 0.0% | 1 |  |
| voluntary musculoskeletal movement | 3 out of 2891 genes, 0.1% | 4 out of 15382 genes, 0.0% | 1 |  |
| positive regulation of NK T cell differentiation | 3 out of 2891 genes, 0.1% | 4 out of 15382 genes, 0.0% | 1 |  |
| myeloid leukocyte cytokine production | 3 out of 2891 genes, 0.1% | 4 out of 15382 genes, 0.0% | 1 |  |
| regulation of Wnt protein secretion | 3 out of 2891 genes, 0.1% | 4 out of 15382 genes, 0.0% | 1 |  |
| dephosphorylation of RNA polymerase II C-terminal domain | 3 out of 2891 genes, 0.1% | 4 out of 15382 genes, 0.0% | 1 |  |
| cellular response to hyperoxia | 3 out of 2891 genes, 0.1% | 4 out of 15382 genes, 0.0% | 1 |  |
| minus-end-directed organelle transport along microtubule | 3 out of 2891 genes, 0.1% | 4 out of 15382 genes, 0.0% | 1 |  |
| adrenergic receptor signaling pathway involved in heart process | 3 out of 2891 genes, 0.1% | 4 out of 15382 genes, 0.0% | 1 |  |
| negative regulation of protein K63-linked ubiquitination | 3 out of 2891 genes, 0.1% | 4 out of 15382 genes, 0.0% | 1 |  |
| regulation of membrane depolarization during cardiac muscle cell action potential | 3 out of 2891 genes, 0.1% | 4 out of 15382 genes, 0.0% | 1 |  |
| negative regulation of lymphocyte chemotaxis | 3 out of 2891 genes, 0.1% | 4 out of 15382 genes, 0.0% | 1 |  |
| regulation of peptidyl-serine dephosphorylation | 3 out of 2891 genes, 0.1% | 4 out of 15382 genes, 0.0% | 1 |  |
| positive regulation of endoplasmic reticulum tubular network organization | 3 out of 2891 genes, 0.1% | 4 out of 15382 genes, 0.0% | 1 |  |
| response to Thyroglobulin triiodothyronine | 3 out of 2891 genes, 0.1% | 4 out of 15382 genes, 0.0% | 1 |  |
| negative regulation of metalloendopeptidase activity | 3 out of 2891 genes, 0.1% | 4 out of 15382 genes, 0.0% | 1 |  |
| endoplasmic reticulum tubular network membrane organization | 3 out of 2891 genes, 0.1% | 4 out of 15382 genes, 0.0% | 1 |  |
| embryonic placenta development | 23 out of 2891 genes, 0.8% | 81 out of 15382 genes, 0.5% | 1 |  |
| carbohydrate metabolic process | 100 out of 2891 genes, 3.5% | 442 out of 15382 genes, 2.9% | 1 |  |
| antigen receptor-mediated signaling pathway | 40 out of 2891 genes, 1.4% | 157 out of 15382 genes, 1.0% | 1 |  |
| lipid localization | 57 out of 2891 genes, 2.0% | 236 out of 15382 genes, 1.5% | 1 |  |
| nucleobase-containing compound transport | 48 out of 2891 genes, 1.7% | 194 out of 15382 genes, 1.3% | 1 |  |
| G2/M transition of mitotic cell cycle | 32 out of 2891 genes, 1.1% | 121 out of 15382 genes, 0.8% | 1 |  |
| coagulation | 62 out of 2891 genes, 2.1% | 260 out of 15382 genes, 1.7% | 1 |  |
| positive regulation of cell motility | 91 out of 2891 genes, 3.1% | 399 out of 15382 genes, 2.6% | 1 |  |
| negative regulation of protein complex disassembly | 18 out of 2891 genes, 0.6% | 60 out of 15382 genes, 0.4% | 1 |  |
| regulation of cellular protein catabolic process | 59 out of 2891 genes, 2.0% | 246 out of 15382 genes, 1.6% | 1 |  |
| regulation of peptide transport | 159 out of 2891 genes, 5.5% | 734 out of 15382 genes, 4.8% | 1 |  |
| production of miRNAs involved in gene silencing by miRNA | 9 out of 2891 genes, 0.3% | 24 out of 15382 genes, 0.2% | 1 |  |
| negative regulation of signal transduction by p53 class mediator | 9 out of 2891 genes, 0.3% | 24 out of 15382 genes, 0.2% | 1 |  |
| regulation of glycolytic process | 17 out of 2891 genes, 0.6% | 56 out of 15382 genes, 0.4% | 1 |  |
| organelle disassembly | 17 out of 2891 genes, 0.6% | 56 out of 15382 genes, 0.4% | 1 |  |
| regulation of JUN kinase activity | 21 out of 2891 genes, 0.7% | 73 out of 15382 genes, 0.5% | 1 |  |
| positive regulation of neurotransmitter secretion | 5 out of 2891 genes, 0.2% | 10 out of 15382 genes, 0.1% | 1 |  |
| biotin metabolic process | 5 out of 2891 genes, 0.2% | 10 out of 15382 genes, 0.1% | 1 |  |
| neuron remodeling | 5 out of 2891 genes, 0.2% | 10 out of 15382 genes, 0.1% | 1 |  |
| mitochondrial calcium uptake | 5 out of 2891 genes, 0.2% | 10 out of 15382 genes, 0.1% | 1 |  |
| positive regulation of fatty acid oxidation | 5 out of 2891 genes, 0.2% | 10 out of 15382 genes, 0.1% | 1 |  |
| white fat cell differentiation | 5 out of 2891 genes, 0.2% | 10 out of 15382 genes, 0.1% | 1 |  |
| regulation of nuclear-transcribed mRNA poly(A) tail shortening | 5 out of 2891 genes, 0.2% | 10 out of 15382 genes, 0.1% | 1 |  |
| positive regulation of nuclear-transcribed mRNA poly(A) tail shortening | 5 out of 2891 genes, 0.2% | 10 out of 15382 genes, 0.1% | 1 |  |
| regulation of mRNA polyadenylation | 5 out of 2891 genes, 0.2% | 10 out of 15382 genes, 0.1% | 1 |  |
| positive regulation of protein localization to cell surface | 5 out of 2891 genes, 0.2% | 10 out of 15382 genes, 0.1% | 1 |  |
| regulation of cell-cell adhesion | 86 out of 2891 genes, 3.0% | 376 out of 15382 genes, 2.4% | 1 |  |
| nucleic acid transport | 43 out of 2891 genes, 1.5% | 172 out of 15382 genes, 1.1% | 1 |  |
| RNA transport | 43 out of 2891 genes, 1.5% | 172 out of 15382 genes, 1.1% | 1 |  |
| secretion by cell | 188 out of 2891 genes, 6.5% | 880 out of 15382 genes, 5.7% | 1 |  |
| protein glycosylation | 59 out of 2891 genes, 2.0% | 247 out of 15382 genes, 1.6% | 1 |  |
| macromolecule glycosylation | 59 out of 2891 genes, 2.0% | 247 out of 15382 genes, 1.6% | 1 |  |
| positive regulation of B cell activation | 20 out of 2891 genes, 0.7% | 69 out of 15382 genes, 0.4% | 1 |  |
| positive regulation of sodium ion transport | 10 out of 2891 genes, 0.3% | 28 out of 15382 genes, 0.2% | 1 |  |
| positive regulation of interferon-beta production | 10 out of 2891 genes, 0.3% | 28 out of 15382 genes, 0.2% | 1 |  |
| actin nucleation | 10 out of 2891 genes, 0.3% | 28 out of 15382 genes, 0.2% | 1 |  |
| myeloid leukocyte differentiation | 23 out of 2891 genes, 0.8% | 82 out of 15382 genes, 0.5% | 1 |  |
| circadian regulation of gene expression | 15 out of 2891 genes, 0.5% | 48 out of 15382 genes, 0.3% | 1 |  |
| regulation of alpha-beta T cell differentiation | 15 out of 2891 genes, 0.5% | 48 out of 15382 genes, 0.3% | 1 |  |
| response to sterol depletion | 4 out of 2891 genes, 0.1% | 7 out of 15382 genes, 0.0% | 1 |  |
| neuron-neuron synaptic transmission | 4 out of 2891 genes, 0.1% | 7 out of 15382 genes, 0.0% | 1 |  |
| regulation of cytoplasmic mRNA processing body assembly | 4 out of 2891 genes, 0.1% | 7 out of 15382 genes, 0.0% | 1 |  |
| astral microtubule organization | 4 out of 2891 genes, 0.1% | 7 out of 15382 genes, 0.0% | 1 |  |
| positive regulation of vesicle fusion | 4 out of 2891 genes, 0.1% | 7 out of 15382 genes, 0.0% | 1 |  |
| positive regulation of fatty acid beta-oxidation | 4 out of 2891 genes, 0.1% | 7 out of 15382 genes, 0.0% | 1 |  |
| SREBP signaling pathway | 4 out of 2891 genes, 0.1% | 7 out of 15382 genes, 0.0% | 1 |  |
| miRNA loading onto RISC involved in gene silencing by miRNA | 4 out of 2891 genes, 0.1% | 7 out of 15382 genes, 0.0% | 1 |  |
| peroxisome proliferator activated receptor signaling pathway | 4 out of 2891 genes, 0.1% | 7 out of 15382 genes, 0.0% | 1 |  |
| positive regulation of B cell receptor signaling pathway | 4 out of 2891 genes, 0.1% | 7 out of 15382 genes, 0.0% | 1 |  |
| centrosome separation | 4 out of 2891 genes, 0.1% | 7 out of 15382 genes, 0.0% | 1 |  |
| cellular response to sterol depletion | 4 out of 2891 genes, 0.1% | 7 out of 15382 genes, 0.0% | 1 |  |
| negative regulation of transforming growth factor beta production | 4 out of 2891 genes, 0.1% | 7 out of 15382 genes, 0.0% | 1 |  |
| positive regulation of deacetylase activity | 4 out of 2891 genes, 0.1% | 7 out of 15382 genes, 0.0% | 1 |  |
| positive regulation of spindle checkpoint | 4 out of 2891 genes, 0.1% | 7 out of 15382 genes, 0.0% | 1 |  |
| regulation of store-operated calcium channel activity | 4 out of 2891 genes, 0.1% | 7 out of 15382 genes, 0.0% | 1 |  |
| regulation of DNA catabolic process | 4 out of 2891 genes, 0.1% | 7 out of 15382 genes, 0.0% | 1 |  |
| positive regulation of chemokine (C-X-C motif) ligand 2 production | 4 out of 2891 genes, 0.1% | 7 out of 15382 genes, 0.0% | 1 |  |
| modulation by virus of host morphology or physiology | 11 out of 2891 genes, 0.4% | 32 out of 15382 genes, 0.2% | 1 |  |
| regulation of membrane lipid distribution | 11 out of 2891 genes, 0.4% | 32 out of 15382 genes, 0.2% | 1 |  |
| cellular response to drug | 65 out of 2891 genes, 2.2% | 276 out of 15382 genes, 1.8% | 1 |  |
| negative regulation of type I interferon production | 13 out of 2891 genes, 0.4% | 40 out of 15382 genes, 0.3% | 1 |  |
| coronary vasculature development | 12 out of 2891 genes, 0.4% | 36 out of 15382 genes, 0.2% | 1 |  |
| response to interleukin-7 | 19 out of 2891 genes, 0.7% | 65 out of 15382 genes, 0.4% | 1 |  |
| cellular response to interleukin-7 | 19 out of 2891 genes, 0.7% | 65 out of 15382 genes, 0.4% | 1 |  |
| regulation of platelet-derived growth factor receptor signaling pathway | 7 out of 2891 genes, 0.2% | 17 out of 15382 genes, 0.1% | 1 |  |
| regulation of cardiac muscle contraction by calcium ion signaling | 7 out of 2891 genes, 0.2% | 17 out of 15382 genes, 0.1% | 1 |  |
| negative regulation of telomere maintenance via telomerase | 7 out of 2891 genes, 0.2% | 17 out of 15382 genes, 0.1% | 1 |  |
| cytokinetic process | 7 out of 2891 genes, 0.2% | 17 out of 15382 genes, 0.1% | 1 |  |
| muscle cell proliferation | 7 out of 2891 genes, 0.2% | 17 out of 15382 genes, 0.1% | 1 |  |
| positive regulation of lipid catabolic process | 7 out of 2891 genes, 0.2% | 17 out of 15382 genes, 0.1% | 1 |  |
| negative regulation of cell junction assembly | 7 out of 2891 genes, 0.2% | 17 out of 15382 genes, 0.1% | 1 |  |
| positive regulation of cysteine-type endopeptidase activity involved in apoptotic signaling pathway | 7 out of 2891 genes, 0.2% | 17 out of 15382 genes, 0.1% | 1 |  |
| leukocyte differentiation | 66 out of 2891 genes, 2.3% | 281 out of 15382 genes, 1.8% | 1 |  |
| regulation of cell cycle G1/S phase transition | 35 out of 2891 genes, 1.2% | 136 out of 15382 genes, 0.9% | 1 |  |
| stem cell population maintenance | 33 out of 2891 genes, 1.1% | 127 out of 15382 genes, 0.8% | 1 |  |
| muscle cell development | 33 out of 2891 genes, 1.1% | 127 out of 15382 genes, 0.8% | 1 |  |
| regulation of generation of precursor metabolites and energy | 29 out of 2891 genes, 1.0% | 109 out of 15382 genes, 0.7% | 1 |  |
| response to organophosphorus | 31 out of 2891 genes, 1.1% | 118 out of 15382 genes, 0.8% | 1 |  |
| establishment of RNA localization | 43 out of 2891 genes, 1.5% | 173 out of 15382 genes, 1.1% | 1 |  |
| regulation of phosphorus metabolic process | 320 out of 2891 genes, 11.1% | 1551 out of 15382 genes, 10.1% | 1 |  |
| negative regulation of cell projection organization | 36 out of 2891 genes, 1.2% | 141 out of 15382 genes, 0.9% | 1 |  |
| regulation of carbohydrate metabolic process | 41 out of 2891 genes, 1.4% | 164 out of 15382 genes, 1.1% | 1 |  |
| organophosphate metabolic process | 180 out of 2891 genes, 6.2% | 843 out of 15382 genes, 5.5% | 1 |  |
| protein tetramerization | 30 out of 2891 genes, 1.0% | 114 out of 15382 genes, 0.7% | 1 |  |
| hemostasis | 62 out of 2891 genes, 2.1% | 263 out of 15382 genes, 1.7% | 1 |  |
| negative regulation of kinase activity | 51 out of 2891 genes, 1.8% | 211 out of 15382 genes, 1.4% | 1 |  |
| regulation of DNA repair | 23 out of 2891 genes, 0.8% | 83 out of 15382 genes, 0.5% | 1 |  |
| regulation of phosphate metabolic process | 317 out of 2891 genes, 11.0% | 1538 out of 15382 genes, 10.0% | 1 |  |
| dendrite morphogenesis | 16 out of 2891 genes, 0.6% | 53 out of 15382 genes, 0.3% | 1 |  |
| regulation of kinase activity | 152 out of 2891 genes, 5.3% | 704 out of 15382 genes, 4.6% | 1 |  |
| mitochondrial genome maintenance | 8 out of 2891 genes, 0.3% | 21 out of 15382 genes, 0.1% | 1 |  |
| regulation of chromatin binding | 8 out of 2891 genes, 0.3% | 21 out of 15382 genes, 0.1% | 1 |  |
| genetic imprinting | 8 out of 2891 genes, 0.3% | 21 out of 15382 genes, 0.1% | 1 |  |
| positive regulation of adherens junction organization | 8 out of 2891 genes, 0.3% | 21 out of 15382 genes, 0.1% | 1 |  |
| vasculature development | 105 out of 2891 genes, 3.6% | 472 out of 15382 genes, 3.1% | 1 |  |
| regulation of insulin secretion involved in cellular response to glucose stimulus | 15 out of 2891 genes, 0.5% | 49 out of 15382 genes, 0.3% | 1 |  |
| leukocyte activation | 175 out of 2891 genes, 6.1% | 820 out of 15382 genes, 5.3% | 1 |  |
| neuron death | 14 out of 2891 genes, 0.5% | 45 out of 15382 genes, 0.3% | 1 |  |
| regulation of exocytosis | 39 out of 2891 genes, 1.3% | 156 out of 15382 genes, 1.0% | 1 |  |
| positive regulation of defense response to virus by host | 9 out of 2891 genes, 0.3% | 25 out of 15382 genes, 0.2% | 1 |  |
| protein quality control for misfolded or incompletely synthesized proteins | 9 out of 2891 genes, 0.3% | 25 out of 15382 genes, 0.2% | 1 |  |
| protein kinase B signaling | 9 out of 2891 genes, 0.3% | 25 out of 15382 genes, 0.2% | 1 |  |
| regulation of B cell differentiation | 9 out of 2891 genes, 0.3% | 25 out of 15382 genes, 0.2% | 1 |  |
| microtubule polymerization | 9 out of 2891 genes, 0.3% | 25 out of 15382 genes, 0.2% | 1 |  |
| positive regulation of mRNA processing | 9 out of 2891 genes, 0.3% | 25 out of 15382 genes, 0.2% | 1 |  |
| protein K63-linked deubiquitination | 9 out of 2891 genes, 0.3% | 25 out of 15382 genes, 0.2% | 1 |  |
| ribonucleoprotein complex subunit organization | 48 out of 2891 genes, 1.7% | 198 out of 15382 genes, 1.3% | 1 |  |
| striated muscle cell proliferation | 6 out of 2891 genes, 0.2% | 14 out of 15382 genes, 0.1% | 1 |  |
| cellular response to ethanol | 6 out of 2891 genes, 0.2% | 14 out of 15382 genes, 0.1% | 1 |  |
| regulation of spindle checkpoint | 6 out of 2891 genes, 0.2% | 14 out of 15382 genes, 0.1% | 1 |  |
| protein destabilization | 13 out of 2891 genes, 0.4% | 41 out of 15382 genes, 0.3% | 1 |  |
| regulation of coenzyme metabolic process | 21 out of 2891 genes, 0.7% | 75 out of 15382 genes, 0.5% | 1 |  |
| regulation of defense response to virus by host | 12 out of 2891 genes, 0.4% | 37 out of 15382 genes, 0.2% | 1 |  |
| tricarboxylic acid metabolic process | 12 out of 2891 genes, 0.4% | 37 out of 15382 genes, 0.2% | 1 |  |
| activation of JUN kinase activity | 11 out of 2891 genes, 0.4% | 33 out of 15382 genes, 0.2% | 1 |  |
| regulation of microtubule polymerization | 11 out of 2891 genes, 0.4% | 33 out of 15382 genes, 0.2% | 1 |  |
| regulation of phosphoprotein phosphatase activity | 11 out of 2891 genes, 0.4% | 33 out of 15382 genes, 0.2% | 1 |  |
| regulation of cofactor metabolic process | 23 out of 2891 genes, 0.8% | 84 out of 15382 genes, 0.5% | 1 |  |
| maintenance of cell number | 33 out of 2891 genes, 1.1% | 129 out of 15382 genes, 0.8% | 1 |  |
| myeloid cell activation involved in immune response | 104 out of 2891 genes, 3.6% | 469 out of 15382 genes, 3.0% | 1 |  |
| actin filament-based movement | 25 out of 2891 genes, 0.9% | 93 out of 15382 genes, 0.6% | 1 |  |
| negative regulation of phosphatase activity | 20 out of 2891 genes, 0.7% | 71 out of 15382 genes, 0.5% | 1 |  |
| box H/ACA snoRNP assembly | 2 out of 2891 genes, 0.1% | 2 out of 15382 genes, 0.0% | 1 |  |
| DNA catabolic process, exonucleolytic | 2 out of 2891 genes, 0.1% | 2 out of 15382 genes, 0.0% | 1 |  |
| adenosine receptor signaling pathway | 2 out of 2891 genes, 0.1% | 2 out of 15382 genes, 0.0% | 1 |  |
| CDP-choline pathway | 2 out of 2891 genes, 0.1% | 2 out of 15382 genes, 0.0% | 1 |  |
| female courtship behavior | 2 out of 2891 genes, 0.1% | 2 out of 15382 genes, 0.0% | 1 |  |
| pathogenesis | 2 out of 2891 genes, 0.1% | 2 out of 15382 genes, 0.0% | 1 |  |
| response to high light intensity | 2 out of 2891 genes, 0.1% | 2 out of 15382 genes, 0.0% | 1 |  |
| unidimensional cell growth | 2 out of 2891 genes, 0.1% | 2 out of 15382 genes, 0.0% | 1 |  |
| regulation of chromatin disassembly | 2 out of 2891 genes, 0.1% | 2 out of 15382 genes, 0.0% | 1 |  |
| cholesterol storage | 2 out of 2891 genes, 0.1% | 2 out of 15382 genes, 0.0% | 1 |  |
| macrophage cytokine production | 2 out of 2891 genes, 0.1% | 2 out of 15382 genes, 0.0% | 1 |  |
| Schwann cell proliferation | 2 out of 2891 genes, 0.1% | 2 out of 15382 genes, 0.0% | 1 |  |
| regulation of lung blood pressure | 2 out of 2891 genes, 0.1% | 2 out of 15382 genes, 0.0% | 1 |  |
| ADP transport | 2 out of 2891 genes, 0.1% | 2 out of 15382 genes, 0.0% | 1 |  |
| N-terminal peptidyl-glycine N-myristoylation | 2 out of 2891 genes, 0.1% | 2 out of 15382 genes, 0.0% | 1 |  |
| virus maturation | 2 out of 2891 genes, 0.1% | 2 out of 15382 genes, 0.0% | 1 |  |
| sulfur oxidation | 2 out of 2891 genes, 0.1% | 2 out of 15382 genes, 0.0% | 1 |  |
| cerebellar Purkinje cell layer maturation | 2 out of 2891 genes, 0.1% | 2 out of 15382 genes, 0.0% | 1 |  |
| cerebellar cortex maturation | 2 out of 2891 genes, 0.1% | 2 out of 15382 genes, 0.0% | 1 |  |
| Golgi calcium ion transport | 2 out of 2891 genes, 0.1% | 2 out of 15382 genes, 0.0% | 1 |  |
| negative regulation of low-density lipoprotein particle receptor catabolic process | 2 out of 2891 genes, 0.1% | 2 out of 15382 genes, 0.0% | 1 |  |
| protein glycosylation in Golgi | 2 out of 2891 genes, 0.1% | 2 out of 15382 genes, 0.0% | 1 |  |
| glutathione transport | 2 out of 2891 genes, 0.1% | 2 out of 15382 genes, 0.0% | 1 |  |
| external genitalia morphogenesis | 2 out of 2891 genes, 0.1% | 2 out of 15382 genes, 0.0% | 1 |  |
| mRNA cleavage involved in gene silencing by miRNA | 2 out of 2891 genes, 0.1% | 2 out of 15382 genes, 0.0% | 1 |  |
| histone H3-T6 phosphorylation | 2 out of 2891 genes, 0.1% | 2 out of 15382 genes, 0.0% | 1 |  |
| histone H4-K20 demethylation | 2 out of 2891 genes, 0.1% | 2 out of 15382 genes, 0.0% | 1 |  |
| G-protein coupled purinergic receptor signaling pathway | 2 out of 2891 genes, 0.1% | 2 out of 15382 genes, 0.0% | 1 |  |
| oligopeptide transmembrane transport | 2 out of 2891 genes, 0.1% | 2 out of 15382 genes, 0.0% | 1 |  |
| protein localization to nuclear inner membrane | 2 out of 2891 genes, 0.1% | 2 out of 15382 genes, 0.0% | 1 |  |
| insulin receptor internalization | 2 out of 2891 genes, 0.1% | 2 out of 15382 genes, 0.0% | 1 |  |
| interleukin-23-mediated signaling pathway | 2 out of 2891 genes, 0.1% | 2 out of 15382 genes, 0.0% | 1 |  |
| modulation by virus of host autophagy | 2 out of 2891 genes, 0.1% | 2 out of 15382 genes, 0.0% | 1 |  |
| single stranded viral RNA replication via double stranded DNA intermediate | 2 out of 2891 genes, 0.1% | 2 out of 15382 genes, 0.0% | 1 |  |
| thiamine diphosphate metabolic process | 2 out of 2891 genes, 0.1% | 2 out of 15382 genes, 0.0% | 1 |  |
| tripeptide transport | 2 out of 2891 genes, 0.1% | 2 out of 15382 genes, 0.0% | 1 |  |
| positive regulation of amyloid precursor protein biosynthetic process | 2 out of 2891 genes, 0.1% | 2 out of 15382 genes, 0.0% | 1 |  |
| regulation of natural killer cell degranulation | 2 out of 2891 genes, 0.1% | 2 out of 15382 genes, 0.0% | 1 |  |
| positive regulation of natural killer cell degranulation | 2 out of 2891 genes, 0.1% | 2 out of 15382 genes, 0.0% | 1 |  |
| regulation of CD8-positive, alpha-beta T cell differentiation | 2 out of 2891 genes, 0.1% | 2 out of 15382 genes, 0.0% | 1 |  |
| regulation of myosin II filament organization | 2 out of 2891 genes, 0.1% | 2 out of 15382 genes, 0.0% | 1 |  |
| cortical microtubule organization | 2 out of 2891 genes, 0.1% | 2 out of 15382 genes, 0.0% | 1 |  |
| protein retention in Golgi apparatus | 2 out of 2891 genes, 0.1% | 2 out of 15382 genes, 0.0% | 1 |  |
| microtubule sliding | 2 out of 2891 genes, 0.1% | 2 out of 15382 genes, 0.0% | 1 |  |
| dihydrobiopterin metabolic process | 2 out of 2891 genes, 0.1% | 2 out of 15382 genes, 0.0% | 1 |  |
| inactivation of MAPKK activity | 2 out of 2891 genes, 0.1% | 2 out of 15382 genes, 0.0% | 1 |  |
| circadian temperature homeostasis | 2 out of 2891 genes, 0.1% | 2 out of 15382 genes, 0.0% | 1 |  |
| negative regulation of growth hormone secretion | 2 out of 2891 genes, 0.1% | 2 out of 15382 genes, 0.0% | 1 |  |
| definitive erythrocyte differentiation | 2 out of 2891 genes, 0.1% | 2 out of 15382 genes, 0.0% | 1 |  |
| cytoplasmic actin-based contraction involved in cell motility | 2 out of 2891 genes, 0.1% | 2 out of 15382 genes, 0.0% | 1 |  |
| cardiac fibroblast cell differentiation | 2 out of 2891 genes, 0.1% | 2 out of 15382 genes, 0.0% | 1 |  |
| cardiac fibroblast cell development | 2 out of 2891 genes, 0.1% | 2 out of 15382 genes, 0.0% | 1 |  |
| epicardium-derived cardiac fibroblast cell differentiation | 2 out of 2891 genes, 0.1% | 2 out of 15382 genes, 0.0% | 1 |  |
| epicardium-derived cardiac fibroblast cell development | 2 out of 2891 genes, 0.1% | 2 out of 15382 genes, 0.0% | 1 |  |
| regulation of chromatin silencing at rDNA | 2 out of 2891 genes, 0.1% | 2 out of 15382 genes, 0.0% | 1 |  |
| negative regulation of chromatin silencing at rDNA | 2 out of 2891 genes, 0.1% | 2 out of 15382 genes, 0.0% | 1 |  |
| reticulophagy | 2 out of 2891 genes, 0.1% | 2 out of 15382 genes, 0.0% | 1 |  |
| negative regulation of interleukin-6-mediated signaling pathway | 2 out of 2891 genes, 0.1% | 2 out of 15382 genes, 0.0% | 1 |  |
| negative regulation of nucleotide-binding oligomerization domain containing signaling pathway | 2 out of 2891 genes, 0.1% | 2 out of 15382 genes, 0.0% | 1 |  |
| negative regulation of nucleotide-binding oligomerization domain containing 2 signaling pathway | 2 out of 2891 genes, 0.1% | 2 out of 15382 genes, 0.0% | 1 |  |
| monocyte aggregation | 2 out of 2891 genes, 0.1% | 2 out of 15382 genes, 0.0% | 1 |  |
| cellular response to molecule of fungal origin | 2 out of 2891 genes, 0.1% | 2 out of 15382 genes, 0.0% | 1 |  |
| cellular response to light intensity | 2 out of 2891 genes, 0.1% | 2 out of 15382 genes, 0.0% | 1 |  |
| kidney mesenchymal cell proliferation | 2 out of 2891 genes, 0.1% | 2 out of 15382 genes, 0.0% | 1 |  |
| metanephric mesenchymal cell proliferation involved in metanephros development | 2 out of 2891 genes, 0.1% | 2 out of 15382 genes, 0.0% | 1 |  |
| purine-containing compound transmembrane transport | 2 out of 2891 genes, 0.1% | 2 out of 15382 genes, 0.0% | 1 |  |
| viral penetration into host nucleus | 2 out of 2891 genes, 0.1% | 2 out of 15382 genes, 0.0% | 1 |  |
| negative regulation of protein kinase C signaling | 2 out of 2891 genes, 0.1% | 2 out of 15382 genes, 0.0% | 1 |  |
| asymmetric Golgi ribbon formation | 2 out of 2891 genes, 0.1% | 2 out of 15382 genes, 0.0% | 1 |  |
| negative regulation of protein glycosylation in Golgi | 2 out of 2891 genes, 0.1% | 2 out of 15382 genes, 0.0% | 1 |  |
| hematopoietic stem cell migration to bone marrow | 2 out of 2891 genes, 0.1% | 2 out of 15382 genes, 0.0% | 1 |  |
| mRNA cleavage involved in gene silencing | 2 out of 2891 genes, 0.1% | 2 out of 15382 genes, 0.0% | 1 |  |
| synaptic vesicle endosomal processing | 2 out of 2891 genes, 0.1% | 2 out of 15382 genes, 0.0% | 1 |  |
| regulation of cargo loading into COPII-coated vesicle | 2 out of 2891 genes, 0.1% | 2 out of 15382 genes, 0.0% | 1 |  |
| negative regulation of hematopoietic stem cell differentiation | 2 out of 2891 genes, 0.1% | 2 out of 15382 genes, 0.0% | 1 |  |
| positive regulation of keratinocyte apoptotic process | 2 out of 2891 genes, 0.1% | 2 out of 15382 genes, 0.0% | 1 |  |
| multi-organism nuclear import | 2 out of 2891 genes, 0.1% | 2 out of 15382 genes, 0.0% | 1 |  |
| terminal web assembly | 2 out of 2891 genes, 0.1% | 2 out of 15382 genes, 0.0% | 1 |  |
| regulation of early endosome to recycling endosome transport | 2 out of 2891 genes, 0.1% | 2 out of 15382 genes, 0.0% | 1 |  |
| regulation of metalloendopeptidase activity involved in amyloid precursor protein catabolic process | 2 out of 2891 genes, 0.1% | 2 out of 15382 genes, 0.0% | 1 |  |
| negative regulation of metalloendopeptidase activity involved in amyloid precursor protein catabolic process | 2 out of 2891 genes, 0.1% | 2 out of 15382 genes, 0.0% | 1 |  |
| regulation of calcium ion-dependent exocytosis of neurotransmitter | 2 out of 2891 genes, 0.1% | 2 out of 15382 genes, 0.0% | 1 |  |
| regulation of homophilic cell adhesion | 2 out of 2891 genes, 0.1% | 2 out of 15382 genes, 0.0% | 1 |  |
| positive regulation of DNA catabolic process | 2 out of 2891 genes, 0.1% | 2 out of 15382 genes, 0.0% | 1 |  |
| regulation of transcription from RNA polymerase II promoter by histone modification | 2 out of 2891 genes, 0.1% | 2 out of 15382 genes, 0.0% | 1 |  |
| negative regulation of transcription from RNA polymerase II promoter by histone modification | 2 out of 2891 genes, 0.1% | 2 out of 15382 genes, 0.0% | 1 |  |
| regulation of protein processing in phagocytic vesicle | 2 out of 2891 genes, 0.1% | 2 out of 15382 genes, 0.0% | 1 |  |
| positive regulation of protein processing in phagocytic vesicle | 2 out of 2891 genes, 0.1% | 2 out of 15382 genes, 0.0% | 1 |  |
| cellular response to Thyroglobulin triiodothyronine | 2 out of 2891 genes, 0.1% | 2 out of 15382 genes, 0.0% | 1 |  |
| membrane protein proteolysis involved in retrograde protein transport, ER to cytosol | 2 out of 2891 genes, 0.1% | 2 out of 15382 genes, 0.0% | 1 |  |
| phosphatidylinositol 5-phosphate metabolic process | 2 out of 2891 genes, 0.1% | 2 out of 15382 genes, 0.0% | 1 |  |
| regulation of intracellular mRNA localization | 2 out of 2891 genes, 0.1% | 2 out of 15382 genes, 0.0% | 1 |  |
| positive regulation of intracellular mRNA localization | 2 out of 2891 genes, 0.1% | 2 out of 15382 genes, 0.0% | 1 |  |
| positive regulation of metalloendopeptidase activity | 2 out of 2891 genes, 0.1% | 2 out of 15382 genes, 0.0% | 1 |  |
| negative regulation of myofibroblast differentiation | 2 out of 2891 genes, 0.1% | 2 out of 15382 genes, 0.0% | 1 |  |
| regulation of neural crest cell differentiation | 2 out of 2891 genes, 0.1% | 2 out of 15382 genes, 0.0% | 1 |  |
| positive regulation of neural crest cell differentiation | 2 out of 2891 genes, 0.1% | 2 out of 15382 genes, 0.0% | 1 |  |
| regulation of cardiac neural crest cell migration involved in outflow tract morphogenesis | 2 out of 2891 genes, 0.1% | 2 out of 15382 genes, 0.0% | 1 |  |
| positive regulation of cardiac neural crest cell migration involved in outflow tract morphogenesis | 2 out of 2891 genes, 0.1% | 2 out of 15382 genes, 0.0% | 1 |  |
| regulation of Fc-gamma receptor signaling pathway involved in phagocytosis | 2 out of 2891 genes, 0.1% | 2 out of 15382 genes, 0.0% | 1 |  |
| positive regulation of Fc-gamma receptor signaling pathway involved in phagocytosis | 2 out of 2891 genes, 0.1% | 2 out of 15382 genes, 0.0% | 1 |  |
| negative regulation of lamellipodium morphogenesis | 2 out of 2891 genes, 0.1% | 2 out of 15382 genes, 0.0% | 1 |  |
| positive regulation of platelet-derived growth factor receptor-beta signaling pathway | 2 out of 2891 genes, 0.1% | 2 out of 15382 genes, 0.0% | 1 |  |
| negative regulation of dendritic cell apoptotic process | 2 out of 2891 genes, 0.1% | 2 out of 15382 genes, 0.0% | 1 |  |
| positive regulation of dense core granule biogenesis | 2 out of 2891 genes, 0.1% | 2 out of 15382 genes, 0.0% | 1 |  |
| negative regulation of integrin-mediated signaling pathway | 2 out of 2891 genes, 0.1% | 2 out of 15382 genes, 0.0% | 1 |  |
| regulation of response to gamma radiation | 2 out of 2891 genes, 0.1% | 2 out of 15382 genes, 0.0% | 1 |  |
| malonyl-CoA biosynthetic process | 2 out of 2891 genes, 0.1% | 2 out of 15382 genes, 0.0% | 1 |  |
| neutrophil activation involved in immune response | 98 out of 2891 genes, 3.4% | 440 out of 15382 genes, 2.9% | 1 |  |
| organic anion transport | 75 out of 2891 genes, 2.6% | 328 out of 15382 genes, 2.1% | 1 |  |
| regulation of proteolysis | 130 out of 2891 genes, 4.5% | 598 out of 15382 genes, 3.9% | 1 |  |
| regulation of TOR signaling | 22 out of 2891 genes, 0.8% | 80 out of 15382 genes, 0.5% | 1 |  |
| regulation of peptidase activity | 61 out of 2891 genes, 2.1% | 261 out of 15382 genes, 1.7% | 1 |  |
| dendrite development | 24 out of 2891 genes, 0.8% | 89 out of 15382 genes, 0.6% | 1 |  |
| stress-activated MAPK cascade | 24 out of 2891 genes, 0.8% | 89 out of 15382 genes, 0.6% | 1 |  |
| neuron projection development | 127 out of 2891 genes, 4.4% | 584 out of 15382 genes, 3.8% | 1 |  |
| regulation of proteasomal ubiquitin-dependent protein catabolic process | 26 out of 2891 genes, 0.9% | 98 out of 15382 genes, 0.6% | 1 |  |
| negative regulation of epithelial cell migration | 15 out of 2891 genes, 0.5% | 50 out of 15382 genes, 0.3% | 1 |  |
| negative regulation of cell-substrate adhesion | 15 out of 2891 genes, 0.5% | 50 out of 15382 genes, 0.3% | 1 |  |
| regulation of blood vessel endothelial cell migration | 15 out of 2891 genes, 0.5% | 50 out of 15382 genes, 0.3% | 1 |  |
| negative regulation of response to cytokine stimulus | 15 out of 2891 genes, 0.5% | 50 out of 15382 genes, 0.3% | 1 |  |
| miRNA metabolic process | 7 out of 2891 genes, 0.2% | 18 out of 15382 genes, 0.1% | 1 |  |
| Rac protein signal transduction | 7 out of 2891 genes, 0.2% | 18 out of 15382 genes, 0.1% | 1 |  |
| activation of protein kinase A activity | 7 out of 2891 genes, 0.2% | 18 out of 15382 genes, 0.1% | 1 |  |
| negative regulation of DNA repair | 7 out of 2891 genes, 0.2% | 18 out of 15382 genes, 0.1% | 1 |  |
| negative regulation of stress fiber assembly | 7 out of 2891 genes, 0.2% | 18 out of 15382 genes, 0.1% | 1 |  |
| negative regulation of gene silencing | 7 out of 2891 genes, 0.2% | 18 out of 15382 genes, 0.1% | 1 |  |
| global genome nucleotide-excision repair | 7 out of 2891 genes, 0.2% | 18 out of 15382 genes, 0.1% | 1 |  |
| regulation of cell migration involved in sprouting angiogenesis | 7 out of 2891 genes, 0.2% | 18 out of 15382 genes, 0.1% | 1 |  |
| lipid transport | 51 out of 2891 genes, 1.8% | 214 out of 15382 genes, 1.4% | 1 |  |
| regulation of G1/S transition of mitotic cell cycle | 31 out of 2891 genes, 1.1% | 121 out of 15382 genes, 0.8% | 1 |  |
| negative regulation of cytokine-mediated signaling pathway | 14 out of 2891 genes, 0.5% | 46 out of 15382 genes, 0.3% | 1 |  |
| regulation of tumor necrosis factor-mediated signaling pathway | 14 out of 2891 genes, 0.5% | 46 out of 15382 genes, 0.3% | 1 |  |
| receptor metabolic process | 23 out of 2891 genes, 0.8% | 85 out of 15382 genes, 0.6% | 1 |  |
| negative regulation of DNA metabolic process | 29 out of 2891 genes, 1.0% | 112 out of 15382 genes, 0.7% | 1 |  |
| positive regulation of endocytosis | 27 out of 2891 genes, 0.9% | 103 out of 15382 genes, 0.7% | 1 |  |
| keratinocyte development | 5 out of 2891 genes, 0.2% | 11 out of 15382 genes, 0.1% | 1 |  |
| lactate metabolic process | 5 out of 2891 genes, 0.2% | 11 out of 15382 genes, 0.1% | 1 |  |
| negative regulation of platelet-derived growth factor receptor signaling pathway | 5 out of 2891 genes, 0.2% | 11 out of 15382 genes, 0.1% | 1 |  |
| positive regulation of fibroblast migration | 5 out of 2891 genes, 0.2% | 11 out of 15382 genes, 0.1% | 1 |  |
| response to caffeine | 5 out of 2891 genes, 0.2% | 11 out of 15382 genes, 0.1% | 1 |  |
| regulation of histone phosphorylation | 5 out of 2891 genes, 0.2% | 11 out of 15382 genes, 0.1% | 1 |  |
| negative regulation of histone acetylation | 5 out of 2891 genes, 0.2% | 11 out of 15382 genes, 0.1% | 1 |  |
| response to diuretic | 5 out of 2891 genes, 0.2% | 11 out of 15382 genes, 0.1% | 1 |  |
| insulin-like growth factor receptor signaling pathway | 5 out of 2891 genes, 0.2% | 11 out of 15382 genes, 0.1% | 1 |  |
| chaperone cofactor-dependent protein refolding | 5 out of 2891 genes, 0.2% | 11 out of 15382 genes, 0.1% | 1 |  |
| Golgi localization | 5 out of 2891 genes, 0.2% | 11 out of 15382 genes, 0.1% | 1 |  |
| relaxation of cardiac muscle | 5 out of 2891 genes, 0.2% | 11 out of 15382 genes, 0.1% | 1 |  |
| regulation of ER to Golgi vesicle-mediated transport | 5 out of 2891 genes, 0.2% | 11 out of 15382 genes, 0.1% | 1 |  |
| Golgi ribbon formation | 5 out of 2891 genes, 0.2% | 11 out of 15382 genes, 0.1% | 1 |  |
| hepatocyte apoptotic process | 5 out of 2891 genes, 0.2% | 11 out of 15382 genes, 0.1% | 1 |  |
| neurotransmitter receptor transport to plasma membrane | 5 out of 2891 genes, 0.2% | 11 out of 15382 genes, 0.1% | 1 |  |
| granulocyte activation | 100 out of 2891 genes, 3.5% | 452 out of 15382 genes, 2.9% | 1 |  |
| regulation of insulin receptor signaling pathway | 13 out of 2891 genes, 0.4% | 42 out of 15382 genes, 0.3% | 1 |  |
| regulation of peptidyl-lysine acetylation | 13 out of 2891 genes, 0.4% | 42 out of 15382 genes, 0.3% | 1 |  |
| cellular response to nutrient levels | 43 out of 2891 genes, 1.5% | 177 out of 15382 genes, 1.2% | 1 |  |
| positive regulation of ion transmembrane transporter activity | 20 out of 2891 genes, 0.7% | 72 out of 15382 genes, 0.5% | 1 |  |
| response to purine-containing compound | 32 out of 2891 genes, 1.1% | 126 out of 15382 genes, 0.8% | 1 |  |
| regulation of developmental growth | 59 out of 2891 genes, 2.0% | 253 out of 15382 genes, 1.6% | 1 |  |
| positive regulation of JUN kinase activity | 17 out of 2891 genes, 0.6% | 59 out of 15382 genes, 0.4% | 1 |  |
| histone phosphorylation | 8 out of 2891 genes, 0.3% | 22 out of 15382 genes, 0.1% | 1 |  |
| protein transport within lipid bilayer | 8 out of 2891 genes, 0.3% | 22 out of 15382 genes, 0.1% | 1 |  |
| RNA destabilization | 8 out of 2891 genes, 0.3% | 22 out of 15382 genes, 0.1% | 1 |  |
| plasma membrane bounded cell projection morphogenesis | 95 out of 2891 genes, 3.3% | 428 out of 15382 genes, 2.8% | 1 |  |
| positive regulation of cell-matrix adhesion | 12 out of 2891 genes, 0.4% | 38 out of 15382 genes, 0.2% | 1 |  |
| negative regulation of TOR signaling | 12 out of 2891 genes, 0.4% | 38 out of 15382 genes, 0.2% | 1 |  |
| positive regulation of binding | 35 out of 2891 genes, 1.2% | 140 out of 15382 genes, 0.9% | 1 |  |
| positive regulation of transporter activity | 22 out of 2891 genes, 0.8% | 81 out of 15382 genes, 0.5% | 1 |  |
| maintenance of protein location | 22 out of 2891 genes, 0.8% | 81 out of 15382 genes, 0.5% | 1 |  |
| developmental cell growth | 22 out of 2891 genes, 0.8% | 81 out of 15382 genes, 0.5% | 1 |  |
| neutrophil degranulation | 97 out of 2891 genes, 3.4% | 438 out of 15382 genes, 2.8% | 1 |  |
| protein deubiquitination | 56 out of 2891 genes, 1.9% | 239 out of 15382 genes, 1.6% | 1 |  |
| syncytium formation by plasma membrane fusion | 9 out of 2891 genes, 0.3% | 26 out of 15382 genes, 0.2% | 1 |  |
| actin filament polymerization | 9 out of 2891 genes, 0.3% | 26 out of 15382 genes, 0.2% | 1 |  |
| modulation by symbiont of host cellular process | 9 out of 2891 genes, 0.3% | 26 out of 15382 genes, 0.2% | 1 |  |
| heart growth | 9 out of 2891 genes, 0.3% | 26 out of 15382 genes, 0.2% | 1 |  |
| positive regulation of mitochondrial outer membrane permeabilization involved in apoptotic signaling pathway | 9 out of 2891 genes, 0.3% | 26 out of 15382 genes, 0.2% | 1 |  |
| axonal transport | 11 out of 2891 genes, 0.4% | 34 out of 15382 genes, 0.2% | 1 |  |
| leukocyte degranulation | 101 out of 2891 genes, 3.5% | 458 out of 15382 genes, 3.0% | 1 |  |
| tricarboxylic acid cycle | 10 out of 2891 genes, 0.3% | 30 out of 15382 genes, 0.2% | 1 |  |
| Wnt signaling pathway, calcium modulating pathway | 10 out of 2891 genes, 0.3% | 30 out of 15382 genes, 0.2% | 1 |  |
| postsynapse organization | 10 out of 2891 genes, 0.3% | 30 out of 15382 genes, 0.2% | 1 |  |
| ribonucleoprotein complex assembly | 45 out of 2891 genes, 1.6% | 187 out of 15382 genes, 1.2% | 1 |  |
| execution phase of apoptosis | 16 out of 2891 genes, 0.6% | 55 out of 15382 genes, 0.4% | 1 |  |
| negative regulation of cellular response to transforming growth factor beta stimulus | 16 out of 2891 genes, 0.6% | 55 out of 15382 genes, 0.4% | 1 |  |
| regulation of leukocyte cell-cell adhesion | 68 out of 2891 genes, 2.4% | 297 out of 15382 genes, 1.9% | 1 |  |
| regulation of vesicle fusion | 19 out of 2891 genes, 0.7% | 68 out of 15382 genes, 0.4% | 1 |  |
| cell junction assembly | 31 out of 2891 genes, 1.1% | 122 out of 15382 genes, 0.8% | 1 |  |
| neuron projection morphogenesis | 94 out of 2891 genes, 3.3% | 424 out of 15382 genes, 2.8% | 1 |  |
| cell projection morphogenesis | 95 out of 2891 genes, 3.3% | 429 out of 15382 genes, 2.8% | 1 |  |
| glycosylation | 59 out of 2891 genes, 2.0% | 254 out of 15382 genes, 1.7% | 1 |  |
| RNA export from nucleus | 29 out of 2891 genes, 1.0% | 113 out of 15382 genes, 0.7% | 1 |  |
| cellular glucose homeostasis | 21 out of 2891 genes, 0.7% | 77 out of 15382 genes, 0.5% | 1 |  |
| regulation of actin filament bundle assembly | 21 out of 2891 genes, 0.7% | 77 out of 15382 genes, 0.5% | 1 |  |
| negative regulation of cell-cell adhesion | 34 out of 2891 genes, 1.2% | 136 out of 15382 genes, 0.9% | 1 |  |
| neutrophil activation | 99 out of 2891 genes, 3.4% | 449 out of 15382 genes, 2.9% | 1 |  |
| negative regulation of protein kinase activity | 47 out of 2891 genes, 1.6% | 197 out of 15382 genes, 1.3% | 1 |  |
| post-embryonic development | 23 out of 2891 genes, 0.8% | 86 out of 15382 genes, 0.6% | 1 |  |
| activation of GTPase activity | 23 out of 2891 genes, 0.8% | 86 out of 15382 genes, 0.6% | 1 |  |
| response to ionizing radiation | 32 out of 2891 genes, 1.1% | 127 out of 15382 genes, 0.8% | 1 |  |
| interleukin-7-mediated signaling pathway | 18 out of 2891 genes, 0.6% | 64 out of 15382 genes, 0.4% | 1 |  |
| positive regulation of neuron death | 18 out of 2891 genes, 0.6% | 64 out of 15382 genes, 0.4% | 1 |  |
| regulation of proteolysis involved in cellular protein catabolic process | 52 out of 2891 genes, 1.8% | 221 out of 15382 genes, 1.4% | 1 |  |
| regulation of mRNA catabolic process | 38 out of 2891 genes, 1.3% | 155 out of 15382 genes, 1.0% | 1 |  |
| aging | 58 out of 2891 genes, 2.0% | 250 out of 15382 genes, 1.6% | 1 |  |
| regulation of chromatin assembly or disassembly | 4 out of 2891 genes, 0.1% | 8 out of 15382 genes, 0.1% | 1 |  |
| substrate-dependent cell migration, cell extension | 4 out of 2891 genes, 0.1% | 8 out of 15382 genes, 0.1% | 1 |  |
| regulation of cardiac muscle adaptation | 4 out of 2891 genes, 0.1% | 8 out of 15382 genes, 0.1% | 1 |  |
| production of siRNA involved in RNA interference | 4 out of 2891 genes, 0.1% | 8 out of 15382 genes, 0.1% | 1 |  |
| nuclear-transcribed mRNA catabolic process, deadenylation-independent decay | 4 out of 2891 genes, 0.1% | 8 out of 15382 genes, 0.1% | 1 |  |
| positive regulation of natural killer cell differentiation | 4 out of 2891 genes, 0.1% | 8 out of 15382 genes, 0.1% | 1 |  |
| negative regulation of T cell differentiation in thymus | 4 out of 2891 genes, 0.1% | 8 out of 15382 genes, 0.1% | 1 |  |
| early endosome to Golgi transport | 4 out of 2891 genes, 0.1% | 8 out of 15382 genes, 0.1% | 1 |  |
| embryonic cleavage | 4 out of 2891 genes, 0.1% | 8 out of 15382 genes, 0.1% | 1 |  |
| response to folic acid | 4 out of 2891 genes, 0.1% | 8 out of 15382 genes, 0.1% | 1 |  |
| positive regulation of type I interferon-mediated signaling pathway | 4 out of 2891 genes, 0.1% | 8 out of 15382 genes, 0.1% | 1 |  |
| positive regulation of mucus secretion | 4 out of 2891 genes, 0.1% | 8 out of 15382 genes, 0.1% | 1 |  |
| regulation of protein K63-linked ubiquitination | 4 out of 2891 genes, 0.1% | 8 out of 15382 genes, 0.1% | 1 |  |
| negative regulation of mRNA polyadenylation | 4 out of 2891 genes, 0.1% | 8 out of 15382 genes, 0.1% | 1 |  |
| regulation of extracellular matrix assembly | 4 out of 2891 genes, 0.1% | 8 out of 15382 genes, 0.1% | 1 |  |
| regulation of interferon-alpha secretion | 4 out of 2891 genes, 0.1% | 8 out of 15382 genes, 0.1% | 1 |  |
| positive regulation of interferon-alpha secretion | 4 out of 2891 genes, 0.1% | 8 out of 15382 genes, 0.1% | 1 |  |
| negative regulation of autophagy of mitochondrion | 4 out of 2891 genes, 0.1% | 8 out of 15382 genes, 0.1% | 1 |  |
| regulation of cardiac muscle hypertrophy in response to stress | 4 out of 2891 genes, 0.1% | 8 out of 15382 genes, 0.1% | 1 |  |
| regulation of plasma membrane organization | 4 out of 2891 genes, 0.1% | 8 out of 15382 genes, 0.1% | 1 |  |
| negative regulation of sodium ion transmembrane transporter activity | 4 out of 2891 genes, 0.1% | 8 out of 15382 genes, 0.1% | 1 |  |
| regulation of alpha-beta T cell activation | 20 out of 2891 genes, 0.7% | 73 out of 15382 genes, 0.5% | 1 |  |
| protein polymerization | 20 out of 2891 genes, 0.7% | 73 out of 15382 genes, 0.5% | 1 |  |
| cytoskeleton-dependent cytokinesis | 14 out of 2891 genes, 0.5% | 47 out of 15382 genes, 0.3% | 1 |  |
| negative regulation of cytoskeleton organization | 28 out of 2891 genes, 1.0% | 109 out of 15382 genes, 0.7% | 1 |  |
| positive regulation of cytoskeleton organization | 42 out of 2891 genes, 1.5% | 174 out of 15382 genes, 1.1% | 1 |  |
| positive regulation of kinase activity | 101 out of 2891 genes, 3.5% | 460 out of 15382 genes, 3.0% | 1 |  |
| regulation of insulin secretion | 39 out of 2891 genes, 1.3% | 160 out of 15382 genes, 1.0% | 1 |  |
| myeloid leukocyte mediated immunity | 103 out of 2891 genes, 3.6% | 470 out of 15382 genes, 3.1% | 1 |  |
| mitochondrial fusion | 6 out of 2891 genes, 0.2% | 15 out of 15382 genes, 0.1% | 1 |  |
| ovulation | 6 out of 2891 genes, 0.2% | 15 out of 15382 genes, 0.1% | 1 |  |
| retrograde protein transport, ER to cytosol | 6 out of 2891 genes, 0.2% | 15 out of 15382 genes, 0.1% | 1 |  |
| V(D)J recombination | 6 out of 2891 genes, 0.2% | 15 out of 15382 genes, 0.1% | 1 |  |
| regulation of calcineurin-NFAT signaling cascade | 6 out of 2891 genes, 0.2% | 15 out of 15382 genes, 0.1% | 1 |  |
| regulation of calcineurin-mediated signaling | 6 out of 2891 genes, 0.2% | 15 out of 15382 genes, 0.1% | 1 |  |
| regulation of nuclear-transcribed mRNA catabolic process, deadenylation-dependent decay | 6 out of 2891 genes, 0.2% | 15 out of 15382 genes, 0.1% | 1 |  |
| positive regulation of nuclear-transcribed mRNA catabolic process, deadenylation-dependent decay | 6 out of 2891 genes, 0.2% | 15 out of 15382 genes, 0.1% | 1 |  |
| endoplasmic reticulum to cytosol transport | 6 out of 2891 genes, 0.2% | 15 out of 15382 genes, 0.1% | 1 |  |
| positive regulation of actin cytoskeleton reorganization | 6 out of 2891 genes, 0.2% | 15 out of 15382 genes, 0.1% | 1 |  |
| secretion | 204 out of 2891 genes, 7.1% | 977 out of 15382 genes, 6.4% | 1 |  |
| astrocyte differentiation | 13 out of 2891 genes, 0.4% | 43 out of 15382 genes, 0.3% | 1 |  |
| sister chromatid cohesion | 29 out of 2891 genes, 1.0% | 114 out of 15382 genes, 0.7% | 1 |  |
| embryonic limb morphogenesis | 29 out of 2891 genes, 1.0% | 114 out of 15382 genes, 0.7% | 1 |  |
| embryonic appendage morphogenesis | 29 out of 2891 genes, 1.0% | 114 out of 15382 genes, 0.7% | 1 |  |
| regulation of body fluid levels | 98 out of 2891 genes, 3.4% | 446 out of 15382 genes, 2.9% | 1 |  |
| cell cycle checkpoint | 44 out of 2891 genes, 1.5% | 184 out of 15382 genes, 1.2% | 1 |  |
| positive regulation of lymphocyte activation | 57 out of 2891 genes, 2.0% | 246 out of 15382 genes, 1.6% | 1 |  |
| neutrophil mediated immunity | 99 out of 2891 genes, 3.4% | 451 out of 15382 genes, 2.9% | 1 |  |
| regulation of receptor-mediated endocytosis | 19 out of 2891 genes, 0.7% | 69 out of 15382 genes, 0.4% | 1 |  |
| regulation of endothelial cell migration | 27 out of 2891 genes, 0.9% | 105 out of 15382 genes, 0.7% | 1 |  |
| calcium ion transmembrane transport | 27 out of 2891 genes, 0.9% | 105 out of 15382 genes, 0.7% | 1 |  |
| N-terminal protein lipidation | 3 out of 2891 genes, 0.1% | 5 out of 15382 genes, 0.0% | 1 |  |
| leucine catabolic process | 3 out of 2891 genes, 0.1% | 5 out of 15382 genes, 0.0% | 1 |  |
| L-serine biosynthetic process | 3 out of 2891 genes, 0.1% | 5 out of 15382 genes, 0.0% | 1 |  |
| tetrahydrobiopterin biosynthetic process | 3 out of 2891 genes, 0.1% | 5 out of 15382 genes, 0.0% | 1 |  |
| mitotic centrosome separation | 3 out of 2891 genes, 0.1% | 5 out of 15382 genes, 0.0% | 1 |  |
| detection of virus | 3 out of 2891 genes, 0.1% | 5 out of 15382 genes, 0.0% | 1 |  |
| negative regulation of cardiac muscle adaptation | 3 out of 2891 genes, 0.1% | 5 out of 15382 genes, 0.0% | 1 |  |
| negative regulation of Schwann cell proliferation | 3 out of 2891 genes, 0.1% | 5 out of 15382 genes, 0.0% | 1 |  |
| muscle cell apoptotic process | 3 out of 2891 genes, 0.1% | 5 out of 15382 genes, 0.0% | 1 |  |
| macrophage derived foam cell differentiation | 3 out of 2891 genes, 0.1% | 5 out of 15382 genes, 0.0% | 1 |  |
| positive regulation of lipoprotein particle clearance | 3 out of 2891 genes, 0.1% | 5 out of 15382 genes, 0.0% | 1 |  |
| regulation of skeletal muscle contraction by calcium ion signaling | 3 out of 2891 genes, 0.1% | 5 out of 15382 genes, 0.0% | 1 |  |
| protein myristoylation | 3 out of 2891 genes, 0.1% | 5 out of 15382 genes, 0.0% | 1 |  |
| activation of store-operated calcium channel activity | 3 out of 2891 genes, 0.1% | 5 out of 15382 genes, 0.0% | 1 |  |
| negative regulation of DNA endoreduplication | 3 out of 2891 genes, 0.1% | 5 out of 15382 genes, 0.0% | 1 |  |
| regulation of immature T cell proliferation in thymus | 3 out of 2891 genes, 0.1% | 5 out of 15382 genes, 0.0% | 1 |  |
| negative regulation of immature T cell proliferation | 3 out of 2891 genes, 0.1% | 5 out of 15382 genes, 0.0% | 1 |  |
| positive regulation of histone ubiquitination | 3 out of 2891 genes, 0.1% | 5 out of 15382 genes, 0.0% | 1 |  |
| regulation of interferon-beta secretion | 3 out of 2891 genes, 0.1% | 5 out of 15382 genes, 0.0% | 1 |  |
| positive regulation of interferon-beta secretion | 3 out of 2891 genes, 0.1% | 5 out of 15382 genes, 0.0% | 1 |  |
| osteoclast development | 3 out of 2891 genes, 0.1% | 5 out of 15382 genes, 0.0% | 1 |  |
| p38MAPK cascade | 3 out of 2891 genes, 0.1% | 5 out of 15382 genes, 0.0% | 1 |  |
| TORC1 signaling | 3 out of 2891 genes, 0.1% | 5 out of 15382 genes, 0.0% | 1 |  |
| macropinocytosis | 3 out of 2891 genes, 0.1% | 5 out of 15382 genes, 0.0% | 1 |  |
| tetrahydrobiopterin metabolic process | 3 out of 2891 genes, 0.1% | 5 out of 15382 genes, 0.0% | 1 |  |
| establishment of meiotic spindle localization | 3 out of 2891 genes, 0.1% | 5 out of 15382 genes, 0.0% | 1 |  |
| negative regulation of cell growth involved in cardiac muscle cell development | 3 out of 2891 genes, 0.1% | 5 out of 15382 genes, 0.0% | 1 |  |
| osteoclast fusion | 3 out of 2891 genes, 0.1% | 5 out of 15382 genes, 0.0% | 1 |  |
| foam cell differentiation | 3 out of 2891 genes, 0.1% | 5 out of 15382 genes, 0.0% | 1 |  |
| regulation of cardiac muscle cell action potential involved in regulation of contraction | 3 out of 2891 genes, 0.1% | 5 out of 15382 genes, 0.0% | 1 |  |
| positive regulation of amyloid-beta clearance | 3 out of 2891 genes, 0.1% | 5 out of 15382 genes, 0.0% | 1 |  |
| positive regulation of store-operated calcium channel activity | 3 out of 2891 genes, 0.1% | 5 out of 15382 genes, 0.0% | 1 |  |
| regulation of apoptotic DNA fragmentation | 3 out of 2891 genes, 0.1% | 5 out of 15382 genes, 0.0% | 1 |  |
| negative regulation of protein polyubiquitination | 3 out of 2891 genes, 0.1% | 5 out of 15382 genes, 0.0% | 1 |  |
| positive regulation of aspartic-type endopeptidase activity involved in amyloid precursor protein catabolic process | 3 out of 2891 genes, 0.1% | 5 out of 15382 genes, 0.0% | 1 |  |
| negative regulation of cardiac muscle hypertrophy in response to stress | 3 out of 2891 genes, 0.1% | 5 out of 15382 genes, 0.0% | 1 |  |
| regulation of endoplasmic reticulum tubular network organization | 3 out of 2891 genes, 0.1% | 5 out of 15382 genes, 0.0% | 1 |  |
| positive regulation of aspartic-type peptidase activity | 3 out of 2891 genes, 0.1% | 5 out of 15382 genes, 0.0% | 1 |  |
| regulation of protein localization to chromatin | 3 out of 2891 genes, 0.1% | 5 out of 15382 genes, 0.0% | 1 |  |
| regulation of amyloid fibril formation | 3 out of 2891 genes, 0.1% | 5 out of 15382 genes, 0.0% | 1 |  |
| positive regulation of cholesterol homeostasis | 3 out of 2891 genes, 0.1% | 5 out of 15382 genes, 0.0% | 1 |  |
| negative regulation of cAMP-dependent protein kinase activity | 3 out of 2891 genes, 0.1% | 5 out of 15382 genes, 0.0% | 1 |  |
| positive regulation of receptor catabolic process | 3 out of 2891 genes, 0.1% | 5 out of 15382 genes, 0.0% | 1 |  |
| regulation of dendritic cell apoptotic process | 3 out of 2891 genes, 0.1% | 5 out of 15382 genes, 0.0% | 1 |  |
| wound healing | 89 out of 2891 genes, 3.1% | 402 out of 15382 genes, 2.6% | 1 |  |
| response to osmotic stress | 16 out of 2891 genes, 0.6% | 56 out of 15382 genes, 0.4% | 1 |  |
| positive regulation of macroautophagy | 16 out of 2891 genes, 0.6% | 56 out of 15382 genes, 0.4% | 1 |  |
| regulation of B cell proliferation | 16 out of 2891 genes, 0.6% | 56 out of 15382 genes, 0.4% | 1 |  |
| negative regulation of nucleocytoplasmic transport | 16 out of 2891 genes, 0.6% | 56 out of 15382 genes, 0.4% | 1 |  |
| negative regulation of response to DNA damage stimulus | 16 out of 2891 genes, 0.6% | 56 out of 15382 genes, 0.4% | 1 |  |
| regulation of heart rate | 21 out of 2891 genes, 0.7% | 78 out of 15382 genes, 0.5% | 1 |  |
| receptor clustering | 12 out of 2891 genes, 0.4% | 39 out of 15382 genes, 0.3% | 1 |  |
| regulation of lipid metabolic process | 73 out of 2891 genes, 2.5% | 324 out of 15382 genes, 2.1% | 1 |  |
| microtubule nucleation | 7 out of 2891 genes, 0.2% | 19 out of 15382 genes, 0.1% | 1 |  |
| positive regulation of natural killer cell activation | 7 out of 2891 genes, 0.2% | 19 out of 15382 genes, 0.1% | 1 |  |
| regulation of DNA methylation | 7 out of 2891 genes, 0.2% | 19 out of 15382 genes, 0.1% | 1 |  |
| negative regulation of protein localization to plasma membrane | 7 out of 2891 genes, 0.2% | 19 out of 15382 genes, 0.1% | 1 |  |
| protein acylation | 33 out of 2891 genes, 1.1% | 133 out of 15382 genes, 0.9% | 1 |  |
| neuron apoptotic process | 11 out of 2891 genes, 0.4% | 35 out of 15382 genes, 0.2% | 1 |  |
| negative regulation of response to endoplasmic reticulum stress | 11 out of 2891 genes, 0.4% | 35 out of 15382 genes, 0.2% | 1 |  |
| response to peptide | 87 out of 2891 genes, 3.0% | 393 out of 15382 genes, 2.6% | 1 |  |
| regulation of transferase activity | 182 out of 2891 genes, 6.3% | 868 out of 15382 genes, 5.6% | 1 |  |
| negative regulation of mRNA processing | 10 out of 2891 genes, 0.3% | 31 out of 15382 genes, 0.2% | 1 |  |
| positive regulation of telomerase activity | 10 out of 2891 genes, 0.3% | 31 out of 15382 genes, 0.2% | 1 |  |
| protein targeting to vacuole | 8 out of 2891 genes, 0.3% | 23 out of 15382 genes, 0.1% | 1 |  |
| neuroblast proliferation | 8 out of 2891 genes, 0.3% | 23 out of 15382 genes, 0.1% | 1 |  |
| modulation by virus of host process | 8 out of 2891 genes, 0.3% | 23 out of 15382 genes, 0.1% | 1 |  |
| negative regulation of peptidyl-serine phosphorylation | 8 out of 2891 genes, 0.3% | 23 out of 15382 genes, 0.1% | 1 |  |
| regulation of transcription elongation from RNA polymerase II promoter | 8 out of 2891 genes, 0.3% | 23 out of 15382 genes, 0.1% | 1 |  |
| regulation of defense response to virus by virus | 8 out of 2891 genes, 0.3% | 23 out of 15382 genes, 0.1% | 1 |  |
| regulation of membrane repolarization | 8 out of 2891 genes, 0.3% | 23 out of 15382 genes, 0.1% | 1 |  |
| positive regulation of calcium ion transmembrane transporter activity | 8 out of 2891 genes, 0.3% | 23 out of 15382 genes, 0.1% | 1 |  |
| regulation of intrinsic apoptotic signaling pathway by p53 class mediator | 8 out of 2891 genes, 0.3% | 23 out of 15382 genes, 0.1% | 1 |  |
| regulation of steroid biosynthetic process | 20 out of 2891 genes, 0.7% | 74 out of 15382 genes, 0.5% | 1 |  |
| protein modification by small protein removal | 58 out of 2891 genes, 2.0% | 252 out of 15382 genes, 1.6% | 1 |  |
| negative regulation of protein serine/threonine kinase activity | 24 out of 2891 genes, 0.8% | 92 out of 15382 genes, 0.6% | 1 |  |
| regulation of lamellipodium assembly | 9 out of 2891 genes, 0.3% | 27 out of 15382 genes, 0.2% | 1 |  |
| regulation of protein export from nucleus | 9 out of 2891 genes, 0.3% | 27 out of 15382 genes, 0.2% | 1 |  |
| cell development | 290 out of 2891 genes, 10.0% | 1419 out of 15382 genes, 9.2% | 1 |  |
| positive regulation of stress-activated protein kinase signaling cascade | 32 out of 2891 genes, 1.1% | 129 out of 15382 genes, 0.8% | 1 |  |
| positive regulation of organelle assembly | 17 out of 2891 genes, 0.6% | 61 out of 15382 genes, 0.4% | 1 |  |
| regulation of double-strand break repair | 14 out of 2891 genes, 0.5% | 48 out of 15382 genes, 0.3% | 1 |  |
| cellular response to carbohydrate stimulus | 19 out of 2891 genes, 0.7% | 70 out of 15382 genes, 0.5% | 1 |  |
| regulation of protein localization to nucleus | 43 out of 2891 genes, 1.5% | 181 out of 15382 genes, 1.2% | 1 |  |
| positive regulation of proteasomal protein catabolic process | 21 out of 2891 genes, 0.7% | 79 out of 15382 genes, 0.5% | 1 |  |
| regulation of immune response | 178 out of 2891 genes, 6.2% | 850 out of 15382 genes, 5.5% | 1 |  |
| regulation of muscle system process | 44 out of 2891 genes, 1.5% | 186 out of 15382 genes, 1.2% | 1 |  |
| anion transport | 88 out of 2891 genes, 3.0% | 400 out of 15382 genes, 2.6% | 1 |  |
| cell activation | 197 out of 2891 genes, 6.8% | 947 out of 15382 genes, 6.2% | 1 |  |
| regulation of proteasomal protein catabolic process | 34 out of 2891 genes, 1.2% | 139 out of 15382 genes, 0.9% | 1 |  |
| RNA localization | 45 out of 2891 genes, 1.6% | 191 out of 15382 genes, 1.2% | 1 |  |
| nucleotide-excision repair, DNA duplex unwinding | 5 out of 2891 genes, 0.2% | 12 out of 15382 genes, 0.1% | 1 |  |
| positive regulation of receptor recycling | 5 out of 2891 genes, 0.2% | 12 out of 15382 genes, 0.1% | 1 |  |
| UDP-N-acetylglucosamine metabolic process | 5 out of 2891 genes, 0.2% | 12 out of 15382 genes, 0.1% | 1 |  |
| RNA interference | 5 out of 2891 genes, 0.2% | 12 out of 15382 genes, 0.1% | 1 |  |
| peptidyl-lysine dimethylation | 5 out of 2891 genes, 0.2% | 12 out of 15382 genes, 0.1% | 1 |  |
| pre-miRNA processing | 5 out of 2891 genes, 0.2% | 12 out of 15382 genes, 0.1% | 1 |  |
| regulation of fatty acid beta-oxidation | 5 out of 2891 genes, 0.2% | 12 out of 15382 genes, 0.1% | 1 |  |
| regulation of microvillus organization | 5 out of 2891 genes, 0.2% | 12 out of 15382 genes, 0.1% | 1 |  |
| protein localization to phagophore assembly site | 5 out of 2891 genes, 0.2% | 12 out of 15382 genes, 0.1% | 1 |  |
| positive regulation of vacuole organization | 5 out of 2891 genes, 0.2% | 12 out of 15382 genes, 0.1% | 1 |  |
| actin crosslink formation | 5 out of 2891 genes, 0.2% | 12 out of 15382 genes, 0.1% | 1 |  |
| negative regulation of focal adhesion assembly | 5 out of 2891 genes, 0.2% | 12 out of 15382 genes, 0.1% | 1 |  |
| coronary vasculature morphogenesis | 5 out of 2891 genes, 0.2% | 12 out of 15382 genes, 0.1% | 1 |  |
| 3'-UTR-mediated mRNA destabilization | 5 out of 2891 genes, 0.2% | 12 out of 15382 genes, 0.1% | 1 |  |
| mitotic cell cycle arrest | 5 out of 2891 genes, 0.2% | 12 out of 15382 genes, 0.1% | 1 |  |
| regulation of mitotic cell cycle spindle assembly checkpoint | 5 out of 2891 genes, 0.2% | 12 out of 15382 genes, 0.1% | 1 |  |
| activation of cysteine-type endopeptidase activity involved in apoptotic signaling pathway | 5 out of 2891 genes, 0.2% | 12 out of 15382 genes, 0.1% | 1 |  |
| regulation of mitotic spindle checkpoint | 5 out of 2891 genes, 0.2% | 12 out of 15382 genes, 0.1% | 1 |  |
| response to chemical | 698 out of 2891 genes, 24.1% | 3540 out of 15382 genes, 23.0% | 1 |  |
| regulation of carbohydrate catabolic process | 18 out of 2891 genes, 0.6% | 66 out of 15382 genes, 0.4% | 1 |  |
| gland development | 81 out of 2891 genes, 2.8% | 366 out of 15382 genes, 2.4% | 1 |  |
| ribonucleoprotein complex export from nucleus | 29 out of 2891 genes, 1.0% | 116 out of 15382 genes, 0.8% | 1 |  |
| morphogenesis of an epithelial sheet | 12 out of 2891 genes, 0.4% | 40 out of 15382 genes, 0.3% | 1 |  |
| modification by symbiont of host morphology or physiology | 12 out of 2891 genes, 0.4% | 40 out of 15382 genes, 0.3% | 1 |  |
| regulation of cholesterol biosynthetic process | 12 out of 2891 genes, 0.4% | 40 out of 15382 genes, 0.3% | 1 |  |
| myeloid leukocyte activation | 112 out of 2891 genes, 3.9% | 520 out of 15382 genes, 3.4% | 1 |  |
| positive regulation of transferase activity | 126 out of 2891 genes, 4.4% | 590 out of 15382 genes, 3.8% | 1 |  |
| lysosome organization | 15 out of 2891 genes, 0.5% | 53 out of 15382 genes, 0.3% | 1 |  |
| negative regulation of transforming growth factor beta receptor signaling pathway | 15 out of 2891 genes, 0.5% | 53 out of 15382 genes, 0.3% | 1 |  |
| lytic vacuole organization | 15 out of 2891 genes, 0.5% | 53 out of 15382 genes, 0.3% | 1 |  |
| homeostasis of number of cells | 40 out of 2891 genes, 1.4% | 168 out of 15382 genes, 1.1% | 1 |  |
| mitochondrial transport | 49 out of 2891 genes, 1.7% | 211 out of 15382 genes, 1.4% | 1 |  |
| cellular response to reactive oxygen species | 25 out of 2891 genes, 0.9% | 98 out of 15382 genes, 0.6% | 1 |  |
| protein deacetylation | 11 out of 2891 genes, 0.4% | 36 out of 15382 genes, 0.2% | 1 |  |
| histone ubiquitination | 11 out of 2891 genes, 0.4% | 36 out of 15382 genes, 0.2% | 1 |  |
| regulation of keratinocyte differentiation | 11 out of 2891 genes, 0.4% | 36 out of 15382 genes, 0.2% | 1 |  |
| regulation of spindle organization | 11 out of 2891 genes, 0.4% | 36 out of 15382 genes, 0.2% | 1 |  |
| positive regulation of T cell differentiation | 17 out of 2891 genes, 0.6% | 62 out of 15382 genes, 0.4% | 1 |  |
| regulation of response to endoplasmic reticulum stress | 17 out of 2891 genes, 0.6% | 62 out of 15382 genes, 0.4% | 1 |  |
| cell activation involved in immune response | 120 out of 2891 genes, 4.2% | 561 out of 15382 genes, 3.6% | 1 |  |
| cardiac muscle cell differentiation | 19 out of 2891 genes, 0.7% | 71 out of 15382 genes, 0.5% | 1 |  |
| response to hexose | 31 out of 2891 genes, 1.1% | 126 out of 15382 genes, 0.8% | 1 |  |
| transforming growth factor beta receptor signaling pathway | 21 out of 2891 genes, 0.7% | 80 out of 15382 genes, 0.5% | 1 |  |
| protein targeting to peroxisome | 6 out of 2891 genes, 0.2% | 16 out of 15382 genes, 0.1% | 1 |  |
| nuclear migration | 6 out of 2891 genes, 0.2% | 16 out of 15382 genes, 0.1% | 1 |  |
| negative regulation of B cell proliferation | 6 out of 2891 genes, 0.2% | 16 out of 15382 genes, 0.1% | 1 |  |
| positive regulation of transcription from RNA polymerase II promoter in response to stress | 6 out of 2891 genes, 0.2% | 16 out of 15382 genes, 0.1% | 1 |  |
| inositol phosphate-mediated signaling | 6 out of 2891 genes, 0.2% | 16 out of 15382 genes, 0.1% | 1 |  |
| protein localization to peroxisome | 6 out of 2891 genes, 0.2% | 16 out of 15382 genes, 0.1% | 1 |  |
| establishment of protein localization to peroxisome | 6 out of 2891 genes, 0.2% | 16 out of 15382 genes, 0.1% | 1 |  |
| negative regulation of endoplasmic reticulum stress-induced intrinsic apoptotic signaling pathway | 6 out of 2891 genes, 0.2% | 16 out of 15382 genes, 0.1% | 1 |  |
| positive regulation of sodium ion transmembrane transport | 6 out of 2891 genes, 0.2% | 16 out of 15382 genes, 0.1% | 1 |  |
| negative regulation of double-strand break repair | 6 out of 2891 genes, 0.2% | 16 out of 15382 genes, 0.1% | 1 |  |
| phospholipid transport | 14 out of 2891 genes, 0.5% | 49 out of 15382 genes, 0.3% | 1 |  |
| positive regulation of alpha-beta T cell activation | 14 out of 2891 genes, 0.5% | 49 out of 15382 genes, 0.3% | 1 |  |
| positive regulation of RNA splicing | 10 out of 2891 genes, 0.3% | 32 out of 15382 genes, 0.2% | 1 |  |
| positive regulation of intracellular transport | 47 out of 2891 genes, 1.6% | 202 out of 15382 genes, 1.3% | 1 |  |
| regulation of microtubule cytoskeleton organization | 35 out of 2891 genes, 1.2% | 145 out of 15382 genes, 0.9% | 1 |  |
| muscle tissue development | 60 out of 2891 genes, 2.1% | 265 out of 15382 genes, 1.7% | 1 |  |
| circadian rhythm | 29 out of 2891 genes, 1.0% | 117 out of 15382 genes, 0.8% | 1 |  |
| negative regulation of neuron projection development | 29 out of 2891 genes, 1.0% | 117 out of 15382 genes, 0.8% | 1 |  |
| mRNA polyadenylation | 9 out of 2891 genes, 0.3% | 28 out of 15382 genes, 0.2% | 1 |  |
| syncytium formation | 9 out of 2891 genes, 0.3% | 28 out of 15382 genes, 0.2% | 1 |  |
| regulation of keratinocyte proliferation | 9 out of 2891 genes, 0.3% | 28 out of 15382 genes, 0.2% | 1 |  |
| sulfur compound transport | 9 out of 2891 genes, 0.3% | 28 out of 15382 genes, 0.2% | 1 |  |
| regulation of endoplasmic reticulum stress-induced intrinsic apoptotic signaling pathway | 9 out of 2891 genes, 0.3% | 28 out of 15382 genes, 0.2% | 1 |  |
| regulation of cardiocyte differentiation | 9 out of 2891 genes, 0.3% | 28 out of 15382 genes, 0.2% | 1 |  |
| ruffle organization | 7 out of 2891 genes, 0.2% | 20 out of 15382 genes, 0.1% | 1 |  |
| negative regulation of actin filament bundle assembly | 7 out of 2891 genes, 0.2% | 20 out of 15382 genes, 0.1% | 1 |  |
| microtubule anchoring | 7 out of 2891 genes, 0.2% | 20 out of 15382 genes, 0.1% | 1 |  |
| cytoplasmic sequestering of protein | 7 out of 2891 genes, 0.2% | 20 out of 15382 genes, 0.1% | 1 |  |
| mitochondrion morphogenesis | 7 out of 2891 genes, 0.2% | 20 out of 15382 genes, 0.1% | 1 |  |
| protein K48-linked deubiquitination | 7 out of 2891 genes, 0.2% | 20 out of 15382 genes, 0.1% | 1 |  |
| regulation of stem cell population maintenance | 7 out of 2891 genes, 0.2% | 20 out of 15382 genes, 0.1% | 1 |  |
| regulation of anoikis | 7 out of 2891 genes, 0.2% | 20 out of 15382 genes, 0.1% | 1 |  |
| chromatin silencing | 16 out of 2891 genes, 0.6% | 58 out of 15382 genes, 0.4% | 1 |  |
| cilium organization | 71 out of 2891 genes, 2.5% | 319 out of 15382 genes, 2.1% | 1 |  |
| apoptotic nuclear changes | 8 out of 2891 genes, 0.3% | 24 out of 15382 genes, 0.2% | 1 |  |
| positive regulation of microtubule polymerization or depolymerization | 8 out of 2891 genes, 0.3% | 24 out of 15382 genes, 0.2% | 1 |  |
| regulation of homotypic cell-cell adhesion | 8 out of 2891 genes, 0.3% | 24 out of 15382 genes, 0.2% | 1 |  |
| intrinsic apoptotic signaling pathway in response to DNA damage by p53 class mediator | 8 out of 2891 genes, 0.3% | 24 out of 15382 genes, 0.2% | 1 |  |
| negative regulation of fibroblast proliferation | 8 out of 2891 genes, 0.3% | 24 out of 15382 genes, 0.2% | 1 |  |
| regulation of spindle assembly | 8 out of 2891 genes, 0.3% | 24 out of 15382 genes, 0.2% | 1 |  |
| regulation of ruffle assembly | 8 out of 2891 genes, 0.3% | 24 out of 15382 genes, 0.2% | 1 |  |
| positive regulation of JNK cascade | 27 out of 2891 genes, 0.9% | 108 out of 15382 genes, 0.7% | 1 |  |
| organophosphate ester transport | 18 out of 2891 genes, 0.6% | 67 out of 15382 genes, 0.4% | 1 |  |
| positive regulation of myeloid cell differentiation | 20 out of 2891 genes, 0.7% | 76 out of 15382 genes, 0.5% | 1 |  |
| regulation of viral life cycle | 30 out of 2891 genes, 1.0% | 122 out of 15382 genes, 0.8% | 1 |  |
| muscle structure development | 92 out of 2891 genes, 3.2% | 423 out of 15382 genes, 2.7% | 1 |  |
| double-strand break repair | 37 out of 2891 genes, 1.3% | 155 out of 15382 genes, 1.0% | 1 |  |
| negative regulation of protein catabolic process | 25 out of 2891 genes, 0.9% | 99 out of 15382 genes, 0.6% | 1 |  |
| blood vessel morphogenesis | 80 out of 2891 genes, 2.8% | 364 out of 15382 genes, 2.4% | 1 |  |
| cellular response to extracellular stimulus | 47 out of 2891 genes, 1.6% | 203 out of 15382 genes, 1.3% | 1 |  |
| regulation of epithelial cell differentiation | 28 out of 2891 genes, 1.0% | 113 out of 15382 genes, 0.7% | 1 |  |
| positive regulation of leukocyte mediated immunity | 23 out of 2891 genes, 0.8% | 90 out of 15382 genes, 0.6% | 1 |  |
| appendage morphogenesis | 32 out of 2891 genes, 1.1% | 132 out of 15382 genes, 0.9% | 1 |  |
| limb morphogenesis | 32 out of 2891 genes, 1.1% | 132 out of 15382 genes, 0.9% | 1 |  |
| neuron development | 151 out of 2891 genes, 5.2% | 720 out of 15382 genes, 4.7% | 1 |  |
| regulation of T cell activation | 64 out of 2891 genes, 2.2% | 286 out of 15382 genes, 1.9% | 1 |  |
| embryonic epithelial tube formation | 26 out of 2891 genes, 0.9% | 104 out of 15382 genes, 0.7% | 1 |  |
| pinocytosis | 4 out of 2891 genes, 0.1% | 9 out of 15382 genes, 0.1% | 1 |  |
| muscle atrophy | 4 out of 2891 genes, 0.1% | 9 out of 15382 genes, 0.1% | 1 |  |
| stress granule assembly | 4 out of 2891 genes, 0.1% | 9 out of 15382 genes, 0.1% | 1 |  |
| histone arginine methylation | 4 out of 2891 genes, 0.1% | 9 out of 15382 genes, 0.1% | 1 |  |
| intraciliary retrograde transport | 4 out of 2891 genes, 0.1% | 9 out of 15382 genes, 0.1% | 1 |  |
| ATF6-mediated unfolded protein response | 4 out of 2891 genes, 0.1% | 9 out of 15382 genes, 0.1% | 1 |  |
| regulation of amyloid precursor protein biosynthetic process | 4 out of 2891 genes, 0.1% | 9 out of 15382 genes, 0.1% | 1 |  |
| amino sugar biosynthetic process | 4 out of 2891 genes, 0.1% | 9 out of 15382 genes, 0.1% | 1 |  |
| positive regulation of viral entry into host cell | 4 out of 2891 genes, 0.1% | 9 out of 15382 genes, 0.1% | 1 |  |
| contact inhibition | 4 out of 2891 genes, 0.1% | 9 out of 15382 genes, 0.1% | 1 |  |
| regulation of cell growth involved in cardiac muscle cell development | 4 out of 2891 genes, 0.1% | 9 out of 15382 genes, 0.1% | 1 |  |
| small RNA loading onto RISC | 4 out of 2891 genes, 0.1% | 9 out of 15382 genes, 0.1% | 1 |  |
| histone mRNA catabolic process | 4 out of 2891 genes, 0.1% | 9 out of 15382 genes, 0.1% | 1 |  |
| positive regulation of ruffle assembly | 4 out of 2891 genes, 0.1% | 9 out of 15382 genes, 0.1% | 1 |  |
| positive regulation of cell cycle checkpoint | 4 out of 2891 genes, 0.1% | 9 out of 15382 genes, 0.1% | 1 |  |
| negative regulation of sodium ion transmembrane transport | 4 out of 2891 genes, 0.1% | 9 out of 15382 genes, 0.1% | 1 |  |
| negative regulation of cardiocyte differentiation | 4 out of 2891 genes, 0.1% | 9 out of 15382 genes, 0.1% | 1 |  |
| negative regulation of cardiac muscle cell differentiation | 4 out of 2891 genes, 0.1% | 9 out of 15382 genes, 0.1% | 1 |  |
| regulation of CD8-positive, alpha-beta T cell activation | 4 out of 2891 genes, 0.1% | 9 out of 15382 genes, 0.1% | 1 |  |
| negative regulation of cellular protein catabolic process | 17 out of 2891 genes, 0.6% | 63 out of 15382 genes, 0.4% | 1 |  |
| positive regulation of lymphocyte mediated immunity | 19 out of 2891 genes, 0.7% | 72 out of 15382 genes, 0.5% | 1 |  |
| ribonucleoprotein complex localization | 29 out of 2891 genes, 1.0% | 118 out of 15382 genes, 0.8% | 1 |  |
| antigen processing and presentation of exogenous antigen | 40 out of 2891 genes, 1.4% | 170 out of 15382 genes, 1.1% | 1 |  |
| response to carbohydrate | 36 out of 2891 genes, 1.2% | 151 out of 15382 genes, 1.0% | 1 |  |
| positive regulation of immune system process | 175 out of 2891 genes, 6.1% | 842 out of 15382 genes, 5.5% | 1 |  |
| positive regulation of developmental growth | 33 out of 2891 genes, 1.1% | 137 out of 15382 genes, 0.9% | 1 |  |
| regulation of protein kinase activity | 137 out of 2891 genes, 4.7% | 650 out of 15382 genes, 4.2% | 1 |  |
| renal system process | 24 out of 2891 genes, 0.8% | 95 out of 15382 genes, 0.6% | 1 |  |
| regulation of cell cycle arrest | 24 out of 2891 genes, 0.8% | 95 out of 15382 genes, 0.6% | 1 |  |
| antigen processing and presentation | 46 out of 2891 genes, 1.6% | 199 out of 15382 genes, 1.3% | 1 |  |
| regulation of transporter activity | 46 out of 2891 genes, 1.6% | 199 out of 15382 genes, 1.3% | 1 |  |
| transcription initiation from RNA polymerase II promoter | 30 out of 2891 genes, 1.0% | 123 out of 15382 genes, 0.8% | 1 |  |
| mitotic cytokinesis | 11 out of 2891 genes, 0.4% | 37 out of 15382 genes, 0.2% | 1 |  |
| inner mitochondrial membrane organization | 11 out of 2891 genes, 0.4% | 37 out of 15382 genes, 0.2% | 1 |  |
| regulation of T cell receptor signaling pathway | 11 out of 2891 genes, 0.4% | 37 out of 15382 genes, 0.2% | 1 |  |
| glycogen metabolic process | 14 out of 2891 genes, 0.5% | 50 out of 15382 genes, 0.3% | 1 |  |
| negative regulation of protein import into nucleus | 14 out of 2891 genes, 0.5% | 50 out of 15382 genes, 0.3% | 1 |  |
| negative regulation of viral genome replication | 14 out of 2891 genes, 0.5% | 50 out of 15382 genes, 0.3% | 1 |  |
| regulation of cholesterol metabolic process | 14 out of 2891 genes, 0.5% | 50 out of 15382 genes, 0.3% | 1 |  |
| negative regulation of protein import | 14 out of 2891 genes, 0.5% | 50 out of 15382 genes, 0.3% | 1 |  |
| response to antineoplastic agent | 22 out of 2891 genes, 0.8% | 86 out of 15382 genes, 0.6% | 1 |  |
| regulation of protein ubiquitination | 54 out of 2891 genes, 1.9% | 238 out of 15382 genes, 1.5% | 1 |  |
| carbohydrate derivative biosynthetic process | 118 out of 2891 genes, 4.1% | 555 out of 15382 genes, 3.6% | 1 |  |
| regulation of cell growth | 76 out of 2891 genes, 2.6% | 346 out of 15382 genes, 2.2% | 1 |  |
| response to dsRNA | 20 out of 2891 genes, 0.7% | 77 out of 15382 genes, 0.5% | 1 |  |
| extrinsic apoptotic signaling pathway | 20 out of 2891 genes, 0.7% | 77 out of 15382 genes, 0.5% | 1 |  |
| positive regulation of stress-activated MAPK cascade | 31 out of 2891 genes, 1.1% | 128 out of 15382 genes, 0.8% | 1 |  |
| ubiquitin-dependent ERAD pathway | 16 out of 2891 genes, 0.6% | 59 out of 15382 genes, 0.4% | 1 |  |
| regulation of mitochondrial membrane permeability | 16 out of 2891 genes, 0.6% | 59 out of 15382 genes, 0.4% | 1 |  |
| epithelial cell development | 39 out of 2891 genes, 1.3% | 166 out of 15382 genes, 1.1% | 1 |  |
| regulation of protein kinase B signaling | 28 out of 2891 genes, 1.0% | 114 out of 15382 genes, 0.7% | 1 |  |
| alcohol metabolic process | 57 out of 2891 genes, 2.0% | 253 out of 15382 genes, 1.6% | 1 |  |
| anatomical structure formation involved in morphogenesis | 164 out of 2891 genes, 5.7% | 788 out of 15382 genes, 5.1% | 1 |  |
| cilium assembly | 68 out of 2891 genes, 2.4% | 307 out of 15382 genes, 2.0% | 1 |  |
| regulation of mitotic spindle organization | 10 out of 2891 genes, 0.3% | 33 out of 15382 genes, 0.2% | 1 |  |
| negative regulation of T cell activation | 23 out of 2891 genes, 0.8% | 91 out of 15382 genes, 0.6% | 1 |  |
| protein heterooligomerization | 23 out of 2891 genes, 0.8% | 91 out of 15382 genes, 0.6% | 1 |  |
| leukocyte activation involved in immune response | 118 out of 2891 genes, 4.1% | 556 out of 15382 genes, 3.6% | 1 |  |
| regulation of leukocyte differentiation | 52 out of 2891 genes, 1.8% | 229 out of 15382 genes, 1.5% | 1 |  |
| interleukin-12-mediated signaling pathway | 13 out of 2891 genes, 0.4% | 46 out of 15382 genes, 0.3% | 1 |  |
| cellular response to interleukin-12 | 13 out of 2891 genes, 0.4% | 46 out of 15382 genes, 0.3% | 1 |  |
| positive regulation of leukocyte differentiation | 29 out of 2891 genes, 1.0% | 119 out of 15382 genes, 0.8% | 1 |  |
| histone acetylation | 21 out of 2891 genes, 0.7% | 82 out of 15382 genes, 0.5% | 1 |  |
| regulation of multi-organism process | 79 out of 2891 genes, 2.7% | 362 out of 15382 genes, 2.4% | 1 |  |
| divalent metal ion transport | 48 out of 2891 genes, 1.7% | 210 out of 15382 genes, 1.4% | 1 |  |
| positive regulation of protein complex disassembly | 9 out of 2891 genes, 0.3% | 29 out of 15382 genes, 0.2% | 1 |  |
| protein depolymerization | 9 out of 2891 genes, 0.3% | 29 out of 15382 genes, 0.2% | 1 |  |
| cellular response to glucagon stimulus | 9 out of 2891 genes, 0.3% | 29 out of 15382 genes, 0.2% | 1 |  |
| positive regulation of response to endoplasmic reticulum stress | 9 out of 2891 genes, 0.3% | 29 out of 15382 genes, 0.2% | 1 |  |
| cellular protein complex assembly | 80 out of 2891 genes, 2.8% | 367 out of 15382 genes, 2.4% | 1 |  |
| negative regulation of protein depolymerization | 15 out of 2891 genes, 0.5% | 55 out of 15382 genes, 0.4% | 1 |  |
| JNK cascade | 19 out of 2891 genes, 0.7% | 73 out of 15382 genes, 0.5% | 1 |  |
| negative regulation of myeloid cell differentiation | 19 out of 2891 genes, 0.7% | 73 out of 15382 genes, 0.5% | 1 |  |
| nuclear transport | 30 out of 2891 genes, 1.0% | 124 out of 15382 genes, 0.8% | 1 |  |
| cell migration involved in sprouting angiogenesis | 5 out of 2891 genes, 0.2% | 13 out of 15382 genes, 0.1% | 1 |  |
| protein import into peroxisome matrix | 5 out of 2891 genes, 0.2% | 13 out of 15382 genes, 0.1% | 1 |  |
| cardiolipin metabolic process | 5 out of 2891 genes, 0.2% | 13 out of 15382 genes, 0.1% | 1 |  |
| endosome transport via multivesicular body sorting pathway | 5 out of 2891 genes, 0.2% | 13 out of 15382 genes, 0.1% | 1 |  |
| regulation of Arp2/3 complex-mediated actin nucleation | 5 out of 2891 genes, 0.2% | 13 out of 15382 genes, 0.1% | 1 |  |
| regulation of smooth muscle cell apoptotic process | 5 out of 2891 genes, 0.2% | 13 out of 15382 genes, 0.1% | 1 |  |
| positive regulation of chromatin binding | 5 out of 2891 genes, 0.2% | 13 out of 15382 genes, 0.1% | 1 |  |
| histone H4-K5 acetylation | 5 out of 2891 genes, 0.2% | 13 out of 15382 genes, 0.1% | 1 |  |
| histone H4-K8 acetylation | 5 out of 2891 genes, 0.2% | 13 out of 15382 genes, 0.1% | 1 |  |
| growth hormone receptor signaling pathway | 5 out of 2891 genes, 0.2% | 13 out of 15382 genes, 0.1% | 1 |  |
| response to fungicide | 5 out of 2891 genes, 0.2% | 13 out of 15382 genes, 0.1% | 1 |  |
| negative regulation of adherens junction organization | 5 out of 2891 genes, 0.2% | 13 out of 15382 genes, 0.1% | 1 |  |
| negative regulation of sprouting angiogenesis | 5 out of 2891 genes, 0.2% | 13 out of 15382 genes, 0.1% | 1 |  |
| regulation of metallopeptidase activity | 5 out of 2891 genes, 0.2% | 13 out of 15382 genes, 0.1% | 1 |  |
| regulation of cAMP-dependent protein kinase activity | 5 out of 2891 genes, 0.2% | 13 out of 15382 genes, 0.1% | 1 |  |
| negative regulation of peptidyl-lysine acetylation | 5 out of 2891 genes, 0.2% | 13 out of 15382 genes, 0.1% | 1 |  |
| negative regulation of mitotic cell cycle | 56 out of 2891 genes, 1.9% | 249 out of 15382 genes, 1.6% | 1 |  |
| negative regulation of cell cycle phase transition | 43 out of 2891 genes, 1.5% | 186 out of 15382 genes, 1.2% | 1 |  |
| negative regulation of leukocyte cell-cell adhesion | 24 out of 2891 genes, 0.8% | 96 out of 15382 genes, 0.6% | 1 |  |
| positive regulation of smooth muscle cell migration | 8 out of 2891 genes, 0.3% | 25 out of 15382 genes, 0.2% | 1 |  |
| melanosome organization | 8 out of 2891 genes, 0.3% | 25 out of 15382 genes, 0.2% | 1 |  |
| signal transduction in absence of ligand | 8 out of 2891 genes, 0.3% | 25 out of 15382 genes, 0.2% | 1 |  |
| positive regulation of blood vessel endothelial cell migration | 8 out of 2891 genes, 0.3% | 25 out of 15382 genes, 0.2% | 1 |  |
| pigment granule organization | 8 out of 2891 genes, 0.3% | 25 out of 15382 genes, 0.2% | 1 |  |
| cardiac muscle tissue growth | 8 out of 2891 genes, 0.3% | 25 out of 15382 genes, 0.2% | 1 |  |
| extrinsic apoptotic signaling pathway in absence of ligand | 8 out of 2891 genes, 0.3% | 25 out of 15382 genes, 0.2% | 1 |  |
| regulation of cellular senescence | 8 out of 2891 genes, 0.3% | 25 out of 15382 genes, 0.2% | 1 |  |
| JAK-STAT cascade | 12 out of 2891 genes, 0.4% | 42 out of 15382 genes, 0.3% | 1 |  |
| regulation of lipid catabolic process | 12 out of 2891 genes, 0.4% | 42 out of 15382 genes, 0.3% | 1 |  |
| regulation of filopodium assembly | 12 out of 2891 genes, 0.4% | 42 out of 15382 genes, 0.3% | 1 |  |
| STAT cascade | 12 out of 2891 genes, 0.4% | 42 out of 15382 genes, 0.3% | 1 |  |
| positive regulation of cell adhesion | 77 out of 2891 genes, 2.7% | 353 out of 15382 genes, 2.3% | 1 |  |
| membrane protein ectodomain proteolysis | 6 out of 2891 genes, 0.2% | 17 out of 15382 genes, 0.1% | 1 |  |
| protein targeting to lysosome | 6 out of 2891 genes, 0.2% | 17 out of 15382 genes, 0.1% | 1 |  |
| negative regulation of tumor necrosis factor-mediated signaling pathway | 6 out of 2891 genes, 0.2% | 17 out of 15382 genes, 0.1% | 1 |  |
| peroxisomal transport | 6 out of 2891 genes, 0.2% | 17 out of 15382 genes, 0.1% | 1 |  |
| relaxation of muscle | 6 out of 2891 genes, 0.2% | 17 out of 15382 genes, 0.1% | 1 |  |
| regulation of platelet aggregation | 6 out of 2891 genes, 0.2% | 17 out of 15382 genes, 0.1% | 1 |  |
| regulation of protein polyubiquitination | 6 out of 2891 genes, 0.2% | 17 out of 15382 genes, 0.1% | 1 |  |
| glycogen biosynthetic process | 7 out of 2891 genes, 0.2% | 21 out of 15382 genes, 0.1% | 1 |  |
| RNA-dependent DNA biosynthetic process | 7 out of 2891 genes, 0.2% | 21 out of 15382 genes, 0.1% | 1 |  |
| glucan biosynthetic process | 7 out of 2891 genes, 0.2% | 21 out of 15382 genes, 0.1% | 1 |  |
| positive regulation of heart rate | 7 out of 2891 genes, 0.2% | 21 out of 15382 genes, 0.1% | 1 |  |
| regulation of vascular endothelial growth factor receptor signaling pathway | 7 out of 2891 genes, 0.2% | 21 out of 15382 genes, 0.1% | 1 |  |
| regulation of histone deacetylation | 7 out of 2891 genes, 0.2% | 21 out of 15382 genes, 0.1% | 1 |  |
| regulation of microtubule depolymerization | 7 out of 2891 genes, 0.2% | 21 out of 15382 genes, 0.1% | 1 |  |
| positive regulation of microtubule polymerization | 7 out of 2891 genes, 0.2% | 21 out of 15382 genes, 0.1% | 1 |  |
| protein localization to Golgi apparatus | 7 out of 2891 genes, 0.2% | 21 out of 15382 genes, 0.1% | 1 |  |
| negative regulation of protein dephosphorylation | 7 out of 2891 genes, 0.2% | 21 out of 15382 genes, 0.1% | 1 |  |
| positive T cell selection | 7 out of 2891 genes, 0.2% | 21 out of 15382 genes, 0.1% | 1 |  |
| regulation of lipoprotein lipase activity | 7 out of 2891 genes, 0.2% | 21 out of 15382 genes, 0.1% | 1 |  |
| negative regulation of protein tyrosine kinase activity | 7 out of 2891 genes, 0.2% | 21 out of 15382 genes, 0.1% | 1 |  |
| negative regulation of protein localization to cell periphery | 7 out of 2891 genes, 0.2% | 21 out of 15382 genes, 0.1% | 1 |  |
| negative regulation of response to drug | 7 out of 2891 genes, 0.2% | 21 out of 15382 genes, 0.1% | 1 |  |
| regulation of cysteine-type endopeptidase activity involved in apoptotic signaling pathway | 7 out of 2891 genes, 0.2% | 21 out of 15382 genes, 0.1% | 1 |  |
| regulation of ATP metabolic process | 20 out of 2891 genes, 0.7% | 78 out of 15382 genes, 0.5% | 1 |  |
| mitochondrion organization | 82 out of 2891 genes, 2.8% | 378 out of 15382 genes, 2.5% | 1 |  |
| glycoprotein biosynthetic process | 66 out of 2891 genes, 2.3% | 299 out of 15382 genes, 1.9% | 1 |  |
| response to wounding | 104 out of 2891 genes, 3.6% | 488 out of 15382 genes, 3.2% | 1 |  |
| cellular glucan metabolic process | 14 out of 2891 genes, 0.5% | 51 out of 15382 genes, 0.3% | 1 |  |
| glucan metabolic process | 14 out of 2891 genes, 0.5% | 51 out of 15382 genes, 0.3% | 1 |  |
| positive regulation of GTPase activity | 48 out of 2891 genes, 1.7% | 211 out of 15382 genes, 1.4% | 1 |  |
| regulation of response to oxidative stress | 18 out of 2891 genes, 0.6% | 69 out of 15382 genes, 0.4% | 1 |  |
| regulation of mitotic recombination | 3 out of 2891 genes, 0.1% | 6 out of 15382 genes, 0.0% | 1 |  |
| pyrimidine dimer repair | 3 out of 2891 genes, 0.1% | 6 out of 15382 genes, 0.0% | 1 |  |
| chromatin silencing at telomere | 3 out of 2891 genes, 0.1% | 6 out of 15382 genes, 0.0% | 1 |  |
| regulation of Schwann cell proliferation | 3 out of 2891 genes, 0.1% | 6 out of 15382 genes, 0.0% | 1 |  |
| histone dephosphorylation | 3 out of 2891 genes, 0.1% | 6 out of 15382 genes, 0.0% | 1 |  |
| protein geranylgeranylation | 3 out of 2891 genes, 0.1% | 6 out of 15382 genes, 0.0% | 1 |  |
| ubiquitin-dependent SMAD protein catabolic process | 3 out of 2891 genes, 0.1% | 6 out of 15382 genes, 0.0% | 1 |  |
| negative regulation of nuclease activity | 3 out of 2891 genes, 0.1% | 6 out of 15382 genes, 0.0% | 1 |  |
| peripheral nervous system myelin maintenance | 3 out of 2891 genes, 0.1% | 6 out of 15382 genes, 0.0% | 1 |  |
| regulation of microvillus length | 3 out of 2891 genes, 0.1% | 6 out of 15382 genes, 0.0% | 1 |  |
| glial cell apoptotic process | 3 out of 2891 genes, 0.1% | 6 out of 15382 genes, 0.0% | 1 |  |
| sterol import | 3 out of 2891 genes, 0.1% | 6 out of 15382 genes, 0.0% | 1 |  |
| regulation of myosin-light-chain-phosphatase activity | 3 out of 2891 genes, 0.1% | 6 out of 15382 genes, 0.0% | 1 |  |
| cardiolipin acyl-chain remodeling | 3 out of 2891 genes, 0.1% | 6 out of 15382 genes, 0.0% | 1 |  |
| cytoplasmic pattern recognition receptor signaling pathway in response to virus | 3 out of 2891 genes, 0.1% | 6 out of 15382 genes, 0.0% | 1 |  |
| histone H3-S10 phosphorylation | 3 out of 2891 genes, 0.1% | 6 out of 15382 genes, 0.0% | 1 |  |
| multi-organism membrane organization | 3 out of 2891 genes, 0.1% | 6 out of 15382 genes, 0.0% | 1 |  |
| positive regulation of fibroblast growth factor receptor signaling pathway | 3 out of 2891 genes, 0.1% | 6 out of 15382 genes, 0.0% | 1 |  |
| sebaceous gland development | 3 out of 2891 genes, 0.1% | 6 out of 15382 genes, 0.0% | 1 |  |
| negative regulation of actin nucleation | 3 out of 2891 genes, 0.1% | 6 out of 15382 genes, 0.0% | 1 |  |
| regulation of NK T cell differentiation | 3 out of 2891 genes, 0.1% | 6 out of 15382 genes, 0.0% | 1 |  |
| adenine nucleotide transport | 3 out of 2891 genes, 0.1% | 6 out of 15382 genes, 0.0% | 1 |  |
| maintenance of centrosome location | 3 out of 2891 genes, 0.1% | 6 out of 15382 genes, 0.0% | 1 |  |
| establishment of Golgi localization | 3 out of 2891 genes, 0.1% | 6 out of 15382 genes, 0.0% | 1 |  |
| neural plate pattern specification | 3 out of 2891 genes, 0.1% | 6 out of 15382 genes, 0.0% | 1 |  |
| uterus morphogenesis | 3 out of 2891 genes, 0.1% | 6 out of 15382 genes, 0.0% | 1 |  |
| cholesterol import | 3 out of 2891 genes, 0.1% | 6 out of 15382 genes, 0.0% | 1 |  |
| cellular response to leucine | 3 out of 2891 genes, 0.1% | 6 out of 15382 genes, 0.0% | 1 |  |
| cellular response to caffeine | 3 out of 2891 genes, 0.1% | 6 out of 15382 genes, 0.0% | 1 |  |
| cellular response to laminar fluid shear stress | 3 out of 2891 genes, 0.1% | 6 out of 15382 genes, 0.0% | 1 |  |
| podosome assembly | 3 out of 2891 genes, 0.1% | 6 out of 15382 genes, 0.0% | 1 |  |
| multinuclear osteoclast differentiation | 3 out of 2891 genes, 0.1% | 6 out of 15382 genes, 0.0% | 1 |  |
| regulation of tubulin deacetylation | 3 out of 2891 genes, 0.1% | 6 out of 15382 genes, 0.0% | 1 |  |
| positive regulation of cell migration involved in sprouting angiogenesis | 3 out of 2891 genes, 0.1% | 6 out of 15382 genes, 0.0% | 1 |  |
| endoplasmic reticulum membrane organization | 3 out of 2891 genes, 0.1% | 6 out of 15382 genes, 0.0% | 1 |  |
| positive regulation of mitotic cell cycle spindle assembly checkpoint | 3 out of 2891 genes, 0.1% | 6 out of 15382 genes, 0.0% | 1 |  |
| neurotransmitter receptor transport, endosome to plasma membrane | 3 out of 2891 genes, 0.1% | 6 out of 15382 genes, 0.0% | 1 |  |
| positive regulation of extracellular matrix assembly | 3 out of 2891 genes, 0.1% | 6 out of 15382 genes, 0.0% | 1 |  |
| regulation of relaxation of cardiac muscle | 3 out of 2891 genes, 0.1% | 6 out of 15382 genes, 0.0% | 1 |  |
| regulation of receptor clustering | 3 out of 2891 genes, 0.1% | 6 out of 15382 genes, 0.0% | 1 |  |
| positive regulation of protein localization to cell cortex | 3 out of 2891 genes, 0.1% | 6 out of 15382 genes, 0.0% | 1 |  |
| negative regulation of metallopeptidase activity | 3 out of 2891 genes, 0.1% | 6 out of 15382 genes, 0.0% | 1 |  |
| regulation of removal of superoxide radicals | 3 out of 2891 genes, 0.1% | 6 out of 15382 genes, 0.0% | 1 |  |
| positive regulation of lamellipodium morphogenesis | 3 out of 2891 genes, 0.1% | 6 out of 15382 genes, 0.0% | 1 |  |
| regulation of cellular response to oxidative stress | 16 out of 2891 genes, 0.6% | 60 out of 15382 genes, 0.4% | 1 |  |
| neuron projection extension | 16 out of 2891 genes, 0.6% | 60 out of 15382 genes, 0.4% | 1 |  |
| regulation of transmembrane transporter activity | 43 out of 2891 genes, 1.5% | 187 out of 15382 genes, 1.2% | 1 |  |
| antigen processing and presentation of exogenous peptide antigen | 38 out of 2891 genes, 1.3% | 163 out of 15382 genes, 1.1% | 1 |  |
| positive regulation of immunoglobulin production | 11 out of 2891 genes, 0.4% | 38 out of 15382 genes, 0.2% | 1 |  |
| negative regulation of mitochondrion organization | 11 out of 2891 genes, 0.4% | 38 out of 15382 genes, 0.2% | 1 |  |
| microtubule polymerization or depolymerization | 11 out of 2891 genes, 0.4% | 38 out of 15382 genes, 0.2% | 1 |  |
| positive regulation of glucose import | 11 out of 2891 genes, 0.4% | 38 out of 15382 genes, 0.2% | 1 |  |
| thymus development | 11 out of 2891 genes, 0.4% | 38 out of 15382 genes, 0.2% | 1 |  |
| hexose metabolic process | 34 out of 2891 genes, 1.2% | 144 out of 15382 genes, 0.9% | 1 |  |
| positive regulation of leukocyte activation | 61 out of 2891 genes, 2.1% | 275 out of 15382 genes, 1.8% | 1 |  |
| positive regulation of cellular protein catabolic process | 39 out of 2891 genes, 1.3% | 168 out of 15382 genes, 1.1% | 1 |  |
| positive regulation of peptidyl-serine phosphorylation | 21 out of 2891 genes, 0.7% | 83 out of 15382 genes, 0.5% | 1 |  |
| negative regulation of vasculature development | 21 out of 2891 genes, 0.7% | 83 out of 15382 genes, 0.5% | 1 |  |
| regulated exocytosis | 129 out of 2891 genes, 4.5% | 615 out of 15382 genes, 4.0% | 1 |  |
| antigen processing and presentation of peptide antigen | 40 out of 2891 genes, 1.4% | 173 out of 15382 genes, 1.1% | 1 |  |
| negative regulation of mitotic cell cycle phase transition | 41 out of 2891 genes, 1.4% | 178 out of 15382 genes, 1.2% | 1 |  |
| lymphocyte homeostasis | 13 out of 2891 genes, 0.4% | 47 out of 15382 genes, 0.3% | 1 |  |
| regulation of protein acetylation | 13 out of 2891 genes, 0.4% | 47 out of 15382 genes, 0.3% | 1 |  |
| regulation of extrinsic apoptotic signaling pathway via death domain receptors | 13 out of 2891 genes, 0.4% | 47 out of 15382 genes, 0.3% | 1 |  |
| positive regulation of cation channel activity | 13 out of 2891 genes, 0.4% | 47 out of 15382 genes, 0.3% | 1 |  |
| positive regulation of DNA binding transcription factor activity | 48 out of 2891 genes, 1.7% | 212 out of 15382 genes, 1.4% | 1 |  |
| hepaticobiliary system development | 28 out of 2891 genes, 1.0% | 116 out of 15382 genes, 0.8% | 1 |  |
| negative regulation of hemopoiesis | 28 out of 2891 genes, 1.0% | 116 out of 15382 genes, 0.8% | 1 |  |
| regulation of defense response to virus | 17 out of 2891 genes, 0.6% | 65 out of 15382 genes, 0.4% | 1 |  |
| regulation of membrane permeability | 17 out of 2891 genes, 0.6% | 65 out of 15382 genes, 0.4% | 1 |  |
| regulation of protein import into nucleus | 32 out of 2891 genes, 1.1% | 135 out of 15382 genes, 0.9% | 1 |  |
| regulation of mitochondrial membrane potential | 15 out of 2891 genes, 0.5% | 56 out of 15382 genes, 0.4% | 1 |  |
| cellular response to amino acid stimulus | 15 out of 2891 genes, 0.5% | 56 out of 15382 genes, 0.4% | 1 |  |
| regulation of glucose metabolic process | 22 out of 2891 genes, 0.8% | 88 out of 15382 genes, 0.6% | 1 |  |
| peptidyl-lysine acetylation | 22 out of 2891 genes, 0.8% | 88 out of 15382 genes, 0.6% | 1 |  |
| RNA processing | 161 out of 2891 genes, 5.6% | 778 out of 15382 genes, 5.1% | 1 |  |
| renal water homeostasis | 10 out of 2891 genes, 0.3% | 34 out of 15382 genes, 0.2% | 1 |  |
| T cell selection | 10 out of 2891 genes, 0.3% | 34 out of 15382 genes, 0.2% | 1 |  |
| organic acid transport | 49 out of 2891 genes, 1.7% | 217 out of 15382 genes, 1.4% | 1 |  |
| carboxylic acid transport | 49 out of 2891 genes, 1.7% | 217 out of 15382 genes, 1.4% | 1 |  |
| regulation of steroid metabolic process | 25 out of 2891 genes, 0.9% | 102 out of 15382 genes, 0.7% | 1 |  |
| positive regulation of transport | 181 out of 2891 genes, 6.3% | 880 out of 15382 genes, 5.7% | 1 |  |
| negative regulation of neurogenesis | 50 out of 2891 genes, 1.7% | 222 out of 15382 genes, 1.4% | 1 |  |
| cellular response to hormone stimulus | 104 out of 2891 genes, 3.6% | 490 out of 15382 genes, 3.2% | 1 |  |
| negative regulation of cellular protein localization | 29 out of 2891 genes, 1.0% | 121 out of 15382 genes, 0.8% | 1 |  |
| positive regulation of immune effector process | 38 out of 2891 genes, 1.3% | 164 out of 15382 genes, 1.1% | 1 |  |
| mitochondrial RNA 3'-end processing | 2 out of 2891 genes, 0.1% | 3 out of 15382 genes, 0.0% | 1 |  |
| intestinal D-glucose absorption | 2 out of 2891 genes, 0.1% | 3 out of 15382 genes, 0.0% | 1 |  |
| astrocyte activation involved in immune response | 2 out of 2891 genes, 0.1% | 3 out of 15382 genes, 0.0% | 1 |  |
| T cell activation via T cell receptor contact with antigen bound to MHC molecule on antigen presenting cell | 2 out of 2891 genes, 0.1% | 3 out of 15382 genes, 0.0% | 1 |  |
| germinal center B cell differentiation | 2 out of 2891 genes, 0.1% | 3 out of 15382 genes, 0.0% | 1 |  |
| B cell lineage commitment | 2 out of 2891 genes, 0.1% | 3 out of 15382 genes, 0.0% | 1 |  |
| apical constriction | 2 out of 2891 genes, 0.1% | 3 out of 15382 genes, 0.0% | 1 |  |
| dGTP catabolic process | 2 out of 2891 genes, 0.1% | 3 out of 15382 genes, 0.0% | 1 |  |
| dTMP biosynthetic process | 2 out of 2891 genes, 0.1% | 3 out of 15382 genes, 0.0% | 1 |  |
| extracellular transport | 2 out of 2891 genes, 0.1% | 3 out of 15382 genes, 0.0% | 1 |  |
| endomitotic cell cycle | 2 out of 2891 genes, 0.1% | 3 out of 15382 genes, 0.0% | 1 |  |
| signal transduction downstream of smoothened | 2 out of 2891 genes, 0.1% | 3 out of 15382 genes, 0.0% | 1 |  |
| vitellogenesis | 2 out of 2891 genes, 0.1% | 3 out of 15382 genes, 0.0% | 1 |  |
| negative regulation of neuroblast proliferation | 2 out of 2891 genes, 0.1% | 3 out of 15382 genes, 0.0% | 1 |  |
| courtship behavior | 2 out of 2891 genes, 0.1% | 3 out of 15382 genes, 0.0% | 1 |  |
| pentose-phosphate shunt, oxidative branch | 2 out of 2891 genes, 0.1% | 3 out of 15382 genes, 0.0% | 1 |  |
| regulation of definitive erythrocyte differentiation | 2 out of 2891 genes, 0.1% | 3 out of 15382 genes, 0.0% | 1 |  |
| regulation of plasma membrane long-chain fatty acid transport | 2 out of 2891 genes, 0.1% | 3 out of 15382 genes, 0.0% | 1 |  |
| negative regulation of plasma membrane long-chain fatty acid transport | 2 out of 2891 genes, 0.1% | 3 out of 15382 genes, 0.0% | 1 |  |
| negative regulation of nitric oxide mediated signal transduction | 2 out of 2891 genes, 0.1% | 3 out of 15382 genes, 0.0% | 1 |  |
| diadenosine polyphosphate biosynthetic process | 2 out of 2891 genes, 0.1% | 3 out of 15382 genes, 0.0% | 1 |  |
| diadenosine tetraphosphate metabolic process | 2 out of 2891 genes, 0.1% | 3 out of 15382 genes, 0.0% | 1 |  |
| diadenosine tetraphosphate biosynthetic process | 2 out of 2891 genes, 0.1% | 3 out of 15382 genes, 0.0% | 1 |  |
| meiotic chromosome movement towards spindle pole | 2 out of 2891 genes, 0.1% | 3 out of 15382 genes, 0.0% | 1 |  |
| protein import into peroxisome matrix, translocation | 2 out of 2891 genes, 0.1% | 3 out of 15382 genes, 0.0% | 1 |  |
| N-terminal peptidyl-lysine acetylation | 2 out of 2891 genes, 0.1% | 3 out of 15382 genes, 0.0% | 1 |  |
| peptidyl-cysteine methylation | 2 out of 2891 genes, 0.1% | 3 out of 15382 genes, 0.0% | 1 |  |
| peptidyl-glycine modification | 2 out of 2891 genes, 0.1% | 3 out of 15382 genes, 0.0% | 1 |  |
| uracil metabolic process | 2 out of 2891 genes, 0.1% | 3 out of 15382 genes, 0.0% | 1 |  |
| cerebellum maturation | 2 out of 2891 genes, 0.1% | 3 out of 15382 genes, 0.0% | 1 |  |
| establishment or maintenance of actin cytoskeleton polarity | 2 out of 2891 genes, 0.1% | 3 out of 15382 genes, 0.0% | 1 |  |
| prostaglandin secretion | 2 out of 2891 genes, 0.1% | 3 out of 15382 genes, 0.0% | 1 |  |
| positive regulation of intracellular lipid transport | 2 out of 2891 genes, 0.1% | 3 out of 15382 genes, 0.0% | 1 |  |
| positive regulation of intracellular sterol transport | 2 out of 2891 genes, 0.1% | 3 out of 15382 genes, 0.0% | 1 |  |
| positive regulation of intracellular cholesterol transport | 2 out of 2891 genes, 0.1% | 3 out of 15382 genes, 0.0% | 1 |  |
| nerve growth factor processing | 2 out of 2891 genes, 0.1% | 3 out of 15382 genes, 0.0% | 1 |  |
| bile acid secretion | 2 out of 2891 genes, 0.1% | 3 out of 15382 genes, 0.0% | 1 |  |
| inositol trisphosphate biosynthetic process | 2 out of 2891 genes, 0.1% | 3 out of 15382 genes, 0.0% | 1 |  |
| negative regulation of protein import into nucleus, translocation | 2 out of 2891 genes, 0.1% | 3 out of 15382 genes, 0.0% | 1 |  |
| negative regulation of histone ubiquitination | 2 out of 2891 genes, 0.1% | 3 out of 15382 genes, 0.0% | 1 |  |
| gamma-tubulin complex localization | 2 out of 2891 genes, 0.1% | 3 out of 15382 genes, 0.0% | 1 |  |
| cell-cell adhesion mediated by integrin | 2 out of 2891 genes, 0.1% | 3 out of 15382 genes, 0.0% | 1 |  |
| positive regulation of vascular wound healing | 2 out of 2891 genes, 0.1% | 3 out of 15382 genes, 0.0% | 1 |  |
| sequestering of TGFbeta in extracellular matrix | 2 out of 2891 genes, 0.1% | 3 out of 15382 genes, 0.0% | 1 |  |
| stress granule disassembly | 2 out of 2891 genes, 0.1% | 3 out of 15382 genes, 0.0% | 1 |  |
| platelet-derived growth factor receptor-beta signaling pathway | 2 out of 2891 genes, 0.1% | 3 out of 15382 genes, 0.0% | 1 |  |
| egg coat formation | 2 out of 2891 genes, 0.1% | 3 out of 15382 genes, 0.0% | 1 |  |
| chemokine (C-C motif) ligand 2 secretion | 2 out of 2891 genes, 0.1% | 3 out of 15382 genes, 0.0% | 1 |  |
| RNA import into mitochondrion | 2 out of 2891 genes, 0.1% | 3 out of 15382 genes, 0.0% | 1 |  |
| RIG-I signaling pathway | 2 out of 2891 genes, 0.1% | 3 out of 15382 genes, 0.0% | 1 |  |
| negative stranded viral RNA replication | 2 out of 2891 genes, 0.1% | 3 out of 15382 genes, 0.0% | 1 |  |
| GDP-L-fucose biosynthetic process | 2 out of 2891 genes, 0.1% | 3 out of 15382 genes, 0.0% | 1 |  |
| establishment or maintenance of neuroblast polarity | 2 out of 2891 genes, 0.1% | 3 out of 15382 genes, 0.0% | 1 |  |
| establishment of neuroblast polarity | 2 out of 2891 genes, 0.1% | 3 out of 15382 genes, 0.0% | 1 |  |
| negative regulation of MHC class II biosynthetic process | 2 out of 2891 genes, 0.1% | 3 out of 15382 genes, 0.0% | 1 |  |
| positive regulation of granulocyte macrophage colony-stimulating factor biosynthetic process | 2 out of 2891 genes, 0.1% | 3 out of 15382 genes, 0.0% | 1 |  |
| positive regulation of female receptivity | 2 out of 2891 genes, 0.1% | 3 out of 15382 genes, 0.0% | 1 |  |
| dATP catabolic process | 2 out of 2891 genes, 0.1% | 3 out of 15382 genes, 0.0% | 1 |  |
| dTMP metabolic process | 2 out of 2891 genes, 0.1% | 3 out of 15382 genes, 0.0% | 1 |  |
| alpha-beta T cell proliferation | 2 out of 2891 genes, 0.1% | 3 out of 15382 genes, 0.0% | 1 |  |
| hydroxylysine metabolic process | 2 out of 2891 genes, 0.1% | 3 out of 15382 genes, 0.0% | 1 |  |
| hydroxylysine biosynthetic process | 2 out of 2891 genes, 0.1% | 3 out of 15382 genes, 0.0% | 1 |  |
| regulation of eye pigmentation | 2 out of 2891 genes, 0.1% | 3 out of 15382 genes, 0.0% | 1 |  |
| positive regulation of cyclic-nucleotide phosphodiesterase activity | 2 out of 2891 genes, 0.1% | 3 out of 15382 genes, 0.0% | 1 |  |
| convergent extension involved in organogenesis | 2 out of 2891 genes, 0.1% | 3 out of 15382 genes, 0.0% | 1 |  |
| positive regulation of Fc receptor mediated stimulatory signaling pathway | 2 out of 2891 genes, 0.1% | 3 out of 15382 genes, 0.0% | 1 |  |
| ciliary receptor clustering involved in smoothened signaling pathway | 2 out of 2891 genes, 0.1% | 3 out of 15382 genes, 0.0% | 1 |  |
| vasculogenesis involved in coronary vascular morphogenesis | 2 out of 2891 genes, 0.1% | 3 out of 15382 genes, 0.0% | 1 |  |
| parkin-mediated stimulation of mitophagy in response to mitochondrial depolarization | 2 out of 2891 genes, 0.1% | 3 out of 15382 genes, 0.0% | 1 |  |
| regulation of interleukin-6-mediated signaling pathway | 2 out of 2891 genes, 0.1% | 3 out of 15382 genes, 0.0% | 1 |  |
| negative regulation of adiponectin secretion | 2 out of 2891 genes, 0.1% | 3 out of 15382 genes, 0.0% | 1 |  |
| cellular detoxification of nitrogen compound | 2 out of 2891 genes, 0.1% | 3 out of 15382 genes, 0.0% | 1 |  |
| formin-nucleated actin cable assembly | 2 out of 2891 genes, 0.1% | 3 out of 15382 genes, 0.0% | 1 |  |
| positive regulation of bile acid biosynthetic process | 2 out of 2891 genes, 0.1% | 3 out of 15382 genes, 0.0% | 1 |  |
| regulation of transposon integration | 2 out of 2891 genes, 0.1% | 3 out of 15382 genes, 0.0% | 1 |  |
| negative regulation of transposon integration | 2 out of 2891 genes, 0.1% | 3 out of 15382 genes, 0.0% | 1 |  |
| death-inducing signaling complex assembly | 2 out of 2891 genes, 0.1% | 3 out of 15382 genes, 0.0% | 1 |  |
| histone H3-K27 demethylation | 2 out of 2891 genes, 0.1% | 3 out of 15382 genes, 0.0% | 1 |  |
| chemokine (C-X-C motif) ligand 2 production | 2 out of 2891 genes, 0.1% | 3 out of 15382 genes, 0.0% | 1 |  |
| receptor-mediated endocytosis involved in cholesterol transport | 2 out of 2891 genes, 0.1% | 3 out of 15382 genes, 0.0% | 1 |  |
| chemokine secretion | 2 out of 2891 genes, 0.1% | 3 out of 15382 genes, 0.0% | 1 |  |
| regulation of centromere complex assembly | 2 out of 2891 genes, 0.1% | 3 out of 15382 genes, 0.0% | 1 |  |
| regulation of kinetochore assembly | 2 out of 2891 genes, 0.1% | 3 out of 15382 genes, 0.0% | 1 |  |
| negative regulation of histone H4 acetylation | 2 out of 2891 genes, 0.1% | 3 out of 15382 genes, 0.0% | 1 |  |
| regulation of protein glycosylation in Golgi | 2 out of 2891 genes, 0.1% | 3 out of 15382 genes, 0.0% | 1 |  |
| telomerase RNA stabilization | 2 out of 2891 genes, 0.1% | 3 out of 15382 genes, 0.0% | 1 |  |
| establishment of protein localization to endoplasmic reticulum membrane | 2 out of 2891 genes, 0.1% | 3 out of 15382 genes, 0.0% | 1 |  |
| fasciculation of sensory neuron axon | 2 out of 2891 genes, 0.1% | 3 out of 15382 genes, 0.0% | 1 |  |
| circadian regulation of translation | 2 out of 2891 genes, 0.1% | 3 out of 15382 genes, 0.0% | 1 |  |
| hypoxia-inducible factor-1alpha signaling pathway | 2 out of 2891 genes, 0.1% | 3 out of 15382 genes, 0.0% | 1 |  |
| histone H3-K36 dimethylation | 2 out of 2891 genes, 0.1% | 3 out of 15382 genes, 0.0% | 1 |  |
| modification of synaptic structure | 2 out of 2891 genes, 0.1% | 3 out of 15382 genes, 0.0% | 1 |  |
| formin-nucleated actin cable organization | 2 out of 2891 genes, 0.1% | 3 out of 15382 genes, 0.0% | 1 |  |
| regulation of monocyte aggregation | 2 out of 2891 genes, 0.1% | 3 out of 15382 genes, 0.0% | 1 |  |
| positive regulation of monocyte aggregation | 2 out of 2891 genes, 0.1% | 3 out of 15382 genes, 0.0% | 1 |  |
| regulation of histone H2A K63-linked ubiquitination | 2 out of 2891 genes, 0.1% | 3 out of 15382 genes, 0.0% | 1 |  |
| negative regulation of histone H2A K63-linked ubiquitination | 2 out of 2891 genes, 0.1% | 3 out of 15382 genes, 0.0% | 1 |  |
| negative regulation of peptidyl-serine dephosphorylation | 2 out of 2891 genes, 0.1% | 3 out of 15382 genes, 0.0% | 1 |  |
| mitotic cytokinetic process | 2 out of 2891 genes, 0.1% | 3 out of 15382 genes, 0.0% | 1 |  |
| positive regulation of actin filament-based movement | 2 out of 2891 genes, 0.1% | 3 out of 15382 genes, 0.0% | 1 |  |
| negative regulation of wound healing, spreading of epidermal cells | 2 out of 2891 genes, 0.1% | 3 out of 15382 genes, 0.0% | 1 |  |
| regulation of voltage-gated potassium channel activity involved in ventricular cardiac muscle cell action potential repolarization | 2 out of 2891 genes, 0.1% | 3 out of 15382 genes, 0.0% | 1 |  |
| positive regulation of bile acid metabolic process | 2 out of 2891 genes, 0.1% | 3 out of 15382 genes, 0.0% | 1 |  |
| regulation of myofibroblast differentiation | 2 out of 2891 genes, 0.1% | 3 out of 15382 genes, 0.0% | 1 |  |
| regulation of membrane repolarization during ventricular cardiac muscle cell action potential | 2 out of 2891 genes, 0.1% | 3 out of 15382 genes, 0.0% | 1 |  |
| regulation of membrane repolarization during cardiac muscle cell action potential | 2 out of 2891 genes, 0.1% | 3 out of 15382 genes, 0.0% | 1 |  |
| regulation of voltage-gated sodium channel activity | 2 out of 2891 genes, 0.1% | 3 out of 15382 genes, 0.0% | 1 |  |
| regulation of presynapse assembly | 2 out of 2891 genes, 0.1% | 3 out of 15382 genes, 0.0% | 1 |  |
| response to insulin-like growth factor stimulus | 2 out of 2891 genes, 0.1% | 3 out of 15382 genes, 0.0% | 1 |  |
| positive regulation of T-helper 17 cell lineage commitment | 2 out of 2891 genes, 0.1% | 3 out of 15382 genes, 0.0% | 1 |  |
| regulation of chemokine (C-X-C motif) ligand 1 production | 2 out of 2891 genes, 0.1% | 3 out of 15382 genes, 0.0% | 1 |  |
| negative regulation of natural killer cell chemotaxis | 2 out of 2891 genes, 0.1% | 3 out of 15382 genes, 0.0% | 1 |  |
| regulation of histone H4-K16 acetylation | 2 out of 2891 genes, 0.1% | 3 out of 15382 genes, 0.0% | 1 |  |
| negative regulation of receptor catabolic process | 2 out of 2891 genes, 0.1% | 3 out of 15382 genes, 0.0% | 1 |  |
| regulation of dense core granule biogenesis | 2 out of 2891 genes, 0.1% | 3 out of 15382 genes, 0.0% | 1 |  |
| positive regulation of transcription from RNA polymerase II promoter involved in smooth muscle cell differentiation | 2 out of 2891 genes, 0.1% | 3 out of 15382 genes, 0.0% | 1 |  |
| negative regulation of steroid hormone secretion | 2 out of 2891 genes, 0.1% | 3 out of 15382 genes, 0.0% | 1 |  |
| negative regulation of corticosteroid hormone secretion | 2 out of 2891 genes, 0.1% | 3 out of 15382 genes, 0.0% | 1 |  |
| negative regulation of glucocorticoid secretion | 2 out of 2891 genes, 0.1% | 3 out of 15382 genes, 0.0% | 1 |  |
| regulation of histone H3-K79 methylation | 2 out of 2891 genes, 0.1% | 3 out of 15382 genes, 0.0% | 1 |  |
| regulation of microtubule-based process | 40 out of 2891 genes, 1.4% | 174 out of 15382 genes, 1.1% | 1 |  |
| response to oxidative stress | 71 out of 2891 genes, 2.5% | 326 out of 15382 genes, 2.1% | 1 |  |
| protein N-linked glycosylation via asparagine | 12 out of 2891 genes, 0.4% | 43 out of 15382 genes, 0.3% | 1 |  |
| regulation of leukocyte degranulation | 12 out of 2891 genes, 0.4% | 43 out of 15382 genes, 0.3% | 1 |  |
| positive regulation of neuron apoptotic process | 12 out of 2891 genes, 0.4% | 43 out of 15382 genes, 0.3% | 1 |  |
| negative regulation of transcription factor import into nucleus | 9 out of 2891 genes, 0.3% | 30 out of 15382 genes, 0.2% | 1 |  |
| positive regulation of cardiac muscle tissue development | 9 out of 2891 genes, 0.3% | 30 out of 15382 genes, 0.2% | 1 |  |
| positive regulation of substrate adhesion-dependent cell spreading | 9 out of 2891 genes, 0.3% | 30 out of 15382 genes, 0.2% | 1 |  |
| developmental process | 1022 out of 2891 genes, 35.4% | 5275 out of 15382 genes, 34.3% | 1 |  |
| response to monosaccharide | 31 out of 2891 genes, 1.1% | 131 out of 15382 genes, 0.9% | 1 |  |
| negative regulation of transporter activity | 16 out of 2891 genes, 0.6% | 61 out of 15382 genes, 0.4% | 1 |  |
| regulation of cardiac muscle contraction | 16 out of 2891 genes, 0.6% | 61 out of 15382 genes, 0.4% | 1 |  |
| myotube differentiation | 14 out of 2891 genes, 0.5% | 52 out of 15382 genes, 0.3% | 1 |  |
| positive regulation of viral life cycle | 14 out of 2891 genes, 0.5% | 52 out of 15382 genes, 0.3% | 1 |  |
| negative regulation of intrinsic apoptotic signaling pathway | 21 out of 2891 genes, 0.7% | 84 out of 15382 genes, 0.5% | 1 |  |
| regulation of ion transmembrane transporter activity | 42 out of 2891 genes, 1.5% | 184 out of 15382 genes, 1.2% | 1 |  |
| regulation of intracellular protein transport | 50 out of 2891 genes, 1.7% | 223 out of 15382 genes, 1.4% | 1 |  |
| chromosome segregation | 63 out of 2891 genes, 2.2% | 287 out of 15382 genes, 1.9% | 1 |  |
| cardiac muscle tissue development | 32 out of 2891 genes, 1.1% | 136 out of 15382 genes, 0.9% | 1 |  |
| negative regulation of nervous system development | 53 out of 2891 genes, 1.8% | 238 out of 15382 genes, 1.5% | 1 |  |
| negative regulation of mRNA metabolic process | 19 out of 2891 genes, 0.7% | 75 out of 15382 genes, 0.5% | 1 |  |
| mRNA splice site selection | 8 out of 2891 genes, 0.3% | 26 out of 15382 genes, 0.2% | 1 |  |
| cellular component disassembly involved in execution phase of apoptosis | 8 out of 2891 genes, 0.3% | 26 out of 15382 genes, 0.2% | 1 |  |
| histone monoubiquitination | 8 out of 2891 genes, 0.3% | 26 out of 15382 genes, 0.2% | 1 |  |
| regulation of endothelial cell differentiation | 8 out of 2891 genes, 0.3% | 26 out of 15382 genes, 0.2% | 1 |  |
| protein localization to lysosome | 8 out of 2891 genes, 0.3% | 26 out of 15382 genes, 0.2% | 1 |  |
| cellular response to monoamine stimulus | 8 out of 2891 genes, 0.3% | 26 out of 15382 genes, 0.2% | 1 |  |
| cellular response to catecholamine stimulus | 8 out of 2891 genes, 0.3% | 26 out of 15382 genes, 0.2% | 1 |  |
| regulation of cardiac muscle cell contraction | 8 out of 2891 genes, 0.3% | 26 out of 15382 genes, 0.2% | 1 |  |
| positive regulation of protein targeting to membrane | 8 out of 2891 genes, 0.3% | 26 out of 15382 genes, 0.2% | 1 |  |
| response to abiotic stimulus | 207 out of 2891 genes, 7.2% | 1016 out of 15382 genes, 6.6% | 1 |  |
| response to glucose | 29 out of 2891 genes, 1.0% | 122 out of 15382 genes, 0.8% | 1 |  |
| regulation of DNA-templated transcription, elongation | 11 out of 2891 genes, 0.4% | 39 out of 15382 genes, 0.3% | 1 |  |
| sarcomere organization | 11 out of 2891 genes, 0.4% | 39 out of 15382 genes, 0.3% | 1 |  |
| regulation of protein serine/threonine kinase activity | 88 out of 2891 genes, 3.0% | 412 out of 15382 genes, 2.7% | 1 |  |
| retrograde transport, vesicle recycling within Golgi | 4 out of 2891 genes, 0.1% | 10 out of 15382 genes, 0.1% | 1 |  |
| creatine metabolic process | 4 out of 2891 genes, 0.1% | 10 out of 15382 genes, 0.1% | 1 |  |
| protein prenylation | 4 out of 2891 genes, 0.1% | 10 out of 15382 genes, 0.1% | 1 |  |
| lateral ventricle development | 4 out of 2891 genes, 0.1% | 10 out of 15382 genes, 0.1% | 1 |  |
| positive regulation of vascular endothelial growth factor receptor signaling pathway | 4 out of 2891 genes, 0.1% | 10 out of 15382 genes, 0.1% | 1 |  |
| bleb assembly | 4 out of 2891 genes, 0.1% | 10 out of 15382 genes, 0.1% | 1 |  |
| histone H2B ubiquitination | 4 out of 2891 genes, 0.1% | 10 out of 15382 genes, 0.1% | 1 |  |
| endosomal vesicle fusion | 4 out of 2891 genes, 0.1% | 10 out of 15382 genes, 0.1% | 1 |  |
| vesicle fusion with Golgi apparatus | 4 out of 2891 genes, 0.1% | 10 out of 15382 genes, 0.1% | 1 |  |
| positive regulation of membrane protein ectodomain proteolysis | 4 out of 2891 genes, 0.1% | 10 out of 15382 genes, 0.1% | 1 |  |
| actin filament network formation | 4 out of 2891 genes, 0.1% | 10 out of 15382 genes, 0.1% | 1 |  |
| convergent extension | 4 out of 2891 genes, 0.1% | 10 out of 15382 genes, 0.1% | 1 |  |
| regulation of sequestering of zinc ion | 4 out of 2891 genes, 0.1% | 10 out of 15382 genes, 0.1% | 1 |  |
| histone H3-K9 modification | 4 out of 2891 genes, 0.1% | 10 out of 15382 genes, 0.1% | 1 |  |
| heterochromatin organization | 4 out of 2891 genes, 0.1% | 10 out of 15382 genes, 0.1% | 1 |  |
| membrane fission | 4 out of 2891 genes, 0.1% | 10 out of 15382 genes, 0.1% | 1 |  |
| prenylation | 4 out of 2891 genes, 0.1% | 10 out of 15382 genes, 0.1% | 1 |  |
| neurotransmitter receptor transport to postsynaptic membrane | 4 out of 2891 genes, 0.1% | 10 out of 15382 genes, 0.1% | 1 |  |
| actin filament-based transport | 4 out of 2891 genes, 0.1% | 10 out of 15382 genes, 0.1% | 1 |  |
| protein localization to postsynaptic membrane | 4 out of 2891 genes, 0.1% | 10 out of 15382 genes, 0.1% | 1 |  |
| establishment of protein localization to postsynaptic membrane | 4 out of 2891 genes, 0.1% | 10 out of 15382 genes, 0.1% | 1 |  |
| extraembryonic membrane development | 4 out of 2891 genes, 0.1% | 10 out of 15382 genes, 0.1% | 1 |  |
| regulation of hepatocyte proliferation | 4 out of 2891 genes, 0.1% | 10 out of 15382 genes, 0.1% | 1 |  |
| positive regulation of proteasomal ubiquitin-dependent protein catabolic process | 17 out of 2891 genes, 0.6% | 66 out of 15382 genes, 0.4% | 1 |  |
| divalent inorganic cation transport | 48 out of 2891 genes, 1.7% | 214 out of 15382 genes, 1.4% | 1 |  |
| response to calcium ion | 26 out of 2891 genes, 0.9% | 108 out of 15382 genes, 0.7% | 1 |  |
| negative regulation of cell proliferation | 128 out of 2891 genes, 4.4% | 614 out of 15382 genes, 4.0% | 1 |  |
| regulation of microtubule polymerization or depolymerization | 15 out of 2891 genes, 0.5% | 57 out of 15382 genes, 0.4% | 1 |  |
| cellular pigmentation | 13 out of 2891 genes, 0.4% | 48 out of 15382 genes, 0.3% | 1 |  |
| positive regulation of peptidyl-threonine phosphorylation | 7 out of 2891 genes, 0.2% | 22 out of 15382 genes, 0.1% | 1 |  |
| positive regulation of histone acetylation | 7 out of 2891 genes, 0.2% | 22 out of 15382 genes, 0.1% | 1 |  |
| positive regulation of CD4-positive, alpha-beta T cell differentiation | 7 out of 2891 genes, 0.2% | 22 out of 15382 genes, 0.1% | 1 |  |
| negative regulation of blood vessel endothelial cell migration | 7 out of 2891 genes, 0.2% | 22 out of 15382 genes, 0.1% | 1 |  |
| regulation of fatty acid oxidation | 7 out of 2891 genes, 0.2% | 22 out of 15382 genes, 0.1% | 1 |  |
| positive regulation of antigen receptor-mediated signaling pathway | 7 out of 2891 genes, 0.2% | 22 out of 15382 genes, 0.1% | 1 |  |
| lung cell differentiation | 7 out of 2891 genes, 0.2% | 22 out of 15382 genes, 0.1% | 1 |  |
| lung epithelial cell differentiation | 7 out of 2891 genes, 0.2% | 22 out of 15382 genes, 0.1% | 1 |  |
| positive regulation of lamellipodium organization | 7 out of 2891 genes, 0.2% | 22 out of 15382 genes, 0.1% | 1 |  |
| epithelial cell apoptotic process | 7 out of 2891 genes, 0.2% | 22 out of 15382 genes, 0.1% | 1 |  |
| regulation of mRNA splicing, via spliceosome | 20 out of 2891 genes, 0.7% | 80 out of 15382 genes, 0.5% | 1 |  |
| cardiocyte differentiation | 23 out of 2891 genes, 0.8% | 94 out of 15382 genes, 0.6% | 1 |  |
| regulation of transcription from RNA polymerase II promoter in response to stress | 23 out of 2891 genes, 0.8% | 94 out of 15382 genes, 0.6% | 1 |  |
| mitotic cell cycle checkpoint | 31 out of 2891 genes, 1.1% | 132 out of 15382 genes, 0.9% | 1 |  |
| positive regulation of protein kinase activity | 91 out of 2891 genes, 3.1% | 428 out of 15382 genes, 2.8% | 1 |  |
| 2-oxoglutarate metabolic process | 6 out of 2891 genes, 0.2% | 18 out of 15382 genes, 0.1% | 1 |  |
| regulation of toll-like receptor 4 signaling pathway | 6 out of 2891 genes, 0.2% | 18 out of 15382 genes, 0.1% | 1 |  |
| phosphatidylethanolamine metabolic process | 6 out of 2891 genes, 0.2% | 18 out of 15382 genes, 0.1% | 1 |  |
| negative regulation of T cell receptor signaling pathway | 6 out of 2891 genes, 0.2% | 18 out of 15382 genes, 0.1% | 1 |  |
| positive regulation of posttranscriptional gene silencing | 6 out of 2891 genes, 0.2% | 18 out of 15382 genes, 0.1% | 1 |  |
| modified amino acid transport | 6 out of 2891 genes, 0.2% | 18 out of 15382 genes, 0.1% | 1 |  |
| regulation of endoplasmic reticulum unfolded protein response | 6 out of 2891 genes, 0.2% | 18 out of 15382 genes, 0.1% | 1 |  |
| positive regulation of response to oxidative stress | 6 out of 2891 genes, 0.2% | 18 out of 15382 genes, 0.1% | 1 |  |
| glutathione biosynthetic process | 5 out of 2891 genes, 0.2% | 14 out of 15382 genes, 0.1% | 1 |  |
| negative regulation of glycoprotein biosynthetic process | 5 out of 2891 genes, 0.2% | 14 out of 15382 genes, 0.1% | 1 |  |
| corpus callosum development | 5 out of 2891 genes, 0.2% | 14 out of 15382 genes, 0.1% | 1 |  |
| negative regulation of protein oligomerization | 5 out of 2891 genes, 0.2% | 14 out of 15382 genes, 0.1% | 1 |  |
| fatty acid beta-oxidation using acyl-CoA oxidase | 5 out of 2891 genes, 0.2% | 14 out of 15382 genes, 0.1% | 1 |  |
| diol biosynthetic process | 5 out of 2891 genes, 0.2% | 14 out of 15382 genes, 0.1% | 1 |  |
| cytoplasmic sequestering of transcription factor | 5 out of 2891 genes, 0.2% | 14 out of 15382 genes, 0.1% | 1 |  |
| positive regulation of bone resorption | 5 out of 2891 genes, 0.2% | 14 out of 15382 genes, 0.1% | 1 |  |
| positive regulation of bone remodeling | 5 out of 2891 genes, 0.2% | 14 out of 15382 genes, 0.1% | 1 |  |
| paraxial mesoderm development | 5 out of 2891 genes, 0.2% | 14 out of 15382 genes, 0.1% | 1 |  |
| barbed-end actin filament capping | 5 out of 2891 genes, 0.2% | 14 out of 15382 genes, 0.1% | 1 |  |
| regulation of ventricular cardiac muscle cell membrane repolarization | 5 out of 2891 genes, 0.2% | 14 out of 15382 genes, 0.1% | 1 |  |
| mammary gland epithelial cell differentiation | 5 out of 2891 genes, 0.2% | 14 out of 15382 genes, 0.1% | 1 |  |
| cellular response to growth hormone stimulus | 5 out of 2891 genes, 0.2% | 14 out of 15382 genes, 0.1% | 1 |  |
| cellular response to nitric oxide | 5 out of 2891 genes, 0.2% | 14 out of 15382 genes, 0.1% | 1 |  |
| epithelial cell-cell adhesion | 5 out of 2891 genes, 0.2% | 14 out of 15382 genes, 0.1% | 1 |  |
| positive regulation of oxidative stress-induced cell death | 5 out of 2891 genes, 0.2% | 14 out of 15382 genes, 0.1% | 1 |  |
| regulation of cytoplasmic transport | 5 out of 2891 genes, 0.2% | 14 out of 15382 genes, 0.1% | 1 |  |
| negative regulation of cell cycle process | 55 out of 2891 genes, 1.9% | 249 out of 15382 genes, 1.6% | 1 |  |
| mitochondrial transmembrane transport | 18 out of 2891 genes, 0.6% | 71 out of 15382 genes, 0.5% | 1 |  |
| positive regulation of cell-substrate adhesion | 24 out of 2891 genes, 0.8% | 99 out of 15382 genes, 0.6% | 1 |  |
| response to stimulus | 1383 out of 2891 genes, 47.8% | 7195 out of 15382 genes, 46.8% | 1 |  |
| RNA catabolic process | 45 out of 2891 genes, 1.6% | 200 out of 15382 genes, 1.3% | 1 |  |
| regulation of phosphatidylinositol 3-kinase signaling | 28 out of 2891 genes, 1.0% | 118 out of 15382 genes, 0.8% | 1 |  |
| activation of MAPKK activity | 10 out of 2891 genes, 0.3% | 35 out of 15382 genes, 0.2% | 1 |  |
| negative regulation of DNA replication | 10 out of 2891 genes, 0.3% | 35 out of 15382 genes, 0.2% | 1 |  |
| negative regulation of endothelial cell migration | 10 out of 2891 genes, 0.3% | 35 out of 15382 genes, 0.2% | 1 |  |
| synaptic vesicle recycling | 10 out of 2891 genes, 0.3% | 35 out of 15382 genes, 0.2% | 1 |  |
| regulation of RNA stability | 33 out of 2891 genes, 1.1% | 142 out of 15382 genes, 0.9% | 1 |  |
| positive regulation of endothelial cell migration | 16 out of 2891 genes, 0.6% | 62 out of 15382 genes, 0.4% | 1 |  |
| negative regulation of protein localization to nucleus | 16 out of 2891 genes, 0.6% | 62 out of 15382 genes, 0.4% | 1 |  |
| morphogenesis of embryonic epithelium | 29 out of 2891 genes, 1.0% | 123 out of 15382 genes, 0.8% | 1 |  |
| DNA alkylation | 12 out of 2891 genes, 0.4% | 44 out of 15382 genes, 0.3% | 1 |  |
| DNA methylation | 12 out of 2891 genes, 0.4% | 44 out of 15382 genes, 0.3% | 1 |  |
| peptidyl-asparagine modification | 12 out of 2891 genes, 0.4% | 44 out of 15382 genes, 0.3% | 1 |  |
| regulation of substrate adhesion-dependent cell spreading | 12 out of 2891 genes, 0.4% | 44 out of 15382 genes, 0.3% | 1 |  |
| regulation of ion homeostasis | 41 out of 2891 genes, 1.4% | 181 out of 15382 genes, 1.2% | 1 |  |
| negative regulation of T cell proliferation | 14 out of 2891 genes, 0.5% | 53 out of 15382 genes, 0.3% | 1 |  |
| cellular response to calcium ion | 14 out of 2891 genes, 0.5% | 53 out of 15382 genes, 0.3% | 1 |  |
| B cell differentiation | 22 out of 2891 genes, 0.8% | 90 out of 15382 genes, 0.6% | 1 |  |
| regulation of innate immune response | 80 out of 2891 genes, 2.8% | 374 out of 15382 genes, 2.4% | 1 |  |
| response to organic cyclic compound | 158 out of 2891 genes, 5.5% | 769 out of 15382 genes, 5.0% | 1 |  |
| negative regulation of protein complex assembly | 26 out of 2891 genes, 0.9% | 109 out of 15382 genes, 0.7% | 1 |  |
| epithelial tube formation | 26 out of 2891 genes, 0.9% | 109 out of 15382 genes, 0.7% | 1 |  |
| regulation of protein phosphorylation | 245 out of 2891 genes, 8.5% | 1216 out of 15382 genes, 7.9% | 1 |  |
| positive regulation of multi-organism process | 37 out of 2891 genes, 1.3% | 162 out of 15382 genes, 1.1% | 1 |  |
| blastocyst formation | 9 out of 2891 genes, 0.3% | 31 out of 15382 genes, 0.2% | 1 |  |
| positive regulation of TOR signaling | 9 out of 2891 genes, 0.3% | 31 out of 15382 genes, 0.2% | 1 |  |
| regulation of cell aging | 9 out of 2891 genes, 0.3% | 31 out of 15382 genes, 0.2% | 1 |  |
| negative regulation of hydrolase activity | 58 out of 2891 genes, 2.0% | 265 out of 15382 genes, 1.7% | 1 |  |
| negative regulation of mononuclear cell proliferation | 17 out of 2891 genes, 0.6% | 67 out of 15382 genes, 0.4% | 1 |  |
| negative regulation of lymphocyte proliferation | 17 out of 2891 genes, 0.6% | 67 out of 15382 genes, 0.4% | 1 |  |
| cell proliferation | 129 out of 2891 genes, 4.5% | 622 out of 15382 genes, 4.0% | 1 |  |
| response to hormone | 160 out of 2891 genes, 5.5% | 780 out of 15382 genes, 5.1% | 1 |  |
| water-soluble vitamin metabolic process | 20 out of 2891 genes, 0.7% | 81 out of 15382 genes, 0.5% | 1 |  |
| fatty acid catabolic process | 20 out of 2891 genes, 0.7% | 81 out of 15382 genes, 0.5% | 1 |  |
| microtubule organizing center organization | 20 out of 2891 genes, 0.7% | 81 out of 15382 genes, 0.5% | 1 |  |
| positive regulation of protein modification process | 221 out of 2891 genes, 7.6% | 1093 out of 15382 genes, 7.1% | 1 |  |
| regulation of mitotic nuclear division | 33 out of 2891 genes, 1.1% | 143 out of 15382 genes, 0.9% | 1 |  |
| tube formation | 28 out of 2891 genes, 1.0% | 119 out of 15382 genes, 0.8% | 1 |  |
| calcium-mediated signaling | 24 out of 2891 genes, 0.8% | 100 out of 15382 genes, 0.7% | 1 |  |
| regulation of DNA-templated transcription in response to stress | 24 out of 2891 genes, 0.8% | 100 out of 15382 genes, 0.7% | 1 |  |
| T cell differentiation in thymus | 11 out of 2891 genes, 0.4% | 40 out of 15382 genes, 0.3% | 1 |  |
| histone H3 acetylation | 11 out of 2891 genes, 0.4% | 40 out of 15382 genes, 0.3% | 1 |  |
| response to interleukin-12 | 13 out of 2891 genes, 0.4% | 49 out of 15382 genes, 0.3% | 1 |  |
| internal peptidyl-lysine acetylation | 21 out of 2891 genes, 0.7% | 86 out of 15382 genes, 0.6% | 1 |  |
| negative regulation of cysteine-type endopeptidase activity | 21 out of 2891 genes, 0.7% | 86 out of 15382 genes, 0.6% | 1 |  |
| regulation of protein depolymerization | 18 out of 2891 genes, 0.6% | 72 out of 15382 genes, 0.5% | 1 |  |
| negative regulation of viral life cycle | 18 out of 2891 genes, 0.6% | 72 out of 15382 genes, 0.5% | 1 |  |
| cell-substrate adhesion | 35 out of 2891 genes, 1.2% | 153 out of 15382 genes, 1.0% | 1 |  |
| protein acetylation | 25 out of 2891 genes, 0.9% | 105 out of 15382 genes, 0.7% | 1 |  |
| leukotriene metabolic process | 8 out of 2891 genes, 0.3% | 27 out of 15382 genes, 0.2% | 1 |  |
| extrinsic apoptotic signaling pathway via death domain receptors | 8 out of 2891 genes, 0.3% | 27 out of 15382 genes, 0.2% | 1 |  |
| lipid storage | 8 out of 2891 genes, 0.3% | 27 out of 15382 genes, 0.2% | 1 |  |
| intrinsic apoptotic signaling pathway in response to endoplasmic reticulum stress | 8 out of 2891 genes, 0.3% | 27 out of 15382 genes, 0.2% | 1 |  |
| beta-catenin-TCF complex assembly | 8 out of 2891 genes, 0.3% | 27 out of 15382 genes, 0.2% | 1 |  |
| T cell receptor signaling pathway | 30 out of 2891 genes, 1.0% | 129 out of 15382 genes, 0.8% | 1 |  |
| RNA splicing | 77 out of 2891 genes, 2.7% | 361 out of 15382 genes, 2.3% | 1 |  |
| regulation of production of molecular mediator of immune response | 26 out of 2891 genes, 0.9% | 110 out of 15382 genes, 0.7% | 1 |  |
| blastocyst development | 16 out of 2891 genes, 0.6% | 63 out of 15382 genes, 0.4% | 1 |  |
| cellular response to monosaccharide stimulus | 16 out of 2891 genes, 0.6% | 63 out of 15382 genes, 0.4% | 1 |  |
| cellular response to hexose stimulus | 16 out of 2891 genes, 0.6% | 63 out of 15382 genes, 0.4% | 1 |  |
| regulation of viral genome replication | 19 out of 2891 genes, 0.7% | 77 out of 15382 genes, 0.5% | 1 |  |
| regulation of protein import | 32 out of 2891 genes, 1.1% | 139 out of 15382 genes, 0.9% | 1 |  |
| G1/S transition of mitotic cell cycle | 27 out of 2891 genes, 0.9% | 115 out of 15382 genes, 0.7% | 1 |  |
| negative regulation of protein polymerization | 14 out of 2891 genes, 0.5% | 54 out of 15382 genes, 0.4% | 1 |  |
| transcription from RNA polymerase III promoter | 10 out of 2891 genes, 0.3% | 36 out of 15382 genes, 0.2% | 1 |  |
| negative regulation of stress-activated MAPK cascade | 10 out of 2891 genes, 0.3% | 36 out of 15382 genes, 0.2% | 1 |  |
| intraciliary transport involved in cilium assembly | 10 out of 2891 genes, 0.3% | 36 out of 15382 genes, 0.2% | 1 |  |
| negative regulation of stress-activated protein kinase signaling cascade | 10 out of 2891 genes, 0.3% | 36 out of 15382 genes, 0.2% | 1 |  |
| regulation of action potential | 10 out of 2891 genes, 0.3% | 36 out of 15382 genes, 0.2% | 1 |  |
| cell junction organization | 41 out of 2891 genes, 1.4% | 183 out of 15382 genes, 1.2% | 1 |  |
| negative regulation of actin filament polymerization | 12 out of 2891 genes, 0.4% | 45 out of 15382 genes, 0.3% | 1 |  |
| regulation of organic acid transport | 12 out of 2891 genes, 0.4% | 45 out of 15382 genes, 0.3% | 1 |  |
| cardiac cell development | 12 out of 2891 genes, 0.4% | 45 out of 15382 genes, 0.3% | 1 |  |
| stem cell proliferation | 12 out of 2891 genes, 0.4% | 45 out of 15382 genes, 0.3% | 1 |  |
| positive regulation of extrinsic apoptotic signaling pathway | 12 out of 2891 genes, 0.4% | 45 out of 15382 genes, 0.3% | 1 |  |
| cellular response to external stimulus | 57 out of 2891 genes, 2.0% | 262 out of 15382 genes, 1.7% | 1 |  |
| regulation of protein modification by small protein conjugation or removal | 57 out of 2891 genes, 2.0% | 262 out of 15382 genes, 1.7% | 1 |  |
| positive regulation of mitochondrion organization | 34 out of 2891 genes, 1.2% | 149 out of 15382 genes, 1.0% | 1 |  |
| regulation of nuclease activity | 7 out of 2891 genes, 0.2% | 23 out of 15382 genes, 0.1% | 1 |  |
| receptor catabolic process | 7 out of 2891 genes, 0.2% | 23 out of 15382 genes, 0.1% | 1 |  |
| blood vessel endothelial cell migration | 7 out of 2891 genes, 0.2% | 23 out of 15382 genes, 0.1% | 1 |  |
| establishment of spindle orientation | 7 out of 2891 genes, 0.2% | 23 out of 15382 genes, 0.1% | 1 |  |
| mitochondrial calcium ion homeostasis | 7 out of 2891 genes, 0.2% | 23 out of 15382 genes, 0.1% | 1 |  |
| negative regulation of plasma membrane bounded cell projection assembly | 7 out of 2891 genes, 0.2% | 23 out of 15382 genes, 0.1% | 1 |  |
| negative regulation of telomere maintenance via telomere lengthening | 7 out of 2891 genes, 0.2% | 23 out of 15382 genes, 0.1% | 1 |  |
| positive regulation of peptidyl-lysine acetylation | 7 out of 2891 genes, 0.2% | 23 out of 15382 genes, 0.1% | 1 |  |
| positive regulation of homeostatic process | 43 out of 2891 genes, 1.5% | 193 out of 15382 genes, 1.3% | 1 |  |
| adherens junction organization | 17 out of 2891 genes, 0.6% | 68 out of 15382 genes, 0.4% | 1 |  |
| negative regulation of cysteine-type endopeptidase activity involved in apoptotic process | 17 out of 2891 genes, 0.6% | 68 out of 15382 genes, 0.4% | 1 |  |
| positive regulation of proteolysis involved in cellular protein catabolic process | 35 out of 2891 genes, 1.2% | 154 out of 15382 genes, 1.0% | 1 |  |
| positive regulation of signal transduction | 262 out of 2891 genes, 9.1% | 1309 out of 15382 genes, 8.5% | 1 |  |
| activation of innate immune response | 46 out of 2891 genes, 1.6% | 208 out of 15382 genes, 1.4% | 1 |  |
| cellular response to radiation | 30 out of 2891 genes, 1.0% | 130 out of 15382 genes, 0.8% | 1 |  |
| establishment of lymphocyte polarity | 3 out of 2891 genes, 0.1% | 7 out of 15382 genes, 0.0% | 1 |  |
| inner cell mass cell differentiation | 3 out of 2891 genes, 0.1% | 7 out of 15382 genes, 0.0% | 1 |  |
| neural plate development | 3 out of 2891 genes, 0.1% | 7 out of 15382 genes, 0.0% | 1 |  |
| marginal zone B cell differentiation | 3 out of 2891 genes, 0.1% | 7 out of 15382 genes, 0.0% | 1 |  |
| pericardium morphogenesis | 3 out of 2891 genes, 0.1% | 7 out of 15382 genes, 0.0% | 1 |  |
| mitochondrial DNA replication | 3 out of 2891 genes, 0.1% | 7 out of 15382 genes, 0.0% | 1 |  |
| regulation of translational termination | 3 out of 2891 genes, 0.1% | 7 out of 15382 genes, 0.0% | 1 |  |
| leucine metabolic process | 3 out of 2891 genes, 0.1% | 7 out of 15382 genes, 0.0% | 1 |  |
| tachykinin receptor signaling pathway | 3 out of 2891 genes, 0.1% | 7 out of 15382 genes, 0.0% | 1 |  |
| dosage compensation | 3 out of 2891 genes, 0.1% | 7 out of 15382 genes, 0.0% | 1 |  |
| regulation of alkaline phosphatase activity | 3 out of 2891 genes, 0.1% | 7 out of 15382 genes, 0.0% | 1 |  |
| purine ribonucleotide transport | 3 out of 2891 genes, 0.1% | 7 out of 15382 genes, 0.0% | 1 |  |
| pantothenate metabolic process | 3 out of 2891 genes, 0.1% | 7 out of 15382 genes, 0.0% | 1 |  |
| peptidyl-lysine hydroxylation | 3 out of 2891 genes, 0.1% | 7 out of 15382 genes, 0.0% | 1 |  |
| hindbrain radial glia guided cell migration | 3 out of 2891 genes, 0.1% | 7 out of 15382 genes, 0.0% | 1 |  |
| vesicle transport along actin filament | 3 out of 2891 genes, 0.1% | 7 out of 15382 genes, 0.0% | 1 |  |
| negative regulation of vascular endothelial growth factor receptor signaling pathway | 3 out of 2891 genes, 0.1% | 7 out of 15382 genes, 0.0% | 1 |  |
| positive regulation of mRNA 3'-end processing | 3 out of 2891 genes, 0.1% | 7 out of 15382 genes, 0.0% | 1 |  |
| heterochromatin assembly | 3 out of 2891 genes, 0.1% | 7 out of 15382 genes, 0.0% | 1 |  |
| regulation of DNA endoreduplication | 3 out of 2891 genes, 0.1% | 7 out of 15382 genes, 0.0% | 1 |  |
| positive regulation of activin receptor signaling pathway | 3 out of 2891 genes, 0.1% | 7 out of 15382 genes, 0.0% | 1 |  |
| regulation of immature T cell proliferation | 3 out of 2891 genes, 0.1% | 7 out of 15382 genes, 0.0% | 1 |  |
| positive regulation of histone phosphorylation | 3 out of 2891 genes, 0.1% | 7 out of 15382 genes, 0.0% | 1 |  |
| meiotic cytokinesis | 3 out of 2891 genes, 0.1% | 7 out of 15382 genes, 0.0% | 1 |  |
| positive regulation of smooth muscle cell apoptotic process | 3 out of 2891 genes, 0.1% | 7 out of 15382 genes, 0.0% | 1 |  |
| histone H3-K4 demethylation | 3 out of 2891 genes, 0.1% | 7 out of 15382 genes, 0.0% | 1 |  |
| histone H4-K20 methylation | 3 out of 2891 genes, 0.1% | 7 out of 15382 genes, 0.0% | 1 |  |
| common myeloid progenitor cell proliferation | 3 out of 2891 genes, 0.1% | 7 out of 15382 genes, 0.0% | 1 |  |
| nerve growth factor signaling pathway | 3 out of 2891 genes, 0.1% | 7 out of 15382 genes, 0.0% | 1 |  |
| water-soluble vitamin biosynthetic process | 3 out of 2891 genes, 0.1% | 7 out of 15382 genes, 0.0% | 1 |  |
| thiamine-containing compound metabolic process | 3 out of 2891 genes, 0.1% | 7 out of 15382 genes, 0.0% | 1 |  |
| regulation of transcription from RNA polymerase II promoter in response to oxidative stress | 3 out of 2891 genes, 0.1% | 7 out of 15382 genes, 0.0% | 1 |  |
| multi-organism metabolic process | 3 out of 2891 genes, 0.1% | 7 out of 15382 genes, 0.0% | 1 |  |
| positive thymic T cell selection | 3 out of 2891 genes, 0.1% | 7 out of 15382 genes, 0.0% | 1 |  |
| positive regulation of monocyte differentiation | 3 out of 2891 genes, 0.1% | 7 out of 15382 genes, 0.0% | 1 |  |
| autophagic cell death | 3 out of 2891 genes, 0.1% | 7 out of 15382 genes, 0.0% | 1 |  |
| positive regulation of histone H3-K9 methylation | 3 out of 2891 genes, 0.1% | 7 out of 15382 genes, 0.0% | 1 |  |
| Bergmann glial cell differentiation | 3 out of 2891 genes, 0.1% | 7 out of 15382 genes, 0.0% | 1 |  |
| positive regulation of ryanodine-sensitive calcium-release channel activity | 3 out of 2891 genes, 0.1% | 7 out of 15382 genes, 0.0% | 1 |  |
| negative regulation of response to interferon-gamma | 3 out of 2891 genes, 0.1% | 7 out of 15382 genes, 0.0% | 1 |  |
| negative regulation of interferon-gamma-mediated signaling pathway | 3 out of 2891 genes, 0.1% | 7 out of 15382 genes, 0.0% | 1 |  |
| chorio-allantoic fusion | 3 out of 2891 genes, 0.1% | 7 out of 15382 genes, 0.0% | 1 |  |
| peptidyl-serine dephosphorylation | 3 out of 2891 genes, 0.1% | 7 out of 15382 genes, 0.0% | 1 |  |
| rRNA base methylation | 3 out of 2891 genes, 0.1% | 7 out of 15382 genes, 0.0% | 1 |  |
| positive regulation of calcineurin-NFAT signaling cascade | 3 out of 2891 genes, 0.1% | 7 out of 15382 genes, 0.0% | 1 |  |
| skeletal muscle acetylcholine-gated channel clustering | 3 out of 2891 genes, 0.1% | 7 out of 15382 genes, 0.0% | 1 |  |
| cellular response to purine-containing compound | 3 out of 2891 genes, 0.1% | 7 out of 15382 genes, 0.0% | 1 |  |
| establishment of protein localization to Golgi | 3 out of 2891 genes, 0.1% | 7 out of 15382 genes, 0.0% | 1 |  |
| G-protein coupled receptor signaling pathway involved in heart process | 3 out of 2891 genes, 0.1% | 7 out of 15382 genes, 0.0% | 1 |  |
| positive regulation of protein homodimerization activity | 3 out of 2891 genes, 0.1% | 7 out of 15382 genes, 0.0% | 1 |  |
| ruffle assembly | 3 out of 2891 genes, 0.1% | 7 out of 15382 genes, 0.0% | 1 |  |
| asymmetric stem cell division | 3 out of 2891 genes, 0.1% | 7 out of 15382 genes, 0.0% | 1 |  |
| positive regulation of calcineurin-mediated signaling | 3 out of 2891 genes, 0.1% | 7 out of 15382 genes, 0.0% | 1 |  |
| positive regulation of autophagosome maturation | 3 out of 2891 genes, 0.1% | 7 out of 15382 genes, 0.0% | 1 |  |
| negative regulation of anion transmembrane transport | 3 out of 2891 genes, 0.1% | 7 out of 15382 genes, 0.0% | 1 |  |
| regulation of protein localization to cell cortex | 3 out of 2891 genes, 0.1% | 7 out of 15382 genes, 0.0% | 1 |  |
| regulation of guanyl-nucleotide exchange factor activity | 3 out of 2891 genes, 0.1% | 7 out of 15382 genes, 0.0% | 1 |  |
| response to chemokine | 3 out of 2891 genes, 0.1% | 7 out of 15382 genes, 0.0% | 1 |  |
| cellular response to chemokine | 3 out of 2891 genes, 0.1% | 7 out of 15382 genes, 0.0% | 1 |  |
| regulation of peptidyl-cysteine S-nitrosylation | 3 out of 2891 genes, 0.1% | 7 out of 15382 genes, 0.0% | 1 |  |
| negative regulation of fibroblast apoptotic process | 3 out of 2891 genes, 0.1% | 7 out of 15382 genes, 0.0% | 1 |  |
| regulation of regulated secretory pathway | 25 out of 2891 genes, 0.9% | 106 out of 15382 genes, 0.7% | 1 |  |
| response to ketone | 37 out of 2891 genes, 1.3% | 164 out of 15382 genes, 1.1% | 1 |  |
| negative regulation of viral process | 21 out of 2891 genes, 0.7% | 87 out of 15382 genes, 0.6% | 1 |  |
| peptidyl-tyrosine modification | 18 out of 2891 genes, 0.6% | 73 out of 15382 genes, 0.5% | 1 |  |
| monocarboxylic acid transport | 26 out of 2891 genes, 0.9% | 111 out of 15382 genes, 0.7% | 1 |  |
| positive regulation of mesenchymal cell proliferation | 6 out of 2891 genes, 0.2% | 19 out of 15382 genes, 0.1% | 1 |  |
| mature B cell differentiation | 6 out of 2891 genes, 0.2% | 19 out of 15382 genes, 0.1% | 1 |  |
| nucleotide-excision repair, preincision complex assembly | 6 out of 2891 genes, 0.2% | 19 out of 15382 genes, 0.1% | 1 |  |
| positive regulation of interferon-alpha production | 6 out of 2891 genes, 0.2% | 19 out of 15382 genes, 0.1% | 1 |  |
| protein demannosylation | 6 out of 2891 genes, 0.2% | 19 out of 15382 genes, 0.1% | 1 |  |
| protein alpha-1,2-demannosylation | 6 out of 2891 genes, 0.2% | 19 out of 15382 genes, 0.1% | 1 |  |
| positive regulation of mRNA splicing, via spliceosome | 6 out of 2891 genes, 0.2% | 19 out of 15382 genes, 0.1% | 1 |  |
| nucleus localization | 6 out of 2891 genes, 0.2% | 19 out of 15382 genes, 0.1% | 1 |  |
| positive regulation of focal adhesion assembly | 6 out of 2891 genes, 0.2% | 19 out of 15382 genes, 0.1% | 1 |  |
| auditory receptor cell development | 6 out of 2891 genes, 0.2% | 19 out of 15382 genes, 0.1% | 1 |  |
| regulation of mitochondrial fission | 6 out of 2891 genes, 0.2% | 19 out of 15382 genes, 0.1% | 1 |  |
| regulation of execution phase of apoptosis | 6 out of 2891 genes, 0.2% | 19 out of 15382 genes, 0.1% | 1 |  |
| glucosamine-containing compound metabolic process | 6 out of 2891 genes, 0.2% | 19 out of 15382 genes, 0.1% | 1 |  |
| positive regulation of cardiocyte differentiation | 6 out of 2891 genes, 0.2% | 19 out of 15382 genes, 0.1% | 1 |  |
| negative regulation of DNA-dependent DNA replication | 6 out of 2891 genes, 0.2% | 19 out of 15382 genes, 0.1% | 1 |  |
| regulation of cardiac muscle cell differentiation | 6 out of 2891 genes, 0.2% | 19 out of 15382 genes, 0.1% | 1 |  |
| glycoprotein metabolic process | 75 out of 2891 genes, 2.6% | 353 out of 15382 genes, 2.3% | 1 |  |
| positive regulation of cell activation | 61 out of 2891 genes, 2.1% | 283 out of 15382 genes, 1.8% | 1 |  |
| amino sugar metabolic process | 9 out of 2891 genes, 0.3% | 32 out of 15382 genes, 0.2% | 1 |  |
| DNA duplex unwinding | 9 out of 2891 genes, 0.3% | 32 out of 15382 genes, 0.2% | 1 |  |
| spleen development | 9 out of 2891 genes, 0.3% | 32 out of 15382 genes, 0.2% | 1 |  |
| pyridine-containing compound biosynthetic process | 9 out of 2891 genes, 0.3% | 32 out of 15382 genes, 0.2% | 1 |  |
| regulation of triglyceride metabolic process | 9 out of 2891 genes, 0.3% | 32 out of 15382 genes, 0.2% | 1 |  |
| regulation of epidermal cell differentiation | 13 out of 2891 genes, 0.4% | 50 out of 15382 genes, 0.3% | 1 |  |
| negative regulation of transferase activity | 65 out of 2891 genes, 2.2% | 303 out of 15382 genes, 2.0% | 1 |  |
| cell cycle G1/S phase transition | 27 out of 2891 genes, 0.9% | 116 out of 15382 genes, 0.8% | 1 |  |
| protein deacylation | 11 out of 2891 genes, 0.4% | 41 out of 15382 genes, 0.3% | 1 |  |
| positive regulation of DNA repair | 11 out of 2891 genes, 0.4% | 41 out of 15382 genes, 0.3% | 1 |  |
| macromolecule deacylation | 11 out of 2891 genes, 0.4% | 41 out of 15382 genes, 0.3% | 1 |  |
| negative regulation of proteasomal protein catabolic process | 11 out of 2891 genes, 0.4% | 41 out of 15382 genes, 0.3% | 1 |  |
| regulation of cilium assembly | 11 out of 2891 genes, 0.4% | 41 out of 15382 genes, 0.3% | 1 |  |
| negative regulation of catalytic activity | 127 out of 2891 genes, 4.4% | 617 out of 15382 genes, 4.0% | 1 |  |
| mature B cell differentiation involved in immune response | 5 out of 2891 genes, 0.2% | 15 out of 15382 genes, 0.1% | 1 |  |
| phosphatidylethanolamine biosynthetic process | 5 out of 2891 genes, 0.2% | 15 out of 15382 genes, 0.1% | 1 |  |
| negative regulation of glucose transport | 5 out of 2891 genes, 0.2% | 15 out of 15382 genes, 0.1% | 1 |  |
| regulation of ARF protein signal transduction | 5 out of 2891 genes, 0.2% | 15 out of 15382 genes, 0.1% | 1 |  |
| negative regulation of phosphoprotein phosphatase activity | 5 out of 2891 genes, 0.2% | 15 out of 15382 genes, 0.1% | 1 |  |
| fatty acid beta-oxidation using acyl-CoA dehydrogenase | 5 out of 2891 genes, 0.2% | 15 out of 15382 genes, 0.1% | 1 |  |
| megakaryocyte development | 5 out of 2891 genes, 0.2% | 15 out of 15382 genes, 0.1% | 1 |  |
| regulation of protein homodimerization activity | 5 out of 2891 genes, 0.2% | 15 out of 15382 genes, 0.1% | 1 |  |
| positive regulation by host of viral transcription | 5 out of 2891 genes, 0.2% | 15 out of 15382 genes, 0.1% | 1 |  |
| dendritic spine morphogenesis | 5 out of 2891 genes, 0.2% | 15 out of 15382 genes, 0.1% | 1 |  |
| renal absorption | 5 out of 2891 genes, 0.2% | 15 out of 15382 genes, 0.1% | 1 |  |
| positive regulation of cellular response to oxidative stress | 5 out of 2891 genes, 0.2% | 15 out of 15382 genes, 0.1% | 1 |  |
| negative regulation of protein acetylation | 5 out of 2891 genes, 0.2% | 15 out of 15382 genes, 0.1% | 1 |  |
| cellular response to reactive nitrogen species | 5 out of 2891 genes, 0.2% | 15 out of 15382 genes, 0.1% | 1 |  |
| regulation of delayed rectifier potassium channel activity | 5 out of 2891 genes, 0.2% | 15 out of 15382 genes, 0.1% | 1 |  |
| positive regulation of autophagy of mitochondrion | 5 out of 2891 genes, 0.2% | 15 out of 15382 genes, 0.1% | 1 |  |
| carnitine shuttle | 4 out of 2891 genes, 0.1% | 11 out of 15382 genes, 0.1% | 1 |  |
| negative regulation of epidermal growth factor-activated receptor activity | 4 out of 2891 genes, 0.1% | 11 out of 15382 genes, 0.1% | 1 |  |
| dorsal/ventral axis specification | 4 out of 2891 genes, 0.1% | 11 out of 15382 genes, 0.1% | 1 |  |
| regulation of cell communication by electrical coupling | 4 out of 2891 genes, 0.1% | 11 out of 15382 genes, 0.1% | 1 |  |
| positive regulation of triglyceride biosynthetic process | 4 out of 2891 genes, 0.1% | 11 out of 15382 genes, 0.1% | 1 |  |
| regulation of skeletal muscle adaptation | 4 out of 2891 genes, 0.1% | 11 out of 15382 genes, 0.1% | 1 |  |
| peptidyl-arginine methylation | 4 out of 2891 genes, 0.1% | 11 out of 15382 genes, 0.1% | 1 |  |
| nuclear body organization | 4 out of 2891 genes, 0.1% | 11 out of 15382 genes, 0.1% | 1 |  |
| mitochondrial DNA metabolic process | 4 out of 2891 genes, 0.1% | 11 out of 15382 genes, 0.1% | 1 |  |
| negative regulation of DNA-templated transcription, elongation | 4 out of 2891 genes, 0.1% | 11 out of 15382 genes, 0.1% | 1 |  |
| positive regulation of toll-like receptor 4 signaling pathway | 4 out of 2891 genes, 0.1% | 11 out of 15382 genes, 0.1% | 1 |  |
| protein folding in endoplasmic reticulum | 4 out of 2891 genes, 0.1% | 11 out of 15382 genes, 0.1% | 1 |  |
| regulation of hippo signaling | 4 out of 2891 genes, 0.1% | 11 out of 15382 genes, 0.1% | 1 |  |
| negative T cell selection | 4 out of 2891 genes, 0.1% | 11 out of 15382 genes, 0.1% | 1 |  |
| positive regulation of actin nucleation | 4 out of 2891 genes, 0.1% | 11 out of 15382 genes, 0.1% | 1 |  |
| maintenance of organelle location | 4 out of 2891 genes, 0.1% | 11 out of 15382 genes, 0.1% | 1 |  |
| JAK-STAT cascade involved in growth hormone signaling pathway | 4 out of 2891 genes, 0.1% | 11 out of 15382 genes, 0.1% | 1 |  |
| branching involved in labyrinthine layer morphogenesis | 4 out of 2891 genes, 0.1% | 11 out of 15382 genes, 0.1% | 1 |  |
| spongiotrophoblast layer development | 4 out of 2891 genes, 0.1% | 11 out of 15382 genes, 0.1% | 1 |  |
| regulation of mucus secretion | 4 out of 2891 genes, 0.1% | 11 out of 15382 genes, 0.1% | 1 |  |
| negative regulation of cell migration involved in sprouting angiogenesis | 4 out of 2891 genes, 0.1% | 11 out of 15382 genes, 0.1% | 1 |  |
| regulation of synapse maturation | 4 out of 2891 genes, 0.1% | 11 out of 15382 genes, 0.1% | 1 |  |
| negative regulation of release of cytochrome c from mitochondria | 4 out of 2891 genes, 0.1% | 11 out of 15382 genes, 0.1% | 1 |  |
| regulation of ventricular cardiac muscle cell action potential | 4 out of 2891 genes, 0.1% | 11 out of 15382 genes, 0.1% | 1 |  |
| positive regulation of endoplasmic reticulum unfolded protein response | 4 out of 2891 genes, 0.1% | 11 out of 15382 genes, 0.1% | 1 |  |
| negative regulation of potassium ion transmembrane transporter activity | 4 out of 2891 genes, 0.1% | 11 out of 15382 genes, 0.1% | 1 |  |
| regulation of autophagosome maturation | 4 out of 2891 genes, 0.1% | 11 out of 15382 genes, 0.1% | 1 |  |
| negative regulation of autophagosome assembly | 4 out of 2891 genes, 0.1% | 11 out of 15382 genes, 0.1% | 1 |  |
| positive regulation of membrane depolarization | 4 out of 2891 genes, 0.1% | 11 out of 15382 genes, 0.1% | 1 |  |
| positive regulation of autophagosome assembly | 4 out of 2891 genes, 0.1% | 11 out of 15382 genes, 0.1% | 1 |  |
| positive regulation of neuron migration | 4 out of 2891 genes, 0.1% | 11 out of 15382 genes, 0.1% | 1 |  |
| cellular carbohydrate metabolic process | 29 out of 2891 genes, 1.0% | 126 out of 15382 genes, 0.8% | 1 |  |
| cell morphogenesis involved in neuron differentiation | 81 out of 2891 genes, 2.8% | 384 out of 15382 genes, 2.5% | 1 |  |
| DNA-templated transcription, initiation | 37 out of 2891 genes, 1.3% | 165 out of 15382 genes, 1.1% | 1 |  |
| striated muscle tissue development | 55 out of 2891 genes, 1.9% | 254 out of 15382 genes, 1.7% | 1 |  |
| fatty acid beta-oxidation | 14 out of 2891 genes, 0.5% | 55 out of 15382 genes, 0.4% | 1 |  |
| negative regulation of ion transmembrane transporter activity | 14 out of 2891 genes, 0.5% | 55 out of 15382 genes, 0.4% | 1 |  |
| regulation of alcohol biosynthetic process | 14 out of 2891 genes, 0.5% | 55 out of 15382 genes, 0.4% | 1 |  |
| regulation of fibroblast proliferation | 17 out of 2891 genes, 0.6% | 69 out of 15382 genes, 0.4% | 1 |  |
| negative regulation of leukocyte proliferation | 17 out of 2891 genes, 0.6% | 69 out of 15382 genes, 0.4% | 1 |  |
| mammary gland development | 25 out of 2891 genes, 0.9% | 107 out of 15382 genes, 0.7% | 1 |  |
| negative regulation of cellular response to growth factor stimulus | 25 out of 2891 genes, 0.9% | 107 out of 15382 genes, 0.7% | 1 |  |
| placenta development | 31 out of 2891 genes, 1.1% | 136 out of 15382 genes, 0.9% | 1 |  |
| cardiac septum development | 21 out of 2891 genes, 0.7% | 88 out of 15382 genes, 0.6% | 1 |  |
| neural tube patterning | 8 out of 2891 genes, 0.3% | 28 out of 15382 genes, 0.2% | 1 |  |
| positive regulation of bone mineralization | 8 out of 2891 genes, 0.3% | 28 out of 15382 genes, 0.2% | 1 |  |
| negative regulation of microtubule polymerization or depolymerization | 8 out of 2891 genes, 0.3% | 28 out of 15382 genes, 0.2% | 1 |  |
| positive regulation of telomere maintenance via telomerase | 8 out of 2891 genes, 0.3% | 28 out of 15382 genes, 0.2% | 1 |  |
| positive regulation of interleukin-2 production | 8 out of 2891 genes, 0.3% | 28 out of 15382 genes, 0.2% | 1 |  |
| ERBB2 signaling pathway | 8 out of 2891 genes, 0.3% | 28 out of 15382 genes, 0.2% | 1 |  |
| inner ear auditory receptor cell differentiation | 8 out of 2891 genes, 0.3% | 28 out of 15382 genes, 0.2% | 1 |  |
| regulation of isotype switching | 8 out of 2891 genes, 0.3% | 28 out of 15382 genes, 0.2% | 1 |  |
| synaptic vesicle endocytosis | 8 out of 2891 genes, 0.3% | 28 out of 15382 genes, 0.2% | 1 |  |
| response to monoamine | 8 out of 2891 genes, 0.3% | 28 out of 15382 genes, 0.2% | 1 |  |
| response to catecholamine | 8 out of 2891 genes, 0.3% | 28 out of 15382 genes, 0.2% | 1 |  |
| cell-matrix adhesion | 26 out of 2891 genes, 0.9% | 112 out of 15382 genes, 0.7% | 1 |  |
| regulation of smooth muscle cell migration | 12 out of 2891 genes, 0.4% | 46 out of 15382 genes, 0.3% | 1 |  |
| intraciliary transport | 12 out of 2891 genes, 0.4% | 46 out of 15382 genes, 0.3% | 1 |  |
| nervous system development | 413 out of 2891 genes, 14.3% | 2100 out of 15382 genes, 13.7% | 1 |  |
| positive regulation of adaptive immune response | 18 out of 2891 genes, 0.6% | 74 out of 15382 genes, 0.5% | 1 |  |
| regulation of striated muscle contraction | 18 out of 2891 genes, 0.6% | 74 out of 15382 genes, 0.5% | 1 |  |
| protein complex localization | 18 out of 2891 genes, 0.6% | 74 out of 15382 genes, 0.5% | 1 |  |
| acid secretion | 18 out of 2891 genes, 0.6% | 74 out of 15382 genes, 0.5% | 1 |  |
| nucleotide-excision repair | 22 out of 2891 genes, 0.8% | 93 out of 15382 genes, 0.6% | 1 |  |
| negative regulation of MAP kinase activity | 15 out of 2891 genes, 0.5% | 60 out of 15382 genes, 0.4% | 1 |  |
| cellular response to glucose stimulus | 15 out of 2891 genes, 0.5% | 60 out of 15382 genes, 0.4% | 1 |  |
| RNA splicing, via transesterification reactions with bulged adenosine as nucleophile | 55 out of 2891 genes, 1.9% | 255 out of 15382 genes, 1.7% | 1 |  |
| mRNA splicing, via spliceosome | 55 out of 2891 genes, 1.9% | 255 out of 15382 genes, 1.7% | 1 |  |
| regulation of Wnt signaling pathway | 59 out of 2891 genes, 2.0% | 275 out of 15382 genes, 1.8% | 1 |  |
| heart process | 19 out of 2891 genes, 0.7% | 79 out of 15382 genes, 0.5% | 1 |  |
| negative regulation of blood vessel morphogenesis | 19 out of 2891 genes, 0.7% | 79 out of 15382 genes, 0.5% | 1 |  |
| regulation of cell proliferation | 291 out of 2891 genes, 10.1% | 1466 out of 15382 genes, 9.5% | 1 |  |
| cellular response to ionizing radiation | 13 out of 2891 genes, 0.4% | 51 out of 15382 genes, 0.3% | 1 |  |
| regulation of peptide hormone secretion | 41 out of 2891 genes, 1.4% | 186 out of 15382 genes, 1.2% | 1 |  |
| N-terminal protein amino acid modification | 7 out of 2891 genes, 0.2% | 24 out of 15382 genes, 0.2% | 1 |  |
| intracellular lipid transport | 7 out of 2891 genes, 0.2% | 24 out of 15382 genes, 0.2% | 1 |  |
| regulation of interferon-alpha production | 7 out of 2891 genes, 0.2% | 24 out of 15382 genes, 0.2% | 1 |  |
| positive regulation of glucose transport | 11 out of 2891 genes, 0.4% | 42 out of 15382 genes, 0.3% | 1 |  |
| regulation of neuron death | 53 out of 2891 genes, 1.8% | 246 out of 15382 genes, 1.6% | 1 |  |
| liver development | 26 out of 2891 genes, 0.9% | 113 out of 15382 genes, 0.7% | 1 |  |
| positive regulation of innate immune response | 58 out of 2891 genes, 2.0% | 271 out of 15382 genes, 1.8% | 1 |  |
| 'de novo' protein folding | 9 out of 2891 genes, 0.3% | 33 out of 15382 genes, 0.2% | 1 |  |
| positive regulation of epithelial to mesenchymal transition | 9 out of 2891 genes, 0.3% | 33 out of 15382 genes, 0.2% | 1 |  |
| T cell proliferation | 9 out of 2891 genes, 0.3% | 33 out of 15382 genes, 0.2% | 1 |  |
| positive regulation of heart contraction | 9 out of 2891 genes, 0.3% | 33 out of 15382 genes, 0.2% | 1 |  |
| regulation of mitochondrial outer membrane permeabilization involved in apoptotic signaling pathway | 9 out of 2891 genes, 0.3% | 33 out of 15382 genes, 0.2% | 1 |  |
| negative regulation of signal transduction in absence of ligand | 9 out of 2891 genes, 0.3% | 33 out of 15382 genes, 0.2% | 1 |  |
| negative regulation of extrinsic apoptotic signaling pathway in absence of ligand | 9 out of 2891 genes, 0.3% | 33 out of 15382 genes, 0.2% | 1 |  |
| positive regulation of ossification | 17 out of 2891 genes, 0.6% | 70 out of 15382 genes, 0.5% | 1 |  |
| maintenance of location in cell | 17 out of 2891 genes, 0.6% | 70 out of 15382 genes, 0.5% | 1 |  |
| regulation of calcium ion transmembrane transporter activity | 17 out of 2891 genes, 0.6% | 70 out of 15382 genes, 0.5% | 1 |  |
| negative regulation of homeostatic process | 27 out of 2891 genes, 0.9% | 118 out of 15382 genes, 0.8% | 1 |  |
| response to alkaloid | 22 out of 2891 genes, 0.8% | 94 out of 15382 genes, 0.6% | 1 |  |
| developmental growth involved in morphogenesis | 23 out of 2891 genes, 0.8% | 99 out of 15382 genes, 0.6% | 1 |  |
| negative regulation of microtubule depolymerization | 6 out of 2891 genes, 0.2% | 20 out of 15382 genes, 0.1% | 1 |  |
| histone H2A ubiquitination | 6 out of 2891 genes, 0.2% | 20 out of 15382 genes, 0.1% | 1 |  |
| positive regulation of natural killer cell mediated cytotoxicity | 6 out of 2891 genes, 0.2% | 20 out of 15382 genes, 0.1% | 1 |  |
| zinc ion homeostasis | 6 out of 2891 genes, 0.2% | 20 out of 15382 genes, 0.1% | 1 |  |
| retrograde transport, endosome to plasma membrane | 6 out of 2891 genes, 0.2% | 20 out of 15382 genes, 0.1% | 1 |  |
| negative regulation of receptor activity | 6 out of 2891 genes, 0.2% | 20 out of 15382 genes, 0.1% | 1 |  |
| positive regulation of excitatory postsynaptic potential | 6 out of 2891 genes, 0.2% | 20 out of 15382 genes, 0.1% | 1 |  |
| regulation of bicellular tight junction assembly | 6 out of 2891 genes, 0.2% | 20 out of 15382 genes, 0.1% | 1 |  |
| response to cocaine | 12 out of 2891 genes, 0.4% | 47 out of 15382 genes, 0.3% | 1 |  |
| ATP-dependent chromatin remodeling | 15 out of 2891 genes, 0.5% | 61 out of 15382 genes, 0.4% | 1 |  |
| negative regulation of cell development | 55 out of 2891 genes, 1.9% | 257 out of 15382 genes, 1.7% | 1 |  |
| response to radiation | 81 out of 2891 genes, 2.8% | 388 out of 15382 genes, 2.5% | 1 |  |
| apical junction assembly | 10 out of 2891 genes, 0.3% | 38 out of 15382 genes, 0.2% | 1 |  |
| regulation of type I interferon-mediated signaling pathway | 10 out of 2891 genes, 0.3% | 38 out of 15382 genes, 0.2% | 1 |  |
| allantoin metabolic process | 2 out of 2891 genes, 0.1% | 4 out of 15382 genes, 0.0% | 1 |  |
| mRNA 3'-splice site recognition | 2 out of 2891 genes, 0.1% | 4 out of 15382 genes, 0.0% | 1 |  |
| regulation of histone H3-K36 methylation | 2 out of 2891 genes, 0.1% | 4 out of 15382 genes, 0.0% | 1 |  |
| regulation of mitochondrial RNA catabolic process | 2 out of 2891 genes, 0.1% | 4 out of 15382 genes, 0.0% | 1 |  |
| Mullerian duct regression | 2 out of 2891 genes, 0.1% | 4 out of 15382 genes, 0.0% | 1 |  |
| positive regulation of heart rate by epinephrine-norepinephrine | 2 out of 2891 genes, 0.1% | 4 out of 15382 genes, 0.0% | 1 |  |
| regulation of thyroid hormone mediated signaling pathway | 2 out of 2891 genes, 0.1% | 4 out of 15382 genes, 0.0% | 1 |  |
| immunoglobulin transcytosis in epithelial cells | 2 out of 2891 genes, 0.1% | 4 out of 15382 genes, 0.0% | 1 |  |
| tricuspid valve morphogenesis | 2 out of 2891 genes, 0.1% | 4 out of 15382 genes, 0.0% | 1 |  |
| succinyl-CoA metabolic process | 2 out of 2891 genes, 0.1% | 4 out of 15382 genes, 0.0% | 1 |  |
| purine nucleobase catabolic process | 2 out of 2891 genes, 0.1% | 4 out of 15382 genes, 0.0% | 1 |  |
| arginyl-tRNA aminoacylation | 2 out of 2891 genes, 0.1% | 4 out of 15382 genes, 0.0% | 1 |  |
| phenylalanyl-tRNA aminoacylation | 2 out of 2891 genes, 0.1% | 4 out of 15382 genes, 0.0% | 1 |  |
| protein processing involved in protein targeting to mitochondrion | 2 out of 2891 genes, 0.1% | 4 out of 15382 genes, 0.0% | 1 |  |
| triglyceride mobilization | 2 out of 2891 genes, 0.1% | 4 out of 15382 genes, 0.0% | 1 |  |
| thiamine metabolic process | 2 out of 2891 genes, 0.1% | 4 out of 15382 genes, 0.0% | 1 |  |
| DNA damage induced protein phosphorylation | 2 out of 2891 genes, 0.1% | 4 out of 15382 genes, 0.0% | 1 |  |
| post-chaperonin tubulin folding pathway | 2 out of 2891 genes, 0.1% | 4 out of 15382 genes, 0.0% | 1 |  |
| metaphase/anaphase transition of mitotic cell cycle | 2 out of 2891 genes, 0.1% | 4 out of 15382 genes, 0.0% | 1 |  |
| common-partner SMAD protein phosphorylation | 2 out of 2891 genes, 0.1% | 4 out of 15382 genes, 0.0% | 1 |  |
| mesodermal cell migration | 2 out of 2891 genes, 0.1% | 4 out of 15382 genes, 0.0% | 1 |  |
| GDP-mannose biosynthetic process | 2 out of 2891 genes, 0.1% | 4 out of 15382 genes, 0.0% | 1 |  |
| miRNA catabolic process | 2 out of 2891 genes, 0.1% | 4 out of 15382 genes, 0.0% | 1 |  |
| positive regulation of platelet-derived growth factor receptor signaling pathway | 2 out of 2891 genes, 0.1% | 4 out of 15382 genes, 0.0% | 1 |  |
| striated muscle cell apoptotic process | 2 out of 2891 genes, 0.1% | 4 out of 15382 genes, 0.0% | 1 |  |
| cardiac muscle cell apoptotic process | 2 out of 2891 genes, 0.1% | 4 out of 15382 genes, 0.0% | 1 |  |
| positive regulation of transcription via serum response element binding | 2 out of 2891 genes, 0.1% | 4 out of 15382 genes, 0.0% | 1 |  |
| negative regulation of sequestering of triglyceride | 2 out of 2891 genes, 0.1% | 4 out of 15382 genes, 0.0% | 1 |  |
| regulation of skeletal muscle contraction by regulation of release of sequestered calcium ion | 2 out of 2891 genes, 0.1% | 4 out of 15382 genes, 0.0% | 1 |  |
| cadmium ion transport | 2 out of 2891 genes, 0.1% | 4 out of 15382 genes, 0.0% | 1 |  |
| myo-inositol transport | 2 out of 2891 genes, 0.1% | 4 out of 15382 genes, 0.0% | 1 |  |
| L-cystine transport | 2 out of 2891 genes, 0.1% | 4 out of 15382 genes, 0.0% | 1 |  |
| nucleobase transport | 2 out of 2891 genes, 0.1% | 4 out of 15382 genes, 0.0% | 1 |  |
| ATP transport | 2 out of 2891 genes, 0.1% | 4 out of 15382 genes, 0.0% | 1 |  |
| N-acetylneuraminate catabolic process | 2 out of 2891 genes, 0.1% | 4 out of 15382 genes, 0.0% | 1 |  |
| pentose biosynthetic process | 2 out of 2891 genes, 0.1% | 4 out of 15382 genes, 0.0% | 1 |  |
| corpus callosum morphogenesis | 2 out of 2891 genes, 0.1% | 4 out of 15382 genes, 0.0% | 1 |  |
| smoothened signaling pathway involved in ventral spinal cord patterning | 2 out of 2891 genes, 0.1% | 4 out of 15382 genes, 0.0% | 1 |  |
| radial glia guided migration of Purkinje cell | 2 out of 2891 genes, 0.1% | 4 out of 15382 genes, 0.0% | 1 |  |
| PML body organization | 2 out of 2891 genes, 0.1% | 4 out of 15382 genes, 0.0% | 1 |  |
| post-embryonic camera-type eye development | 2 out of 2891 genes, 0.1% | 4 out of 15382 genes, 0.0% | 1 |  |
| positive regulation of translational initiation in response to stress | 2 out of 2891 genes, 0.1% | 4 out of 15382 genes, 0.0% | 1 |  |
| serine transport | 2 out of 2891 genes, 0.1% | 4 out of 15382 genes, 0.0% | 1 |  |
| endosome localization | 2 out of 2891 genes, 0.1% | 4 out of 15382 genes, 0.0% | 1 |  |
| late endosome to vacuole transport via multivesicular body sorting pathway | 2 out of 2891 genes, 0.1% | 4 out of 15382 genes, 0.0% | 1 |  |
| regulation of low-density lipoprotein particle receptor catabolic process | 2 out of 2891 genes, 0.1% | 4 out of 15382 genes, 0.0% | 1 |  |
| negative regulation of glucokinase activity | 2 out of 2891 genes, 0.1% | 4 out of 15382 genes, 0.0% | 1 |  |
| regulation of toll-like receptor 7 signaling pathway | 2 out of 2891 genes, 0.1% | 4 out of 15382 genes, 0.0% | 1 |  |
| negative regulation of Arp2/3 complex-mediated actin nucleation | 2 out of 2891 genes, 0.1% | 4 out of 15382 genes, 0.0% | 1 |  |
| response to carbon monoxide | 2 out of 2891 genes, 0.1% | 4 out of 15382 genes, 0.0% | 1 |  |
| negative regulation of Rac protein signal transduction | 2 out of 2891 genes, 0.1% | 4 out of 15382 genes, 0.0% | 1 |  |
| siRNA loading onto RISC involved in RNA interference | 2 out of 2891 genes, 0.1% | 4 out of 15382 genes, 0.0% | 1 |  |
| post-embryonic hemopoiesis | 2 out of 2891 genes, 0.1% | 4 out of 15382 genes, 0.0% | 1 |  |
| regulation of gluconeogenesis by regulation of transcription from RNA polymerase II promoter | 2 out of 2891 genes, 0.1% | 4 out of 15382 genes, 0.0% | 1 |  |
| maintenance of lens transparency | 2 out of 2891 genes, 0.1% | 4 out of 15382 genes, 0.0% | 1 |  |
| TORC2 signaling | 2 out of 2891 genes, 0.1% | 4 out of 15382 genes, 0.0% | 1 |  |
| xenobiotic transport | 2 out of 2891 genes, 0.1% | 4 out of 15382 genes, 0.0% | 1 |  |
| positive regulation of Golgi to plasma membrane protein transport | 2 out of 2891 genes, 0.1% | 4 out of 15382 genes, 0.0% | 1 |  |
| histone H4-R3 methylation | 2 out of 2891 genes, 0.1% | 4 out of 15382 genes, 0.0% | 1 |  |
| metaphase/anaphase transition of cell cycle | 2 out of 2891 genes, 0.1% | 4 out of 15382 genes, 0.0% | 1 |  |
| type I interferon biosynthetic process | 2 out of 2891 genes, 0.1% | 4 out of 15382 genes, 0.0% | 1 |  |
| regulation of granulocyte macrophage colony-stimulating factor biosynthetic process | 2 out of 2891 genes, 0.1% | 4 out of 15382 genes, 0.0% | 1 |  |
| negative regulation of cell volume | 2 out of 2891 genes, 0.1% | 4 out of 15382 genes, 0.0% | 1 |  |
| negative regulation of mitotic recombination | 2 out of 2891 genes, 0.1% | 4 out of 15382 genes, 0.0% | 1 |  |
| dGTP metabolic process | 2 out of 2891 genes, 0.1% | 4 out of 15382 genes, 0.0% | 1 |  |
| creatinine metabolic process | 2 out of 2891 genes, 0.1% | 4 out of 15382 genes, 0.0% | 1 |  |
| positive regulation of isotype switching to IgA isotypes | 2 out of 2891 genes, 0.1% | 4 out of 15382 genes, 0.0% | 1 |  |
| leukemia inhibitory factor signaling pathway | 2 out of 2891 genes, 0.1% | 4 out of 15382 genes, 0.0% | 1 |  |
| catecholamine secretion | 2 out of 2891 genes, 0.1% | 4 out of 15382 genes, 0.0% | 1 |  |
| negative regulation of filopodium assembly | 2 out of 2891 genes, 0.1% | 4 out of 15382 genes, 0.0% | 1 |  |
| establishment of centrosome localization | 2 out of 2891 genes, 0.1% | 4 out of 15382 genes, 0.0% | 1 |  |
| asymmetric neuroblast division | 2 out of 2891 genes, 0.1% | 4 out of 15382 genes, 0.0% | 1 |  |
| convergent extension involved in gastrulation | 2 out of 2891 genes, 0.1% | 4 out of 15382 genes, 0.0% | 1 |  |
| positive regulation of dopamine receptor signaling pathway | 2 out of 2891 genes, 0.1% | 4 out of 15382 genes, 0.0% | 1 |  |
| negative regulation of transcription initiation from RNA polymerase II promoter | 2 out of 2891 genes, 0.1% | 4 out of 15382 genes, 0.0% | 1 |  |
| regulation of branching involved in prostate gland morphogenesis | 2 out of 2891 genes, 0.1% | 4 out of 15382 genes, 0.0% | 1 |  |
| regulation of ribonuclease activity | 2 out of 2891 genes, 0.1% | 4 out of 15382 genes, 0.0% | 1 |  |
| neural plate regionalization | 2 out of 2891 genes, 0.1% | 4 out of 15382 genes, 0.0% | 1 |  |
| cardiac vascular smooth muscle cell differentiation | 2 out of 2891 genes, 0.1% | 4 out of 15382 genes, 0.0% | 1 |  |
| embryonic heart tube left/right pattern formation | 2 out of 2891 genes, 0.1% | 4 out of 15382 genes, 0.0% | 1 |  |
| regulation of vascular wound healing | 2 out of 2891 genes, 0.1% | 4 out of 15382 genes, 0.0% | 1 |  |
| pancreas morphogenesis | 2 out of 2891 genes, 0.1% | 4 out of 15382 genes, 0.0% | 1 |  |
| T follicular helper cell differentiation | 2 out of 2891 genes, 0.1% | 4 out of 15382 genes, 0.0% | 1 |  |
| regulation of adiponectin secretion | 2 out of 2891 genes, 0.1% | 4 out of 15382 genes, 0.0% | 1 |  |
| regulation of nucleotide-binding oligomerization domain containing 2 signaling pathway | 2 out of 2891 genes, 0.1% | 4 out of 15382 genes, 0.0% | 1 |  |
| cadmium ion transmembrane transport | 2 out of 2891 genes, 0.1% | 4 out of 15382 genes, 0.0% | 1 |  |
| response to indole-3-methanol | 2 out of 2891 genes, 0.1% | 4 out of 15382 genes, 0.0% | 1 |  |
| cellular response to indole-3-methanol | 2 out of 2891 genes, 0.1% | 4 out of 15382 genes, 0.0% | 1 |  |
| regulation of cohesin loading | 2 out of 2891 genes, 0.1% | 4 out of 15382 genes, 0.0% | 1 |  |
| cell proliferation involved in metanephros development | 2 out of 2891 genes, 0.1% | 4 out of 15382 genes, 0.0% | 1 |  |
| rescue of stalled ribosome | 2 out of 2891 genes, 0.1% | 4 out of 15382 genes, 0.0% | 1 |  |
| fibroblast activation | 2 out of 2891 genes, 0.1% | 4 out of 15382 genes, 0.0% | 1 |  |
| interleukin-8 secretion | 2 out of 2891 genes, 0.1% | 4 out of 15382 genes, 0.0% | 1 |  |
| response to sorbitol | 2 out of 2891 genes, 0.1% | 4 out of 15382 genes, 0.0% | 1 |  |
| response to actinomycin D | 2 out of 2891 genes, 0.1% | 4 out of 15382 genes, 0.0% | 1 |  |
| membrane depolarization during AV node cell action potential | 2 out of 2891 genes, 0.1% | 4 out of 15382 genes, 0.0% | 1 |  |
| negative regulation of lipid kinase activity | 2 out of 2891 genes, 0.1% | 4 out of 15382 genes, 0.0% | 1 |  |
| regulation of vesicle size | 2 out of 2891 genes, 0.1% | 4 out of 15382 genes, 0.0% | 1 |  |
| potassium ion export across plasma membrane | 2 out of 2891 genes, 0.1% | 4 out of 15382 genes, 0.0% | 1 |  |
| regulation of presynapse organization | 2 out of 2891 genes, 0.1% | 4 out of 15382 genes, 0.0% | 1 |  |
| positive regulation of peptidyl-tyrosine autophosphorylation | 2 out of 2891 genes, 0.1% | 4 out of 15382 genes, 0.0% | 1 |  |
| negative regulation of vascular endothelial growth factor signaling pathway | 2 out of 2891 genes, 0.1% | 4 out of 15382 genes, 0.0% | 1 |  |
| negative regulation of autophagosome maturation | 2 out of 2891 genes, 0.1% | 4 out of 15382 genes, 0.0% | 1 |  |
| positive regulation of trophoblast cell migration | 2 out of 2891 genes, 0.1% | 4 out of 15382 genes, 0.0% | 1 |  |
| positive regulation of postsynaptic membrane organization | 2 out of 2891 genes, 0.1% | 4 out of 15382 genes, 0.0% | 1 |  |
| positive regulation of histone deacetylase activity | 2 out of 2891 genes, 0.1% | 4 out of 15382 genes, 0.0% | 1 |  |
| regulation of hepatocyte growth factor receptor signaling pathway | 2 out of 2891 genes, 0.1% | 4 out of 15382 genes, 0.0% | 1 |  |
| negative regulation of nuclear cell cycle DNA replication | 2 out of 2891 genes, 0.1% | 4 out of 15382 genes, 0.0% | 1 |  |
| regulation of protein localization to early endosome | 2 out of 2891 genes, 0.1% | 4 out of 15382 genes, 0.0% | 1 |  |
| positive regulation of protein localization to early endosome | 2 out of 2891 genes, 0.1% | 4 out of 15382 genes, 0.0% | 1 |  |
| negative regulation of hexokinase activity | 2 out of 2891 genes, 0.1% | 4 out of 15382 genes, 0.0% | 1 |  |
| negative regulation of bicellular tight junction assembly | 2 out of 2891 genes, 0.1% | 4 out of 15382 genes, 0.0% | 1 |  |
| positive regulation of extracellular exosome assembly | 2 out of 2891 genes, 0.1% | 4 out of 15382 genes, 0.0% | 1 |  |
| negative regulation of p38MAPK cascade | 2 out of 2891 genes, 0.1% | 4 out of 15382 genes, 0.0% | 1 |  |
| regulation of amino acid transmembrane transport | 2 out of 2891 genes, 0.1% | 4 out of 15382 genes, 0.0% | 1 |  |
| negative regulation of PERK-mediated unfolded protein response | 2 out of 2891 genes, 0.1% | 4 out of 15382 genes, 0.0% | 1 |  |
| regulation of establishment of T cell polarity | 2 out of 2891 genes, 0.1% | 4 out of 15382 genes, 0.0% | 1 |  |
| positive regulation of receptor clustering | 2 out of 2891 genes, 0.1% | 4 out of 15382 genes, 0.0% | 1 |  |
| positive regulation of adipose tissue development | 2 out of 2891 genes, 0.1% | 4 out of 15382 genes, 0.0% | 1 |  |
| regulation of Ras GTPase binding | 2 out of 2891 genes, 0.1% | 4 out of 15382 genes, 0.0% | 1 |  |
| regulation of core promoter binding | 2 out of 2891 genes, 0.1% | 4 out of 15382 genes, 0.0% | 1 |  |
| telomerase holoenzyme complex assembly | 2 out of 2891 genes, 0.1% | 4 out of 15382 genes, 0.0% | 1 |  |
| regulation of protein localization to endosome | 2 out of 2891 genes, 0.1% | 4 out of 15382 genes, 0.0% | 1 |  |
| positive regulation of protein localization to endosome | 2 out of 2891 genes, 0.1% | 4 out of 15382 genes, 0.0% | 1 |  |
| positive regulation of spindle assembly | 2 out of 2891 genes, 0.1% | 4 out of 15382 genes, 0.0% | 1 |  |
| negative regulation of amyloid fibril formation | 2 out of 2891 genes, 0.1% | 4 out of 15382 genes, 0.0% | 1 |  |
| positive regulation of T-helper 17 cell differentiation | 2 out of 2891 genes, 0.1% | 4 out of 15382 genes, 0.0% | 1 |  |
| negative regulation of hepatocyte proliferation | 2 out of 2891 genes, 0.1% | 4 out of 15382 genes, 0.0% | 1 |  |
| positive regulation of hepatocyte proliferation | 2 out of 2891 genes, 0.1% | 4 out of 15382 genes, 0.0% | 1 |  |
| negative regulation of platelet-derived growth factor receptor-beta signaling pathway | 2 out of 2891 genes, 0.1% | 4 out of 15382 genes, 0.0% | 1 |  |
| regulation of nuclear-transcribed mRNA catabolic process, nonsense-mediated decay | 2 out of 2891 genes, 0.1% | 4 out of 15382 genes, 0.0% | 1 |  |
| negative regulation of nuclear-transcribed mRNA catabolic process, nonsense-mediated decay | 2 out of 2891 genes, 0.1% | 4 out of 15382 genes, 0.0% | 1 |  |
| regulation of genetic imprinting | 2 out of 2891 genes, 0.1% | 4 out of 15382 genes, 0.0% | 1 |  |
| regulation of Rho guanyl-nucleotide exchange factor activity | 2 out of 2891 genes, 0.1% | 4 out of 15382 genes, 0.0% | 1 |  |
| positive regulation of endocytic recycling | 2 out of 2891 genes, 0.1% | 4 out of 15382 genes, 0.0% | 1 |  |
| regulation of histone H2B ubiquitination | 2 out of 2891 genes, 0.1% | 4 out of 15382 genes, 0.0% | 1 |  |
| positive regulation of histone H2B ubiquitination | 2 out of 2891 genes, 0.1% | 4 out of 15382 genes, 0.0% | 1 |  |
| negative regulation of endothelial cell proliferation | 8 out of 2891 genes, 0.3% | 29 out of 15382 genes, 0.2% | 1 |  |
| blood vessel remodeling | 8 out of 2891 genes, 0.3% | 29 out of 15382 genes, 0.2% | 1 |  |
| glutamate secretion | 8 out of 2891 genes, 0.3% | 29 out of 15382 genes, 0.2% | 1 |  |
| nicotinamide nucleotide biosynthetic process | 8 out of 2891 genes, 0.3% | 29 out of 15382 genes, 0.2% | 1 |  |
| pyridine nucleotide biosynthetic process | 8 out of 2891 genes, 0.3% | 29 out of 15382 genes, 0.2% | 1 |  |
| nucleotide-excision repair, DNA incision | 8 out of 2891 genes, 0.3% | 29 out of 15382 genes, 0.2% | 1 |  |
| cristae formation | 8 out of 2891 genes, 0.3% | 29 out of 15382 genes, 0.2% | 1 |  |
| positive regulation of viral genome replication | 8 out of 2891 genes, 0.3% | 29 out of 15382 genes, 0.2% | 1 |  |
| cellular response to dexamethasone stimulus | 8 out of 2891 genes, 0.3% | 29 out of 15382 genes, 0.2% | 1 |  |
| regulation of protein localization to cell surface | 8 out of 2891 genes, 0.3% | 29 out of 15382 genes, 0.2% | 1 |  |
| regulation of sister chromatid segregation | 16 out of 2891 genes, 0.6% | 66 out of 15382 genes, 0.4% | 1 |  |
| Wnt signaling pathway | 65 out of 2891 genes, 2.2% | 308 out of 15382 genes, 2.0% | 1 |  |
| cell-cell signaling by wnt | 65 out of 2891 genes, 2.2% | 308 out of 15382 genes, 2.0% | 1 |  |
| glutathione metabolic process | 13 out of 2891 genes, 0.4% | 52 out of 15382 genes, 0.3% | 1 |  |
| telomere organization | 21 out of 2891 genes, 0.7% | 90 out of 15382 genes, 0.6% | 1 |  |
| DNA replication | 43 out of 2891 genes, 1.5% | 198 out of 15382 genes, 1.3% | 1 |  |
| RNA splicing, via transesterification reactions | 55 out of 2891 genes, 1.9% | 258 out of 15382 genes, 1.7% | 1 |  |
| nucleotide-excision repair, preincision complex stabilization | 5 out of 2891 genes, 0.2% | 16 out of 15382 genes, 0.1% | 1 |  |
| nucleotide-excision repair, DNA incision, 3'-to lesion | 5 out of 2891 genes, 0.2% | 16 out of 15382 genes, 0.1% | 1 |  |
| cell volume homeostasis | 5 out of 2891 genes, 0.2% | 16 out of 15382 genes, 0.1% | 1 |  |
| peroxisomal membrane transport | 5 out of 2891 genes, 0.2% | 16 out of 15382 genes, 0.1% | 1 |  |
| nonribosomal peptide biosynthetic process | 5 out of 2891 genes, 0.2% | 16 out of 15382 genes, 0.1% | 1 |  |
| telomere localization | 5 out of 2891 genes, 0.2% | 16 out of 15382 genes, 0.1% | 1 |  |
| error-prone translesion synthesis | 5 out of 2891 genes, 0.2% | 16 out of 15382 genes, 0.1% | 1 |  |
| ubiquitin-dependent protein catabolic process via the multivesicular body sorting pathway | 5 out of 2891 genes, 0.2% | 16 out of 15382 genes, 0.1% | 1 |  |
| thymic T cell selection | 5 out of 2891 genes, 0.2% | 16 out of 15382 genes, 0.1% | 1 |  |
| regulation of mitochondrial depolarization | 5 out of 2891 genes, 0.2% | 16 out of 15382 genes, 0.1% | 1 |  |
| endoplasmic reticulum mannose trimming | 5 out of 2891 genes, 0.2% | 16 out of 15382 genes, 0.1% | 1 |  |
| regulation of telomerase RNA localization to Cajal body | 5 out of 2891 genes, 0.2% | 16 out of 15382 genes, 0.1% | 1 |  |
| coenzyme biosynthetic process | 29 out of 2891 genes, 1.0% | 129 out of 15382 genes, 0.8% | 1 |  |
| peptidyl-tyrosine phosphorylation | 17 out of 2891 genes, 0.6% | 71 out of 15382 genes, 0.5% | 1 |  |
| regulation of mRNA stability | 30 out of 2891 genes, 1.0% | 134 out of 15382 genes, 0.9% | 1 |  |
| stimulatory C-type lectin receptor signaling pathway | 23 out of 2891 genes, 0.8% | 100 out of 15382 genes, 0.7% | 1 |  |
| protein processing | 32 out of 2891 genes, 1.1% | 144 out of 15382 genes, 0.9% | 1 |  |
| positive regulation of regulated secretory pathway | 11 out of 2891 genes, 0.4% | 43 out of 15382 genes, 0.3% | 1 |  |
| regulation of extrinsic apoptotic signaling pathway in absence of ligand | 11 out of 2891 genes, 0.4% | 43 out of 15382 genes, 0.3% | 1 |  |
| nuclear-transcribed mRNA catabolic process | 35 out of 2891 genes, 1.2% | 159 out of 15382 genes, 1.0% | 1 |  |
| positive regulation of signaling | 285 out of 2891 genes, 9.9% | 1443 out of 15382 genes, 9.4% | 1 |  |
| regulation of establishment of protein localization to mitochondrion | 25 out of 2891 genes, 0.9% | 110 out of 15382 genes, 0.7% | 1 |  |
| DNA modification | 19 out of 2891 genes, 0.7% | 81 out of 15382 genes, 0.5% | 1 |  |
| calcium ion transport | 39 out of 2891 genes, 1.3% | 179 out of 15382 genes, 1.2% | 1 |  |
| transmembrane receptor protein serine/threonine kinase signaling pathway | 39 out of 2891 genes, 1.3% | 179 out of 15382 genes, 1.2% | 1 |  |
| endodermal cell fate commitment | 4 out of 2891 genes, 0.1% | 12 out of 15382 genes, 0.1% | 1 |  |
| galactose metabolic process | 4 out of 2891 genes, 0.1% | 12 out of 15382 genes, 0.1% | 1 |  |
| ethanol oxidation | 4 out of 2891 genes, 0.1% | 12 out of 15382 genes, 0.1% | 1 |  |
| nitric oxide biosynthetic process | 4 out of 2891 genes, 0.1% | 12 out of 15382 genes, 0.1% | 1 |  |
| DNA damage response, signal transduction by p53 class mediator resulting in transcription of p21 class mediator | 4 out of 2891 genes, 0.1% | 12 out of 15382 genes, 0.1% | 1 |  |
| asymmetric cell division | 4 out of 2891 genes, 0.1% | 12 out of 15382 genes, 0.1% | 1 |  |
| response to water | 4 out of 2891 genes, 0.1% | 12 out of 15382 genes, 0.1% | 1 |  |
| response to light intensity | 4 out of 2891 genes, 0.1% | 12 out of 15382 genes, 0.1% | 1 |  |
| negative regulation of sodium ion transport | 4 out of 2891 genes, 0.1% | 12 out of 15382 genes, 0.1% | 1 |  |
| negative regulation of keratinocyte proliferation | 4 out of 2891 genes, 0.1% | 12 out of 15382 genes, 0.1% | 1 |  |
| monocyte differentiation | 4 out of 2891 genes, 0.1% | 12 out of 15382 genes, 0.1% | 1 |  |
| response to laminar fluid shear stress | 4 out of 2891 genes, 0.1% | 12 out of 15382 genes, 0.1% | 1 |  |
| negative regulation of circadian rhythm | 4 out of 2891 genes, 0.1% | 12 out of 15382 genes, 0.1% | 1 |  |
| pigment accumulation | 4 out of 2891 genes, 0.1% | 12 out of 15382 genes, 0.1% | 1 |  |
| cellular pigment accumulation | 4 out of 2891 genes, 0.1% | 12 out of 15382 genes, 0.1% | 1 |  |
| protein neddylation | 4 out of 2891 genes, 0.1% | 12 out of 15382 genes, 0.1% | 1 |  |
| positive regulation of keratinocyte differentiation | 4 out of 2891 genes, 0.1% | 12 out of 15382 genes, 0.1% | 1 |  |
| positive regulation of cardiac muscle contraction | 4 out of 2891 genes, 0.1% | 12 out of 15382 genes, 0.1% | 1 |  |
| cellular response to fluid shear stress | 4 out of 2891 genes, 0.1% | 12 out of 15382 genes, 0.1% | 1 |  |
| ventricular cardiac muscle cell action potential | 4 out of 2891 genes, 0.1% | 12 out of 15382 genes, 0.1% | 1 |  |
| calcineurin-mediated signaling | 4 out of 2891 genes, 0.1% | 12 out of 15382 genes, 0.1% | 1 |  |
| regulation of postsynaptic membrane neurotransmitter receptor levels | 4 out of 2891 genes, 0.1% | 12 out of 15382 genes, 0.1% | 1 |  |
| positive regulation of long-term synaptic potentiation | 4 out of 2891 genes, 0.1% | 12 out of 15382 genes, 0.1% | 1 |  |
| fatty acid transmembrane transport | 4 out of 2891 genes, 0.1% | 12 out of 15382 genes, 0.1% | 1 |  |
| negative regulation of intrinsic apoptotic signaling pathway in response to DNA damage by p53 class mediator | 4 out of 2891 genes, 0.1% | 12 out of 15382 genes, 0.1% | 1 |  |
| mononuclear cell differentiation | 4 out of 2891 genes, 0.1% | 12 out of 15382 genes, 0.1% | 1 |  |
| regulation of bile acid metabolic process | 4 out of 2891 genes, 0.1% | 12 out of 15382 genes, 0.1% | 1 |  |
| negative regulation of ERAD pathway | 4 out of 2891 genes, 0.1% | 12 out of 15382 genes, 0.1% | 1 |  |
| regulation of early endosome to late endosome transport | 4 out of 2891 genes, 0.1% | 12 out of 15382 genes, 0.1% | 1 |  |
| regulation of double-strand break repair via nonhomologous end joining | 4 out of 2891 genes, 0.1% | 12 out of 15382 genes, 0.1% | 1 |  |
| regulation of cell cycle G2/M phase transition | 40 out of 2891 genes, 1.4% | 184 out of 15382 genes, 1.2% | 1 |  |
| positive regulation of leukocyte mediated cytotoxicity | 9 out of 2891 genes, 0.3% | 34 out of 15382 genes, 0.2% | 1 |  |
| regulation of CD4-positive, alpha-beta T cell differentiation | 9 out of 2891 genes, 0.3% | 34 out of 15382 genes, 0.2% | 1 |  |
| regulation of lymphocyte proliferation | 41 out of 2891 genes, 1.4% | 189 out of 15382 genes, 1.2% | 1 |  |
| regulation of I-kappaB kinase/NF-kappaB signaling | 44 out of 2891 genes, 1.5% | 204 out of 15382 genes, 1.3% | 1 |  |
| substrate-dependent cell migration | 7 out of 2891 genes, 0.2% | 25 out of 15382 genes, 0.2% | 1 |  |
| regulation of mesenchymal cell proliferation | 7 out of 2891 genes, 0.2% | 25 out of 15382 genes, 0.2% | 1 |  |
| stem cell division | 7 out of 2891 genes, 0.2% | 25 out of 15382 genes, 0.2% | 1 |  |
| negative regulation of proteasomal ubiquitin-dependent protein catabolic process | 7 out of 2891 genes, 0.2% | 25 out of 15382 genes, 0.2% | 1 |  |
| regulation of DNA damage response, signal transduction by p53 class mediator | 7 out of 2891 genes, 0.2% | 25 out of 15382 genes, 0.2% | 1 |  |
| erythrocyte development | 7 out of 2891 genes, 0.2% | 25 out of 15382 genes, 0.2% | 1 |  |
| regulation of amino acid transport | 7 out of 2891 genes, 0.2% | 25 out of 15382 genes, 0.2% | 1 |  |
| dendritic spine development | 7 out of 2891 genes, 0.2% | 25 out of 15382 genes, 0.2% | 1 |  |
| autophagosome maturation | 7 out of 2891 genes, 0.2% | 25 out of 15382 genes, 0.2% | 1 |  |
| neutral lipid metabolic process | 20 out of 2891 genes, 0.7% | 86 out of 15382 genes, 0.6% | 1 |  |
| acylglycerol metabolic process | 20 out of 2891 genes, 0.7% | 86 out of 15382 genes, 0.6% | 1 |  |
| ovulation from ovarian follicle | 3 out of 2891 genes, 0.1% | 8 out of 15382 genes, 0.1% | 1 |  |
| UDP-N-acetylglucosamine biosynthetic process | 3 out of 2891 genes, 0.1% | 8 out of 15382 genes, 0.1% | 1 |  |
| N-acetylneuraminate metabolic process | 3 out of 2891 genes, 0.1% | 8 out of 15382 genes, 0.1% | 1 |  |
| DNA topological change | 3 out of 2891 genes, 0.1% | 8 out of 15382 genes, 0.1% | 1 |  |
| transcription initiation from RNA polymerase III promoter | 3 out of 2891 genes, 0.1% | 8 out of 15382 genes, 0.1% | 1 |  |
| activation of signaling protein activity involved in unfolded protein response | 3 out of 2891 genes, 0.1% | 8 out of 15382 genes, 0.1% | 1 |  |
| cytoskeletal anchoring at plasma membrane | 3 out of 2891 genes, 0.1% | 8 out of 15382 genes, 0.1% | 1 |  |
| deoxyribonucleoside monophosphate metabolic process | 3 out of 2891 genes, 0.1% | 8 out of 15382 genes, 0.1% | 1 |  |
| regulation of mitochondrial fusion | 3 out of 2891 genes, 0.1% | 8 out of 15382 genes, 0.1% | 1 |  |
| negative regulation of phosphatidylinositol 3-kinase signaling | 3 out of 2891 genes, 0.1% | 8 out of 15382 genes, 0.1% | 1 |  |
| negative regulation of muscle adaptation | 3 out of 2891 genes, 0.1% | 8 out of 15382 genes, 0.1% | 1 |  |
| striated muscle atrophy | 3 out of 2891 genes, 0.1% | 8 out of 15382 genes, 0.1% | 1 |  |
| polyol transport | 3 out of 2891 genes, 0.1% | 8 out of 15382 genes, 0.1% | 1 |  |
| purine nucleotide transport | 3 out of 2891 genes, 0.1% | 8 out of 15382 genes, 0.1% | 1 |  |
| synaptic vesicle docking | 3 out of 2891 genes, 0.1% | 8 out of 15382 genes, 0.1% | 1 |  |
| nucleosome positioning | 3 out of 2891 genes, 0.1% | 8 out of 15382 genes, 0.1% | 1 |  |
| GDP-mannose metabolic process | 3 out of 2891 genes, 0.1% | 8 out of 15382 genes, 0.1% | 1 |  |
| axonal transport of mitochondrion | 3 out of 2891 genes, 0.1% | 8 out of 15382 genes, 0.1% | 1 |  |
| midbrain-hindbrain boundary development | 3 out of 2891 genes, 0.1% | 8 out of 15382 genes, 0.1% | 1 |  |
| positive regulation of synaptic plasticity | 3 out of 2891 genes, 0.1% | 8 out of 15382 genes, 0.1% | 1 |  |
| calcineurin-NFAT signaling cascade | 3 out of 2891 genes, 0.1% | 8 out of 15382 genes, 0.1% | 1 |  |
| regulation of maintenance of sister chromatid cohesion | 3 out of 2891 genes, 0.1% | 8 out of 15382 genes, 0.1% | 1 |  |
| regulation of maintenance of mitotic sister chromatid cohesion | 3 out of 2891 genes, 0.1% | 8 out of 15382 genes, 0.1% | 1 |  |
| negative regulation of transcription elongation from RNA polymerase II promoter | 3 out of 2891 genes, 0.1% | 8 out of 15382 genes, 0.1% | 1 |  |
| regulation of glial cell apoptotic process | 3 out of 2891 genes, 0.1% | 8 out of 15382 genes, 0.1% | 1 |  |
| negative regulation of glial cell apoptotic process | 3 out of 2891 genes, 0.1% | 8 out of 15382 genes, 0.1% | 1 |  |
| negative regulation of hippo signaling | 3 out of 2891 genes, 0.1% | 8 out of 15382 genes, 0.1% | 1 |  |
| protein localization to microtubule | 3 out of 2891 genes, 0.1% | 8 out of 15382 genes, 0.1% | 1 |  |
| negative regulation of chromatin binding | 3 out of 2891 genes, 0.1% | 8 out of 15382 genes, 0.1% | 1 |  |
| regulation of MHC class II biosynthetic process | 3 out of 2891 genes, 0.1% | 8 out of 15382 genes, 0.1% | 1 |  |
| positive regulation of action potential | 3 out of 2891 genes, 0.1% | 8 out of 15382 genes, 0.1% | 1 |  |
| regulation of female receptivity | 3 out of 2891 genes, 0.1% | 8 out of 15382 genes, 0.1% | 1 |  |
| carbohydrate phosphorylation | 3 out of 2891 genes, 0.1% | 8 out of 15382 genes, 0.1% | 1 |  |
| paraxial mesoderm morphogenesis | 3 out of 2891 genes, 0.1% | 8 out of 15382 genes, 0.1% | 1 |  |
| regulation of pinocytosis | 3 out of 2891 genes, 0.1% | 8 out of 15382 genes, 0.1% | 1 |  |
| histone H3-K9 methylation | 3 out of 2891 genes, 0.1% | 8 out of 15382 genes, 0.1% | 1 |  |
| negative regulation of ryanodine-sensitive calcium-release channel activity | 3 out of 2891 genes, 0.1% | 8 out of 15382 genes, 0.1% | 1 |  |
| chorion development | 3 out of 2891 genes, 0.1% | 8 out of 15382 genes, 0.1% | 1 |  |
| cellular response to histamine | 3 out of 2891 genes, 0.1% | 8 out of 15382 genes, 0.1% | 1 |  |
| cellular response to epinephrine stimulus | 3 out of 2891 genes, 0.1% | 8 out of 15382 genes, 0.1% | 1 |  |
| positive regulation of synapse maturation | 3 out of 2891 genes, 0.1% | 8 out of 15382 genes, 0.1% | 1 |  |
| protein localization to nuclear envelope | 3 out of 2891 genes, 0.1% | 8 out of 15382 genes, 0.1% | 1 |  |
| self proteolysis | 3 out of 2891 genes, 0.1% | 8 out of 15382 genes, 0.1% | 1 |  |
| observational learning | 3 out of 2891 genes, 0.1% | 8 out of 15382 genes, 0.1% | 1 |  |
| endosome to plasma membrane protein transport | 3 out of 2891 genes, 0.1% | 8 out of 15382 genes, 0.1% | 1 |  |
| negative regulation of endoplasmic reticulum unfolded protein response | 3 out of 2891 genes, 0.1% | 8 out of 15382 genes, 0.1% | 1 |  |
| regulation of amyloid-beta clearance | 3 out of 2891 genes, 0.1% | 8 out of 15382 genes, 0.1% | 1 |  |
| calcium ion export | 3 out of 2891 genes, 0.1% | 8 out of 15382 genes, 0.1% | 1 |  |
| regulation of Golgi organization | 3 out of 2891 genes, 0.1% | 8 out of 15382 genes, 0.1% | 1 |  |
| positive regulation of cytoplasmic transport | 3 out of 2891 genes, 0.1% | 8 out of 15382 genes, 0.1% | 1 |  |
| regulation of PERK-mediated unfolded protein response | 3 out of 2891 genes, 0.1% | 8 out of 15382 genes, 0.1% | 1 |  |
| amyloid fibril formation | 3 out of 2891 genes, 0.1% | 8 out of 15382 genes, 0.1% | 1 |  |
| embryonic brain development | 3 out of 2891 genes, 0.1% | 8 out of 15382 genes, 0.1% | 1 |  |
| cellular response to brain-derived neurotrophic factor stimulus | 3 out of 2891 genes, 0.1% | 8 out of 15382 genes, 0.1% | 1 |  |
| positive regulation of transcription from RNA polymerase II promoter in response to endoplasmic reticulum stress | 3 out of 2891 genes, 0.1% | 8 out of 15382 genes, 0.1% | 1 |  |
| negative regulation of double-strand break repair via homologous recombination | 3 out of 2891 genes, 0.1% | 8 out of 15382 genes, 0.1% | 1 |  |
| regulation of endocytic recycling | 3 out of 2891 genes, 0.1% | 8 out of 15382 genes, 0.1% | 1 |  |
| negative regulation of ERK1 and ERK2 cascade | 12 out of 2891 genes, 0.4% | 48 out of 15382 genes, 0.3% | 1 |  |
| protein transport along microtubule | 12 out of 2891 genes, 0.4% | 48 out of 15382 genes, 0.3% | 1 |  |
| microtubule-based protein transport | 12 out of 2891 genes, 0.4% | 48 out of 15382 genes, 0.3% | 1 |  |
| internal protein amino acid acetylation | 21 out of 2891 genes, 0.7% | 91 out of 15382 genes, 0.6% | 1 |  |
| nucleobase-containing compound catabolic process | 65 out of 2891 genes, 2.2% | 310 out of 15382 genes, 2.0% | 1 |  |
| negative regulation of ion transmembrane transport | 16 out of 2891 genes, 0.6% | 67 out of 15382 genes, 0.4% | 1 |  |
| protein homotetramerization | 16 out of 2891 genes, 0.6% | 67 out of 15382 genes, 0.4% | 1 |  |
| cytosolic calcium ion transport | 16 out of 2891 genes, 0.6% | 67 out of 15382 genes, 0.4% | 1 |  |
| regulation of epithelial cell apoptotic process | 16 out of 2891 genes, 0.6% | 67 out of 15382 genes, 0.4% | 1 |  |
| cell cycle arrest | 30 out of 2891 genes, 1.0% | 135 out of 15382 genes, 0.9% | 1 |  |
| amino acid transport | 22 out of 2891 genes, 0.8% | 96 out of 15382 genes, 0.6% | 1 |  |
| response to leukemia inhibitory factor | 22 out of 2891 genes, 0.8% | 96 out of 15382 genes, 0.6% | 1 |  |
| cellular response to leukemia inhibitory factor | 22 out of 2891 genes, 0.8% | 96 out of 15382 genes, 0.6% | 1 |  |
| regulation of MAP kinase activity | 60 out of 2891 genes, 2.1% | 285 out of 15382 genes, 1.9% | 1 |  |
| fat cell differentiation | 23 out of 2891 genes, 0.8% | 101 out of 15382 genes, 0.7% | 1 |  |
| positive regulation of intracellular protein transport | 36 out of 2891 genes, 1.2% | 165 out of 15382 genes, 1.1% | 1 |  |
| maintenance of protein location in cell | 13 out of 2891 genes, 0.4% | 53 out of 15382 genes, 0.3% | 1 |  |
| regulation of cardiac muscle tissue development | 13 out of 2891 genes, 0.4% | 53 out of 15382 genes, 0.3% | 1 |  |
| negative regulation of proteolysis involved in cellular protein catabolic process | 13 out of 2891 genes, 0.4% | 53 out of 15382 genes, 0.3% | 1 |  |
| cell motility | 171 out of 2891 genes, 5.9% | 854 out of 15382 genes, 5.6% | 1 |  |
| localization of cell | 171 out of 2891 genes, 5.9% | 854 out of 15382 genes, 5.6% | 1 |  |
| regulation of G2/M transition of mitotic cell cycle | 38 out of 2891 genes, 1.3% | 175 out of 15382 genes, 1.1% | 1 |  |
| cellular response to amino acid starvation | 10 out of 2891 genes, 0.3% | 39 out of 15382 genes, 0.3% | 1 |  |
| regulation of cardiac muscle tissue growth | 10 out of 2891 genes, 0.3% | 39 out of 15382 genes, 0.3% | 1 |  |
| positive regulation of response to cytokine stimulus | 10 out of 2891 genes, 0.3% | 39 out of 15382 genes, 0.3% | 1 |  |
| monosaccharide metabolic process | 40 out of 2891 genes, 1.4% | 185 out of 15382 genes, 1.2% | 1 |  |
| cell surface receptor signaling pathway involved in cell-cell signaling | 70 out of 2891 genes, 2.4% | 336 out of 15382 genes, 2.2% | 1 |  |
| innate immune response-activating signal transduction | 41 out of 2891 genes, 1.4% | 190 out of 15382 genes, 1.2% | 1 |  |
| regulation of mononuclear cell proliferation | 41 out of 2891 genes, 1.4% | 190 out of 15382 genes, 1.2% | 1 |  |
| developmental maturation | 43 out of 2891 genes, 1.5% | 200 out of 15382 genes, 1.3% | 1 |  |
| mesoderm development | 24 out of 2891 genes, 0.8% | 106 out of 15382 genes, 0.7% | 1 |  |
| post-translational protein modification | 81 out of 2891 genes, 2.8% | 392 out of 15382 genes, 2.5% | 1 |  |
| negative regulation of angiogenesis | 18 out of 2891 genes, 0.6% | 77 out of 15382 genes, 0.5% | 1 |  |
| regulation of fatty acid metabolic process | 18 out of 2891 genes, 0.6% | 77 out of 15382 genes, 0.5% | 1 |  |
| embryonic organ development | 79 out of 2891 genes, 2.7% | 382 out of 15382 genes, 2.5% | 1 |  |
| regulation of receptor recycling | 6 out of 2891 genes, 0.2% | 21 out of 15382 genes, 0.1% | 1 |  |
| regulation of sister chromatid cohesion | 6 out of 2891 genes, 0.2% | 21 out of 15382 genes, 0.1% | 1 |  |
| positive regulation of leukocyte degranulation | 6 out of 2891 genes, 0.2% | 21 out of 15382 genes, 0.1% | 1 |  |
| positive regulation of isotype switching | 6 out of 2891 genes, 0.2% | 21 out of 15382 genes, 0.1% | 1 |  |
| regulation of axon regeneration | 6 out of 2891 genes, 0.2% | 21 out of 15382 genes, 0.1% | 1 |  |
| regulation of ryanodine-sensitive calcium-release channel activity | 6 out of 2891 genes, 0.2% | 21 out of 15382 genes, 0.1% | 1 |  |
| myofibril assembly | 14 out of 2891 genes, 0.5% | 58 out of 15382 genes, 0.4% | 1 |  |
| negative regulation of cation transmembrane transport | 14 out of 2891 genes, 0.5% | 58 out of 15382 genes, 0.4% | 1 |  |
| response to ischemia | 8 out of 2891 genes, 0.3% | 30 out of 15382 genes, 0.2% | 1 |  |
| long-term memory | 8 out of 2891 genes, 0.3% | 30 out of 15382 genes, 0.2% | 1 |  |
| G2 DNA damage checkpoint | 8 out of 2891 genes, 0.3% | 30 out of 15382 genes, 0.2% | 1 |  |
| negative regulation of telomere maintenance | 8 out of 2891 genes, 0.3% | 30 out of 15382 genes, 0.2% | 1 |  |
| regulation of natural killer cell activation | 8 out of 2891 genes, 0.3% | 30 out of 15382 genes, 0.2% | 1 |  |
| regulation of phosphatidylinositol 3-kinase activity | 8 out of 2891 genes, 0.3% | 30 out of 15382 genes, 0.2% | 1 |  |
| modulation of excitatory postsynaptic potential | 8 out of 2891 genes, 0.3% | 30 out of 15382 genes, 0.2% | 1 |  |
| positive regulation of epithelial cell apoptotic process | 8 out of 2891 genes, 0.3% | 30 out of 15382 genes, 0.2% | 1 |  |
| positive regulation of telomere maintenance via telomere lengthening | 8 out of 2891 genes, 0.3% | 30 out of 15382 genes, 0.2% | 1 |  |
| negative regulation of lymphocyte activation | 27 out of 2891 genes, 0.9% | 121 out of 15382 genes, 0.8% | 1 |  |
| regulation of striated muscle cell differentiation | 19 out of 2891 genes, 0.7% | 82 out of 15382 genes, 0.5% | 1 |  |
| cell migration | 152 out of 2891 genes, 5.3% | 757 out of 15382 genes, 4.9% | 1 |  |
| membrane invagination | 11 out of 2891 genes, 0.4% | 44 out of 15382 genes, 0.3% | 1 |  |
| regulation of heart growth | 11 out of 2891 genes, 0.4% | 44 out of 15382 genes, 0.3% | 1 |  |
| regulation of potassium ion transmembrane transporter activity | 11 out of 2891 genes, 0.4% | 44 out of 15382 genes, 0.3% | 1 |  |
| protein stabilization | 31 out of 2891 genes, 1.1% | 141 out of 15382 genes, 0.9% | 1 |  |
| rhythmic process | 53 out of 2891 genes, 1.8% | 251 out of 15382 genes, 1.6% | 1 |  |
| energy reserve metabolic process | 15 out of 2891 genes, 0.5% | 63 out of 15382 genes, 0.4% | 1 |  |
| cardiac muscle contraction | 15 out of 2891 genes, 0.5% | 63 out of 15382 genes, 0.4% | 1 |  |
| cerebellum development | 21 out of 2891 genes, 0.7% | 92 out of 15382 genes, 0.6% | 1 |  |
| cellular response to biotic stimulus | 35 out of 2891 genes, 1.2% | 161 out of 15382 genes, 1.0% | 1 |  |
| sister chromatid segregation | 39 out of 2891 genes, 1.3% | 181 out of 15382 genes, 1.2% | 1 |  |
| regulation of transmembrane receptor protein serine/threonine kinase signaling pathway | 40 out of 2891 genes, 1.4% | 186 out of 15382 genes, 1.2% | 1 |  |
| regulation of epidermis development | 16 out of 2891 genes, 0.6% | 68 out of 15382 genes, 0.4% | 1 |  |
| response to anesthetic | 16 out of 2891 genes, 0.6% | 68 out of 15382 genes, 0.4% | 1 |  |
| leukocyte mediated immunity | 116 out of 2891 genes, 4.0% | 573 out of 15382 genes, 3.7% | 1 |  |
| negative regulation of RNA catabolic process | 12 out of 2891 genes, 0.4% | 49 out of 15382 genes, 0.3% | 1 |  |
| positive regulation of synaptic transmission | 24 out of 2891 genes, 0.8% | 107 out of 15382 genes, 0.7% | 1 |  |
| regulation of growth | 114 out of 2891 genes, 3.9% | 563 out of 15382 genes, 3.7% | 1 |  |
| positive regulation of cytokine-mediated signaling pathway | 9 out of 2891 genes, 0.3% | 35 out of 15382 genes, 0.2% | 1 |  |
| DNA replication-independent nucleosome assembly | 9 out of 2891 genes, 0.3% | 35 out of 15382 genes, 0.2% | 1 |  |
| negative regulation of actin filament depolymerization | 9 out of 2891 genes, 0.3% | 35 out of 15382 genes, 0.2% | 1 |  |
| lysosome localization | 9 out of 2891 genes, 0.3% | 35 out of 15382 genes, 0.2% | 1 |  |
| regulation of protein oligomerization | 9 out of 2891 genes, 0.3% | 35 out of 15382 genes, 0.2% | 1 |  |
| hair cell differentiation | 9 out of 2891 genes, 0.3% | 35 out of 15382 genes, 0.2% | 1 |  |
| axon extension | 9 out of 2891 genes, 0.3% | 35 out of 15382 genes, 0.2% | 1 |  |
| lymphocyte activation | 70 out of 2891 genes, 2.4% | 338 out of 15382 genes, 2.2% | 1 |  |
| regulation of transcription factor import into nucleus | 17 out of 2891 genes, 0.6% | 73 out of 15382 genes, 0.5% | 1 |  |
| cellular response to ketone | 17 out of 2891 genes, 0.6% | 73 out of 15382 genes, 0.5% | 1 |  |
| cellular lipid metabolic process | 172 out of 2891 genes, 5.9% | 863 out of 15382 genes, 5.6% | 1 |  |
| pyrimidine nucleobase metabolic process | 5 out of 2891 genes, 0.2% | 17 out of 15382 genes, 0.1% | 1 |  |
| cyclic nucleotide catabolic process | 5 out of 2891 genes, 0.2% | 17 out of 15382 genes, 0.1% | 1 |  |
| regulation of triglyceride biosynthetic process | 5 out of 2891 genes, 0.2% | 17 out of 15382 genes, 0.1% | 1 |  |
| regulation of receptor biosynthetic process | 5 out of 2891 genes, 0.2% | 17 out of 15382 genes, 0.1% | 1 |  |
| maintenance of cell polarity | 5 out of 2891 genes, 0.2% | 17 out of 15382 genes, 0.1% | 1 |  |
| nuclear envelope reassembly | 5 out of 2891 genes, 0.2% | 17 out of 15382 genes, 0.1% | 1 |  |
| regulation of mammary gland epithelial cell proliferation | 5 out of 2891 genes, 0.2% | 17 out of 15382 genes, 0.1% | 1 |  |
| diol metabolic process | 5 out of 2891 genes, 0.2% | 17 out of 15382 genes, 0.1% | 1 |  |
| lipid particle organization | 5 out of 2891 genes, 0.2% | 17 out of 15382 genes, 0.1% | 1 |  |
| positive regulation of T-helper cell differentiation | 5 out of 2891 genes, 0.2% | 17 out of 15382 genes, 0.1% | 1 |  |
| regulation of membrane protein ectodomain proteolysis | 5 out of 2891 genes, 0.2% | 17 out of 15382 genes, 0.1% | 1 |  |
| protein heterotetramerization | 5 out of 2891 genes, 0.2% | 17 out of 15382 genes, 0.1% | 1 |  |
| pericardium development | 5 out of 2891 genes, 0.2% | 17 out of 15382 genes, 0.1% | 1 |  |
| transepithelial transport | 5 out of 2891 genes, 0.2% | 17 out of 15382 genes, 0.1% | 1 |  |
| DNA-templated transcriptional preinitiation complex assembly | 5 out of 2891 genes, 0.2% | 17 out of 15382 genes, 0.1% | 1 |  |
| response to nitric oxide | 5 out of 2891 genes, 0.2% | 17 out of 15382 genes, 0.1% | 1 |  |
| positive regulation of triglyceride metabolic process | 5 out of 2891 genes, 0.2% | 17 out of 15382 genes, 0.1% | 1 |  |
| response to mitochondrial depolarisation | 5 out of 2891 genes, 0.2% | 17 out of 15382 genes, 0.1% | 1 |  |
| regulation of cardiac muscle cell membrane repolarization | 5 out of 2891 genes, 0.2% | 17 out of 15382 genes, 0.1% | 1 |  |
| negative regulation of response to reactive oxygen species | 5 out of 2891 genes, 0.2% | 17 out of 15382 genes, 0.1% | 1 |  |
| negative regulation of glycoprotein metabolic process | 5 out of 2891 genes, 0.2% | 17 out of 15382 genes, 0.1% | 1 |  |
| negative regulation of hydrogen peroxide-induced cell death | 5 out of 2891 genes, 0.2% | 17 out of 15382 genes, 0.1% | 1 |  |
| regulation of dendrite extension | 5 out of 2891 genes, 0.2% | 17 out of 15382 genes, 0.1% | 1 |  |
| positive regulation of gene silencing by miRNA | 5 out of 2891 genes, 0.2% | 17 out of 15382 genes, 0.1% | 1 |  |
| negative regulation of cellular response to drug | 5 out of 2891 genes, 0.2% | 17 out of 15382 genes, 0.1% | 1 |  |
| negative regulation of DNA binding transcription factor activity | 29 out of 2891 genes, 1.0% | 132 out of 15382 genes, 0.9% | 1 |  |
| mitotic nuclear division | 29 out of 2891 genes, 1.0% | 132 out of 15382 genes, 0.9% | 1 |  |
| IRE1-mediated unfolded protein response | 13 out of 2891 genes, 0.4% | 54 out of 15382 genes, 0.4% | 1 |  |
| regulation of oxidative stress-induced cell death | 13 out of 2891 genes, 0.4% | 54 out of 15382 genes, 0.4% | 1 |  |
| behavioral fear response | 7 out of 2891 genes, 0.2% | 26 out of 15382 genes, 0.2% | 1 |  |
| behavioral defense response | 7 out of 2891 genes, 0.2% | 26 out of 15382 genes, 0.2% | 1 |  |
| mismatch repair | 7 out of 2891 genes, 0.2% | 26 out of 15382 genes, 0.2% | 1 |  |
| bile acid and bile salt transport | 7 out of 2891 genes, 0.2% | 26 out of 15382 genes, 0.2% | 1 |  |
| central nervous system neuron axonogenesis | 7 out of 2891 genes, 0.2% | 26 out of 15382 genes, 0.2% | 1 |  |
| positive regulation of tissue remodeling | 7 out of 2891 genes, 0.2% | 26 out of 15382 genes, 0.2% | 1 |  |
| cellular component maintenance | 7 out of 2891 genes, 0.2% | 26 out of 15382 genes, 0.2% | 1 |  |
| negative regulation of striated muscle cell differentiation | 7 out of 2891 genes, 0.2% | 26 out of 15382 genes, 0.2% | 1 |  |
| positive regulation of heart growth | 7 out of 2891 genes, 0.2% | 26 out of 15382 genes, 0.2% | 1 |  |
| cell differentiation involved in embryonic placenta development | 7 out of 2891 genes, 0.2% | 26 out of 15382 genes, 0.2% | 1 |  |
| positive regulation of protein acetylation | 7 out of 2891 genes, 0.2% | 26 out of 15382 genes, 0.2% | 1 |  |
| negative regulation of organelle assembly | 7 out of 2891 genes, 0.2% | 26 out of 15382 genes, 0.2% | 1 |  |
| lymphocyte differentiation | 44 out of 2891 genes, 1.5% | 207 out of 15382 genes, 1.3% | 1 |  |
| response to nutrient levels | 83 out of 2891 genes, 2.9% | 405 out of 15382 genes, 2.6% | 1 |  |
| positive regulation of protein ubiquitination | 35 out of 2891 genes, 1.2% | 162 out of 15382 genes, 1.1% | 1 |  |
| mRNA catabolic process | 37 out of 2891 genes, 1.3% | 172 out of 15382 genes, 1.1% | 1 |  |
| positive regulation of cell communication | 282 out of 2891 genes, 9.8% | 1436 out of 15382 genes, 9.3% | 1 |  |
| leukocyte cell-cell adhesion | 10 out of 2891 genes, 0.3% | 40 out of 15382 genes, 0.3% | 1 |  |
| negative regulation of I-kappaB kinase/NF-kappaB signaling | 10 out of 2891 genes, 0.3% | 40 out of 15382 genes, 0.3% | 1 |  |
| inner ear receptor cell development | 10 out of 2891 genes, 0.3% | 40 out of 15382 genes, 0.3% | 1 |  |
| response to dexamethasone | 10 out of 2891 genes, 0.3% | 40 out of 15382 genes, 0.3% | 1 |  |
| cardiac muscle cell action potential | 10 out of 2891 genes, 0.3% | 40 out of 15382 genes, 0.3% | 1 |  |
| DNA methylation or demethylation | 14 out of 2891 genes, 0.5% | 59 out of 15382 genes, 0.4% | 1 |  |
| cardiac septum morphogenesis | 14 out of 2891 genes, 0.5% | 59 out of 15382 genes, 0.4% | 1 |  |
| lipid biosynthetic process | 103 out of 2891 genes, 3.6% | 508 out of 15382 genes, 3.3% | 1 |  |
| determination of left/right symmetry | 22 out of 2891 genes, 0.8% | 98 out of 15382 genes, 0.6% | 1 |  |
| regulation of epithelial to mesenchymal transition | 15 out of 2891 genes, 0.5% | 64 out of 15382 genes, 0.4% | 1 |  |
| innate immune response activating cell surface receptor signaling pathway | 23 out of 2891 genes, 0.8% | 103 out of 15382 genes, 0.7% | 1 |  |
| non-canonical Wnt signaling pathway | 24 out of 2891 genes, 0.8% | 108 out of 15382 genes, 0.7% | 1 |  |
| regulation of alternative mRNA splicing, via spliceosome | 11 out of 2891 genes, 0.4% | 45 out of 15382 genes, 0.3% | 1 |  |
| mammary gland epithelium development | 11 out of 2891 genes, 0.4% | 45 out of 15382 genes, 0.3% | 1 |  |
| regulation of DNA-dependent DNA replication | 11 out of 2891 genes, 0.4% | 45 out of 15382 genes, 0.3% | 1 |  |
| aerobic respiration | 16 out of 2891 genes, 0.6% | 69 out of 15382 genes, 0.4% | 1 |  |
| positive regulation of response to DNA damage stimulus | 16 out of 2891 genes, 0.6% | 69 out of 15382 genes, 0.4% | 1 |  |
| intracellular receptor signaling pathway | 27 out of 2891 genes, 0.9% | 123 out of 15382 genes, 0.8% | 1 |  |
| regulation of muscle cell differentiation | 27 out of 2891 genes, 0.9% | 123 out of 15382 genes, 0.8% | 1 |  |
| heart morphogenesis | 41 out of 2891 genes, 1.4% | 193 out of 15382 genes, 1.3% | 1 |  |
| establishment or maintenance of epithelial cell apical/basal polarity | 8 out of 2891 genes, 0.3% | 31 out of 15382 genes, 0.2% | 1 |  |
| negative regulation of T cell differentiation | 8 out of 2891 genes, 0.3% | 31 out of 15382 genes, 0.2% | 1 |  |
| phospholipid dephosphorylation | 8 out of 2891 genes, 0.3% | 31 out of 15382 genes, 0.2% | 1 |  |
| lung epithelium development | 8 out of 2891 genes, 0.3% | 31 out of 15382 genes, 0.2% | 1 |  |
| regulation of actin filament-based movement | 8 out of 2891 genes, 0.3% | 31 out of 15382 genes, 0.2% | 1 |  |
| negative regulation of DNA biosynthetic process | 8 out of 2891 genes, 0.3% | 31 out of 15382 genes, 0.2% | 1 |  |
| positive regulation of DNA metabolic process | 39 out of 2891 genes, 1.3% | 183 out of 15382 genes, 1.2% | 1 |  |
| regulation of intrinsic apoptotic signaling pathway | 30 out of 2891 genes, 1.0% | 138 out of 15382 genes, 0.9% | 1 |  |
| appendage development | 33 out of 2891 genes, 1.1% | 153 out of 15382 genes, 1.0% | 1 |  |
| limb development | 33 out of 2891 genes, 1.1% | 153 out of 15382 genes, 1.0% | 1 |  |
| production of molecular mediator involved in inflammatory response | 4 out of 2891 genes, 0.1% | 13 out of 15382 genes, 0.1% | 1 |  |
| negative regulation of calcium ion transport into cytosol | 4 out of 2891 genes, 0.1% | 13 out of 15382 genes, 0.1% | 1 |  |
| dorsal/ventral neural tube patterning | 4 out of 2891 genes, 0.1% | 13 out of 15382 genes, 0.1% | 1 |  |
| positive regulation of CREB transcription factor activity | 4 out of 2891 genes, 0.1% | 13 out of 15382 genes, 0.1% | 1 |  |
| regulation of natural killer cell differentiation | 4 out of 2891 genes, 0.1% | 13 out of 15382 genes, 0.1% | 1 |  |
| alpha-linolenic acid metabolic process | 4 out of 2891 genes, 0.1% | 13 out of 15382 genes, 0.1% | 1 |  |
| DNA damage response, signal transduction resulting in transcription | 4 out of 2891 genes, 0.1% | 13 out of 15382 genes, 0.1% | 1 |  |
| negative regulation of vascular permeability | 4 out of 2891 genes, 0.1% | 13 out of 15382 genes, 0.1% | 1 |  |
| anoikis | 4 out of 2891 genes, 0.1% | 13 out of 15382 genes, 0.1% | 1 |  |
| negative regulation of fatty acid biosynthetic process | 4 out of 2891 genes, 0.1% | 13 out of 15382 genes, 0.1% | 1 |  |
| negative regulation of glucose import | 4 out of 2891 genes, 0.1% | 13 out of 15382 genes, 0.1% | 1 |  |
| peripheral nervous system neuron differentiation | 4 out of 2891 genes, 0.1% | 13 out of 15382 genes, 0.1% | 1 |  |
| peripheral nervous system neuron development | 4 out of 2891 genes, 0.1% | 13 out of 15382 genes, 0.1% | 1 |  |
| leukocyte tethering or rolling | 4 out of 2891 genes, 0.1% | 13 out of 15382 genes, 0.1% | 1 |  |
| Sertoli cell development | 4 out of 2891 genes, 0.1% | 13 out of 15382 genes, 0.1% | 1 |  |
| negative regulation of glial cell proliferation | 4 out of 2891 genes, 0.1% | 13 out of 15382 genes, 0.1% | 1 |  |
| leukocyte adhesion to vascular endothelial cell | 4 out of 2891 genes, 0.1% | 13 out of 15382 genes, 0.1% | 1 |  |
| protein kinase C signaling | 4 out of 2891 genes, 0.1% | 13 out of 15382 genes, 0.1% | 1 |  |
| cellular response to cholesterol | 4 out of 2891 genes, 0.1% | 13 out of 15382 genes, 0.1% | 1 |  |
| hematopoietic stem cell proliferation | 4 out of 2891 genes, 0.1% | 13 out of 15382 genes, 0.1% | 1 |  |
| regulation of intrinsic apoptotic signaling pathway in response to DNA damage by p53 class mediator | 4 out of 2891 genes, 0.1% | 13 out of 15382 genes, 0.1% | 1 |  |
| regulation of exosomal secretion | 4 out of 2891 genes, 0.1% | 13 out of 15382 genes, 0.1% | 1 |  |
| positive regulation of exosomal secretion | 4 out of 2891 genes, 0.1% | 13 out of 15382 genes, 0.1% | 1 |  |
| positive regulation of sprouting angiogenesis | 4 out of 2891 genes, 0.1% | 13 out of 15382 genes, 0.1% | 1 |  |
| negative regulation of TORC1 signaling | 4 out of 2891 genes, 0.1% | 13 out of 15382 genes, 0.1% | 1 |  |
| positive regulation of endothelial cell apoptotic process | 4 out of 2891 genes, 0.1% | 13 out of 15382 genes, 0.1% | 1 |  |
| regulation of mesoderm development | 4 out of 2891 genes, 0.1% | 13 out of 15382 genes, 0.1% | 1 |  |
| negative regulation of stem cell differentiation | 4 out of 2891 genes, 0.1% | 13 out of 15382 genes, 0.1% | 1 |  |
| signal transduction by protein phosphorylation | 66 out of 2891 genes, 2.3% | 320 out of 15382 genes, 2.1% | 1 |  |
| cerebellar cortex development | 12 out of 2891 genes, 0.4% | 50 out of 15382 genes, 0.3% | 1 |  |
| receptor internalization | 12 out of 2891 genes, 0.4% | 50 out of 15382 genes, 0.3% | 1 |  |
| inner ear receptor cell differentiation | 12 out of 2891 genes, 0.4% | 50 out of 15382 genes, 0.3% | 1 |  |
| positive regulation of natural killer cell mediated immunity | 6 out of 2891 genes, 0.2% | 22 out of 15382 genes, 0.1% | 1 |  |
| atrial septum development | 6 out of 2891 genes, 0.2% | 22 out of 15382 genes, 0.1% | 1 |  |
| glutamine metabolic process | 6 out of 2891 genes, 0.2% | 22 out of 15382 genes, 0.1% | 1 |  |
| protein export from nucleus | 6 out of 2891 genes, 0.2% | 22 out of 15382 genes, 0.1% | 1 |  |
| cell-substrate adherens junction assembly | 6 out of 2891 genes, 0.2% | 22 out of 15382 genes, 0.1% | 1 |  |
| positive regulation of DNA-templated transcription, elongation | 6 out of 2891 genes, 0.2% | 22 out of 15382 genes, 0.1% | 1 |  |
| peptidyl-tyrosine dephosphorylation | 6 out of 2891 genes, 0.2% | 22 out of 15382 genes, 0.1% | 1 |  |
| DNA dealkylation | 6 out of 2891 genes, 0.2% | 22 out of 15382 genes, 0.1% | 1 |  |
| establishment of mitotic spindle localization | 6 out of 2891 genes, 0.2% | 22 out of 15382 genes, 0.1% | 1 |  |
| regulation of hair cycle | 6 out of 2891 genes, 0.2% | 22 out of 15382 genes, 0.1% | 1 |  |
| regulation of cellular respiration | 6 out of 2891 genes, 0.2% | 22 out of 15382 genes, 0.1% | 1 |  |
| regulation of regulatory T cell differentiation | 6 out of 2891 genes, 0.2% | 22 out of 15382 genes, 0.1% | 1 |  |
| focal adhesion assembly | 6 out of 2891 genes, 0.2% | 22 out of 15382 genes, 0.1% | 1 |  |
| negative regulation of antigen receptor-mediated signaling pathway | 6 out of 2891 genes, 0.2% | 22 out of 15382 genes, 0.1% | 1 |  |
| positive regulation of cardiac muscle tissue growth | 6 out of 2891 genes, 0.2% | 22 out of 15382 genes, 0.1% | 1 |  |
| cellular response to misfolded protein | 6 out of 2891 genes, 0.2% | 22 out of 15382 genes, 0.1% | 1 |  |
| cellular response to cadmium ion | 6 out of 2891 genes, 0.2% | 22 out of 15382 genes, 0.1% | 1 |  |
| cellular response to interleukin-6 | 6 out of 2891 genes, 0.2% | 22 out of 15382 genes, 0.1% | 1 |  |
| telomere maintenance | 20 out of 2891 genes, 0.7% | 89 out of 15382 genes, 0.6% | 1 |  |
| cellular component assembly involved in morphogenesis | 20 out of 2891 genes, 0.7% | 89 out of 15382 genes, 0.6% | 1 |  |
| response to drug | 160 out of 2891 genes, 5.5% | 805 out of 15382 genes, 5.2% | 1 |  |
| regulation of mitotic sister chromatid segregation | 13 out of 2891 genes, 0.4% | 55 out of 15382 genes, 0.4% | 1 |  |
| mechanoreceptor differentiation | 13 out of 2891 genes, 0.4% | 55 out of 15382 genes, 0.4% | 1 |  |
| autophagy of mitochondrion | 9 out of 2891 genes, 0.3% | 36 out of 15382 genes, 0.2% | 1 |  |
| response to progesterone | 9 out of 2891 genes, 0.3% | 36 out of 15382 genes, 0.2% | 1 |  |
| DNA replication-independent nucleosome organization | 9 out of 2891 genes, 0.3% | 36 out of 15382 genes, 0.2% | 1 |  |
| positive regulation of viral transcription | 9 out of 2891 genes, 0.3% | 36 out of 15382 genes, 0.2% | 1 |  |
| multicellular organismal movement | 9 out of 2891 genes, 0.3% | 36 out of 15382 genes, 0.2% | 1 |  |
| musculoskeletal movement | 9 out of 2891 genes, 0.3% | 36 out of 15382 genes, 0.2% | 1 |  |
| head morphogenesis | 9 out of 2891 genes, 0.3% | 36 out of 15382 genes, 0.2% | 1 |  |
| mitochondrion disassembly | 9 out of 2891 genes, 0.3% | 36 out of 15382 genes, 0.2% | 1 |  |
| metencephalon development | 22 out of 2891 genes, 0.8% | 99 out of 15382 genes, 0.6% | 1 |  |
| response to lipopolysaccharide | 57 out of 2891 genes, 2.0% | 275 out of 15382 genes, 1.8% | 1 |  |
| leukocyte homeostasis | 14 out of 2891 genes, 0.5% | 60 out of 15382 genes, 0.4% | 1 |  |
| Rho protein signal transduction | 14 out of 2891 genes, 0.5% | 60 out of 15382 genes, 0.4% | 1 |  |
| multicellular organismal water homeostasis | 14 out of 2891 genes, 0.5% | 60 out of 15382 genes, 0.4% | 1 |  |
| response to estradiol | 25 out of 2891 genes, 0.9% | 114 out of 15382 genes, 0.7% | 1 |  |
| response to ammonium ion | 25 out of 2891 genes, 0.9% | 114 out of 15382 genes, 0.7% | 1 |  |
| lipid metabolic process | 216 out of 2891 genes, 7.5% | 1097 out of 15382 genes, 7.1% | 1 |  |
| negative regulation of neuron differentiation | 37 out of 2891 genes, 1.3% | 174 out of 15382 genes, 1.1% | 1 |  |
| defense response to virus | 37 out of 2891 genes, 1.3% | 174 out of 15382 genes, 1.1% | 1 |  |
| synapse organization | 36 out of 2891 genes, 1.2% | 169 out of 15382 genes, 1.1% | 1 |  |
| regulation of protein targeting | 27 out of 2891 genes, 0.9% | 124 out of 15382 genes, 0.8% | 1 |  |
| bone development | 34 out of 2891 genes, 1.2% | 159 out of 15382 genes, 1.0% | 1 |  |
| water homeostasis | 15 out of 2891 genes, 0.5% | 65 out of 15382 genes, 0.4% | 1 |  |
| regulation of protein secretion | 79 out of 2891 genes, 2.7% | 388 out of 15382 genes, 2.5% | 1 |  |
| aorta development | 10 out of 2891 genes, 0.3% | 41 out of 15382 genes, 0.3% | 1 |  |
| positive regulation of receptor-mediated endocytosis | 10 out of 2891 genes, 0.3% | 41 out of 15382 genes, 0.3% | 1 |  |
| deadenylation-dependent decapping of nuclear-transcribed mRNA | 3 out of 2891 genes, 0.1% | 9 out of 15382 genes, 0.1% | 1 |  |
| fructose 6-phosphate metabolic process | 3 out of 2891 genes, 0.1% | 9 out of 15382 genes, 0.1% | 1 |  |
| DNA dealkylation involved in DNA repair | 3 out of 2891 genes, 0.1% | 9 out of 15382 genes, 0.1% | 1 |  |
| L-serine metabolic process | 3 out of 2891 genes, 0.1% | 9 out of 15382 genes, 0.1% | 1 |  |
| valine metabolic process | 3 out of 2891 genes, 0.1% | 9 out of 15382 genes, 0.1% | 1 |  |
| phosphatidylglycerol biosynthetic process | 3 out of 2891 genes, 0.1% | 9 out of 15382 genes, 0.1% | 1 |  |
| cytoplasm organization | 3 out of 2891 genes, 0.1% | 9 out of 15382 genes, 0.1% | 1 |  |
| adenylate cyclase-activating dopamine receptor signaling pathway | 3 out of 2891 genes, 0.1% | 9 out of 15382 genes, 0.1% | 1 |  |
| positive regulation of steroid biosynthetic process | 3 out of 2891 genes, 0.1% | 9 out of 15382 genes, 0.1% | 1 |  |
| positive regulation of glutamate secretion | 3 out of 2891 genes, 0.1% | 9 out of 15382 genes, 0.1% | 1 |  |
| DNA integration | 3 out of 2891 genes, 0.1% | 9 out of 15382 genes, 0.1% | 1 |  |
| peptidyl-lysine monomethylation | 3 out of 2891 genes, 0.1% | 9 out of 15382 genes, 0.1% | 1 |  |
| virion attachment to host cell | 3 out of 2891 genes, 0.1% | 9 out of 15382 genes, 0.1% | 1 |  |
| regulation of natural killer cell proliferation | 3 out of 2891 genes, 0.1% | 9 out of 15382 genes, 0.1% | 1 |  |
| regulation of transforming growth factor beta1 production | 3 out of 2891 genes, 0.1% | 9 out of 15382 genes, 0.1% | 1 |  |
| regulation of glucokinase activity | 3 out of 2891 genes, 0.1% | 9 out of 15382 genes, 0.1% | 1 |  |
| mitotic DNA replication checkpoint | 3 out of 2891 genes, 0.1% | 9 out of 15382 genes, 0.1% | 1 |  |
| regulation of integrin activation | 3 out of 2891 genes, 0.1% | 9 out of 15382 genes, 0.1% | 1 |  |
| negative regulation of cell adhesion mediated by integrin | 3 out of 2891 genes, 0.1% | 9 out of 15382 genes, 0.1% | 1 |  |
| negative regulation of heterotypic cell-cell adhesion | 3 out of 2891 genes, 0.1% | 9 out of 15382 genes, 0.1% | 1 |  |
| histone-serine phosphorylation | 3 out of 2891 genes, 0.1% | 9 out of 15382 genes, 0.1% | 1 |  |
| peptidyl-threonine dephosphorylation | 3 out of 2891 genes, 0.1% | 9 out of 15382 genes, 0.1% | 1 |  |
| neuronal stem cell division | 3 out of 2891 genes, 0.1% | 9 out of 15382 genes, 0.1% | 1 |  |
| adhesion of symbiont to host cell | 3 out of 2891 genes, 0.1% | 9 out of 15382 genes, 0.1% | 1 |  |
| negative regulation of endothelial cell differentiation | 3 out of 2891 genes, 0.1% | 9 out of 15382 genes, 0.1% | 1 |  |
| negative regulation of erythrocyte differentiation | 3 out of 2891 genes, 0.1% | 9 out of 15382 genes, 0.1% | 1 |  |
| regulation of low-density lipoprotein particle receptor biosynthetic process | 3 out of 2891 genes, 0.1% | 9 out of 15382 genes, 0.1% | 1 |  |
| hepatocyte growth factor receptor signaling pathway | 3 out of 2891 genes, 0.1% | 9 out of 15382 genes, 0.1% | 1 |  |
| Golgi inheritance | 3 out of 2891 genes, 0.1% | 9 out of 15382 genes, 0.1% | 1 |  |
| negative regulation of release of sequestered calcium ion into cytosol | 3 out of 2891 genes, 0.1% | 9 out of 15382 genes, 0.1% | 1 |  |
| positive regulation of sequestering of calcium ion | 3 out of 2891 genes, 0.1% | 9 out of 15382 genes, 0.1% | 1 |  |
| myoblast migration | 3 out of 2891 genes, 0.1% | 9 out of 15382 genes, 0.1% | 1 |  |
| neuroblast division | 3 out of 2891 genes, 0.1% | 9 out of 15382 genes, 0.1% | 1 |  |
| anatomical structure regression | 3 out of 2891 genes, 0.1% | 9 out of 15382 genes, 0.1% | 1 |  |
| membrane hyperpolarization | 3 out of 2891 genes, 0.1% | 9 out of 15382 genes, 0.1% | 1 |  |
| regulation of dopamine receptor signaling pathway | 3 out of 2891 genes, 0.1% | 9 out of 15382 genes, 0.1% | 1 |  |
| female mating behavior | 3 out of 2891 genes, 0.1% | 9 out of 15382 genes, 0.1% | 1 |  |
| negative regulation of type I interferon-mediated signaling pathway | 3 out of 2891 genes, 0.1% | 9 out of 15382 genes, 0.1% | 1 |  |
| trachea morphogenesis | 3 out of 2891 genes, 0.1% | 9 out of 15382 genes, 0.1% | 1 |  |
| leukocyte aggregation | 3 out of 2891 genes, 0.1% | 9 out of 15382 genes, 0.1% | 1 |  |
| regulation of cell-cell adhesion involved in gastrulation | 3 out of 2891 genes, 0.1% | 9 out of 15382 genes, 0.1% | 1 |  |
| histone H3-K27 methylation | 3 out of 2891 genes, 0.1% | 9 out of 15382 genes, 0.1% | 1 |  |
| histone H3 deacetylation | 3 out of 2891 genes, 0.1% | 9 out of 15382 genes, 0.1% | 1 |  |
| glomerular visceral epithelial cell development | 3 out of 2891 genes, 0.1% | 9 out of 15382 genes, 0.1% | 1 |  |
| response to cisplatin | 3 out of 2891 genes, 0.1% | 9 out of 15382 genes, 0.1% | 1 |  |
| positive regulation of cell aging | 3 out of 2891 genes, 0.1% | 9 out of 15382 genes, 0.1% | 1 |  |
| mitochondrial respiratory chain complex III biogenesis | 3 out of 2891 genes, 0.1% | 9 out of 15382 genes, 0.1% | 1 |  |
| activation of cysteine-type endopeptidase activity | 3 out of 2891 genes, 0.1% | 9 out of 15382 genes, 0.1% | 1 |  |
| renal filtration | 3 out of 2891 genes, 0.1% | 9 out of 15382 genes, 0.1% | 1 |  |
| regulation of endothelial cell development | 3 out of 2891 genes, 0.1% | 9 out of 15382 genes, 0.1% | 1 |  |
| regulation of postsynaptic membrane organization | 3 out of 2891 genes, 0.1% | 9 out of 15382 genes, 0.1% | 1 |  |
| regulation of non-motile cilium assembly | 3 out of 2891 genes, 0.1% | 9 out of 15382 genes, 0.1% | 1 |  |
| regulation of establishment of endothelial barrier | 3 out of 2891 genes, 0.1% | 9 out of 15382 genes, 0.1% | 1 |  |
| negative regulation of stem cell proliferation | 3 out of 2891 genes, 0.1% | 9 out of 15382 genes, 0.1% | 1 |  |
| positive regulation of production of molecular mediator of immune response | 16 out of 2891 genes, 0.6% | 70 out of 15382 genes, 0.5% | 1 |  |
| smoothened signaling pathway | 16 out of 2891 genes, 0.6% | 70 out of 15382 genes, 0.5% | 1 |  |
| negative regulation of transmembrane transport | 16 out of 2891 genes, 0.6% | 70 out of 15382 genes, 0.5% | 1 |  |
| regulation of organ growth | 16 out of 2891 genes, 0.6% | 70 out of 15382 genes, 0.5% | 1 |  |
| spindle assembly | 16 out of 2891 genes, 0.6% | 70 out of 15382 genes, 0.5% | 1 |  |
| nucleotide-excision repair, DNA incision, 5'-to lesion | 7 out of 2891 genes, 0.2% | 27 out of 15382 genes, 0.2% | 1 |  |
| anterograde axonal transport | 7 out of 2891 genes, 0.2% | 27 out of 15382 genes, 0.2% | 1 |  |
| myotube cell development | 7 out of 2891 genes, 0.2% | 27 out of 15382 genes, 0.2% | 1 |  |
| muscle adaptation | 7 out of 2891 genes, 0.2% | 27 out of 15382 genes, 0.2% | 1 |  |
| positive regulation of translational initiation | 7 out of 2891 genes, 0.2% | 27 out of 15382 genes, 0.2% | 1 |  |
| response to growth hormone | 7 out of 2891 genes, 0.2% | 27 out of 15382 genes, 0.2% | 1 |  |
| dendritic cell differentiation | 7 out of 2891 genes, 0.2% | 27 out of 15382 genes, 0.2% | 1 |  |
| positive regulation of CD4-positive, alpha-beta T cell activation | 7 out of 2891 genes, 0.2% | 27 out of 15382 genes, 0.2% | 1 |  |
| epithelium migration | 17 out of 2891 genes, 0.6% | 75 out of 15382 genes, 0.5% | 1 |  |
| protein oligomerization | 89 out of 2891 genes, 3.1% | 440 out of 15382 genes, 2.9% | 1 |  |
| hematopoietic progenitor cell differentiation | 18 out of 2891 genes, 0.6% | 80 out of 15382 genes, 0.5% | 1 |  |
| regulation of leukocyte proliferation | 41 out of 2891 genes, 1.4% | 195 out of 15382 genes, 1.3% | 1 |  |
| pigmentation | 19 out of 2891 genes, 0.7% | 85 out of 15382 genes, 0.6% | 1 |  |
| regulation of neuron apoptotic process | 37 out of 2891 genes, 1.3% | 175 out of 15382 genes, 1.1% | 1 |  |
| sulfur amino acid transport | 2 out of 2891 genes, 0.1% | 5 out of 15382 genes, 0.0% | 1 |  |
| neural plate morphogenesis | 2 out of 2891 genes, 0.1% | 5 out of 15382 genes, 0.0% | 1 |  |
| somatic diversification of T cell receptor genes | 2 out of 2891 genes, 0.1% | 5 out of 15382 genes, 0.0% | 1 |  |
| somatic recombination of T cell receptor gene segments | 2 out of 2891 genes, 0.1% | 5 out of 15382 genes, 0.0% | 1 |  |
| renal sodium ion transport | 2 out of 2891 genes, 0.1% | 5 out of 15382 genes, 0.0% | 1 |  |
| tricuspid valve development | 2 out of 2891 genes, 0.1% | 5 out of 15382 genes, 0.0% | 1 |  |
| fructose 2,6-bisphosphate metabolic process | 2 out of 2891 genes, 0.1% | 5 out of 15382 genes, 0.0% | 1 |  |
| isocitrate metabolic process | 2 out of 2891 genes, 0.1% | 5 out of 15382 genes, 0.0% | 1 |  |
| base-excision repair, gap-filling | 2 out of 2891 genes, 0.1% | 5 out of 15382 genes, 0.0% | 1 |  |
| isoleucine metabolic process | 2 out of 2891 genes, 0.1% | 5 out of 15382 genes, 0.0% | 1 |  |
| posttranslational protein targeting to endoplasmic reticulum membrane | 2 out of 2891 genes, 0.1% | 5 out of 15382 genes, 0.0% | 1 |  |
| transmembrane receptor protein tyrosine phosphatase signaling pathway | 2 out of 2891 genes, 0.1% | 5 out of 15382 genes, 0.0% | 1 |  |
| granzyme-mediated apoptotic signaling pathway | 2 out of 2891 genes, 0.1% | 5 out of 15382 genes, 0.0% | 1 |  |
| dosage compensation by inactivation of X chromosome | 2 out of 2891 genes, 0.1% | 5 out of 15382 genes, 0.0% | 1 |  |
| purine deoxyribonucleotide catabolic process | 2 out of 2891 genes, 0.1% | 5 out of 15382 genes, 0.0% | 1 |  |
| deoxyribonucleoside monophosphate biosynthetic process | 2 out of 2891 genes, 0.1% | 5 out of 15382 genes, 0.0% | 1 |  |
| pyrimidine deoxyribonucleoside monophosphate biosynthetic process | 2 out of 2891 genes, 0.1% | 5 out of 15382 genes, 0.0% | 1 |  |
| purine deoxyribonucleoside triphosphate catabolic process | 2 out of 2891 genes, 0.1% | 5 out of 15382 genes, 0.0% | 1 |  |
| response to fructose | 2 out of 2891 genes, 0.1% | 5 out of 15382 genes, 0.0% | 1 |  |
| response to aluminum ion | 2 out of 2891 genes, 0.1% | 5 out of 15382 genes, 0.0% | 1 |  |
| negative regulation of phospholipase activity | 2 out of 2891 genes, 0.1% | 5 out of 15382 genes, 0.0% | 1 |  |
| negative regulation of mitochondrial fusion | 2 out of 2891 genes, 0.1% | 5 out of 15382 genes, 0.0% | 1 |  |
| negative regulation of receptor biosynthetic process | 2 out of 2891 genes, 0.1% | 5 out of 15382 genes, 0.0% | 1 |  |
| prostaglandin transport | 2 out of 2891 genes, 0.1% | 5 out of 15382 genes, 0.0% | 1 |  |
| synaptic vesicle uncoating | 2 out of 2891 genes, 0.1% | 5 out of 15382 genes, 0.0% | 1 |  |
| fusion of virus membrane with host plasma membrane | 2 out of 2891 genes, 0.1% | 5 out of 15382 genes, 0.0% | 1 |  |
| oxalate transport | 2 out of 2891 genes, 0.1% | 5 out of 15382 genes, 0.0% | 1 |  |
| parallel actin filament bundle assembly | 2 out of 2891 genes, 0.1% | 5 out of 15382 genes, 0.0% | 1 |  |
| autophagy of peroxisome | 2 out of 2891 genes, 0.1% | 5 out of 15382 genes, 0.0% | 1 |  |
| apoptotic chromosome condensation | 2 out of 2891 genes, 0.1% | 5 out of 15382 genes, 0.0% | 1 |  |
| transepithelial chloride transport | 2 out of 2891 genes, 0.1% | 5 out of 15382 genes, 0.0% | 1 |  |
| negative regulation of interleukin-4 production | 2 out of 2891 genes, 0.1% | 5 out of 15382 genes, 0.0% | 1 |  |
| regulation of neutrophil apoptotic process | 2 out of 2891 genes, 0.1% | 5 out of 15382 genes, 0.0% | 1 |  |
| T cell receptor V(D)J recombination | 2 out of 2891 genes, 0.1% | 5 out of 15382 genes, 0.0% | 1 |  |
| secretion of lysosomal enzymes | 2 out of 2891 genes, 0.1% | 5 out of 15382 genes, 0.0% | 1 |  |
| DNA replication, Okazaki fragment processing | 2 out of 2891 genes, 0.1% | 5 out of 15382 genes, 0.0% | 1 |  |
| positive regulation of maintenance of sister chromatid cohesion | 2 out of 2891 genes, 0.1% | 5 out of 15382 genes, 0.0% | 1 |  |
| positive regulation of maintenance of mitotic sister chromatid cohesion | 2 out of 2891 genes, 0.1% | 5 out of 15382 genes, 0.0% | 1 |  |
| 'de novo' NAD biosynthetic process | 2 out of 2891 genes, 0.1% | 5 out of 15382 genes, 0.0% | 1 |  |
| extracellular matrix-cell signaling | 2 out of 2891 genes, 0.1% | 5 out of 15382 genes, 0.0% | 1 |  |
| mitochondrial protein catabolic process | 2 out of 2891 genes, 0.1% | 5 out of 15382 genes, 0.0% | 1 |  |
| histone H3-K9 trimethylation | 2 out of 2891 genes, 0.1% | 5 out of 15382 genes, 0.0% | 1 |  |
| positive regulation of cell migration by vascular endothelial growth factor signaling pathway | 2 out of 2891 genes, 0.1% | 5 out of 15382 genes, 0.0% | 1 |  |
| membrane fusion involved in viral entry into host cell | 2 out of 2891 genes, 0.1% | 5 out of 15382 genes, 0.0% | 1 |  |
| polar body extrusion after meiotic divisions | 2 out of 2891 genes, 0.1% | 5 out of 15382 genes, 0.0% | 1 |  |
| negative regulation of hair cycle | 2 out of 2891 genes, 0.1% | 5 out of 15382 genes, 0.0% | 1 |  |
| regulation of mesodermal cell fate specification | 2 out of 2891 genes, 0.1% | 5 out of 15382 genes, 0.0% | 1 |  |
| very long-chain fatty acid catabolic process | 2 out of 2891 genes, 0.1% | 5 out of 15382 genes, 0.0% | 1 |  |
| activation of Janus kinase activity | 2 out of 2891 genes, 0.1% | 5 out of 15382 genes, 0.0% | 1 |  |
| positive regulation of memory T cell differentiation | 2 out of 2891 genes, 0.1% | 5 out of 15382 genes, 0.0% | 1 |  |
| Wnt signaling pathway involved in dorsal/ventral axis specification | 2 out of 2891 genes, 0.1% | 5 out of 15382 genes, 0.0% | 1 |  |
| multi-organism membrane fusion | 2 out of 2891 genes, 0.1% | 5 out of 15382 genes, 0.0% | 1 |  |
| negative regulation by host of viral genome replication | 2 out of 2891 genes, 0.1% | 5 out of 15382 genes, 0.0% | 1 |  |
| protein import into peroxisome membrane | 2 out of 2891 genes, 0.1% | 5 out of 15382 genes, 0.0% | 1 |  |
| negative regulation of sister chromatid cohesion | 2 out of 2891 genes, 0.1% | 5 out of 15382 genes, 0.0% | 1 |  |
| dATP metabolic process | 2 out of 2891 genes, 0.1% | 5 out of 15382 genes, 0.0% | 1 |  |
| dUMP metabolic process | 2 out of 2891 genes, 0.1% | 5 out of 15382 genes, 0.0% | 1 |  |
| response to cycloheximide | 2 out of 2891 genes, 0.1% | 5 out of 15382 genes, 0.0% | 1 |  |
| regulation of isotype switching to IgA isotypes | 2 out of 2891 genes, 0.1% | 5 out of 15382 genes, 0.0% | 1 |  |
| intracellular distribution of mitochondria | 2 out of 2891 genes, 0.1% | 5 out of 15382 genes, 0.0% | 1 |  |
| mesendoderm development | 2 out of 2891 genes, 0.1% | 5 out of 15382 genes, 0.0% | 1 |  |
| positive regulation of skeletal muscle tissue growth | 2 out of 2891 genes, 0.1% | 5 out of 15382 genes, 0.0% | 1 |  |
| activated T cell proliferation | 2 out of 2891 genes, 0.1% | 5 out of 15382 genes, 0.0% | 1 |  |
| negative regulation of lipoprotein lipase activity | 2 out of 2891 genes, 0.1% | 5 out of 15382 genes, 0.0% | 1 |  |
| positive regulation of neurotrophin TRK receptor signaling pathway | 2 out of 2891 genes, 0.1% | 5 out of 15382 genes, 0.0% | 1 |  |
| detoxification of nitrogen compound | 2 out of 2891 genes, 0.1% | 5 out of 15382 genes, 0.0% | 1 |  |
| response to hydrostatic pressure | 2 out of 2891 genes, 0.1% | 5 out of 15382 genes, 0.0% | 1 |  |
| negative regulation of hair follicle development | 2 out of 2891 genes, 0.1% | 5 out of 15382 genes, 0.0% | 1 |  |
| regulation of Fc receptor mediated stimulatory signaling pathway | 2 out of 2891 genes, 0.1% | 5 out of 15382 genes, 0.0% | 1 |  |
| trachea formation | 2 out of 2891 genes, 0.1% | 5 out of 15382 genes, 0.0% | 1 |  |
| lung lobe development | 2 out of 2891 genes, 0.1% | 5 out of 15382 genes, 0.0% | 1 |  |
| lung lobe morphogenesis | 2 out of 2891 genes, 0.1% | 5 out of 15382 genes, 0.0% | 1 |  |
| Type I pneumocyte differentiation | 2 out of 2891 genes, 0.1% | 5 out of 15382 genes, 0.0% | 1 |  |
| ciliary body morphogenesis | 2 out of 2891 genes, 0.1% | 5 out of 15382 genes, 0.0% | 1 |  |
| ciliary neurotrophic factor-mediated signaling pathway | 2 out of 2891 genes, 0.1% | 5 out of 15382 genes, 0.0% | 1 |  |
| thrombin-activated receptor signaling pathway | 2 out of 2891 genes, 0.1% | 5 out of 15382 genes, 0.0% | 1 |  |
| regulation of vitamin D receptor signaling pathway | 2 out of 2891 genes, 0.1% | 5 out of 15382 genes, 0.0% | 1 |  |
| autophagy of host cells involved in interaction with symbiont | 2 out of 2891 genes, 0.1% | 5 out of 15382 genes, 0.0% | 1 |  |
| autophagy involved in symbiotic interaction | 2 out of 2891 genes, 0.1% | 5 out of 15382 genes, 0.0% | 1 |  |
| viral translational termination-reinitiation | 2 out of 2891 genes, 0.1% | 5 out of 15382 genes, 0.0% | 1 |  |
| regulation of mitochondrial DNA replication | 2 out of 2891 genes, 0.1% | 5 out of 15382 genes, 0.0% | 1 |  |
| cellular lipid biosynthetic process | 2 out of 2891 genes, 0.1% | 5 out of 15382 genes, 0.0% | 1 |  |
| exocytic insertion of neurotransmitter receptor to plasma membrane | 2 out of 2891 genes, 0.1% | 5 out of 15382 genes, 0.0% | 1 |  |
| neurotransmitter receptor transport, endosome to postsynaptic membrane | 2 out of 2891 genes, 0.1% | 5 out of 15382 genes, 0.0% | 1 |  |
| regulation of membrane repolarization during action potential | 2 out of 2891 genes, 0.1% | 5 out of 15382 genes, 0.0% | 1 |  |
| membrane repolarization during ventricular cardiac muscle cell action potential | 2 out of 2891 genes, 0.1% | 5 out of 15382 genes, 0.0% | 1 |  |
| exocytic insertion of neurotransmitter receptor to postsynaptic membrane | 2 out of 2891 genes, 0.1% | 5 out of 15382 genes, 0.0% | 1 |  |
| trans-synaptic signaling, modulating synaptic transmission | 2 out of 2891 genes, 0.1% | 5 out of 15382 genes, 0.0% | 1 |  |
| intestinal hexose absorption | 2 out of 2891 genes, 0.1% | 5 out of 15382 genes, 0.0% | 1 |  |
| regulation of basement membrane organization | 2 out of 2891 genes, 0.1% | 5 out of 15382 genes, 0.0% | 1 |  |
| negative regulation of ruffle assembly | 2 out of 2891 genes, 0.1% | 5 out of 15382 genes, 0.0% | 1 |  |
| regulation of peptidyl-tyrosine autophosphorylation | 2 out of 2891 genes, 0.1% | 5 out of 15382 genes, 0.0% | 1 |  |
| regulation of phospholipase C-activating G-protein coupled receptor signaling pathway | 2 out of 2891 genes, 0.1% | 5 out of 15382 genes, 0.0% | 1 |  |
| negative regulation of hematopoietic progenitor cell differentiation | 2 out of 2891 genes, 0.1% | 5 out of 15382 genes, 0.0% | 1 |  |
| regulation of histone deacetylase activity | 2 out of 2891 genes, 0.1% | 5 out of 15382 genes, 0.0% | 1 |  |
| negative regulation of peptidyl-cysteine S-nitrosylation | 2 out of 2891 genes, 0.1% | 5 out of 15382 genes, 0.0% | 1 |  |
| positive regulation of delayed rectifier potassium channel activity | 2 out of 2891 genes, 0.1% | 5 out of 15382 genes, 0.0% | 1 |  |
| negative regulation of stem cell population maintenance | 2 out of 2891 genes, 0.1% | 5 out of 15382 genes, 0.0% | 1 |  |
| negative regulation of cellular response to vascular endothelial growth factor stimulus | 2 out of 2891 genes, 0.1% | 5 out of 15382 genes, 0.0% | 1 |  |
| response to fluoride | 2 out of 2891 genes, 0.1% | 5 out of 15382 genes, 0.0% | 1 |  |
| negative regulation of lamellipodium organization | 2 out of 2891 genes, 0.1% | 5 out of 15382 genes, 0.0% | 1 |  |
| melanosome assembly | 2 out of 2891 genes, 0.1% | 5 out of 15382 genes, 0.0% | 1 |  |
| positive regulation of cell maturation | 2 out of 2891 genes, 0.1% | 5 out of 15382 genes, 0.0% | 1 |  |
| regulation of blood vessel endothelial cell proliferation involved in sprouting angiogenesis | 2 out of 2891 genes, 0.1% | 5 out of 15382 genes, 0.0% | 1 |  |
| regulation of adipose tissue development | 2 out of 2891 genes, 0.1% | 5 out of 15382 genes, 0.0% | 1 |  |
| regulation of basement membrane assembly involved in embryonic body morphogenesis | 2 out of 2891 genes, 0.1% | 5 out of 15382 genes, 0.0% | 1 |  |
| positive regulation of basement membrane assembly involved in embryonic body morphogenesis | 2 out of 2891 genes, 0.1% | 5 out of 15382 genes, 0.0% | 1 |  |
| regulation of lipophagy | 2 out of 2891 genes, 0.1% | 5 out of 15382 genes, 0.0% | 1 |  |
| positive regulation of lipophagy | 2 out of 2891 genes, 0.1% | 5 out of 15382 genes, 0.0% | 1 |  |
| brush border assembly | 2 out of 2891 genes, 0.1% | 5 out of 15382 genes, 0.0% | 1 |  |
| negative regulation of guanyl-nucleotide exchange factor activity | 2 out of 2891 genes, 0.1% | 5 out of 15382 genes, 0.0% | 1 |  |
| regulation of mesodermal cell differentiation | 2 out of 2891 genes, 0.1% | 5 out of 15382 genes, 0.0% | 1 |  |
| regulation of mesoderm formation | 2 out of 2891 genes, 0.1% | 5 out of 15382 genes, 0.0% | 1 |  |
| negative regulation of transcription from RNA polymerase II promoter in response to endoplasmic reticulum stress | 2 out of 2891 genes, 0.1% | 5 out of 15382 genes, 0.0% | 1 |  |
| dense core granule exocytosis | 2 out of 2891 genes, 0.1% | 5 out of 15382 genes, 0.0% | 1 |  |
| negative regulation of cell-cell adhesion mediated by cadherin | 2 out of 2891 genes, 0.1% | 5 out of 15382 genes, 0.0% | 1 |  |
| negative regulation of DNA-templated transcription, initiation | 2 out of 2891 genes, 0.1% | 5 out of 15382 genes, 0.0% | 1 |  |
| regulation of CD40 signaling pathway | 2 out of 2891 genes, 0.1% | 5 out of 15382 genes, 0.0% | 1 |  |
| positive regulation of cAMP-dependent protein kinase activity | 2 out of 2891 genes, 0.1% | 5 out of 15382 genes, 0.0% | 1 |  |
| regulation of interleukin-1-mediated signaling pathway | 2 out of 2891 genes, 0.1% | 5 out of 15382 genes, 0.0% | 1 |  |
| positive regulation of double-strand break repair via nonhomologous end joining | 2 out of 2891 genes, 0.1% | 5 out of 15382 genes, 0.0% | 1 |  |
| regulation of DNA binding | 21 out of 2891 genes, 0.7% | 95 out of 15382 genes, 0.6% | 1 |  |
| establishment of mitotic spindle orientation | 5 out of 2891 genes, 0.2% | 18 out of 15382 genes, 0.1% | 1 |  |
| myoblast fusion | 5 out of 2891 genes, 0.2% | 18 out of 15382 genes, 0.1% | 1 |  |
| positive regulation of cardiac muscle cell proliferation | 5 out of 2891 genes, 0.2% | 18 out of 15382 genes, 0.1% | 1 |  |
| lymphocyte apoptotic process | 5 out of 2891 genes, 0.2% | 18 out of 15382 genes, 0.1% | 1 |  |
| 3'-UTR-mediated mRNA stabilization | 5 out of 2891 genes, 0.2% | 18 out of 15382 genes, 0.1% | 1 |  |
| cellular response to gamma radiation | 5 out of 2891 genes, 0.2% | 18 out of 15382 genes, 0.1% | 1 |  |
| regulation of mitotic spindle assembly | 5 out of 2891 genes, 0.2% | 18 out of 15382 genes, 0.1% | 1 |  |
| regulation of anion transmembrane transport | 5 out of 2891 genes, 0.2% | 18 out of 15382 genes, 0.1% | 1 |  |
| positive regulation of lipid metabolic process | 22 out of 2891 genes, 0.8% | 100 out of 15382 genes, 0.7% | 1 |  |
| positive regulation of establishment of protein localization to mitochondrion | 22 out of 2891 genes, 0.8% | 100 out of 15382 genes, 0.7% | 1 |  |
| spliceosomal complex assembly | 12 out of 2891 genes, 0.4% | 51 out of 15382 genes, 0.3% | 1 |  |
| embryonic heart tube morphogenesis | 12 out of 2891 genes, 0.4% | 51 out of 15382 genes, 0.3% | 1 |  |
| positive regulation of osteoblast differentiation | 12 out of 2891 genes, 0.4% | 51 out of 15382 genes, 0.3% | 1 |  |
| positive regulation of striated muscle tissue development | 12 out of 2891 genes, 0.4% | 51 out of 15382 genes, 0.3% | 1 |  |
| positive regulation of muscle organ development | 12 out of 2891 genes, 0.4% | 51 out of 15382 genes, 0.3% | 1 |  |
| organic acid transmembrane transport | 12 out of 2891 genes, 0.4% | 51 out of 15382 genes, 0.3% | 1 |  |
| carboxylic acid transmembrane transport | 12 out of 2891 genes, 0.4% | 51 out of 15382 genes, 0.3% | 1 |  |
| cholesterol metabolic process | 23 out of 2891 genes, 0.8% | 105 out of 15382 genes, 0.7% | 1 |  |
| specification of symmetry | 23 out of 2891 genes, 0.8% | 105 out of 15382 genes, 0.7% | 1 |  |
| determination of bilateral symmetry | 23 out of 2891 genes, 0.8% | 105 out of 15382 genes, 0.7% | 1 |  |
| anion transmembrane transport | 23 out of 2891 genes, 0.8% | 105 out of 15382 genes, 0.7% | 1 |  |
| B cell activation | 29 out of 2891 genes, 1.0% | 135 out of 15382 genes, 0.9% | 1 |  |
| nuclear-transcribed mRNA catabolic process, exonucleolytic | 8 out of 2891 genes, 0.3% | 32 out of 15382 genes, 0.2% | 1 |  |
| NADP metabolic process | 8 out of 2891 genes, 0.3% | 32 out of 15382 genes, 0.2% | 1 |  |
| neuron maturation | 8 out of 2891 genes, 0.3% | 32 out of 15382 genes, 0.2% | 1 |  |
| myoblast differentiation | 8 out of 2891 genes, 0.3% | 32 out of 15382 genes, 0.2% | 1 |  |
| actin filament capping | 8 out of 2891 genes, 0.3% | 32 out of 15382 genes, 0.2% | 1 |  |
| cellular developmental process | 659 out of 2891 genes, 22.8% | 3429 out of 15382 genes, 22.3% | 1 |  |
| vasculogenesis | 13 out of 2891 genes, 0.4% | 56 out of 15382 genes, 0.4% | 1 |  |
| mesoderm morphogenesis | 14 out of 2891 genes, 0.5% | 61 out of 15382 genes, 0.4% | 1 |  |
| cell redox homeostasis | 15 out of 2891 genes, 0.5% | 66 out of 15382 genes, 0.4% | 1 |  |
| negative regulation of immune system process | 76 out of 2891 genes, 2.6% | 375 out of 15382 genes, 2.4% | 1 |  |
| polysaccharide biosynthetic process | 9 out of 2891 genes, 0.3% | 37 out of 15382 genes, 0.2% | 1 |  |
| cell-substrate junction assembly | 9 out of 2891 genes, 0.3% | 37 out of 15382 genes, 0.2% | 1 |  |
| bicarbonate transport | 9 out of 2891 genes, 0.3% | 37 out of 15382 genes, 0.2% | 1 |  |
| positive regulation of B cell proliferation | 9 out of 2891 genes, 0.3% | 37 out of 15382 genes, 0.2% | 1 |  |
| negative regulation of muscle cell differentiation | 9 out of 2891 genes, 0.3% | 37 out of 15382 genes, 0.2% | 1 |  |
| positive regulation of protein modification by small protein conjugation or removal | 37 out of 2891 genes, 1.3% | 176 out of 15382 genes, 1.1% | 1 |  |
| positive regulation of adaptive immune response based on somatic recombination of immune receptors built from immunoglobulin superfamily domains | 16 out of 2891 genes, 0.6% | 71 out of 15382 genes, 0.5% | 1 |  |
| triglyceride metabolic process | 16 out of 2891 genes, 0.6% | 71 out of 15382 genes, 0.5% | 1 |  |
| centrosome cycle | 16 out of 2891 genes, 0.6% | 71 out of 15382 genes, 0.5% | 1 |  |
| steroid hormone mediated signaling pathway | 16 out of 2891 genes, 0.6% | 71 out of 15382 genes, 0.5% | 1 |  |
| response to extracellular stimulus | 87 out of 2891 genes, 3.0% | 432 out of 15382 genes, 2.8% | 1 |  |
| tissue migration | 18 out of 2891 genes, 0.6% | 81 out of 15382 genes, 0.5% | 1 |  |
| negative regulation of leukocyte activation | 30 out of 2891 genes, 1.0% | 141 out of 15382 genes, 0.9% | 1 |  |
| cGMP biosynthetic process | 6 out of 2891 genes, 0.2% | 23 out of 15382 genes, 0.1% | 1 |  |
| branched-chain amino acid metabolic process | 6 out of 2891 genes, 0.2% | 23 out of 15382 genes, 0.1% | 1 |  |
| somatic recombination of immunoglobulin gene segments | 6 out of 2891 genes, 0.2% | 23 out of 15382 genes, 0.1% | 1 |  |
| cerebellar cortex formation | 6 out of 2891 genes, 0.2% | 23 out of 15382 genes, 0.1% | 1 |  |
| myelination in peripheral nervous system | 6 out of 2891 genes, 0.2% | 23 out of 15382 genes, 0.1% | 1 |  |
| lipopolysaccharide-mediated signaling pathway | 6 out of 2891 genes, 0.2% | 23 out of 15382 genes, 0.1% | 1 |  |
| peripheral nervous system axon ensheathment | 6 out of 2891 genes, 0.2% | 23 out of 15382 genes, 0.1% | 1 |  |
| response to fluid shear stress | 6 out of 2891 genes, 0.2% | 23 out of 15382 genes, 0.1% | 1 |  |
| ncRNA catabolic process | 6 out of 2891 genes, 0.2% | 23 out of 15382 genes, 0.1% | 1 |  |
| negative regulation of organ growth | 6 out of 2891 genes, 0.2% | 23 out of 15382 genes, 0.1% | 1 |  |
| smooth muscle cell differentiation | 6 out of 2891 genes, 0.2% | 23 out of 15382 genes, 0.1% | 1 |  |
| regulation of long-term synaptic potentiation | 6 out of 2891 genes, 0.2% | 23 out of 15382 genes, 0.1% | 1 |  |
| glutathione derivative metabolic process | 6 out of 2891 genes, 0.2% | 23 out of 15382 genes, 0.1% | 1 |  |
| glutathione derivative biosynthetic process | 6 out of 2891 genes, 0.2% | 23 out of 15382 genes, 0.1% | 1 |  |
| regulation of ERAD pathway | 6 out of 2891 genes, 0.2% | 23 out of 15382 genes, 0.1% | 1 |  |
| cell maturation | 29 out of 2891 genes, 1.0% | 136 out of 15382 genes, 0.9% | 1 |  |
| purine nucleotide catabolic process | 10 out of 2891 genes, 0.3% | 42 out of 15382 genes, 0.3% | 1 |  |
| response to gamma radiation | 10 out of 2891 genes, 0.3% | 42 out of 15382 genes, 0.3% | 1 |  |
| hindbrain morphogenesis | 10 out of 2891 genes, 0.3% | 42 out of 15382 genes, 0.3% | 1 |  |
| DNA geometric change | 10 out of 2891 genes, 0.3% | 42 out of 15382 genes, 0.3% | 1 |  |
| response to antibiotic | 10 out of 2891 genes, 0.3% | 42 out of 15382 genes, 0.3% | 1 |  |
| cellular response to transforming growth factor beta stimulus | 27 out of 2891 genes, 0.9% | 126 out of 15382 genes, 0.8% | 1 |  |
| positive regulation of mitotic cell cycle | 26 out of 2891 genes, 0.9% | 121 out of 15382 genes, 0.8% | 1 |  |
| positive regulation of NF-kappaB transcription factor activity | 26 out of 2891 genes, 0.9% | 121 out of 15382 genes, 0.8% | 1 |  |
| secondary alcohol metabolic process | 23 out of 2891 genes, 0.8% | 106 out of 15382 genes, 0.7% | 1 |  |
| regulation of canonical Wnt signaling pathway | 43 out of 2891 genes, 1.5% | 207 out of 15382 genes, 1.3% | 1 |  |
| intracellular protein transmembrane transport | 11 out of 2891 genes, 0.4% | 47 out of 15382 genes, 0.3% | 1 |  |
| positive regulation of protein serine/threonine kinase activity | 59 out of 2891 genes, 2.0% | 289 out of 15382 genes, 1.9% | 1 |  |
| nucleotide-excision repair, DNA damage recognition | 4 out of 2891 genes, 0.1% | 14 out of 15382 genes, 0.1% | 1 |  |
| auditory receptor cell morphogenesis | 4 out of 2891 genes, 0.1% | 14 out of 15382 genes, 0.1% | 1 |  |
| cAMP catabolic process | 4 out of 2891 genes, 0.1% | 14 out of 15382 genes, 0.1% | 1 |  |
| N-terminal protein amino acid acetylation | 4 out of 2891 genes, 0.1% | 14 out of 15382 genes, 0.1% | 1 |  |
| mitotic sister chromatid cohesion | 4 out of 2891 genes, 0.1% | 14 out of 15382 genes, 0.1% | 1 |  |
| positive regulation of muscle cell apoptotic process | 4 out of 2891 genes, 0.1% | 14 out of 15382 genes, 0.1% | 1 |  |
| regulation of glutamate secretion | 4 out of 2891 genes, 0.1% | 14 out of 15382 genes, 0.1% | 1 |  |
| protein autoprocessing | 4 out of 2891 genes, 0.1% | 14 out of 15382 genes, 0.1% | 1 |  |
| polarized epithelial cell differentiation | 4 out of 2891 genes, 0.1% | 14 out of 15382 genes, 0.1% | 1 |  |
| response to magnesium ion | 4 out of 2891 genes, 0.1% | 14 out of 15382 genes, 0.1% | 1 |  |
| negative regulation of organic acid transport | 4 out of 2891 genes, 0.1% | 14 out of 15382 genes, 0.1% | 1 |  |
| cellular response to interferon-beta | 4 out of 2891 genes, 0.1% | 14 out of 15382 genes, 0.1% | 1 |  |
| meiotic telomere clustering | 4 out of 2891 genes, 0.1% | 14 out of 15382 genes, 0.1% | 1 |  |
| ventricular cardiac muscle cell differentiation | 4 out of 2891 genes, 0.1% | 14 out of 15382 genes, 0.1% | 1 |  |
| response to hyperoxia | 4 out of 2891 genes, 0.1% | 14 out of 15382 genes, 0.1% | 1 |  |
| regulation of necroptotic process | 4 out of 2891 genes, 0.1% | 14 out of 15382 genes, 0.1% | 1 |  |
| error-free translesion synthesis | 4 out of 2891 genes, 0.1% | 14 out of 15382 genes, 0.1% | 1 |  |
| negative regulation of potassium ion transmembrane transport | 4 out of 2891 genes, 0.1% | 14 out of 15382 genes, 0.1% | 1 |  |
| positive regulation of double-strand break repair | 4 out of 2891 genes, 0.1% | 14 out of 15382 genes, 0.1% | 1 |  |
| multicellular organism development | 876 out of 2891 genes, 30.3% | 4583 out of 15382 genes, 29.8% | 1 |  |
| regulation of glial cell differentiation | 12 out of 2891 genes, 0.4% | 52 out of 15382 genes, 0.3% | 1 |  |
| positive regulation of muscle tissue development | 12 out of 2891 genes, 0.4% | 52 out of 15382 genes, 0.3% | 1 |  |
| regulation of nuclear division | 35 out of 2891 genes, 1.2% | 167 out of 15382 genes, 1.1% | 1 |  |
| positive regulation of T cell mediated immunity | 7 out of 2891 genes, 0.2% | 28 out of 15382 genes, 0.2% | 1 |  |
| regulation of polysaccharide biosynthetic process | 7 out of 2891 genes, 0.2% | 28 out of 15382 genes, 0.2% | 1 |  |
| positive regulation of glial cell differentiation | 7 out of 2891 genes, 0.2% | 28 out of 15382 genes, 0.2% | 1 |  |
| negative regulation of JNK cascade | 7 out of 2891 genes, 0.2% | 28 out of 15382 genes, 0.2% | 1 |  |
| regulation of viral entry into host cell | 7 out of 2891 genes, 0.2% | 28 out of 15382 genes, 0.2% | 1 |  |
| intestinal absorption | 7 out of 2891 genes, 0.2% | 28 out of 15382 genes, 0.2% | 1 |  |
| response to reactive oxygen species | 32 out of 2891 genes, 1.1% | 152 out of 15382 genes, 1.0% | 1 |  |
| negative regulation of proteolysis | 44 out of 2891 genes, 1.5% | 213 out of 15382 genes, 1.4% | 1 |  |
| cellular response to UV | 14 out of 2891 genes, 0.5% | 62 out of 15382 genes, 0.4% | 1 |  |
| regulation of synapse structure or activity | 30 out of 2891 genes, 1.0% | 142 out of 15382 genes, 0.9% | 1 |  |
| regulation of synapse organization | 29 out of 2891 genes, 1.0% | 137 out of 15382 genes, 0.9% | 1 |  |
| negative regulation of NF-kappaB transcription factor activity | 15 out of 2891 genes, 0.5% | 67 out of 15382 genes, 0.4% | 1 |  |
| cellular polysaccharide metabolic process | 15 out of 2891 genes, 0.5% | 67 out of 15382 genes, 0.4% | 1 |  |
| somite development | 15 out of 2891 genes, 0.5% | 67 out of 15382 genes, 0.4% | 1 |  |
| hindbrain development | 28 out of 2891 genes, 1.0% | 132 out of 15382 genes, 0.9% | 1 |  |
| response to transforming growth factor beta | 28 out of 2891 genes, 1.0% | 132 out of 15382 genes, 0.9% | 1 |  |
| epithelial cell migration | 16 out of 2891 genes, 0.6% | 72 out of 15382 genes, 0.5% | 1 |  |
| chromosome localization | 16 out of 2891 genes, 0.6% | 72 out of 15382 genes, 0.5% | 1 |  |
| heart contraction | 16 out of 2891 genes, 0.6% | 72 out of 15382 genes, 0.5% | 1 |  |
| angiogenesis | 58 out of 2891 genes, 2.0% | 285 out of 15382 genes, 1.9% | 1 |  |
| cellular response to light stimulus | 18 out of 2891 genes, 0.6% | 82 out of 15382 genes, 0.5% | 1 |  |
| cell differentiation | 644 out of 2891 genes, 22.3% | 3359 out of 15382 genes, 21.8% | 1 |  |
| somatic diversification of immune receptors via germline recombination within a single locus | 8 out of 2891 genes, 0.3% | 33 out of 15382 genes, 0.2% | 1 |  |
| positive regulation of B cell mediated immunity | 8 out of 2891 genes, 0.3% | 33 out of 15382 genes, 0.2% | 1 |  |
| positive regulation of immunoglobulin mediated immune response | 8 out of 2891 genes, 0.3% | 33 out of 15382 genes, 0.2% | 1 |  |
| regulation of gastrulation | 8 out of 2891 genes, 0.3% | 33 out of 15382 genes, 0.2% | 1 |  |
| somatic cell DNA recombination | 8 out of 2891 genes, 0.3% | 33 out of 15382 genes, 0.2% | 1 |  |
| cerebellar cortex morphogenesis | 8 out of 2891 genes, 0.3% | 33 out of 15382 genes, 0.2% | 1 |  |
| negative regulation of chemotaxis | 8 out of 2891 genes, 0.3% | 33 out of 15382 genes, 0.2% | 1 |  |
| positive regulation of biomineral tissue development | 8 out of 2891 genes, 0.3% | 33 out of 15382 genes, 0.2% | 1 |  |
| positive regulation of ERBB signaling pathway | 8 out of 2891 genes, 0.3% | 33 out of 15382 genes, 0.2% | 1 |  |
| negative regulation of lipid localization | 8 out of 2891 genes, 0.3% | 33 out of 15382 genes, 0.2% | 1 |  |
| regulation of embryonic development | 22 out of 2891 genes, 0.8% | 102 out of 15382 genes, 0.7% | 1 |  |
| monocarboxylic acid catabolic process | 22 out of 2891 genes, 0.8% | 102 out of 15382 genes, 0.7% | 1 |  |
| positive regulation of transmembrane receptor protein serine/threonine kinase signaling pathway | 20 out of 2891 genes, 0.7% | 92 out of 15382 genes, 0.6% | 1 |  |
| regulation of stem cell differentiation | 20 out of 2891 genes, 0.7% | 92 out of 15382 genes, 0.6% | 1 |  |
| heterocycle catabolic process | 71 out of 2891 genes, 2.5% | 352 out of 15382 genes, 2.3% | 1 |  |
| negative regulation of developmental process | 154 out of 2891 genes, 5.3% | 783 out of 15382 genes, 5.1% | 1 |  |
| coenzyme metabolic process | 61 out of 2891 genes, 2.1% | 301 out of 15382 genes, 2.0% | 1 |  |
| peptidyl-tyrosine autophosphorylation | 9 out of 2891 genes, 0.3% | 38 out of 15382 genes, 0.2% | 1 |  |
| regulation of lipid kinase activity | 9 out of 2891 genes, 0.3% | 38 out of 15382 genes, 0.2% | 1 |  |
| mitotic spindle assembly | 9 out of 2891 genes, 0.3% | 38 out of 15382 genes, 0.2% | 1 |  |
| epithelial tube morphogenesis | 53 out of 2891 genes, 1.8% | 260 out of 15382 genes, 1.7% | 1 |  |
| negative regulation of neuron death | 34 out of 2891 genes, 1.2% | 163 out of 15382 genes, 1.1% | 1 |  |
| anatomical structure development | 940 out of 2891 genes, 32.5% | 4928 out of 15382 genes, 32.0% | 1 |  |
| outflow tract septum morphogenesis | 5 out of 2891 genes, 0.2% | 19 out of 15382 genes, 0.1% | 1 |  |
| zinc II ion transport | 5 out of 2891 genes, 0.2% | 19 out of 15382 genes, 0.1% | 1 |  |
| telomere maintenance via telomerase | 5 out of 2891 genes, 0.2% | 19 out of 15382 genes, 0.1% | 1 |  |
| ectoderm development | 5 out of 2891 genes, 0.2% | 19 out of 15382 genes, 0.1% | 1 |  |
| positive regulation of cardiac muscle hypertrophy | 5 out of 2891 genes, 0.2% | 19 out of 15382 genes, 0.1% | 1 |  |
| positive regulation of muscle hypertrophy | 5 out of 2891 genes, 0.2% | 19 out of 15382 genes, 0.1% | 1 |  |
| muscle cell migration | 5 out of 2891 genes, 0.2% | 19 out of 15382 genes, 0.1% | 1 |  |
| endoplasmic reticulum calcium ion homeostasis | 5 out of 2891 genes, 0.2% | 19 out of 15382 genes, 0.1% | 1 |  |
| regulation of T cell differentiation in thymus | 5 out of 2891 genes, 0.2% | 19 out of 15382 genes, 0.1% | 1 |  |
| regulation of heterotypic cell-cell adhesion | 5 out of 2891 genes, 0.2% | 19 out of 15382 genes, 0.1% | 1 |  |
| regulation of neuroblast proliferation | 5 out of 2891 genes, 0.2% | 19 out of 15382 genes, 0.1% | 1 |  |
| regulation of non-canonical Wnt signaling pathway | 5 out of 2891 genes, 0.2% | 19 out of 15382 genes, 0.1% | 1 |  |
| system development | 770 out of 2891 genes, 26.6% | 4028 out of 15382 genes, 26.2% | 1 |  |
| energy derivation by oxidation of organic compounds | 43 out of 2891 genes, 1.5% | 209 out of 15382 genes, 1.4% | 1 |  |
| signaling | 875 out of 2891 genes, 30.3% | 4584 out of 15382 genes, 29.8% | 1 |  |
| nuclear chromosome segregation | 51 out of 2891 genes, 1.8% | 250 out of 15382 genes, 1.6% | 1 |  |
| body morphogenesis | 10 out of 2891 genes, 0.3% | 43 out of 15382 genes, 0.3% | 1 |  |
| positive regulation of telomere maintenance | 10 out of 2891 genes, 0.3% | 43 out of 15382 genes, 0.3% | 1 |  |
| negative regulation of innate immune response | 10 out of 2891 genes, 0.3% | 43 out of 15382 genes, 0.3% | 1 |  |
| cellular nitrogen compound catabolic process | 71 out of 2891 genes, 2.5% | 353 out of 15382 genes, 2.3% | 1 |  |
| regulation of response to wounding | 29 out of 2891 genes, 1.0% | 138 out of 15382 genes, 0.9% | 1 |  |
| positive regulation of leukocyte cell-cell adhesion | 40 out of 2891 genes, 1.4% | 194 out of 15382 genes, 1.3% | 1 |  |
| heart looping | 11 out of 2891 genes, 0.4% | 48 out of 15382 genes, 0.3% | 1 |  |
| regulation of interleukin-2 production | 11 out of 2891 genes, 0.4% | 48 out of 15382 genes, 0.3% | 1 |  |
| regulation of neurotransmitter secretion | 11 out of 2891 genes, 0.4% | 48 out of 15382 genes, 0.3% | 1 |  |
| protein transmembrane transport | 11 out of 2891 genes, 0.4% | 48 out of 15382 genes, 0.3% | 1 |  |
| positive regulation of cell growth | 28 out of 2891 genes, 1.0% | 133 out of 15382 genes, 0.9% | 1 |  |
| cellular response to lipopolysaccharide | 28 out of 2891 genes, 1.0% | 133 out of 15382 genes, 0.9% | 1 |  |
| cellular response to steroid hormone stimulus | 28 out of 2891 genes, 1.0% | 133 out of 15382 genes, 0.9% | 1 |  |
| regulation of response to biotic stimulus | 26 out of 2891 genes, 0.9% | 123 out of 15382 genes, 0.8% | 1 |  |
| cyclic nucleotide metabolic process | 12 out of 2891 genes, 0.4% | 53 out of 15382 genes, 0.3% | 1 |  |
| regulation of protein tyrosine kinase activity | 12 out of 2891 genes, 0.4% | 53 out of 15382 genes, 0.3% | 1 |  |
| determination of heart left/right asymmetry | 12 out of 2891 genes, 0.4% | 53 out of 15382 genes, 0.3% | 1 |  |
| animal organ development | 550 out of 2891 genes, 19.0% | 2867 out of 15382 genes, 18.6% | 1 |  |
| regulation of cellular carbohydrate metabolic process | 25 out of 2891 genes, 0.9% | 118 out of 15382 genes, 0.8% | 1 |  |
| methylation-dependent chromatin silencing | 3 out of 2891 genes, 0.1% | 10 out of 15382 genes, 0.1% | 1 |  |
| intracellular mRNA localization | 3 out of 2891 genes, 0.1% | 10 out of 15382 genes, 0.1% | 1 |  |
| magnesium ion homeostasis | 3 out of 2891 genes, 0.1% | 10 out of 15382 genes, 0.1% | 1 |  |
| glial cell proliferation | 3 out of 2891 genes, 0.1% | 10 out of 15382 genes, 0.1% | 1 |  |
| regulation of skeletal muscle contraction | 3 out of 2891 genes, 0.1% | 10 out of 15382 genes, 0.1% | 1 |  |
| base conversion or substitution editing | 3 out of 2891 genes, 0.1% | 10 out of 15382 genes, 0.1% | 1 |  |
| antigen processing and presentation of endogenous antigen | 3 out of 2891 genes, 0.1% | 10 out of 15382 genes, 0.1% | 1 |  |
| cell migration in hindbrain | 3 out of 2891 genes, 0.1% | 10 out of 15382 genes, 0.1% | 1 |  |
| rostrocaudal neural tube patterning | 3 out of 2891 genes, 0.1% | 10 out of 15382 genes, 0.1% | 1 |  |
| contractile actin filament bundle assembly | 3 out of 2891 genes, 0.1% | 10 out of 15382 genes, 0.1% | 1 |  |
| maintenance of gastrointestinal epithelium | 3 out of 2891 genes, 0.1% | 10 out of 15382 genes, 0.1% | 1 |  |
| negative regulation of granulocyte differentiation | 3 out of 2891 genes, 0.1% | 10 out of 15382 genes, 0.1% | 1 |  |
| negative regulation of protein homooligomerization | 3 out of 2891 genes, 0.1% | 10 out of 15382 genes, 0.1% | 1 |  |
| low-density lipoprotein receptor particle metabolic process | 3 out of 2891 genes, 0.1% | 10 out of 15382 genes, 0.1% | 1 |  |
| protein hexamerization | 3 out of 2891 genes, 0.1% | 10 out of 15382 genes, 0.1% | 1 |  |
| endosome to melanosome transport | 3 out of 2891 genes, 0.1% | 10 out of 15382 genes, 0.1% | 1 |  |
| endothelial cell activation | 3 out of 2891 genes, 0.1% | 10 out of 15382 genes, 0.1% | 1 |  |
| xenobiotic catabolic process | 3 out of 2891 genes, 0.1% | 10 out of 15382 genes, 0.1% | 1 |  |
| positive regulation of tumor necrosis factor biosynthetic process | 3 out of 2891 genes, 0.1% | 10 out of 15382 genes, 0.1% | 1 |  |
| stress fiber assembly | 3 out of 2891 genes, 0.1% | 10 out of 15382 genes, 0.1% | 1 |  |
| myelin maintenance | 3 out of 2891 genes, 0.1% | 10 out of 15382 genes, 0.1% | 1 |  |
| endosome to pigment granule transport | 3 out of 2891 genes, 0.1% | 10 out of 15382 genes, 0.1% | 1 |  |
| adhesion of symbiont to host | 3 out of 2891 genes, 0.1% | 10 out of 15382 genes, 0.1% | 1 |  |
| negative thymic T cell selection | 3 out of 2891 genes, 0.1% | 10 out of 15382 genes, 0.1% | 1 |  |
| organelle inheritance | 3 out of 2891 genes, 0.1% | 10 out of 15382 genes, 0.1% | 1 |  |
| mitochondrion distribution | 3 out of 2891 genes, 0.1% | 10 out of 15382 genes, 0.1% | 1 |  |
| lateral mesoderm development | 3 out of 2891 genes, 0.1% | 10 out of 15382 genes, 0.1% | 1 |  |
| pigment granule maturation | 3 out of 2891 genes, 0.1% | 10 out of 15382 genes, 0.1% | 1 |  |
| ventricular cardiac muscle cell development | 3 out of 2891 genes, 0.1% | 10 out of 15382 genes, 0.1% | 1 |  |
| response to platelet aggregation inhibitor | 3 out of 2891 genes, 0.1% | 10 out of 15382 genes, 0.1% | 1 |  |
| regulation of bile acid biosynthetic process | 3 out of 2891 genes, 0.1% | 10 out of 15382 genes, 0.1% | 1 |  |
| response to epinephrine | 3 out of 2891 genes, 0.1% | 10 out of 15382 genes, 0.1% | 1 |  |
| glomerular epithelial cell development | 3 out of 2891 genes, 0.1% | 10 out of 15382 genes, 0.1% | 1 |  |
| regulation of establishment of planar polarity involved in neural tube closure | 3 out of 2891 genes, 0.1% | 10 out of 15382 genes, 0.1% | 1 |  |
| dendrite extension | 3 out of 2891 genes, 0.1% | 10 out of 15382 genes, 0.1% | 1 |  |
| extracellular regulation of signal transduction | 3 out of 2891 genes, 0.1% | 10 out of 15382 genes, 0.1% | 1 |  |
| extracellular negative regulation of signal transduction | 3 out of 2891 genes, 0.1% | 10 out of 15382 genes, 0.1% | 1 |  |
| positive regulation of execution phase of apoptosis | 3 out of 2891 genes, 0.1% | 10 out of 15382 genes, 0.1% | 1 |  |
| positive regulation of endoplasmic reticulum stress-induced intrinsic apoptotic signaling pathway | 3 out of 2891 genes, 0.1% | 10 out of 15382 genes, 0.1% | 1 |  |
| protein localization to cell junction | 3 out of 2891 genes, 0.1% | 10 out of 15382 genes, 0.1% | 1 |  |
| positive regulation of amyloid precursor protein catabolic process | 3 out of 2891 genes, 0.1% | 10 out of 15382 genes, 0.1% | 1 |  |
| regulation of hexokinase activity | 3 out of 2891 genes, 0.1% | 10 out of 15382 genes, 0.1% | 1 |  |
| response to forskolin | 3 out of 2891 genes, 0.1% | 10 out of 15382 genes, 0.1% | 1 |  |
| cellular response to forskolin | 3 out of 2891 genes, 0.1% | 10 out of 15382 genes, 0.1% | 1 |  |
| regulation of GTP binding | 3 out of 2891 genes, 0.1% | 10 out of 15382 genes, 0.1% | 1 |  |
| negative regulation of morphogenesis of an epithelium | 3 out of 2891 genes, 0.1% | 10 out of 15382 genes, 0.1% | 1 |  |
| positive regulation of endothelial cell chemotaxis | 3 out of 2891 genes, 0.1% | 10 out of 15382 genes, 0.1% | 1 |  |
| nucleotide biosynthetic process | 37 out of 2891 genes, 1.3% | 179 out of 15382 genes, 1.2% | 1 |  |
| embryonic heart tube development | 13 out of 2891 genes, 0.4% | 58 out of 15382 genes, 0.4% | 1 |  |
| regulation of viral transcription | 13 out of 2891 genes, 0.4% | 58 out of 15382 genes, 0.4% | 1 |  |
| embryonic skeletal system development | 23 out of 2891 genes, 0.8% | 108 out of 15382 genes, 0.7% | 1 |  |
| response to cytokine | 162 out of 2891 genes, 5.6% | 827 out of 15382 genes, 5.4% | 1 |  |
| regulation of neural precursor cell proliferation | 14 out of 2891 genes, 0.5% | 63 out of 15382 genes, 0.4% | 1 |  |
| regulation of DNA recombination | 15 out of 2891 genes, 0.5% | 68 out of 15382 genes, 0.4% | 1 |  |
| peripheral nervous system development | 15 out of 2891 genes, 0.5% | 68 out of 15382 genes, 0.4% | 1 |  |
| cardiac muscle hypertrophy | 6 out of 2891 genes, 0.2% | 24 out of 15382 genes, 0.2% | 1 |  |
| striated muscle adaptation | 6 out of 2891 genes, 0.2% | 24 out of 15382 genes, 0.2% | 1 |  |
| protein localization to synapse | 6 out of 2891 genes, 0.2% | 24 out of 15382 genes, 0.2% | 1 |  |
| positive regulation of insulin secretion involved in cellular response to glucose stimulus | 6 out of 2891 genes, 0.2% | 24 out of 15382 genes, 0.2% | 1 |  |
| regulation of oligodendrocyte differentiation | 6 out of 2891 genes, 0.2% | 24 out of 15382 genes, 0.2% | 1 |  |
| positive regulation of neurotransmitter transport | 6 out of 2891 genes, 0.2% | 24 out of 15382 genes, 0.2% | 1 |  |
| response to misfolded protein | 6 out of 2891 genes, 0.2% | 24 out of 15382 genes, 0.2% | 1 |  |
| establishment of endothelial barrier | 6 out of 2891 genes, 0.2% | 24 out of 15382 genes, 0.2% | 1 |  |
| regulation of neuron projection regeneration | 6 out of 2891 genes, 0.2% | 24 out of 15382 genes, 0.2% | 1 |  |
| regulation of synaptic vesicle exocytosis | 6 out of 2891 genes, 0.2% | 24 out of 15382 genes, 0.2% | 1 |  |
| positive regulation of response to drug | 6 out of 2891 genes, 0.2% | 24 out of 15382 genes, 0.2% | 1 |  |
| antigen processing and presentation of peptide antigen via MHC class I | 19 out of 2891 genes, 0.7% | 88 out of 15382 genes, 0.6% | 1 |  |
| regulation of chromosome segregation | 19 out of 2891 genes, 0.7% | 88 out of 15382 genes, 0.6% | 1 |  |
| polysaccharide metabolic process | 16 out of 2891 genes, 0.6% | 73 out of 15382 genes, 0.5% | 1 |  |
| negative regulation of cell differentiation | 116 out of 2891 genes, 4.0% | 588 out of 15382 genes, 3.8% | 1 |  |
| virion assembly | 7 out of 2891 genes, 0.2% | 29 out of 15382 genes, 0.2% | 1 |  |
| regulation of mast cell activation involved in immune response | 7 out of 2891 genes, 0.2% | 29 out of 15382 genes, 0.2% | 1 |  |
| regulation of vascular permeability | 7 out of 2891 genes, 0.2% | 29 out of 15382 genes, 0.2% | 1 |  |
| regulation of mast cell degranulation | 7 out of 2891 genes, 0.2% | 29 out of 15382 genes, 0.2% | 1 |  |
| brain morphogenesis | 7 out of 2891 genes, 0.2% | 29 out of 15382 genes, 0.2% | 1 |  |
| adipose tissue development | 7 out of 2891 genes, 0.2% | 29 out of 15382 genes, 0.2% | 1 |  |
| placenta blood vessel development | 7 out of 2891 genes, 0.2% | 29 out of 15382 genes, 0.2% | 1 |  |
| regulation of viral release from host cell | 7 out of 2891 genes, 0.2% | 29 out of 15382 genes, 0.2% | 1 |  |
| negative regulation of protein localization to membrane | 7 out of 2891 genes, 0.2% | 29 out of 15382 genes, 0.2% | 1 |  |
| regulation of neuron migration | 7 out of 2891 genes, 0.2% | 29 out of 15382 genes, 0.2% | 1 |  |
| cardiac chamber development | 29 out of 2891 genes, 1.0% | 139 out of 15382 genes, 0.9% | 1 |  |
| tube morphogenesis | 59 out of 2891 genes, 2.0% | 293 out of 15382 genes, 1.9% | 1 |  |
| protein targeting | 45 out of 2891 genes, 1.6% | 221 out of 15382 genes, 1.4% | 1 |  |
| positive regulation of growth | 44 out of 2891 genes, 1.5% | 216 out of 15382 genes, 1.4% | 1 |  |
| regulation of epithelial cell proliferation | 51 out of 2891 genes, 1.8% | 252 out of 15382 genes, 1.6% | 1 |  |
| positive regulation of establishment of protein localization | 91 out of 2891 genes, 3.1% | 459 out of 15382 genes, 3.0% | 1 |  |
| regulation of peptidyl-threonine phosphorylation | 8 out of 2891 genes, 0.3% | 34 out of 15382 genes, 0.2% | 1 |  |
| cellular response to glucose starvation | 8 out of 2891 genes, 0.3% | 34 out of 15382 genes, 0.2% | 1 |  |
| negative regulation of G-protein coupled receptor protein signaling pathway | 8 out of 2891 genes, 0.3% | 34 out of 15382 genes, 0.2% | 1 |  |
| positive regulation of calcium-mediated signaling | 8 out of 2891 genes, 0.3% | 34 out of 15382 genes, 0.2% | 1 |  |
| regulation of protein targeting to membrane | 8 out of 2891 genes, 0.3% | 34 out of 15382 genes, 0.2% | 1 |  |
| negative regulation of endopeptidase activity | 24 out of 2891 genes, 0.8% | 114 out of 15382 genes, 0.7% | 1 |  |
| morphogenesis of an epithelium | 74 out of 2891 genes, 2.6% | 371 out of 15382 genes, 2.4% | 1 |  |
| carbohydrate homeostasis | 34 out of 2891 genes, 1.2% | 165 out of 15382 genes, 1.1% | 1 |  |
| glucose homeostasis | 34 out of 2891 genes, 1.2% | 165 out of 15382 genes, 1.1% | 1 |  |
| cerebellum morphogenesis | 9 out of 2891 genes, 0.3% | 39 out of 15382 genes, 0.3% | 1 |  |
| embryonic cranial skeleton morphogenesis | 9 out of 2891 genes, 0.3% | 39 out of 15382 genes, 0.3% | 1 |  |
| anion homeostasis | 9 out of 2891 genes, 0.3% | 39 out of 15382 genes, 0.3% | 1 |  |
| negative regulation of transport | 83 out of 2891 genes, 2.9% | 418 out of 15382 genes, 2.7% | 1 |  |
| second-messenger-mediated signaling | 41 out of 2891 genes, 1.4% | 201 out of 15382 genes, 1.3% | 1 |  |
| negative regulation of myeloid leukocyte differentiation | 10 out of 2891 genes, 0.3% | 44 out of 15382 genes, 0.3% | 1 |  |
| regulation of myeloid leukocyte mediated immunity | 10 out of 2891 genes, 0.3% | 44 out of 15382 genes, 0.3% | 1 |  |
| respiratory gaseous exchange | 10 out of 2891 genes, 0.3% | 44 out of 15382 genes, 0.3% | 1 |  |
| response to cold | 10 out of 2891 genes, 0.3% | 44 out of 15382 genes, 0.3% | 1 |  |
| thioester biosynthetic process | 10 out of 2891 genes, 0.3% | 44 out of 15382 genes, 0.3% | 1 |  |
| acyl-CoA biosynthetic process | 10 out of 2891 genes, 0.3% | 44 out of 15382 genes, 0.3% | 1 |  |
| regulation of peptide secretion | 82 out of 2891 genes, 2.8% | 413 out of 15382 genes, 2.7% | 1 |  |
| positive regulation of T-helper 1 type immune response | 4 out of 2891 genes, 0.1% | 15 out of 15382 genes, 0.1% | 1 |  |
| apoptotic DNA fragmentation | 4 out of 2891 genes, 0.1% | 15 out of 15382 genes, 0.1% | 1 |  |
| centriole replication | 4 out of 2891 genes, 0.1% | 15 out of 15382 genes, 0.1% | 1 |  |
| poly(A)+ mRNA export from nucleus | 4 out of 2891 genes, 0.1% | 15 out of 15382 genes, 0.1% | 1 |  |
| pentose metabolic process | 4 out of 2891 genes, 0.1% | 15 out of 15382 genes, 0.1% | 1 |  |
| positive regulation of transcription elongation from RNA polymerase II promoter | 4 out of 2891 genes, 0.1% | 15 out of 15382 genes, 0.1% | 1 |  |
| histone H2A monoubiquitination | 4 out of 2891 genes, 0.1% | 15 out of 15382 genes, 0.1% | 1 |  |
| cell death in response to oxidative stress | 4 out of 2891 genes, 0.1% | 15 out of 15382 genes, 0.1% | 1 |  |
| cytokine biosynthetic process | 4 out of 2891 genes, 0.1% | 15 out of 15382 genes, 0.1% | 1 |  |
| lipoprotein transport | 4 out of 2891 genes, 0.1% | 15 out of 15382 genes, 0.1% | 1 |  |
| myeloid dendritic cell differentiation | 4 out of 2891 genes, 0.1% | 15 out of 15382 genes, 0.1% | 1 |  |
| negative regulation of growth of symbiont in host | 4 out of 2891 genes, 0.1% | 15 out of 15382 genes, 0.1% | 1 |  |
| negative regulation of growth of symbiont involved in interaction with host | 4 out of 2891 genes, 0.1% | 15 out of 15382 genes, 0.1% | 1 |  |
| estrous cycle | 4 out of 2891 genes, 0.1% | 15 out of 15382 genes, 0.1% | 1 |  |
| lipoprotein localization | 4 out of 2891 genes, 0.1% | 15 out of 15382 genes, 0.1% | 1 |  |
| nitric oxide metabolic process | 4 out of 2891 genes, 0.1% | 15 out of 15382 genes, 0.1% | 1 |  |
| negative regulation of calcium-mediated signaling | 4 out of 2891 genes, 0.1% | 15 out of 15382 genes, 0.1% | 1 |  |
| positive regulation of amino acid transport | 4 out of 2891 genes, 0.1% | 15 out of 15382 genes, 0.1% | 1 |  |
| negative regulation of cardiac muscle tissue growth | 4 out of 2891 genes, 0.1% | 15 out of 15382 genes, 0.1% | 1 |  |
| negative regulation of heart growth | 4 out of 2891 genes, 0.1% | 15 out of 15382 genes, 0.1% | 1 |  |
| renal filtration cell differentiation | 4 out of 2891 genes, 0.1% | 15 out of 15382 genes, 0.1% | 1 |  |
| cellular response to exogenous dsRNA | 4 out of 2891 genes, 0.1% | 15 out of 15382 genes, 0.1% | 1 |  |
| cellular response to prostaglandin E stimulus | 4 out of 2891 genes, 0.1% | 15 out of 15382 genes, 0.1% | 1 |  |
| glomerular visceral epithelial cell differentiation | 4 out of 2891 genes, 0.1% | 15 out of 15382 genes, 0.1% | 1 |  |
| chromosome localization to nuclear envelope involved in homologous chromosome segregation | 4 out of 2891 genes, 0.1% | 15 out of 15382 genes, 0.1% | 1 |  |
| positive regulation of extrinsic apoptotic signaling pathway via death domain receptors | 4 out of 2891 genes, 0.1% | 15 out of 15382 genes, 0.1% | 1 |  |
| negative regulation of viral release from host cell | 4 out of 2891 genes, 0.1% | 15 out of 15382 genes, 0.1% | 1 |  |
| positive regulation of extracellular matrix organization | 4 out of 2891 genes, 0.1% | 15 out of 15382 genes, 0.1% | 1 |  |
| positive regulation of dendrite extension | 4 out of 2891 genes, 0.1% | 15 out of 15382 genes, 0.1% | 1 |  |
| positive regulation of stem cell differentiation | 4 out of 2891 genes, 0.1% | 15 out of 15382 genes, 0.1% | 1 |  |
| regulation of endothelial cell chemotaxis | 4 out of 2891 genes, 0.1% | 15 out of 15382 genes, 0.1% | 1 |  |
| protein maturation | 40 out of 2891 genes, 1.4% | 196 out of 15382 genes, 1.3% | 1 |  |
| regulation of sequestering of calcium ion | 19 out of 2891 genes, 0.7% | 89 out of 15382 genes, 0.6% | 1 |  |
| regulation of vasculature development | 47 out of 2891 genes, 1.6% | 232 out of 15382 genes, 1.5% | 1 |  |
| regulation of actin filament depolymerization | 11 out of 2891 genes, 0.4% | 49 out of 15382 genes, 0.3% | 1 |  |
| face development | 11 out of 2891 genes, 0.4% | 49 out of 15382 genes, 0.3% | 1 |  |
| purine-containing compound catabolic process | 11 out of 2891 genes, 0.4% | 49 out of 15382 genes, 0.3% | 1 |  |
| transcription elongation from RNA polymerase II promoter | 18 out of 2891 genes, 0.6% | 84 out of 15382 genes, 0.5% | 1 |  |
| regulation of immune effector process | 65 out of 2891 genes, 2.2% | 325 out of 15382 genes, 2.1% | 1 |  |
| embryonic morphogenesis | 101 out of 2891 genes, 3.5% | 512 out of 15382 genes, 3.3% | 1 |  |
| positive regulation of interferon-gamma production | 12 out of 2891 genes, 0.4% | 54 out of 15382 genes, 0.4% | 1 |  |
| cranial skeletal system development | 12 out of 2891 genes, 0.4% | 54 out of 15382 genes, 0.4% | 1 |  |
| actin-mediated cell contraction | 16 out of 2891 genes, 0.6% | 74 out of 15382 genes, 0.5% | 1 |  |
| regulation of response to drug | 16 out of 2891 genes, 0.6% | 74 out of 15382 genes, 0.5% | 1 |  |
| mesoderm formation | 13 out of 2891 genes, 0.4% | 59 out of 15382 genes, 0.4% | 1 |  |
| regulation of calcium-mediated signaling | 15 out of 2891 genes, 0.5% | 69 out of 15382 genes, 0.4% | 1 |  |
| artery development | 15 out of 2891 genes, 0.5% | 69 out of 15382 genes, 0.4% | 1 |  |
| regulation of secretion | 132 out of 2891 genes, 4.6% | 674 out of 15382 genes, 4.4% | 1 |  |
| single strand break repair | 2 out of 2891 genes, 0.1% | 6 out of 15382 genes, 0.0% | 1 |  |
| protein targeting to Golgi | 2 out of 2891 genes, 0.1% | 6 out of 15382 genes, 0.0% | 1 |  |
| chromatin silencing at rDNA | 2 out of 2891 genes, 0.1% | 6 out of 15382 genes, 0.0% | 1 |  |
| mitophagy | 2 out of 2891 genes, 0.1% | 6 out of 15382 genes, 0.0% | 1 |  |
| establishment of T cell polarity | 2 out of 2891 genes, 0.1% | 6 out of 15382 genes, 0.0% | 1 |  |
| B cell apoptotic process | 2 out of 2891 genes, 0.1% | 6 out of 15382 genes, 0.0% | 1 |  |
| store-operated calcium entry | 2 out of 2891 genes, 0.1% | 6 out of 15382 genes, 0.0% | 1 |  |
| pre-B cell differentiation | 2 out of 2891 genes, 0.1% | 6 out of 15382 genes, 0.0% | 1 |  |
| response to tumor cell | 2 out of 2891 genes, 0.1% | 6 out of 15382 genes, 0.0% | 1 |  |
| positive regulation of hypersensitivity | 2 out of 2891 genes, 0.1% | 6 out of 15382 genes, 0.0% | 1 |  |
| glycerol-3-phosphate metabolic process | 2 out of 2891 genes, 0.1% | 6 out of 15382 genes, 0.0% | 1 |  |
| activation-induced cell death of T cells | 2 out of 2891 genes, 0.1% | 6 out of 15382 genes, 0.0% | 1 |  |
| positive regulation of transcription of Notch receptor target | 2 out of 2891 genes, 0.1% | 6 out of 15382 genes, 0.0% | 1 |  |
| foregut morphogenesis | 2 out of 2891 genes, 0.1% | 6 out of 15382 genes, 0.0% | 1 |  |
| Toll signaling pathway | 2 out of 2891 genes, 0.1% | 6 out of 15382 genes, 0.0% | 1 |  |
| purine nucleoside triphosphate catabolic process | 2 out of 2891 genes, 0.1% | 6 out of 15382 genes, 0.0% | 1 |  |
| pyrimidine deoxyribonucleoside monophosphate metabolic process | 2 out of 2891 genes, 0.1% | 6 out of 15382 genes, 0.0% | 1 |  |
| pyrimidine deoxyribonucleotide biosynthetic process | 2 out of 2891 genes, 0.1% | 6 out of 15382 genes, 0.0% | 1 |  |
| negative regulation of plasminogen activation | 2 out of 2891 genes, 0.1% | 6 out of 15382 genes, 0.0% | 1 |  |
| regulation of mRNA export from nucleus | 2 out of 2891 genes, 0.1% | 6 out of 15382 genes, 0.0% | 1 |  |
| regulation of synaptic vesicle priming | 2 out of 2891 genes, 0.1% | 6 out of 15382 genes, 0.0% | 1 |  |
| skeletal muscle atrophy | 2 out of 2891 genes, 0.1% | 6 out of 15382 genes, 0.0% | 1 |  |
| transition between fast and slow fiber | 2 out of 2891 genes, 0.1% | 6 out of 15382 genes, 0.0% | 1 |  |
| N-terminal peptidyl-methionine acetylation | 2 out of 2891 genes, 0.1% | 6 out of 15382 genes, 0.0% | 1 |  |
| peptidyl-L-cysteine S-palmitoylation | 2 out of 2891 genes, 0.1% | 6 out of 15382 genes, 0.0% | 1 |  |
| peptidyl-S-diacylglycerol-L-cysteine biosynthetic process from peptidyl-cysteine | 2 out of 2891 genes, 0.1% | 6 out of 15382 genes, 0.0% | 1 |  |
| hindbrain maturation | 2 out of 2891 genes, 0.1% | 6 out of 15382 genes, 0.0% | 1 |  |
| central nervous system maturation | 2 out of 2891 genes, 0.1% | 6 out of 15382 genes, 0.0% | 1 |  |
| cerebral cortex regionalization | 2 out of 2891 genes, 0.1% | 6 out of 15382 genes, 0.0% | 1 |  |
| interkinetic nuclear migration | 2 out of 2891 genes, 0.1% | 6 out of 15382 genes, 0.0% | 1 |  |
| hyaluronan biosynthetic process | 2 out of 2891 genes, 0.1% | 6 out of 15382 genes, 0.0% | 1 |  |
| deadenylation-independent decapping of nuclear-transcribed mRNA | 2 out of 2891 genes, 0.1% | 6 out of 15382 genes, 0.0% | 1 |  |
| activation of phospholipase D activity | 2 out of 2891 genes, 0.1% | 6 out of 15382 genes, 0.0% | 1 |  |
| positive regulation of translation in response to stress | 2 out of 2891 genes, 0.1% | 6 out of 15382 genes, 0.0% | 1 |  |
| regulation of sodium:proton antiporter activity | 2 out of 2891 genes, 0.1% | 6 out of 15382 genes, 0.0% | 1 |  |
| endosome to lysosome transport via multivesicular body sorting pathway | 2 out of 2891 genes, 0.1% | 6 out of 15382 genes, 0.0% | 1 |  |
| regulation of microvillus assembly | 2 out of 2891 genes, 0.1% | 6 out of 15382 genes, 0.0% | 1 |  |
| positive regulation of glucokinase activity | 2 out of 2891 genes, 0.1% | 6 out of 15382 genes, 0.0% | 1 |  |
| histone H4-K20 trimethylation | 2 out of 2891 genes, 0.1% | 6 out of 15382 genes, 0.0% | 1 |  |
| ectopic germ cell programmed cell death | 2 out of 2891 genes, 0.1% | 6 out of 15382 genes, 0.0% | 1 |  |
| cellular triglyceride homeostasis | 2 out of 2891 genes, 0.1% | 6 out of 15382 genes, 0.0% | 1 |  |
| insulin secretion involved in cellular response to glucose stimulus | 2 out of 2891 genes, 0.1% | 6 out of 15382 genes, 0.0% | 1 |  |
| proteolysis in other organism | 2 out of 2891 genes, 0.1% | 6 out of 15382 genes, 0.0% | 1 |  |
| snRNA transcription from RNA polymerase III promoter | 2 out of 2891 genes, 0.1% | 6 out of 15382 genes, 0.0% | 1 |  |
| negative regulation of amyloid precursor protein biosynthetic process | 2 out of 2891 genes, 0.1% | 6 out of 15382 genes, 0.0% | 1 |  |
| amyloid precursor protein catabolic process | 2 out of 2891 genes, 0.1% | 6 out of 15382 genes, 0.0% | 1 |  |
| sequestering of actin monomers | 2 out of 2891 genes, 0.1% | 6 out of 15382 genes, 0.0% | 1 |  |
| histone H4-K12 acetylation | 2 out of 2891 genes, 0.1% | 6 out of 15382 genes, 0.0% | 1 |  |
| negative regulation of protein kinase activity by regulation of protein phosphorylation | 2 out of 2891 genes, 0.1% | 6 out of 15382 genes, 0.0% | 1 |  |
| relaxation of smooth muscle | 2 out of 2891 genes, 0.1% | 6 out of 15382 genes, 0.0% | 1 |  |
| regulation of interleukin-12 biosynthetic process | 2 out of 2891 genes, 0.1% | 6 out of 15382 genes, 0.0% | 1 |  |
| negative regulation of glycogen biosynthetic process | 2 out of 2891 genes, 0.1% | 6 out of 15382 genes, 0.0% | 1 |  |
| nucleobase catabolic process | 2 out of 2891 genes, 0.1% | 6 out of 15382 genes, 0.0% | 1 |  |
| embryonic foregut morphogenesis | 2 out of 2891 genes, 0.1% | 6 out of 15382 genes, 0.0% | 1 |  |
| collateral sprouting | 2 out of 2891 genes, 0.1% | 6 out of 15382 genes, 0.0% | 1 |  |
| microtubule severing | 2 out of 2891 genes, 0.1% | 6 out of 15382 genes, 0.0% | 1 |  |
| interphase microtubule nucleation by interphase microtubule organizing center | 2 out of 2891 genes, 0.1% | 6 out of 15382 genes, 0.0% | 1 |  |
| negative regulation of protein glycosylation | 2 out of 2891 genes, 0.1% | 6 out of 15382 genes, 0.0% | 1 |  |
| mammary gland involution | 2 out of 2891 genes, 0.1% | 6 out of 15382 genes, 0.0% | 1 |  |
| primitive hemopoiesis | 2 out of 2891 genes, 0.1% | 6 out of 15382 genes, 0.0% | 1 |  |
| cranial suture morphogenesis | 2 out of 2891 genes, 0.1% | 6 out of 15382 genes, 0.0% | 1 |  |
| coronary artery morphogenesis | 2 out of 2891 genes, 0.1% | 6 out of 15382 genes, 0.0% | 1 |  |
| pulmonary artery morphogenesis | 2 out of 2891 genes, 0.1% | 6 out of 15382 genes, 0.0% | 1 |  |
| negative regulation of fat cell proliferation | 2 out of 2891 genes, 0.1% | 6 out of 15382 genes, 0.0% | 1 |  |
| negative regulation of glycogen metabolic process | 2 out of 2891 genes, 0.1% | 6 out of 15382 genes, 0.0% | 1 |  |
| histone H4 deacetylation | 2 out of 2891 genes, 0.1% | 6 out of 15382 genes, 0.0% | 1 |  |
| cellular response to magnesium ion | 2 out of 2891 genes, 0.1% | 6 out of 15382 genes, 0.0% | 1 |  |
| cellular hypotonic response | 2 out of 2891 genes, 0.1% | 6 out of 15382 genes, 0.0% | 1 |  |
| response to heparin | 2 out of 2891 genes, 0.1% | 6 out of 15382 genes, 0.0% | 1 |  |
| commissural neuron axon guidance | 2 out of 2891 genes, 0.1% | 6 out of 15382 genes, 0.0% | 1 |  |
| protein localization to extracellular region | 2 out of 2891 genes, 0.1% | 6 out of 15382 genes, 0.0% | 1 |  |
| maintenance of protein location in extracellular region | 2 out of 2891 genes, 0.1% | 6 out of 15382 genes, 0.0% | 1 |  |
| determination of digestive tract left/right asymmetry | 2 out of 2891 genes, 0.1% | 6 out of 15382 genes, 0.0% | 1 |  |
| metanephric mesenchyme development | 2 out of 2891 genes, 0.1% | 6 out of 15382 genes, 0.0% | 1 |  |
| cell proliferation involved in kidney development | 2 out of 2891 genes, 0.1% | 6 out of 15382 genes, 0.0% | 1 |  |
| ureter development | 2 out of 2891 genes, 0.1% | 6 out of 15382 genes, 0.0% | 1 |  |
| clathrin coat disassembly | 2 out of 2891 genes, 0.1% | 6 out of 15382 genes, 0.0% | 1 |  |
| cellular lactam metabolic process | 2 out of 2891 genes, 0.1% | 6 out of 15382 genes, 0.0% | 1 |  |
| plus-end-directed vesicle transport along microtubule | 2 out of 2891 genes, 0.1% | 6 out of 15382 genes, 0.0% | 1 |  |
| plus-end-directed organelle transport along microtubule | 2 out of 2891 genes, 0.1% | 6 out of 15382 genes, 0.0% | 1 |  |
| response to cell cycle checkpoint signaling | 2 out of 2891 genes, 0.1% | 6 out of 15382 genes, 0.0% | 1 |  |
| response to DNA integrity checkpoint signaling | 2 out of 2891 genes, 0.1% | 6 out of 15382 genes, 0.0% | 1 |  |
| response to DNA damage checkpoint signaling | 2 out of 2891 genes, 0.1% | 6 out of 15382 genes, 0.0% | 1 |  |
| mRNA methylation | 2 out of 2891 genes, 0.1% | 6 out of 15382 genes, 0.0% | 1 |  |
| Golgi disassembly | 2 out of 2891 genes, 0.1% | 6 out of 15382 genes, 0.0% | 1 |  |
| actin filament reorganization | 2 out of 2891 genes, 0.1% | 6 out of 15382 genes, 0.0% | 1 |  |
| modulation of age-related behavioral decline | 2 out of 2891 genes, 0.1% | 6 out of 15382 genes, 0.0% | 1 |  |
| mast cell migration | 2 out of 2891 genes, 0.1% | 6 out of 15382 genes, 0.0% | 1 |  |
| regulation of hyaluronan biosynthetic process | 2 out of 2891 genes, 0.1% | 6 out of 15382 genes, 0.0% | 1 |  |
| negative regulation of interleukin-6 secretion | 2 out of 2891 genes, 0.1% | 6 out of 15382 genes, 0.0% | 1 |  |
| regulation of carbohydrate metabolic process by regulation of transcription from RNA polymerase II promoter | 2 out of 2891 genes, 0.1% | 6 out of 15382 genes, 0.0% | 1 |  |
| negative regulation of hydrogen peroxide-mediated programmed cell death | 2 out of 2891 genes, 0.1% | 6 out of 15382 genes, 0.0% | 1 |  |
| regulation of cell communication by electrical coupling involved in cardiac conduction | 2 out of 2891 genes, 0.1% | 6 out of 15382 genes, 0.0% | 1 |  |
| positive regulation of cellular respiration | 2 out of 2891 genes, 0.1% | 6 out of 15382 genes, 0.0% | 1 |  |
| negative regulation of delayed rectifier potassium channel activity | 2 out of 2891 genes, 0.1% | 6 out of 15382 genes, 0.0% | 1 |  |
| positive regulation of stem cell population maintenance | 2 out of 2891 genes, 0.1% | 6 out of 15382 genes, 0.0% | 1 |  |
| regulation of dendritic spine maintenance | 2 out of 2891 genes, 0.1% | 6 out of 15382 genes, 0.0% | 1 |  |
| regulation of ER-associated ubiquitin-dependent protein catabolic process | 2 out of 2891 genes, 0.1% | 6 out of 15382 genes, 0.0% | 1 |  |
| regulation of extracellular exosome assembly | 2 out of 2891 genes, 0.1% | 6 out of 15382 genes, 0.0% | 1 |  |
| negative regulation of production of miRNAs involved in gene silencing by miRNA | 2 out of 2891 genes, 0.1% | 6 out of 15382 genes, 0.0% | 1 |  |
| negative regulation of voltage-gated potassium channel activity | 2 out of 2891 genes, 0.1% | 6 out of 15382 genes, 0.0% | 1 |  |
| regulation of microglial cell activation | 2 out of 2891 genes, 0.1% | 6 out of 15382 genes, 0.0% | 1 |  |
| regulation of lysosomal protein catabolic process | 2 out of 2891 genes, 0.1% | 6 out of 15382 genes, 0.0% | 1 |  |
| positive regulation of T-helper 17 type immune response | 2 out of 2891 genes, 0.1% | 6 out of 15382 genes, 0.0% | 1 |  |
| regulation of glucocorticoid receptor signaling pathway | 2 out of 2891 genes, 0.1% | 6 out of 15382 genes, 0.0% | 1 |  |
| regulation of T-helper 17 cell lineage commitment | 2 out of 2891 genes, 0.1% | 6 out of 15382 genes, 0.0% | 1 |  |
| regulation of entry of bacterium into host cell | 2 out of 2891 genes, 0.1% | 6 out of 15382 genes, 0.0% | 1 |  |
| regulation of CD8-positive, alpha-beta T cell proliferation | 2 out of 2891 genes, 0.1% | 6 out of 15382 genes, 0.0% | 1 |  |
| positive regulation of Arp2/3 complex-mediated actin nucleation | 2 out of 2891 genes, 0.1% | 6 out of 15382 genes, 0.0% | 1 |  |
| regulation of histone H3-K9 acetylation | 2 out of 2891 genes, 0.1% | 6 out of 15382 genes, 0.0% | 1 |  |
| regulation of interleukin-5 secretion | 2 out of 2891 genes, 0.1% | 6 out of 15382 genes, 0.0% | 1 |  |
| negative regulation of type B pancreatic cell apoptotic process | 2 out of 2891 genes, 0.1% | 6 out of 15382 genes, 0.0% | 1 |  |
| negative regulation of cytoplasmic translation | 2 out of 2891 genes, 0.1% | 6 out of 15382 genes, 0.0% | 1 |  |
| regulation of phospholipid transport | 2 out of 2891 genes, 0.1% | 6 out of 15382 genes, 0.0% | 1 |  |
| positive regulation of phospholipid transport | 2 out of 2891 genes, 0.1% | 6 out of 15382 genes, 0.0% | 1 |  |
| positive regulation of CD8-positive, alpha-beta T cell activation | 2 out of 2891 genes, 0.1% | 6 out of 15382 genes, 0.0% | 1 |  |
| regulation of cation channel activity | 26 out of 2891 genes, 0.9% | 125 out of 15382 genes, 0.8% | 1 |  |
| maturation of LSU-rRNA | 5 out of 2891 genes, 0.2% | 20 out of 15382 genes, 0.1% | 1 |  |
| myeloid dendritic cell activation | 5 out of 2891 genes, 0.2% | 20 out of 15382 genes, 0.1% | 1 |  |
| cytokine production involved in immune response | 5 out of 2891 genes, 0.2% | 20 out of 15382 genes, 0.1% | 1 |  |
| dolichol-linked oligosaccharide biosynthetic process | 5 out of 2891 genes, 0.2% | 20 out of 15382 genes, 0.1% | 1 |  |
| axonal fasciculation | 5 out of 2891 genes, 0.2% | 20 out of 15382 genes, 0.1% | 1 |  |
| peptidyl-arginine modification | 5 out of 2891 genes, 0.2% | 20 out of 15382 genes, 0.1% | 1 |  |
| peptidyl-cysteine modification | 5 out of 2891 genes, 0.2% | 20 out of 15382 genes, 0.1% | 1 |  |
| megakaryocyte differentiation | 5 out of 2891 genes, 0.2% | 20 out of 15382 genes, 0.1% | 1 |  |
| positive regulation of protein autophosphorylation | 5 out of 2891 genes, 0.2% | 20 out of 15382 genes, 0.1% | 1 |  |
| labyrinthine layer blood vessel development | 5 out of 2891 genes, 0.2% | 20 out of 15382 genes, 0.1% | 1 |  |
| left/right pattern formation | 5 out of 2891 genes, 0.2% | 20 out of 15382 genes, 0.1% | 1 |  |
| response to cholesterol | 5 out of 2891 genes, 0.2% | 20 out of 15382 genes, 0.1% | 1 |  |
| neuron projection fasciculation | 5 out of 2891 genes, 0.2% | 20 out of 15382 genes, 0.1% | 1 |  |
| positive regulation of glycoprotein metabolic process | 5 out of 2891 genes, 0.2% | 20 out of 15382 genes, 0.1% | 1 |  |
| positive regulation of I-kappaB kinase/NF-kappaB signaling | 33 out of 2891 genes, 1.1% | 161 out of 15382 genes, 1.0% | 1 |  |
| negative regulation of multi-organism process | 33 out of 2891 genes, 1.1% | 161 out of 15382 genes, 1.0% | 1 |  |
| neuron differentiation | 173 out of 2891 genes, 6.0% | 890 out of 15382 genes, 5.8% | 1 |  |
| DNA-dependent DNA replication | 23 out of 2891 genes, 0.8% | 110 out of 15382 genes, 0.7% | 1 |  |
| regulation of leukocyte mediated immunity | 32 out of 2891 genes, 1.1% | 156 out of 15382 genes, 1.0% | 1 |  |
| cardiac chamber morphogenesis | 22 out of 2891 genes, 0.8% | 105 out of 15382 genes, 0.7% | 1 |  |
| regulation of muscle organ development | 22 out of 2891 genes, 0.8% | 105 out of 15382 genes, 0.7% | 1 |  |
| regulation of cellular ketone metabolic process | 31 out of 2891 genes, 1.1% | 151 out of 15382 genes, 1.0% | 1 |  |
| MyD88-dependent toll-like receptor signaling pathway | 6 out of 2891 genes, 0.2% | 25 out of 15382 genes, 0.2% | 1 |  |
| striated muscle hypertrophy | 6 out of 2891 genes, 0.2% | 25 out of 15382 genes, 0.2% | 1 |  |
| negative regulation of RNA splicing | 6 out of 2891 genes, 0.2% | 25 out of 15382 genes, 0.2% | 1 |  |
| modulation by host of viral transcription | 6 out of 2891 genes, 0.2% | 25 out of 15382 genes, 0.2% | 1 |  |
| multi-organism cellular process | 6 out of 2891 genes, 0.2% | 25 out of 15382 genes, 0.2% | 1 |  |
| positive regulation of nitric oxide biosynthetic process | 6 out of 2891 genes, 0.2% | 25 out of 15382 genes, 0.2% | 1 |  |
| positive regulation of DNA recombination | 6 out of 2891 genes, 0.2% | 25 out of 15382 genes, 0.2% | 1 |  |
| modulation by host of symbiont transcription | 6 out of 2891 genes, 0.2% | 25 out of 15382 genes, 0.2% | 1 |  |
| positive regulation of cell junction assembly | 6 out of 2891 genes, 0.2% | 25 out of 15382 genes, 0.2% | 1 |  |
| regulation of synaptic vesicle transport | 6 out of 2891 genes, 0.2% | 25 out of 15382 genes, 0.2% | 1 |  |
| regulation of TORC1 signaling | 6 out of 2891 genes, 0.2% | 25 out of 15382 genes, 0.2% | 1 |  |
| positive regulation of nitric oxide metabolic process | 6 out of 2891 genes, 0.2% | 25 out of 15382 genes, 0.2% | 1 |  |
| gastrulation | 29 out of 2891 genes, 1.0% | 141 out of 15382 genes, 0.9% | 1 |  |
| small molecule metabolic process | 318 out of 2891 genes, 11.0% | 1654 out of 15382 genes, 10.8% | 1 |  |
| positive regulation of epithelial cell proliferation | 28 out of 2891 genes, 1.0% | 136 out of 15382 genes, 0.9% | 1 |  |
| DNA conformation change | 42 out of 2891 genes, 1.5% | 208 out of 15382 genes, 1.4% | 1 |  |
| development of primary female sexual characteristics | 17 out of 2891 genes, 0.6% | 80 out of 15382 genes, 0.5% | 1 |  |
| regulation of intracellular estrogen receptor signaling pathway | 7 out of 2891 genes, 0.2% | 30 out of 15382 genes, 0.2% | 1 |  |
| fear response | 7 out of 2891 genes, 0.2% | 30 out of 15382 genes, 0.2% | 1 |  |
| peptide catabolic process | 7 out of 2891 genes, 0.2% | 30 out of 15382 genes, 0.2% | 1 |  |
| exonucleolytic nuclear-transcribed mRNA catabolic process involved in deadenylation-dependent decay | 7 out of 2891 genes, 0.2% | 30 out of 15382 genes, 0.2% | 1 |  |
| cAMP metabolic process | 7 out of 2891 genes, 0.2% | 30 out of 15382 genes, 0.2% | 1 |  |
| cGMP metabolic process | 7 out of 2891 genes, 0.2% | 30 out of 15382 genes, 0.2% | 1 |  |
| cardiac atrium development | 8 out of 2891 genes, 0.3% | 35 out of 15382 genes, 0.2% | 1 |  |
| ATP biosynthetic process | 8 out of 2891 genes, 0.3% | 35 out of 15382 genes, 0.2% | 1 |  |
| cellular polysaccharide biosynthetic process | 8 out of 2891 genes, 0.3% | 35 out of 15382 genes, 0.2% | 1 |  |
| substrate adhesion-dependent cell spreading | 8 out of 2891 genes, 0.3% | 35 out of 15382 genes, 0.2% | 1 |  |
| cellular modified amino acid biosynthetic process | 8 out of 2891 genes, 0.3% | 35 out of 15382 genes, 0.2% | 1 |  |
| calcium ion import | 8 out of 2891 genes, 0.3% | 35 out of 15382 genes, 0.2% | 1 |  |
| RNA stabilization | 9 out of 2891 genes, 0.3% | 40 out of 15382 genes, 0.3% | 1 |  |
| negative regulation of endocytosis | 9 out of 2891 genes, 0.3% | 40 out of 15382 genes, 0.3% | 1 |  |
| positive regulation of stress fiber assembly | 9 out of 2891 genes, 0.3% | 40 out of 15382 genes, 0.3% | 1 |  |
| positive regulation of anion transport | 9 out of 2891 genes, 0.3% | 40 out of 15382 genes, 0.3% | 1 |  |
| plasma membrane organization | 12 out of 2891 genes, 0.4% | 55 out of 15382 genes, 0.4% | 1 |  |
| interstrand cross-link repair | 10 out of 2891 genes, 0.3% | 45 out of 15382 genes, 0.3% | 1 |  |
| negative regulation of axonogenesis | 10 out of 2891 genes, 0.3% | 45 out of 15382 genes, 0.3% | 1 |  |
| regulation of glycoprotein metabolic process | 10 out of 2891 genes, 0.3% | 45 out of 15382 genes, 0.3% | 1 |  |
[truncated: 2,110,146 more chars]
