# Supplementary figures and images for: Improvement of intestinal barrier function, gut microbiota, and metabolic endotoxemia in type 2 diabetes rats by curcumin
Source: Bioengineered. 2021 Dec 19;12(2):11947–58. doi: 10.1080/21655979.2021.2009322 (PMC8810160; doi:10.1080/21655979.2021.2009322)

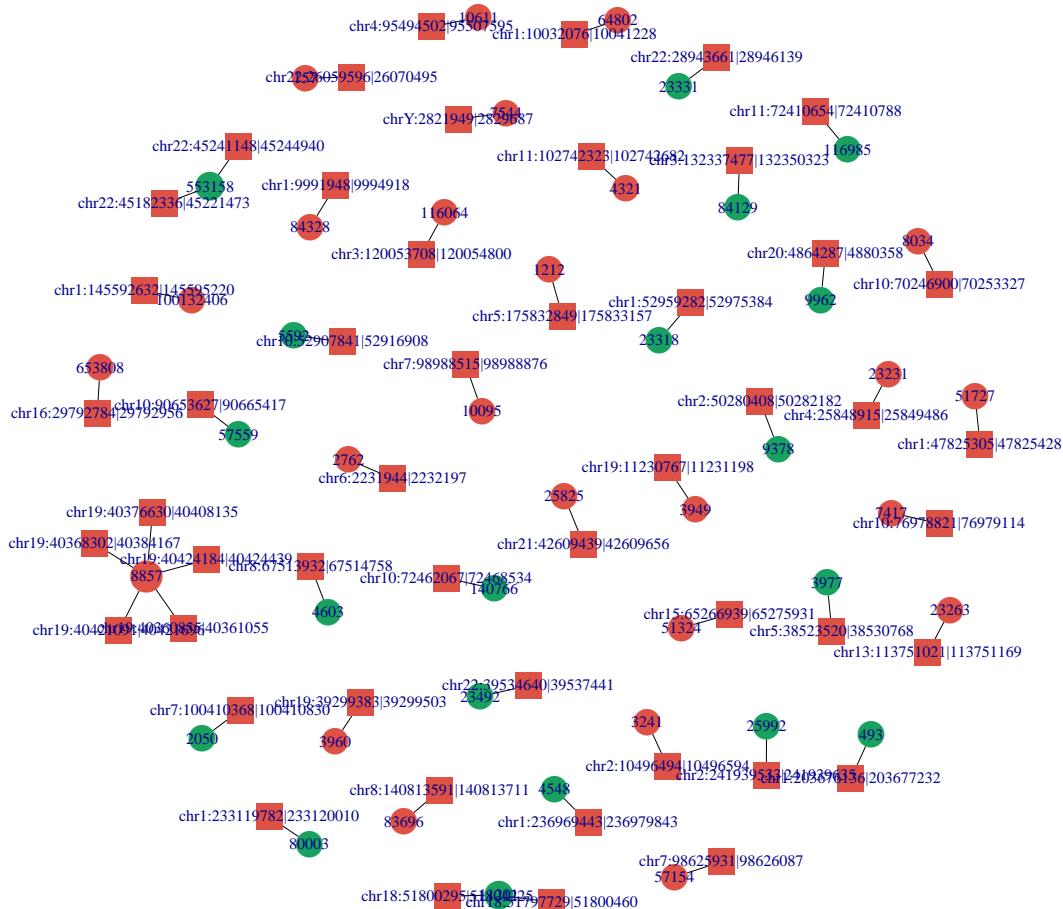

Supplement: Supplemental Material [file KBIE_A_2009322_SM6765.zip › supplementary details/DEGseq/network/network_N-VS-UC.pdf]

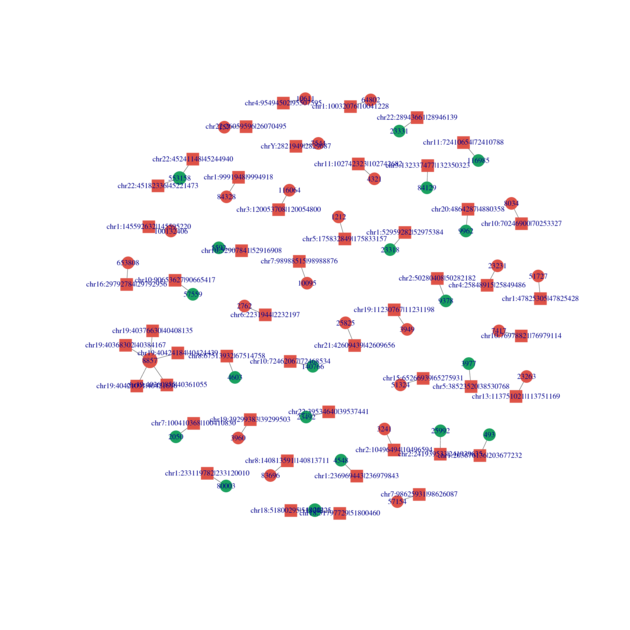

Supplement: Supplemental Material [file KBIE_A_2009322_SM6765.zip › supplementary details/DEGseq/network/network_N-VS-UC.png]

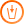

Supplement: Supplemental Material [file KBIE_A_2009322_SM6765.zip › supplementary details/DEGseq/network/network_N-VS-UC_files/vis-4.17.0/img/network/acceptDeleteIcon.png]

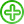

Supplement: Supplemental Material [file KBIE_A_2009322_SM6765.zip › supplementary details/DEGseq/network/network_N-VS-UC_files/vis-4.17.0/img/network/addNodeIcon.png]

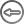

Supplement: Supplemental Material [file KBIE_A_2009322_SM6765.zip › supplementary details/DEGseq/network/network_N-VS-UC_files/vis-4.17.0/img/network/backIcon.png]

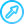

Supplement: Supplemental Material [file KBIE_A_2009322_SM6765.zip › supplementary details/DEGseq/network/network_N-VS-UC_files/vis-4.17.0/img/network/connectIcon.png]

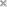

Supplement: Supplemental Material [file KBIE_A_2009322_SM6765.zip › supplementary details/DEGseq/network/network_N-VS-UC_files/vis-4.17.0/img/network/cross.png]

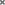

Supplement: Supplemental Material [file KBIE_A_2009322_SM6765.zip › supplementary details/DEGseq/network/network_N-VS-UC_files/vis-4.17.0/img/network/cross2.png]

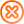

Supplement: Supplemental Material [file KBIE_A_2009322_SM6765.zip › supplementary details/DEGseq/network/network_N-VS-UC_files/vis-4.17.0/img/network/deleteIcon.png]

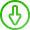

Supplement: Supplemental Material [file KBIE_A_2009322_SM6765.zip › supplementary details/DEGseq/network/network_N-VS-UC_files/vis-4.17.0/img/network/downArrow.png]

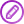

Supplement: Supplemental Material [file KBIE_A_2009322_SM6765.zip › supplementary details/DEGseq/network/network_N-VS-UC_files/vis-4.17.0/img/network/editIcon.png]

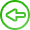

Supplement: Supplemental Material [file KBIE_A_2009322_SM6765.zip › supplementary details/DEGseq/network/network_N-VS-UC_files/vis-4.17.0/img/network/leftArrow.png]

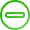

Supplement: Supplemental Material [file KBIE_A_2009322_SM6765.zip › supplementary details/DEGseq/network/network_N-VS-UC_files/vis-4.17.0/img/network/minus.png]

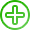

Supplement: Supplemental Material [file KBIE_A_2009322_SM6765.zip › supplementary details/DEGseq/network/network_N-VS-UC_files/vis-4.17.0/img/network/plus.png]

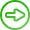

Supplement: Supplemental Material [file KBIE_A_2009322_SM6765.zip › supplementary details/DEGseq/network/network_N-VS-UC_files/vis-4.17.0/img/network/rightArrow.png]

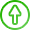

Supplement: Supplemental Material [file KBIE_A_2009322_SM6765.zip › supplementary details/DEGseq/network/network_N-VS-UC_files/vis-4.17.0/img/network/upArrow.png]

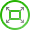

Supplement: Supplemental Material [file KBIE_A_2009322_SM6765.zip › supplementary details/DEGseq/network/network_N-VS-UC_files/vis-4.17.0/img/network/zoomExtends.png]

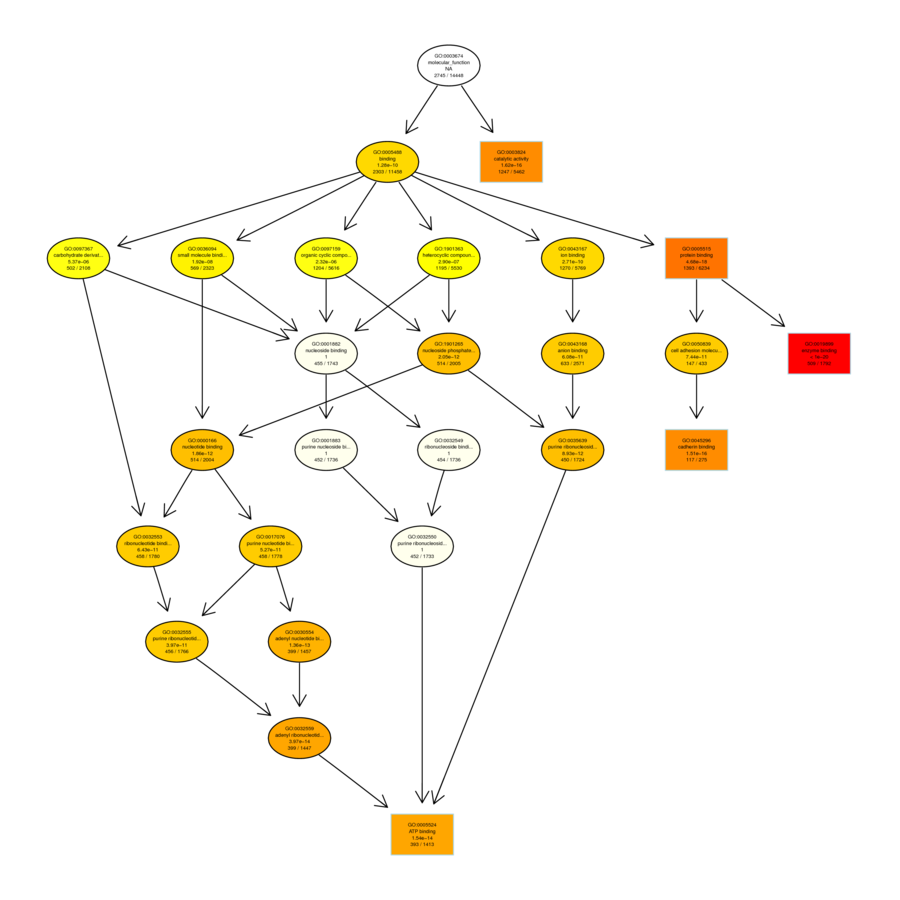

Supplement: Supplemental Material [file KBIE_A_2009322_SM6765.zip › supplementary details/N-VS-UC.DEGseq_Method.Molecular_Function.topGO.png]
